# Sera from women with different metabolic and menopause states differentially regulate cell viability and Akt activation in a breast cancer *in-vitro* model

**DOI:** 10.1371/journal.pone.0266073

**Published:** 2022-04-12

**Authors:** Laura C. Flores-García, José L. Ventura-Gallegos, Sandra L. Romero-Córdoba, Alfredo J. Hernández-Juárez, María A. Naranjo-Meneses, Eduardo García-García, Juan Pablo Méndez, Alberto J. Cabrera-Quintero, Antonio Ramírez-Ruíz, Sigifredo Pedraza-Sánchez, Noemi Meraz-Cruz, Felipe Vadillo-Ortega, Alejandro Zentella-Dehesa

**Affiliations:** 1 Departamento de Medicina Genómica y Toxicología Ambiental, Instituto de Investigaciones Biomédicas (IIBO), Universidad Nacional Autónoma de México (UNAM), Mexico City, Mexico; 2 Unidad de Bioquímica, Instituto Nacional de Ciencias Médicas y Nutrición Salvador Zubirán (INCMNSZ), Mexico City, Mexico; 3 Programa Institucional de Cáncer de Mama, IIBO, UNAM, Mexico City, Mexico; 4 Clínica de Obesidad y Trastornos de la Conducta Alimentaria, Instituto Nacional de Ciencias Médicas y Nutrición Salvador Zubirán (INCMNSZ), Mexico City, Mexico; 5 Unidad de Investigación en Obesidad, Instituto Nacional de Ciencias Médicas y Nutrición Salvador Zubirán (INCMNSZ), Mexico City, Mexico; 6 Unidad de Vinculación Científica de la Facultad de Medicina, Universidad Nacional Autónoma de México en el Instituto Nacional de Medicina Genómica, Mexico City, Mexico; King Faisal Specialist Hospital and Research Center, SAUDI ARABIA

## Abstract

Obesity is associated with an increased incidence and aggressiveness of breast cancer and is estimated to increment the development of this tumor by 50 to 86%. These associations are driven, in part, by changes in the serum molecules. Epidemiological studies have reported that Metformin reduces the incidence of obesity-associated cancer, probably by regulating the metabolic state. In this study, we evaluated in a breast cancer in-vitro model the activation of the IR-β/Akt/p70S6K pathway by exposure to human sera with different metabolic and hormonal characteristics. Furthermore, we evaluated the effect of brief Metformin treatment on sera of obese postmenopausal women and its impact on Akt and NF-κB activation. We demonstrated that MCF-7 cells represent a robust cellular model to differentiate Akt pathway activation influenced by the stimulation with sera from obese women, resulting in increased cell viability rates compared to cells stimulated with sera from normal-weight women. In particular, stimulation with sera from postmenopausal obese women showed an increase in the phosphorylation of IR-β and Akt proteins. These effects were reversed after exposure of MCF-7 cells to sera from postmenopausal obese women with insulin resistance with Metformin treatment. Whereas sera from women without insulin resistance affected NF-κB regulation. We further demonstrated that sera from post-Metformin obese women induced an increase in p38 phosphorylation, independent of insulin resistance. Our results suggest a possible mechanism in which obesity-mediated serum molecules could enhance the development of luminal A-breast cancer by increasing Akt activation. Further, we provided evidence that the phenomenon was reversed by Metformin treatment in a subgroup of women.

## Background

Obesity has become a global epidemic and is an important risk factor for the development of different types of tumors, including breast cancer **[1, 2]**. According to GLOBOCAN 2018 **[3]**, 2 088 849 new cases and 626 679 breast cancer deaths were reported worldwide, representing 46.3% of all female neoplasms, making breast cancer a relevant public health problem.

Relevantly, obesity, among other intrinsic and non-intrinsic risk factors, increases the risk of developing breast cancer by 50 to 86%, depending on the degree of obesity in women **[2]**. Likewise, obese women have a 41% risk of dying compared to normal-weight women, associated with a 46% increased risk of developing metastasis in these women **[2]**.

Several serum molecules associated with obesity provide a molecular link to breast cancer, including increased circulating levels of insulin, glucose, hormones, adipokines, and inflammatory mediators that could affect breast tissue. For example, by altering relevant oncogenic pathways such as the phosphoinositide 3-kinase (PI3K)/protein kinase B (Akt) signaling, which is activated by insulin, Insulin-like Growth Factor 1 (IGF-1), estrogens, progesterone, leptin, among others, which increases the risk of breast cancer development **[4–17]**.

The Insulin Receptor β (IR-β)/PI3K/Akt signaling pathway begins with insulin/IGF-1 binding to IR-β allowing its activation and PI3K coupling, generating the Phosphoinositide-dependent kinase-1 (PDK1), Phosphoinositide-dependent kinase-2 (PDK2) and Akt recruitment **[18–20]**. This promotes Akt activation, resulting in the phosphorylation of mammalian Target of Rapamycin (mTOR) and subsequent Ribosomal protein S6 kinase beta-1 (p70S6K) activation, inducing the translation of proteins that affect carcinogenesis **[20–22]**. Therefore, the inhibition of the PI3K/Akt pathway is a relevant therapeutic target.

Interestingly, Metformin, a drug used to treat diabetes mellitus and metabolic control in obese patients, has recently shown an antitumor effect through IR-β and Akt inhibition **[23–25]**. Moreover, clinical experience and epidemiological associations have reported a lower incidence of invasive breast cancer in people with Metformin treatment **[26, 27]**. It has also been reported that Metformin exerts an effect in the serum of treated patients by reducing circulating levels of glucose, insulin, IGF-1, and triglycerides **[28–30]**.

In the present study, we used a luminal A breast cancer *in vitro* model (MCF-7 cells) exposed to sera from pre-and postmenopausal women with different body mass indexes (BMIs). This allowed us to investigate how the systemic factors associated with obesity affect Akt in a tumoral cell model. Our analysis revealed an early and sustained increase in Akt expression and activation after exposing MCF-7 cells with sera from obese women, resulting in a higher viability rate. These effects were reversed after exposure of MCF-7 cells to sera from obese women with insulin resistance who received a brief treatment with Metformin (10 weeks).

Our findings provide information on a possible mechanism in which obesity may contribute to the development of luminal A subtype breast cancer and that Metformin treatments reverses this effect in a subgroup of women.

## Materials and methods

### Public transcriptomic and proteomic data

TGCA transcriptomic and reverse-phase protein arrays (RPPA) data were downloaded from the public repository Xena Browser (https://xenabrowser.net/datapages, GDC TCGA and TCGA Breast Cancer). RNA-seq expression data were processed from RNA-seq counts, normalized, and compared for differentially expressed patterns through the Deseq2 R/Bioconductor package **[31]**. Proteomic data generated by RPPA, using 225 antibodies targeting total (n = 166), cleaved (n = 2), acetylated (n = 1) and phosphorylated (n = 56) proteins, were retrieved at normalized level 3.

Raw microarray data were downloaded from the Gene Expression Omnibus (GEO) (GSE102088, GSE789588 and GSE24185). The signal intensities of the Affymetrix Arrays were background corrected by RMA and normalized by quantile algorithm with the Oligo Bioconductor library on R environment **[32]**. Affymetrix probes were mapped with biomaRt **[33]** R package, and duplicated genes were collapsed by selecting the probe with the higher interquartile range. Normalized data from GSE33526 were directly downloaded from GEO and processed for further analysis.

### PI3K/AKT/mTOR pathway signatures

We applied public signatures described by Zhang *et al*. **[34]**. AKT, PI3K and mTOR pathway signature were scored based on the RPPA data, computed as the sum of normalized phosphoprotein levels of Akt (S473 and T308 RPPA expression levels), GSK3 (S9 and S21/S9), and PRAS40 pT246. Additionally, mTOR pathway signature included mTOR, 4EBP1 (S65, T37/T46, and T70), RICTOR (T3135) and S6 (S235/S236 and S240/S244) phospho-proteins. Each signature was defined as the sum of phosphoprotein levels evaluated for each pathway.

For gene transcriptional signatures of the AKT pathway we first defined the differentially expressed genes between TCGA protein phosphorylated samples vs non-phosphorylated samples, for each of the Akt phospho-protein (AktS473 and T308). Then, a correlation analysis between messenger RNA (mRNA) and protein level was performed. Only those genes differentially expressed and positive or negative correlated (p≤0.05) were included in the final signature.

For a given gene transcription signature, we extracted the normalized expression values, then: (1) mean values were independently computed for “up” (μUP) and “down-modulated” genes (μDW). We later divided mean of up-regulated genes by mean of down-modulated gene (2) score = 1, (3). We finally scale score 1 values by z-score (Final score) among the total number of samples in each of the evaluated cohorts. Once mRNA signatures were determined, a Wilcoxon analysis was performed between the molecular signatures. Data were plotted with ggplot on R.

### Blood samples

Blood samples under the BQO-2044-17-18-1 protocol were obtained, approved by the Research Ethics Committee and the Research Committee of the Instituto Nacional de Ciencias Médicas y Nutrición Salvador Zubirán (INCMNSZ), following the Declaration of Helsinki and good clinical practices. We obtained the informed consent before participation. To maintain confidentiality samples were coded. The donors were grouped according to the BMI category (normal weight 20–24.9 kg/m^2^, stage I obesity 30–34.9 kg/m^2^, stage II obesity 35–39.9 kg/m^2^ and stage III obesity ≥ 40 kg/m^2^). Blood samples were centrifuged at 3000 rpm for 30 min at 4°C. The serum obtained was labeled and stored at -70°C until use.

For identification of the cellular model, we used sera from pre and postmenopausal normal-weight and obese women. For Metformin treatment we carried out a prospective controlled, non-randomized, and single-blind study, which included obese patients without a diagnosis of diabetes mellitus, who received 2550 mg/day of Metformin for ten weeks. Using the G Power program (version 3.19.4), we determined a sample size of 10 sera per group to ensure a one-tail test with α = 0.05 and 80% statistical power (S8 Fig).

### Cell line

Cell line MCF-7 (HTB-22) from ATCC was used. The MCF-7 line was maintained in RPMI-1640 culture medium supplemented with 10% FBS at 37°C, 5% CO_2_ in a humidified atmosphere.

### Reagents and antibodies

RPMI-1640 culture medium and Fetal Bovine Serum were purchased from Gibco-Thermo Fisher Scientific. RPMI-1640 medium without phenol red, sodium chloride, potassium chloride, disodium phosphate, monopotassium phosphate, Nonidet 40, SDS, Na3VO4, PMSF, NaF, cOmplete cocktail 25x, crystal violet, EDTA, methanol, glycine, Tris-Base, NaOH, glutaraldehyde, acetic acid, beta-mercaptoethanol, bromophenol blue, glycerol, ammonium persulfate, TEMED, Tween-20, 1,1-Dimethylbiguanide hydrochloride, wortmannin, genistein were from Sigma Aldrich. 30% Acrylamide/Bisacrylamide Solution and Quick Start Bradford Protein Assay Dye Reagent from Biorad. Bortezomib was from Sandoz. Fast-acting recombinant human insulin (100 UI/ml) of PiSA. Recombinant human TNF-α of R&D Systems. Antibodies against IR-β (sc-81465), pIR-β (Tyr 1162–1163) (sc-25103), Akt-1 (sc-1618-R), pAkt (Ser 473) (sc-81433), p70S6K (sc-8418), pp70S6K (Thr 389) (sc-8416), Erk (sc-271269), pp38 (Tyr 182) (sc-166182), p38 (sc-7972), p65 (sc-372) and β-Actin (sc-47778) were from Santa Cruz Biotechnology. Antibody to pp65 (Ser 536) (93H1) and pErk (Thr 202-Tyr 204) (4370) of Cell Signaling Technology. Antibody to IKB-α (610690) of BD Transduction Laboratory. Anti-Mouse HRP and anti-Rabbit HRP secondary antibodies were of Invitrogen-Thermo Fisher Scientific. Oligos for RT-PCR were synthesized by Integrated DNA Technologies (IDT).

### Fetal bovine serum reduction protocol

To sensitize the cells to human serum, a gradual reduction of FBS was carried out in all the experiments, as reported by Arellano-Plancarte *et al*. (2010) **[35]**. Cells were seeded with RPMI-1640 medium with 10% FBS for 24 h, then changed to RPMI-1640 medium with 2% FBS for 16 h. Finally, the cells were changed to RPMI-1640 medium without phenol red with 0% FBS for 4 h before starting the experiment.

### Viability assay

In culture plates, we sowed 15 000 cells/cm^2^, which were subjected to the FBS reduction process. Subsequently, MCF-7 cells were exposed for 48 h to RPMI medium supplemented with 10% FBS, 5% FBS, or 5% human sera. After the exposure time, the cells were fixed for 15 min with DMEM medium containing 2% FBS and 1% glutaraldehyde. Afterwards the cells were dyed with 0.5% crystal violet for 15 min and the absorbed dye was dissolved in 500 μl of 10% acetic acid. Absorbance was measured at 590 nm using a microplate reader (SkanIt Re).

### Western blot

The cells were seeded in 100 mm cell culture dishes at the same cell density described in the viability assay and subjected to FBS reduction. After serum deprivation, RPMI-1640 medium without phenol red was added with 0.5 UI/ml insulin for 10 min or 5% human serum (normal-weight or obese, pre or postmenopausal, with or without Metformin treatment) for 5, 10, 30 or 60 min. For the assay with inhibitors, cells were pre-incubated for 30 min with 200 mM Genistein, 100 nM Wortmannin or 80 nM Bortezomib. For NF-*κ*B signaling, 10 ng/ml of TNF-α was used as a positive activation control. After the stimulation time elapsed, protein extraction was performed using RIPA lysis buffer with protease and phosphatase inhibitors. The protein concentration was determined by the Bradford method. Samples were electrophoresed on 8 or 12% acrylamide SDS-PAGE gels and transferred to Immobilon-P PVDF membranes from Merck Millipore in a wet transfer system. The transferred membranes were incubated overnight at 4°C with primary antibodies against pIR-β (Tyr 1162–1163), IR-β, pAkt (Ser 473), Akt-1, pp70S6K (Thr 389), p70S6K, IKBα, p65, pp65 (Ser 536), Erk, pErk (Thr 202-Tyr 204), pp38 (Tyr 182), p38 and β-Actin. Primary antibodies were washed with TBS-Tween-20 before adding secondary antibodies for 30 min. Subsequently, the horseradish peroxidase-conjugated secondary antibodies were added for 1 h at room temperature. The bands were revealed by Super Signal West Pico PLUS chemiluminescent Substrate kit (Thermo Scientific). Images were obtained using the Fusion Fx imaging system from Vilber Lourmant; densitometry was quantified using Image J software (NIH, Bethesda, Maryland, USA). The densitometric analysis was performed first adjusting with β-Actin both total and phosphorylated protein and then phosphorylated protein was adjusted with total protein.

### RT-PCR

Cells were seeded as described in the viability assay and subjected to the FBS reduction protocol described above. Stimulation with 5% human sera in RPMI-1640 without phenol red was carried out for 10 min. After the stimulation time, total RNA was isolated using Tripure Isolation Reagent (Roche) and quantified on the SkanIt RE spectrophotometer. RNA integrity was determined using a denaturing formaldehyde-agarose gel. Reverse transcription was performed with the M-MLV Reverse Transcriptase system (Promega) that lasted 5 min at 70°C and 45 min at 42°C. PCR was performed using the Hot Start Master Mix Kit on Thermal Cycler-100 (MJ Research) starting at 95°C for 15 min, followed by 30 seconds at 94°C, 1 minute at 52°C and 1 min at 72°C per cycle, finally at 72°C for 10 min. The primer sequences were as follows:

**Table:** 

Primer	Sequence	TM	# Cycles	Amplicon
Akt-1 Forward	5’-TCAAGAATGATGGCACCTTCATTG-3’	52°C	32	1008
Akt-1 Reverse	5’-CCTCCATGAGGATGAGCTCAAAAA-3’			
Akt-2 Forward	5’-ATGAATGAGGTGTCTGTCATCAAAG-3’	52°C	32	856
Akt-2 Reverse	5’-CTTTGTCCAGCATGAGGTTTT-3’			
Akt-3 Forward	5’-TTGTGAAAGAAGGTTGGGTTCAG-3’	52°C	32	552
Akt-3 Reverse	5’-CTTCATCCTTTGCAATAATGACTTC-3’			
PPIA Forward	5’-GTTTACCCCTGATCGTGCAGCAG-3’	52°C	32	475
PPIA Reverse	5’-CGAGTTGTCCACAGTCAGCAATG-3’			

The PCR products were separated on 1.5% agarose gels in TAE, soaked with ethidium bromide, and the base pair marker pUCmix was used. The amplicons were imaged using a Vilber Lourmat Fusion Fx imaging system and the bands were quantified using Image J software (NIH, Bethesda, Maryland, USA).

### ELISA Bio-PLEX

To determine the concentration of serum molecules, we took 100 μl of serum from each patient treated with Metformin at 0 weeks (0W) and 10 weeks (10W) of treatment. Samples were added in triplicate to 96-well plates containing polystyrene beads from the 39-analyte assay kit (Millipore MILLIPLEX that includes: interferon γ (IFN-γ), interleukin 1α (IL-1α), interleukin 1β (IL-1β), IL-1 receptor antagonist (IL-ra), interleukin 2 (IL-2), interleukin 4 (IL-4), interleukin 6 (IL-6), interleukin 8 (IL-8), interleukin 10 (IL-10), interleukin 17 (IL-17), monocyte chemotactic protein 1 (MCP-1), macrophage inflammatory protein 1α (MIP-1α), inflammatory protein of macrophages 1β (MIP-1β), tumor necrosis factor α (TNF-α), vascular endothelial growth factor (VEGF), interleukin 12p40 (IL-12p40), interleukin 7 (IL-7), eotaxin 1 (CCL11), protein 10 induced by interferon gamma (IP-10), interleukin 2 receptor antagonist (IL-2ra), interleukin 3 (IL-3), interleukin 12p70 (IL-12p70), interleukin 16 (IL-16), interleukin 18 (IL-18), CC motif chemokine ligand 27 (CTACK), GROa, hepatocyte growth factor (HGF), monocyte chemotactic protein 3 (MCP-3), Leukemia inhibitory factor (LIF), interferon α-2 (IFN- α2), macrophage colony stimulating factor (M-CSF), macrophage migration inhibitory factor (MIF), monokine induced by interferon-gamma (MIG), nerve growth factor (b-NFG), stem cell factor (SCF), stem cell growth factor β (SCGF-β), stromal cell-derived factor 1 (SDF-1), tumor necrosis factor β (TNF-β) and tumor necrosis factor-related apoptosis inducing ligand (TRAIL). After incubation, the beads were washed twice with Bio-Plex buffer and were retained through a filter using a vacuum manifold (Millipore, Bedford, MA). A standard curve was prepared for each of the 39 human analytes in a concentration range of 0.2 to 32,000 pg/mL and added to the antibody-conjugated beads. Plates with beads, sera, and standards were incubated in the dark on a platform shaker for 30 min. After incubation, the solutions with the sera and standards were vacuum aspirated, and again, the beads were washed three times with Bio-Plex wash buffer and retained by filtration. A 1:50 dilution of biotinylated detection antibody was then added to the washed beads, followed by incubation in the dark on a platform shaker for 30 min. Once again, the beads were washed three times and incubated with a 1:100 dilution of streptavidin-phycoerythrin (PE) for 10 min. The beads were washed three times as described above, re-suspended in Bio-Plex assay buffer, and analyzed on a Bio-Plex plate reader.

### Statistical analysis

For statistical analysis, we used one or two-way ANOVA with Dunnet, LSD, or Games-Howell post-test, using SPSS version 25 (Armonk, NY: IBM Corp.), p <0.05 was considered significant. Data were plotted with ggplot on R.

## Results

### In silico exploration of Akt phosphorylation through gene expression signatures in normal and tumor breast tissue of pre and postmenopausal women with different BMIs

Due to the lack of information on the use of tumor cells for human sera comparison with different metabolic characteristics and considering the large number of altered molecules associated with obesity, we decided to focus on the evaluation of the activation of the IR-β/Akt axis. Therefore, Akt protein was our starting point to determine if there was a differential activation due to BMI differences. On account of this, our objective was to perform an in-silico analysis to compare Akt expression and activation in breast-normal and tumoral tissues from women with different BMIs.

First, we examined The Cancer Genome Atlas (TCGA) datasets from pre-menopause (<49 years old, n = 243) breast cancer patients with available gene expression information and reverse-phase protein array (RPPA) data, which evaluated the expression of a set of proteins and phosphorylated modifications. RPPA publicly available signatures for PI3K/AKT and mTOR, as well as their summarization in activity scores, were applied (S1A Fig) **[34]**. We observed a high correlation between AKT protein-derived signature and the Akt (Ser 473 and Thr 308) phospho-protein levels (R~95%, p≤0.05). Similarly, PI3K/AKT activity scores were highly correlated (R:0.84, p<0.05), with the Akt protein signature, while mTOR activity score showed a more moderate correlation (R: 0.29, p<0.05) (S1B Fig). Through this analysis, we defined a correlative relationship that enabled us to examine human breast tumors correlations in the context of PI3K/AKT/mTOR axis.

As a means of identifying a transcriptional (mRNA) signature associated with the PI3K/*Akt*(1,2,3) phosphorylated state to greater extend our analysis in the setting of human breast tissues under different BMI biological contexts, we took advantage of previously reported signatures **[22]** and an in-house transcriptional signature, developed based on messenger RNA genes up-modulated or down-modulated in tumors with high levels of Akt (Ser 473) and Akt (Thr 308) phosphorylation (S1 Table). We observed a strong significant correlation between the PI3K/AKT transcriptional signatures and PI3K/Akt phosphorylation state measured by RPPA (S1C Fig), as well as a differential expression pattern between samples with high vs low levels of Akt (Ser 473) and Akt (Thr 308), both at protein and mRNA level (S1D Fig). Multiomic characterization of TCGA samples and their integration through molecular signatures allowed us to identify transcriptional patterns to assess the role of PI3K/AKT/mTOR pathway activation. Most of the correlations observed in our analysis are in line with the understanding of PI3K/AKT/mTOR signaling. Above all, we conclude that the derivate mRNA-signatures highly correlate with and described Akt phosphorylation status.

**Fig 1:**
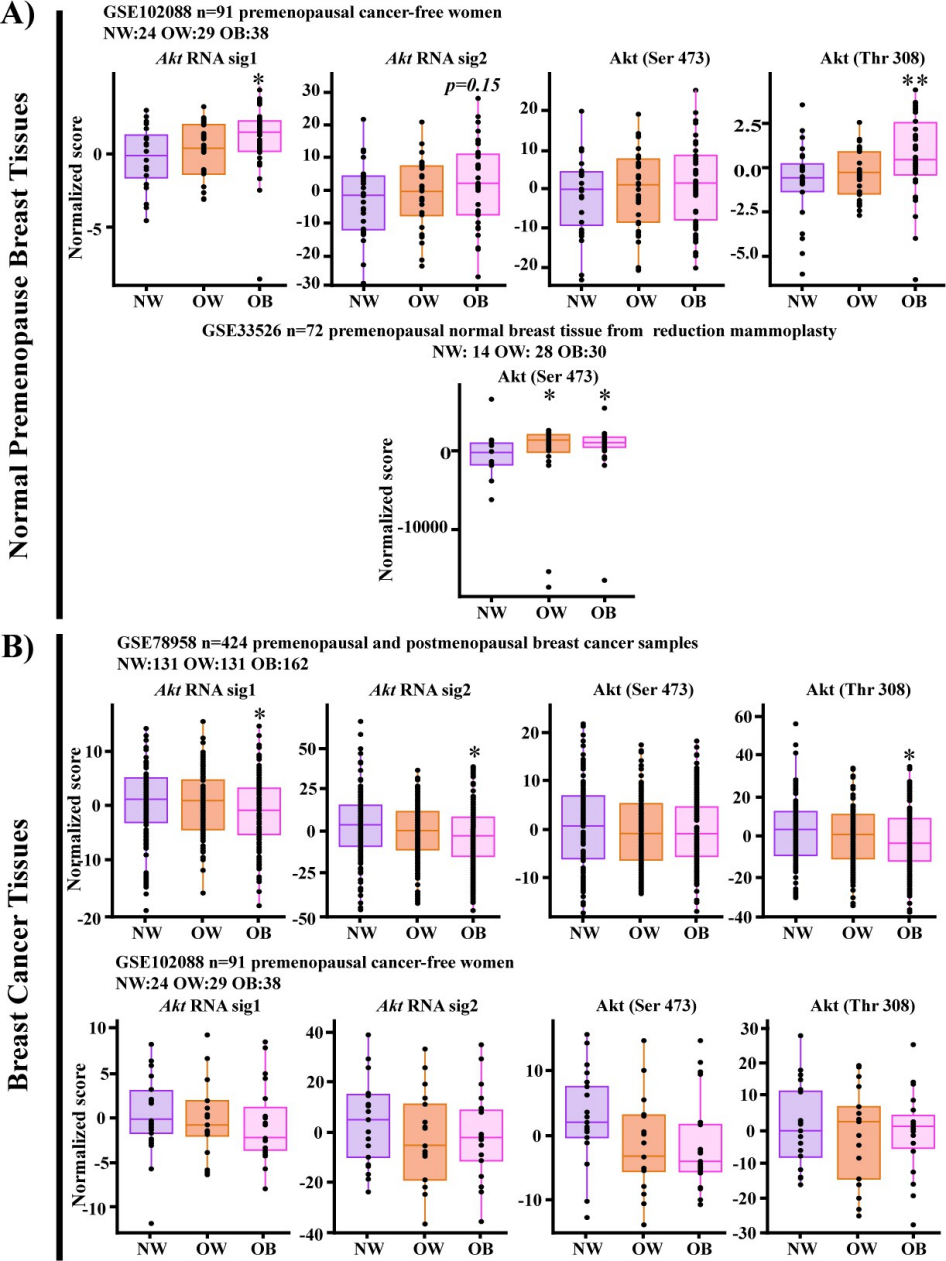
Akt phosphorylation landscape evaluated through gene expression signatures in non-tumoral or tumor breast tissues of women with different BMI. Bioinformatic analysis of the mRNA gene expression signatures that mirrored the phosphorylation of Akt in Ser 473 and Thr 308 in public datasets recovered from the GEO database in breast tissue of normal-weight (NW), overweight (OW) and obese (OB) women. A) RNA gene signature scores describing Akt activation (Akt sig 1 –in-house signature- and sig 2) or Akt phosphorylation in non-tumoral breast tissue from premenopausal women. B) RNA gene signature scores describing Akt activation (Akt sig 1–in-house signature- and sig 2) or Akt phosphorylation in tumors of pre and postmenopausal women (upper panel) or breast tumors from premenopausal women (lower panel).*<0.05 **<0.005 comparisons between groups.

For statistical analysis, those patients with available BMI information were classified as normal-weight (BMI<25 kg/m^2^), overweight (BMI 25–29.9 kg/m^2^) or obese (BMI≥30 kg/m^2^). Through bioinformatic analysis of the transcriptional scores competed with the above-described signatures, we observed that normal tissue of premenopausal obese women presented a significant up-modulation of Akt activation, and increased phosphorylation levels of Akt (Ser 473) and Akt (Thr 308) (p<0.05), corroborated in two datasets of normal mammary gland tissue (GSE102088 and GSE33526, n = 163) (Fig 1A).

Furthermore, when scoring breast tumor tissues (GSE789588, n = 424) for the above signatures, divided or not based on inferred premenopausal status (<49 years old), a substantial fraction of obese cancer patients showed lower Akt activation scores (Fig 1B upper panel) (p<0.05), in comparison to normal-weight or overweight individuals, in contrast to what was observed in normal breast epithelium. However, this trend was not corroborated in independent data and no significant differences were observed among the BMI groups (Fig 1B lower panel), suggesting multiple heterogeneous mechanisms for Akt pathway activation.

Overall, these data highlight the possible role of Akt activation and phosphorylation mediated by obese-derived molecules and its potential impact on the normal mammary epithelium, which resulted enriched in Akt-activated states that might trigger oncogenic programs, a phenomenon that needs to be further investigated.

### MCF-7 cell line as a response model to human sera

Our bioinformatics analysis revealed a differential expression and phosphorylation in Akt protein modulated by BMI (Fig 1). We hypothesized that the exposure of tumor cells to human sera could lead us to identify biological changes due to molecules altered by the presence of obesity.

There is limited information about the use of human sera in culture of human breast cancer cell lines **[5, 36–38]**. Whereby, we first selected an optimal cell model that allows us to compare the effect of sera from normal-weight and obese women. To preserve the effect of heat-sensitive serum molecules that could affect the IR-β/Akt pathway, we began by evaluating the use of human serum with or without heat-inactivation (S2A Fig), as well as tolerance to human serum without heat-inactivation (S2B Fig). On the other hand, although the IR-β/Akt/p70S6K pathway has a relevant role in breast cancer, we did not know which lines in our panel would present activation of the signaling pathway with human sera. Therefore, we also tested the activation of the PI3K/Akt signaling pathway by insulin (Ins) or serum from a normal-weight premenopausal woman (NWSPre) (S3 Fig). Finally, we use cell viability as a way to differentiate sera with different BMI (S4 Fig). Of the 9 human tumor cell lines tested, the MCF-7 line proved to be the most robust cell model for evaluating human sera, so we decided to use this line as our *in vitro* model.

### Evaluation of MCF-7 cells in response to human sera with different BMIs

According to our bioinformatics analysis, in premenopausal women, it was observed that the presence of obesity was associated with an increase in Akt’s expression and phosphorylation. To corroborate if our cellular model could differentiate between sera from women with different BMI, we initially tested the cellular and molecular changes on MCF-7 cells when exposed to sera from premenopausal women.

**Fig 2:**
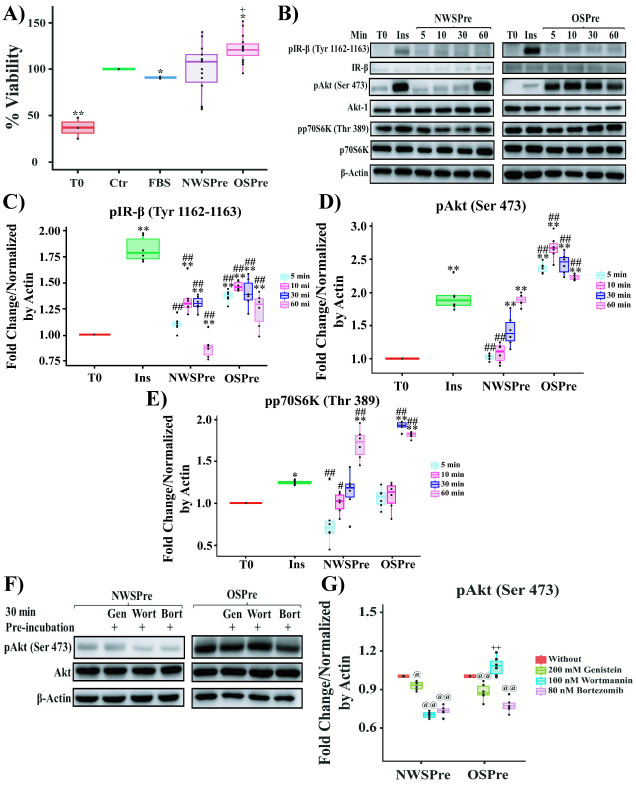
Effect of human sera on MCF-7 line. **A)** Viability of MCF-7 cells stimulated with sera from women with different BMI. **B)** Western Blot of the time course of IR-β/Akt/p70S6K pathway activation under stimulation with sera from women with different BMI. **C)** Group analysis of densitometric quantification stratified by sera condition of pIR-β (Tyr 1162–1163). **D)** Group analysis of densitometric quantification stratified by sera condition of pAkt (Ser 473). **E)** Group analysis of densitometric quantification stratified by sera condition of pp70S6K (Thr 389). **F)** Western Blot of Inhibition of Akt activation by stimulation with sera from women with different BMI. **G)** Group analysis of densitometric quantification of Inhibition of Akt activation by stimulation with sera from women with different BMI. For viability assay, the MCF-7 cells were treated with 10% inactivated fetal bovine serum (Ctr), 5% FBS or 5% sera from normal-weight premenopausal women (NWSPre) or sera from obese premenopausal women (OSPre). T0 corresponds to the viability at the time the different sera were added, and normalized against Ctr. For Western Blot, the cells were stimulated for 10 min with recombinant human insulin (Ins) (0.5 UI/ml) (positive control for activation), 5% NWSPre or 5% OSPre. T0 represents the phosphorylation level before stimulation with Ins or human sera. All experiments were carried out in triplicate (n = 9) for each serum evaluated. Comparison of means ** P <0.005, * P <0.05. ** Ctr or T0, ## 5% FBS or Ins, ++ NWSPre, @@ Without inhibitors.

When evaluating cell viability, we observed that the MCF-7 line under supplementation with sera from obese premenopausal women (OSPre), presented an increase in cell viability of 20% (p = 0.04) compared to the control (Ctr), while compared to the NWSPre sera the increase was 29% (p = 0.05) (Fig 2A).

Subsequently, we determined the optimal stimulation time for the activation of the IR-β/Akt/p70S6K pathway by exposure to human sera through a time course. A mild increase in phosphorylation of pIR-β (Tyr 1162–1163) was induced within the first 5 min of stimulation with OSPre serum (0.38-fold, p = 0.000002) and remained until 60 min (0.27-fold, p = 0.003) compared to T0 (Fig 2B and 2C). For pAkt (Ser 473), stimulation with OSPre serum induced a 1.39-fold (p = 3.7278E-8) increase in phosphorylation with respect to T0 within the first 5 min of stimulation and remained above the signal at T0 up to 60 min (1.22-fold, p = 3.728E-8) (Fig 2B and 2D). Phosphorylation of Akt was higher than that observed under stimulation with Ins. Relevantly, even when phosphorylation of pAkt (Ser 473) reached a higher level in the first 5 min after stimulation with OSPre serum, pp70S6K (Thr 389) phosphorylation increased 0.83-fold (p = 3.7295E-8) at 60 min (Fig 2B and 2E). In conclusion, OSPre sera led to differential activation of the IR-β/Akt/p70S6K pathway, with clear early Akt activation. Differences in the signals induced by insulin result from adjustments of the time of exposure to avoid saturation, both IR-β and Akt reach similar intensities when simultaneously developed.

Next, we examined if only the IR-β/PI3K pathway played a role in Akt phosphorylation by exposure to human sera. We investigated the cellular response to Genistein that interferes with global phosphorylation, Wortmannin that effectively inhibits PI3K and Bortezomib, a proteosome inhibitor. Under stimulation with OSPre, the pre-incubation with Genistein (0.119-fold, p = 0.000714) and Bortezomib (0.224-fold, p = 1.3218E-9) lead to a significant pAkt (Ser 473) decrease compared to OSPre without inhibitors (Fig 2F and 2G). While under NWSPre stimulation, the suppressive effect of Wortmannin (0.3-fold, p = 4.2422E-13) and Bortezomib (0.26-fold, p = 8.2452E-12) on pAkt (Ser 473) was much stronger than the effect of Genistein (Fig 2F and 2G). Showing that Akt activation induced by OSPre sera is mainly explained by a less PI3K-dependent signaling pathway. This suggests that Akt activation is less associated with the presence of insulin/IGF-1 in sera of obese premenopausal women.

Taken together, these results show that stimulation with sera from obese women produces a differential activation mechanism compared to sera from normal-weight women.

### Evaluation of MCF-7 cells in response to human sera with different BMIs and hormonal states

Accumulating evidence has revealed an interaction between obesity and hormonal status in breast cancer patients. For example, postmenopausal women are more likely to develop obesity than premenopausal women, this effect is attributed to estrogen depletion, aging, and lifestyle practices **[39–42]**. Also, the obesity-breast cancer association varies between pre and postmenopausal women **[43]**. To understand how the menopausal state impacts the effects of obesity-associated serum molecules, we analyzed a set of sera collected from pre and postmenopausal women with different BMIs.

The characteristics of the study groups are described in Table 1, where triglycerides presented a statistically significant difference between normal-weight compared to obese women of the same age group.

**Table 1:** Anthropometric and biochemical characteristics of the serum of women with different BMIs and hormonal states.

	Premenopausal		Postmenopausal	
	Normal-Weight	Obese	Normal-Weight	Obese
	n = 10	n = 10	n = 2	n = 10
	(NWSPre)	(OSPre)	(NWSPost)	(OSPost)
**Age**	31±6.5	31.8±4.5	52±4.2	55.6±3.8
**Weight (Kg)**	56±4.2	84.6±13.5**	56.5±6.3	98±12.3**
**Glucose (mg/dL)**	84.6±9.7	86.3±4.1	92.1±8.1	91.2±7.2
**Total Cholesterol (mg/dL)**	167.1±11.6	173.6±39	154±9.15	172.1±30.9
**Cholesterol HDL (mg/dL)**	48.6±8.1	56.6±26.7	55±3.6	43.9±8.9*
**Cholesterol LDL (mg/dL)**	101.6±7.5	98.8±21.7	94±7.2	106.5±31.1
**Triglycerides (mg/dL)**	86.9±14.9	160.3±13.5**	101±5.2	148.9±31.6*

** P <0.005, * P <0.05. ** Normal-weight vs Obese.

**Fig 3:**
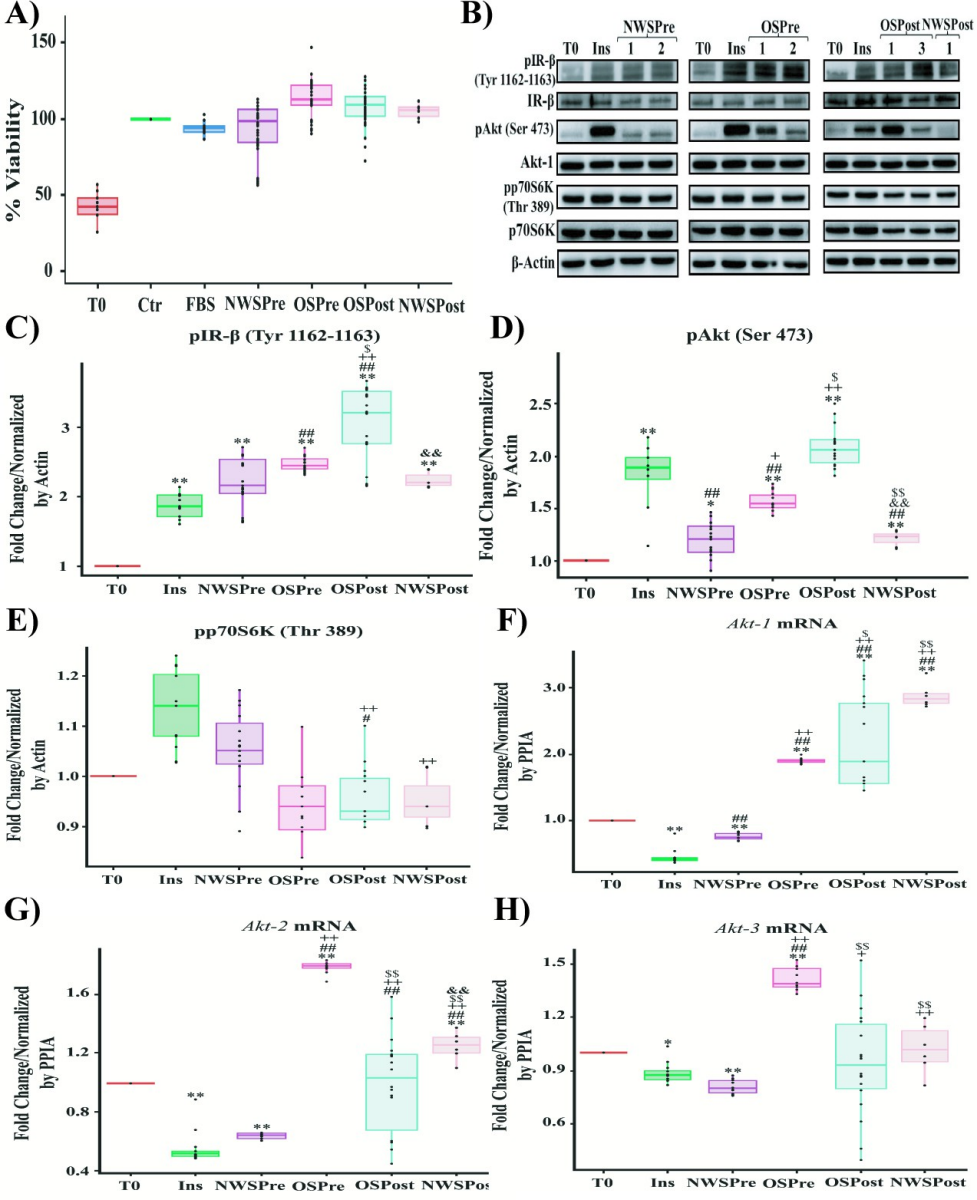
Effect on the viability and activation of the IR-β/Akt/p70S6K pathway of MCF-7 cells by stimulation with human sera from normal-weight and obese women with different hormonal states. **A)** Viability of MCF-7 cells exposed to sera from women with different BMIs and hormonal states. **B)** Western Blot of IR-β/Akt/p70S6K pathway members on MCF-7 cells exposed to sera from women with different BMIs and hormonal states. **C)** Group analysis of densitometric quantification stratified by serum condition of pIR-β (Tyr 1162–1163). **D)** Group analysis of densitometric quantification stratified by serum condition of pAkt (Ser 473). **E)** Group analysis of densitometric quantification stratified by serum condition of pp70S6K (Thr 389). **F)** *Akt-1* expression levels on MCF-7 cells stimulated with human sera. **G)** *Akt-2* expression levels on MCF-7 cells stimulated with human sera. **H)** *Akt-3* expression levels on MCF-7 cells stimulated with human sera. For viability assay, the MCF-7 cells were treated with 10% inactivated fetal bovine serum (Ctr), 5% FBS, sera from normal-weight premenopausal women (NWSPre), sera from obese premenopausal women (OSPre), sera from obese postmenopausal women (OSPost) or sera from normal-weight postmenopausal women (NWSPost). For Western Blot test the cells were stimulated with recombinant human insulin (Ins) (0.5 UI/ml) (positive control for activation of the IR-β/Akt pathway) or 5% NWSPre, OSPre, OSPost or NWSPost. Labels of Western Blot as described in Fig 2. RT-PCR for Akt isoforms: Akt-1, Akt-2, Akt-3, and peptidylprolyl isomerase A (PPIA) as constitutive control. Western blot armed image. All experiments were carried out in triplicate (n = 9) for each serum evaluated. Comparison of means ** P <0.005, * P <0.05. ** Ctr or T0, ## 5% FBS or Ins, ++ NWSPre, $ $ OSPre, && OSPost.

We first evaluated if there was a differential effect on MCF-7 cell viability due to sera stimulation from women with different BMIs and menopausal statuses. Fig 3A shows that OSPre sera induced a significant increment in cell viability compared to Ctr (12.7%, p = 0.00011), 5% FBS (19%, p = 0.000001), and NWSPre (19%, p = 0.00005). Similarly, OSPost (sera from obese postmenopausal women) sera produced higher increment on cell viability when compared to Ctr (7%, p = 0.016), 5% FBS (13.5%, p = 0.000116) and NWSPre (13%, p = 0.005).

Subsequently, we evaluated the variations in the activation of IR-β/Akt/p70S6K under the stimulation with human sera. The Western Blot analysis revealed an increase in pIR-β (Tyr 1162–1163) phosphorylation in response to OSPost stimulation compared to NWSPre (0.87-fold, p = 0.0003), OSPre (0.6-fold, p = .007) and NWSPost (sera from normal-weight postmenopausal women) (0.81-fold, 0.0016) (Fig 3B and 3C). Moreover, OSPost exposition led to a more significant pAkt (Ser 473) phosphorylation than that observed under the stimulation with NWSPre (0.86-fold, p = 0.00002), OSPre (0.5-fold, p = 8.2361E-7) and NWSPost (0.8-fold, p = 0.05) (Fig 3B and 3D). Contrary to what was expected, pp70S6K (Thr 389) showed a lower phosphorylation level on OSPre (0.11-fold, p = 0.003) and OSPost (0.09-fold, p = 0.01) as opposed to NWSPre (Fig 3B and 3E).

Later we evaluated the expression of the three Akt mRNA isoforms under stimulation with sera from women with different BMI and hormonal states, considering that the Akt family members regulate distinct physiological functions **[44–48]**. We observed that NWSPre sera decreased 0.25-fold (p = 0.0031) in *Akt-1* mRNA, while we observed an increase with OSPre (0.9-fold, p = 1.4063E-12), OSPost (1.2-fold, p = 2.2983E-22) and NWSPost (1.9-fold, p = 1.1426E-27) compared to T0 (Figs 3F and S5). *Akt-2* mRNA decreased 0.36-fold (p = 7.3146E-8) by stimulation with NWSPre sera, contrary to the increase under stimulation with OSPre (0.78-fold, p = 2.4309E-8) and NWSPost (0.24-fold, p = 0.0043) respect to T0 (Figs 3G and S5). Finally, *Akt-3* mRNA decreased 0.18-fold (p = 0.0084) due to exposure to NWSPre sera, while only OSPre increased 0.41-fold (p = 2.4338E-8) compared to T0 (Figs 3H and S5).

These data collectively suggest that serum from obese women, mainly from postmenopausal women, might contribute to an increase in the viability levels of tumoral cells, associated with an increase in phosphorylation of IR-β and Akt. These last results and the fact that a higher incidence of breast cancer in obese postmenopausal women has been reported led us to focus on this group to perform a pharmacological intervention with Metformin and determine if the effect on the tumor cell could be reversed.

### Effect of sera from obese postmenopausal women treated with Metformin on MCF-7 cells

Retrospective studies have reported a lower incidence of invasive breast cancer in patients with Metformin treatment **[23–26]**, this could be explained, in part, by changes in circulating molecules contained in the sera from obese women exerted by the pharmacological intervention. Taking advantage of an ongoing study at the Instituto Nacional de Ciencias Médicas y Nutrición Salvador Zubirán (INCMNSZ), we collected 64 sera from obese postmenopausal women with no previous diabetes diagnosis and undergoing Metformin treatment with 2550 mg/day. After ten weeks, 30 patients completed the treatment. We used these sera to determine if the metabolic control generated by the drug was capable of reversing the phenotype observed in the tumor cells, resulting from the exposure to serum from obese women.

The anthropometric and biochemical characteristics are described in Table 2. Our data show that there was a reduction in weight of 5% after treatment with Metformin and only patients with insulin resistance (Homeostatic Model Assessment HOMA>3) presented a reduction of 1.15 in HOMA-IR.

**Table 2:** Anthropometric characteristics, blood chemistry and HOMA of postmenopausal women treated with Metformin for 10 weeks.

	Total Population		HOMA<3		HOMA>3	
	n = 30		n = 21		n = 9	
**Age**	53.4±11.65		52±13.5		56.6±3.7	
	**0 Weeks**	**10 Weeks**	**0 Weeks**	**10 Weeks**	**0 Weeks**	**10 Weeks**
**BMI (Kg/m**^**2**^)	40.2±7.2	39.1±6.7	39.5±7.7	38.9±7.7	42.1±5.8	39.6±4.6
**Glucose (mg/dL)**	92.6±8.4	90.7±10.4	89.7±5.9	91.4±9.5	98.7±9.8	88.5±7.6
**Total Cholesterol (mg/dL)**	185±34.2	183.6±31	187.2±35	183.5±33	179.8±33	184±31.1
**Cholesterol HDL (mg/dL)**	46.8±11.5	49.2±18.3	49±11.5	49±6.97	41.7±10.2	38.5±7.1
**Cholesterol LDL (mg/dL)**	118±30.7	116±31.9	116.7±34	115.2±35	120.8±22	118.5±23
**Triglycerides(mg/dL)**	150.1±60	159.6±67	144.5±57	135.7±51	163±67.7	143.5±47
**HOMA-IR**	2.3±1.45	2.1±1	1.4±0.4	1.9±0.9	4.15±0.9	3±0.6*
**Weight (Kg)**	102.3±17	99.6±13	99.9±19.9	100.4±16	97.3±16.5	92.4±16.2
**% Weight Change**	-5.1±3.7		-4.7±2.5		-5.1±2.2	
**Obesity Grade**	I	n = 12	I	n = 9	I	n = 3
	II	n = 5	II	n = 4	II	n = 1
	III	n = 13	III	n = 8	III	n = 5

** P <0.005, * P <0.05. ** 0 Weeks vs 10 Weeks.

To evaluate the effect of sera from patients under Metformin treatment, we determined cell viability and levels of pAkt (Ser 473) phosphorylation on MCF-7 cells in equivalent experiments to those presented in Fig 3. We used the serum of each patient collected before initiating Metformin treatment (0 Weeks) as their control.

**Fig 4:**
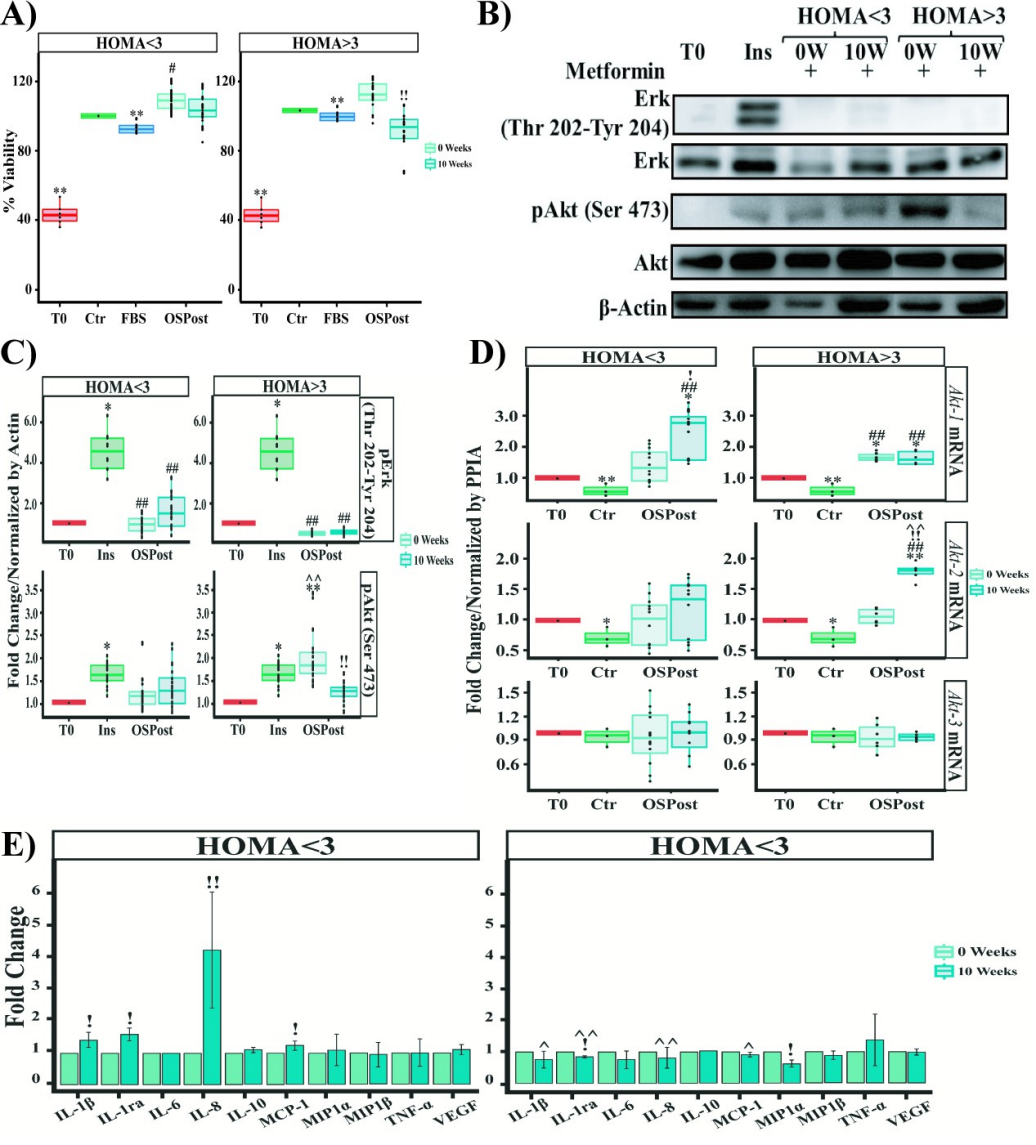
Effect of sera from Metformin-treated postmenopausal obese women on MCF-7 cells. **A)** Viability of MCF-7 cells exposed to OSPost serum without insulin resistance (HOMA<3) and OSPost serum with insulin resistance (HOMA>3). **B)** Western Blot of MCF-7 cells exposed to OSPost serum without insulin resistance (HOMA<3) and OSPost serum with insulin resistance (HOMA>3). **C)** Western Blot’s Densitometry of pErk (Thr 202-Tyr204) and pAkt (Ser 473) of MCF-7 divided by the presence of insulin resistance. **D)** Akt Isoforms expression levels on MCF-7 cells stimulated with OSPost at 0 and 10 weeks of Metformin treatment. **E)** Determination of serum molecule levels in postmenopausal obese women treated at 0 and 10 weeks of treatment with Metformin. Labels of Western Blot as described in Fig 2. Labels of RT-PCR as described in Fig 3. Western blot armed image. The plotted data correspond to three independent experiments (n = 3) for each serum evaluated. Comparison of means ** P <0.005, * P <0.05. ** Ctr or T0, ## 5% FBS or Ins, !! W0, ^^ respect HOMA<3 vs HOMA>3.

The MCF-7 line did not show significant changes in cell viability or pAkt (Ser 473) phosphorylation levels after exposure to sera of patients with ten weeks of treatment with Metformin (S6A and S6B Fig). However, when the population was analyzed by the HOMA score, we observed that the serum of patients with HOMA>3 induced a reduction in viability (21%, p = 5.5E-7) and pAkt (Ser 473) phosphorylation at 10 Weeks compared to 0 Weeks (0.8-folds, p = 3.3E-7) (Fig 4A–4C).

On the other hand, to determine if the viability induced by the sera of women with HOMA<3 was a product of the activation of the MAPK kinase pathway, we evaluated the phosphorylation of pERK (Thr 202-Tyr 204). However, although a slight increase was observed, it was not statistically significant (Fig 4B and 4C lower panel).

We subsequently evaluated the expression of the three *Akt* mRNA isoforms on MCF-7 cells exposed to sera obtained before and after 10 weeks with Metformin treatment. For *Akt-1* mRNA, a 0.7-fold (p = 0.008) increase was observed at ten weeks of treatment with Metformin (10W) compared to 0W (S6C Fig). When dividing our cohort by HOMA, women with HOMA<3 presented a 1-fold (p = 0.01) increase at 10W compared to their 0W (Fig 4D upper left panel). Whereas the expression of *Akt-2* mRNA decreased 0.4-fold (p = 0.03) under stimulation of 10W sera in comparison to 0W (S6C Fig). But the supplementation with sera from women with HOMA>3 induced a 0.75-fold (p = 0.000019) increase at 10W respect their 0W (Fig 4D central right panel). Finally, *Akt-3* mRNA did not present significant changes in the total population or subgrouping it by HOMA (Figs S6C and 4D lower panel).

In obese patients, several studies have shown an increased production of inflammatory cytokines **[49–51]**, so we aimed to measure the cytokines in sera from patients after Metformin treatment. As part of an initial characterization of sera from obese patients treated with Metformin, we performed an ELISA BioPLEX immunoassay (see Materials and methods). Only IL-8 showed a 1.5-fold increase (p = 5.8E-7) at 10W weeks of treatment with Metformin (S6D Fig). When the population was divided according to HOMA score (HOMA>3), it was observed that after 10 weeks of drug treatment, the patients who presented decreased viability and pAkt (Ser 473) phosphorylation showed a reduction in IL-1ra (0.2-fold, p = 3.3e-02) and MIP-1α (0.4-fold, p = 1.1e-01) levels. Contrary to the increase in IL-1β (0.4-fold, p = 0.01), IL-1ra (0.55-fold, p = 0.0081), IL-8 (3.2-fold, p = 1.82e-10) and MCP-1(0.22-fold, p = 0.013) levels in patients who had no change at ten weeks of treatment were observed (Fig 4E).

**Fig 5:**
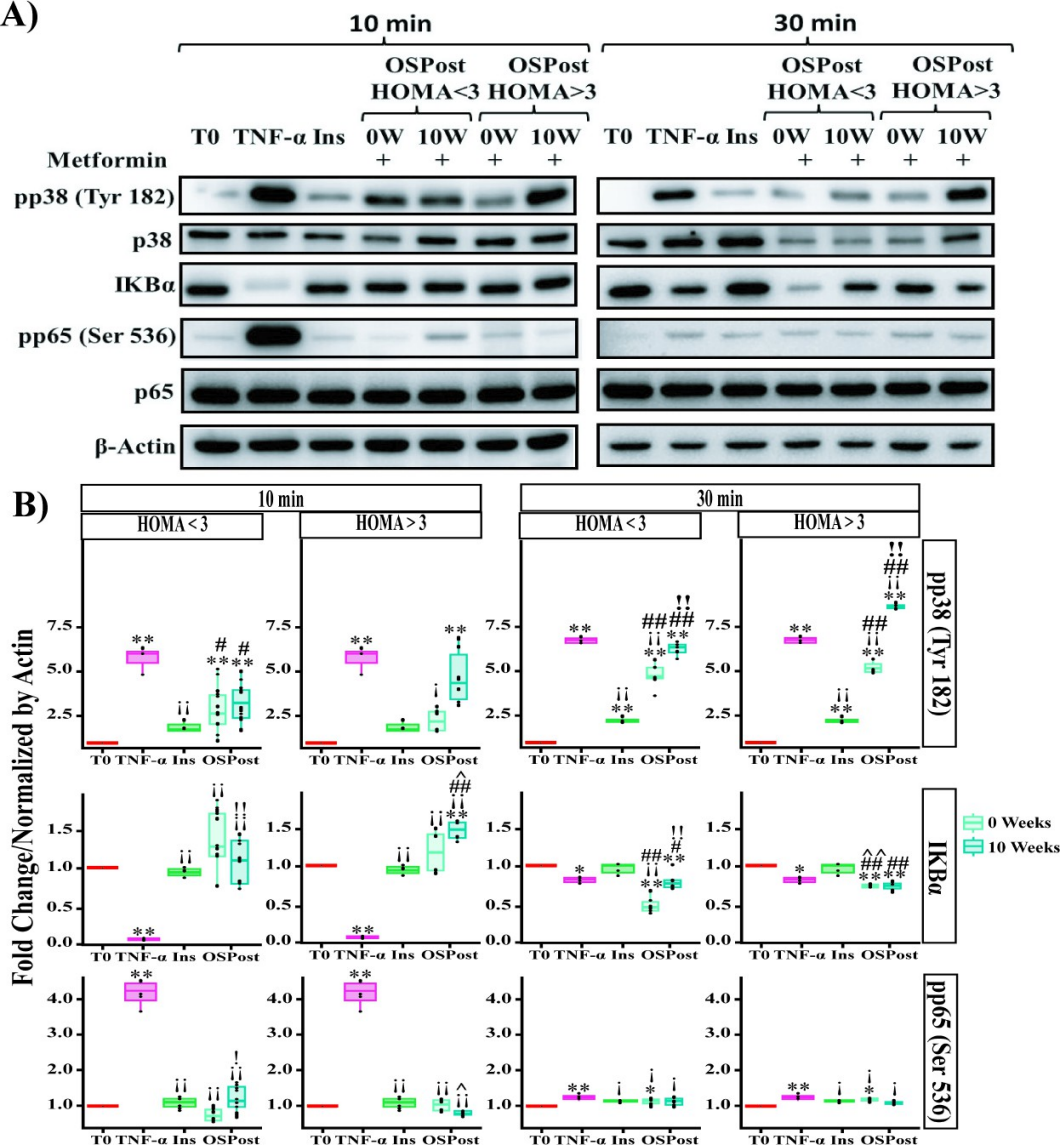
Effect of sera from Metformin-treated postmenopausal obese women on NF-*κ*B signaling in MCF-7 cells. **A)** Western Blot of pp38 (Tyr 182), IKBα and pp65 (Ser 536) under stimulation with OSPost serum without insulin resistance (HOMA<3) and OSPost serum with insulin resistance (HOMA>3) at 10 and 30 min of stimulation. **B)** Group analysis of densitometric quantification of pp38 (Tyr 138), IKBα and pp65 (Ser 536) by stimulation with OSPost serum without insulin resistance (HOMA<3) and OSPost serum with insulin resistance (HOMA>3) at 10 min and 30 min. Labels of Western Blot as described in Fig 2. Western blot armed image. The plotted data correspond to three independent experiments (n = 3) for each serum evaluated. Comparison of means ** P <0.005, * P <0.05). ** T0, ¡¡ TNF-α, ## Ins, !! W0, ^^ respect HOMA<3 vs HOMA>3.

The changes in pro-inflammatory cytokines, after 10 weeks of treatment with Metformin, led us to evaluate NF-κB, which is activated by IL-8 **[52, 53]**. On the other hand, in some cases it has been reported that the activation of NF-κB requires the activation of p38 as a cofactor **[54]**, which has been reported to be regulated by Metformin **[55]**. Contrary to expectations, stimulation with sera from women with HOMA<3 induced a differential regulation in NF-κB (nuclear factor κB) signaling. At 10 min of stimulation, sera from patients with 10 weeks of Metformin treatment (HOMA<3-10W) did not induce changes in pp38 (Tyr 182) phosphorylation (Fig 5A left panel and 5B upper left panel). Although IKBα degradation increased 0.25-fold (p = 0.03) compared to 0W (HOMA<3) (Fig 5A-left panel and 5B-middle left). While pp65 (Ser 536) phosphorylation increased 0.45-fold (p = 0.035) under stimulation with 10 W sera (HOMA<3) compared to 0 W (Fig 5A-left panel and 5B-lower left panel). On the other hand, after 30 min of exposure with 10W sera (HOMA<3) there was an increase of 1.45-fold (p = 4.6E-5) in the phosphorylation of pp38 (Tyr 182) and 0.3-fold (p = 3E-6) in IKBα compared to 0W (HOMA<3) (Fig 5A-right and 5C- upper and middle right panels). But in pp65 (Ser 536) there were no statistically significant changes and the phosphorylation levels were similar to those observed at 10 min of stimulation (Fig 5A-left panel and 5C-right panel). It is worth mentioning that a 3.3-fold increase (p = 2E-8) in pp38 (Tyr 182) phosphorylation was observed at 30 min of stimulation with sera from women with HOMA>3-10W compared to 0W (HOMA>3) (Fig 5A and 5B-upper right panel).

Overall, Metformin pharmacological intervention in obese postmenopausal women with insulin resistance was able to modify the patients’ sera and attenuated the effects observed in our study on cell viability and Akt phosphorylation on MCF-7 cells, related to obesity-associated phenotypes. This finding reinforces the concept that cell viability and Akt activation are relevant indicators of the link between circulating molecules associated to obesity in front of the activation of the oncogenic pathway.

## Discussion

Obesity is associated with an increased risk of mortality regardless of BMI **[56]**. It has been postulated that the secretion of adipocytokines, establishes the connection between obesity and breast cancer **[57]**. Consequently, identifying how these obesity-associated serum molecules drive carcinogenic processes can help to clarify the mechanisms that connect obesity and breast cancer incidence.

Even though it presents high promiscuity to multiple processes and molecules, we decided to focus on Akt and its isoforms due to its relevant role in the relationship between obesity and breast cancer, acting as an integration center for multiple signaling pathways.

Different strategies to assess the relationship between breast cancer and obesity have been used. One of them focuses on the use of sera from patients with breast cancer, but in this approach, a variety of elements may alter the Akt’s expression and activity, such as chemotherapy, hormonal therapy, and target therapy present in the serum of these patients **[58–61]**. The strategy adopted in our study focuses on the effects of serum molecules associated with obesity in individuals free of breast cancer.

The epidemiology in retrospective studies indicated that obesity has a protective effect in premenopausal women, but in recent years an increase in triple-negative breast cancer incidence has been observed in this population **[43, 62, 63]**. On the other hand, obesity is mainly associated with the incidence of luminal subtype of breast cancer in postmenopausal women **[64, 65]**. Increased Akt phosphorylation was reported in both subtypes of breast cancer **[34]**. However, our in-silico analysis did not show an association between obesity and increased expression and phosphorylation of Akt in tumor tissue. The lack of classification in the different subtypes of breast cancer in the two different data sets used may explain the absence of association.

Bowers *et al*. **[5, 36, 37]** have used cultures of breast cancer cells exposed to sera from breast cancer patients with and without obesity. Similarly, we have characterized different phenotypes of MCF-7 cells generated by exposition to sera from obese and normal-weight women without breast cancer. The most significant change that we observed was that the serum of obese women induced earlier and sustained Akt activation associated with cell vitality.

The lack of concordance between increased phosphorylation of IR-β and Akt, and the fact that different inhibitors of the IR-β/Akt pathway do not prevent phosphorylation of Akt, suggests that serum from obese women contains molecules other than insulin or IGF **[66, 67]** that contribute to Akt activation; such as leptin, estrogens, pro-inflammatory cytokines, and triglycerides **[5, 66–69]**.

The lack of normal-human ductal and lobular primary culture prevented us from having an ideal control for non-tumor mammary epithelium. However, normal mammary immortalized epithelium cells (MCF-10A) could be used for that purpose. Although the bioinformatics analysis showed that normal breast tissue presented the highest increase in Akt activation mediated by obesity, MCF-10A cells were discarded, since they did not show adequate activation of the IR-β/Akt/p70S6K pathway by stimulation with human serum (S3A Fig), nor could they differentiate between sera from different BMI (S4A Fig).

In the search for an adequate breast cancer in vitro model, we screened different cell lines from each molecular subtype of breast cancer: luminal (MCF-7, ZR-75-30), Her2+ (SKBR-3, SKBR-3R) and triple-negative (MDA-MB-231, MDA-MB-468). From this panel, MCF7 cells were the most sensitive to human sera of the different BMI groups by presenting greater differences in the percentages of viability, probably associated with the higher concentration of estrogens present in the sera of women with obesity and the sensitivity of MCF-7 cells to this molecule **[5, 11, 40]**. MDA-MB-231 cells are the second most studied breast cancer cellular model. However, although these cells showed the same trend in increasing viability and Akt activation under stimulation with sera from obese women these changes did not reach statistical significance (S4E Fig).

Even with the limited number of samples included in this pilot study, we observed variability between sera from women with the same BMI. Possibly by the existence of obese individuals with a "healthy" metabolic state and normal-weight individuals with a "sick" metabolic state **[70]**. In the case of normal-weight women, the sera that had high levels of triglycerides also presented higher levels of Akt phosphorylation. To validate this hypothesis more patients have to be analyzed.

Diabetes adds complexity and confounding variables to the changes in human serum, for this reason, we excluded patients with this condition. Using a glucose tolerance test, we certified that none of the patients included in our study had diabetes, but 30% were women with a HOMA score higher than three, which was taken as the reference value to determine insulin resistance. After treatment with Metformin and regardless of the degree of obesity, there was a positive effect on the metabolism of the patients with HOMA>3, these sera being the ones that decreased their ability to induce viability in MCF-7 cells through Akt. On the other hand, although it was expected that in patients without insulin resistance (HOMA<3), the increase in proliferation would be mediated through the MAPK pathway, no changes were observed after Metformin treatment in Erk phosphorylation. Therefore, in addition to insulin resistance, other metabolic and physiological alterations could be responsible for the observed effects on MCF-7 cells in these two subgroups of obese women.

Our initial characterization of the sera following Metformin treatment showed insulin and MIP1α reduction in women who presented HOMA>3, which could partly explain Akt phosphorylation reduction **[64, 71]**. However, although a reduction in IL-6, MCP-1, TNF-α, INF-γ, GM-CSF, and IL-17 has been reported after Metformin treatment **[72–74]**, we did not observe significant changes in any of them. Also, we cannot rule out the influence of other molecules that we did not evaluate and that are regulated by drug treatment, such as adiponectin and leptin **[75, 76]**. For MCP-1, TNF-α and VEGF, the reduction in concentration after treatment with Metformin was only observed in women with grade III obesity, suggesting that these changes depend on the degree of obesity (S7 Fig).

In breast cancer, Akt activation induces the activation of multiple pathways, including the NF-κB pathway **[75–77]**. On the other hand, among the antitumor mechanisms of Metformin are the inhibition of Akt and NF-κB signaling **[54, 78–84]**. While in women whose sera did not induce changes in viability and Akt phosphorylation after treatment with Metformin, we observed a reduction in IKBα degradation and an increase in p65 phosphorylation, independently of phosphorylation of p38, which could indicate that treatment with Metformin in patients with HOMA<3 has an anti-apoptotic effect, although we do not rule out that the observed NF-κB activation could have an anti-tumor effect as has been reported in multiple studies **[85–88]**. In addition, an increase in p38 phosphorylation was observed in women with and without insulin resistance after treatment with Metformin, which corroborates what was previously reported on an antitumor effect of Metformin through p38 activation **[89–91]**. These results could indicate that Metformin has two independent effects on the tumor cell: i) The first by regulating Akt in women with insulin resistance, ii) The second to regulate NF-κB independently of Akt in women without insulin resistance.

This study represents the starting point for an in-depth characterization of human sera. Our preliminary results provide novel knowledge about the biological panorama in breast cancer cells established by stimulation with human sera with different metabolic characteristics. Furthermore, our study supports the notion that modulating metabolism through pharmacological interventions affects the molecular serum profile and consequently the breast cancer phenotype.

Certainly, there are limitations associated with the small number of sera evaluated for each group. However, the cellular phenotype that we observed was robustly maintained in the different groups of BMI and hormonal states. Another limitation was the use of a single breast cancer line, which prevents us from examining other possible cellular models. In general, our findings add new data to the growing body of evidence showing that serum’s molecular profile of obese women may be pro-tumorigenic through its signaling activity. Besides, it warrants more studies to understand the mechanism by which some Metformin users have a "protective" effect against the development of breast cancer.

## Conclusions

**Fig 6:**
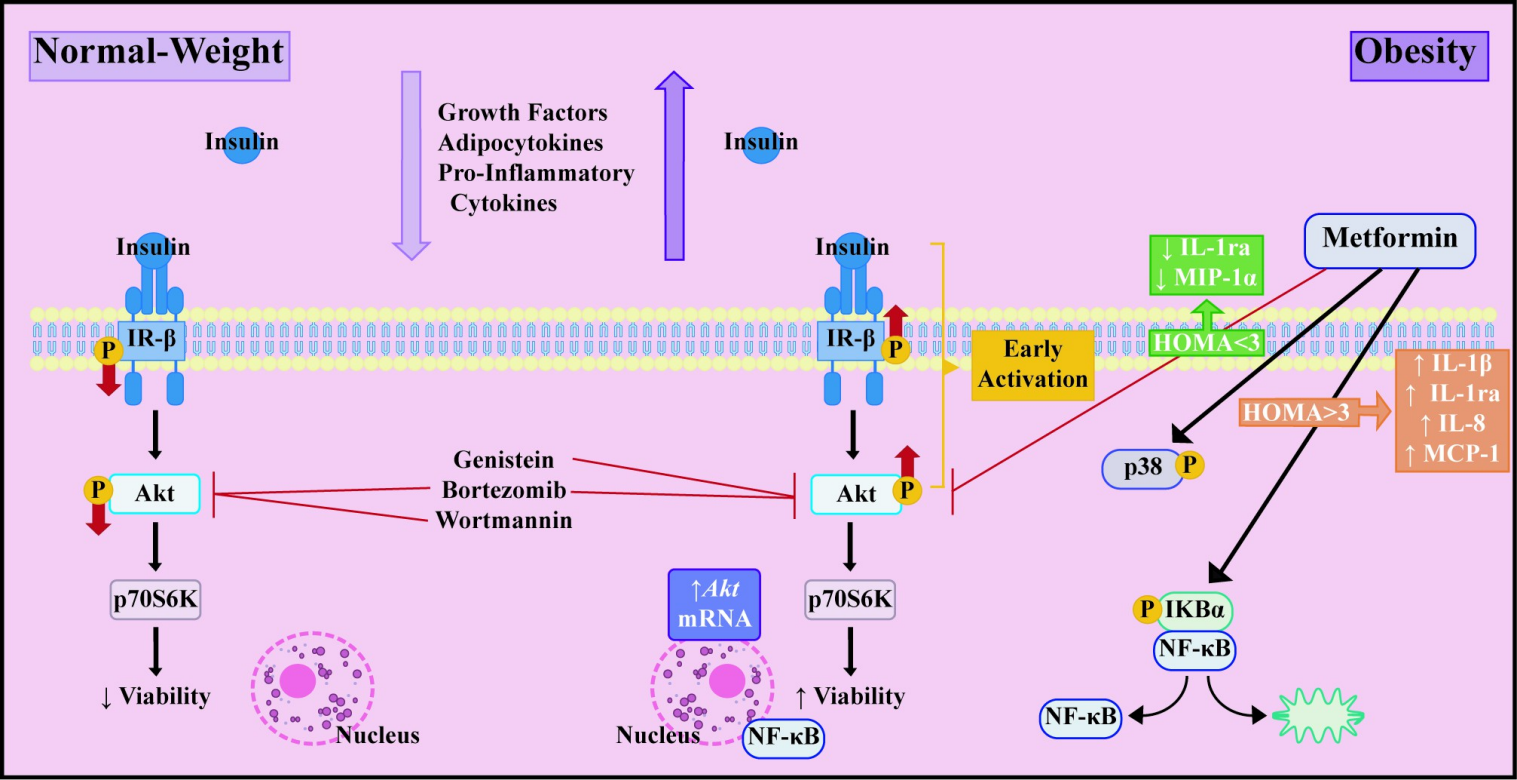
Schematic representation of the activation of the IR-β/Akt/p70S6K pathway in MCF-7 cells exposed to human sera with different metabolic characteristics and effect of Metformin treatment in obese patients.

Our study showed that **in vitro** cultured tumor cells can be used as a cellular model to identify changes in serum molecules associated with different metabolic states. These alterations in serum molecules associated with obesity, both in pre and postmenopausal women, induced an increase in Akt phosphorylation, promoting an increase in the viability of luminal A subtype breast cancer cells. Such effect was reversed with brief Metformin treatment in postmenopausal women with insulin resistance (Fig 6).

## Supporting information

S1 Table Gene transcription signatures associated with AKT pathway activations computed in the TCGA and GEO public available gene expression profiles of breast normal and tumoral tissue. Related to Additional file 2 and Fig 1.
(XLSX)

S1 Fig Comparison of TCGA vs gene expression data set and reverse phase protein matrix data in mammary tumors of premenopausal women. **A)** PI3K/Akt and mTOR signatures of public RPPA and summary of activity scores. **B)** PI3K/Akt and mTOR signatures correlation of public RPPA. **C)** Correlation of transcriptional signature (mRNA) associated with PI3K/AKT phosphorylation state. **D)** Comparison of mRNA and Protein expression pattern in samples with high vs low levels of AktSer473 and AktThr308.
(PDF)

S2 Fig Viability rate of breast cancer cells seeded in sera human sera. **A)** Viability rate of breast cancer cells seeded in sera with or without heat-inactivation. **B)** Tolerance of breast cancer cell lines to human serum without heat-inactivation. All experiments were performed in triplicate (n = 9). The data shows an average +/- SD (** P <0.05 to Ctr).
(PDF)

S3 Fig Activation of IR/Akt/p70S6K pathway by stimulation with normal weight serum premenopausal in breast cancer cell lines. Cells were stimulated for 10 min with Human Recombinant Insulin (Ins) (0.5 U/ml) (positive control of activation of IR/Akt/p70S6K pathway) or 5% Normal Weight Serum Premenopausal (NWSPre). Western Blot against elements of IR/Akt/p70S6K pathway: Insulin beta receptor (IR-b), Phosphorylated beta insulin receptor in tyrosine residues 1162–1163 (pIR-β Tyr 1162–1163), Total Akt-1 (Akt-1), phosphorylated pan-Akt in Serine 473 residue (pAkt Ser 473), beta actin (β-actin) as a constitutive control. **A)** Representative Western blot of elements of IR/Akt pathway of MCF-10A cells. **B)** Representative Western blot of elements of IR/Akt pathway of MCF-7 cells. **C)** Representative Western blot of elements of IR/Akt pathway of ZR-75-30 cells. **D)** Representative Western blot of elements of IR/Akt pathway of BT-474 cells. **E)** Representative Western blot of elements of IR/Akt pathway of BT474R cells. **F)** Representative Western blot of elements of IR/Akt pathway of SKBR-3. **G)** Representative Western blot of elements of IR/Akt pathway of SKBR-3R. **H)** Representative Western blot of elements of IR/Akt pathway of MDA-MB-231. **I)** Representative Western blot of elements of IR/Akt pathway of MDA-MB-468.
(PDF)

S4 Fig Effect of human sera with different metabolic characteristics on viability in breast cancer cells. Breast cancer cells were treated for 48 hours with heat-inactivated fetal bovine serum supplemented with 10% (Ctr), 5% FBS or 5% sera from normal-weight premenopausal women (NWSPre) or sera from obese premenopausal women (OSPre). Boxplot shows the group analysis of cell viability effect on **A)** MCF-7, **B)** MCF-10A, **C)** ZR75-30, **D)** SKBR-3 and **E)** MDA-MB-231 lines seeded with NWSPre or OSPre. T0 corresponds to viability at time the different sera were added. Viability was determined by violet crystal technique and normalizing against Ctr. All experiments were performed by triplicate (n = 9) for each serum evaluated. ** P <0.05.
(PDF)

S5 Fig Expression levels of Akt Isoforms in MCF-7 cells by stimulation of with human sera. MCF-7 cells stimulated for 10 minutes with recombinant human insulin (Ins) (0.5 U/ml) (positive control of activation of IR/Akt pathway) and 5% NWSPre or OSPre or OSPost or NWSPost after 10 min. RT-PCR for Akt isoforms: Akt isoform 1 (Akt-1), Akt isoform 2 (Akt-2), Akt isoform 3 (Akt-3) and Peptidylprolyl isomerase A (PPIA) as constitutive control.
(PDF)

S6 Fig Effect of serum from postmenopausal obese women with Metformin treated on MCF-7 cells. The viability and phosphorylation levels of MCF-7 cells exposed to OSPost sera with Metformin treatment at 0 and 10 weeks were evaluated. **A)** Viability of MCF-7 cells. **B)** Western Blot Densitometry of pAkt (Ser473) of MCF-7 cells. **C)** Akt Isoforms expression levels on MCF-7 cells stimulated with OSPost at 0 and 10 weeks of Metformin treatment. **D)** Determination of serum molecule levels in postmenopausal obese women treated at 0 and 10 weeks of treatment with Metformin. The plotted data correspond to three independent experiments (n = 3) for each serum evaluated. ** P<0.005.
(PDF)

S7 Fig Effect of the grade of obesity on the expression of serum molecules after treatment with Metformin. **A)** MCP-1 levels at T0 and T10 weeks of Metformin treatment in women with grade I obesity. **B)** MCP-1 levels at T0 and T10 weeks of Metformin treatment in women with grade III obesity. **C)** MIP-1beta levels at T0 and T10 weeks of Metformin treatment in women with grade I obesity. **D)** MIP-1beta levels at T0 and T10 weeks of Metformin treatment in women with grade III obesity. **E)** TNFalpha levels at T0 and T10 weeks of treatment with Metformin in women with grade I obesity. **F)** TNFalpha levels at T0 and T10 weeks of treatment with Metformin in women with grade III obesity. **G)** VEGF levels at T0 and T10 weeks of Metformin treatment in women with grade I obesity. **H)** VEGF levels at T0 and T10 weeks of Metformin treatment in women with grade III obesity.
(PDF)

S8 Fig Sample size calculation by G power program. (PDF)

S1 Raw images Western blots: Representative western blot images used for the creation of figures. (PDF)

## Abbreviations

NWSPre: Normal-weight premenopausal women/woman
NWSPost: Normal-weight postmenopausal woman
OSPre: Obese premenopausal women
OSPost: Obese postmenopausal women
Ins: Insulin
Metf: Metformin
FBS: Fetal bovine serum
BMI: Body mass index
AKT: Akt Gene
Akt: Akt mRNA
Akt: Akt protein
