# Supplementary material for: Sera from women with different metabolic and menopause states differentially regulate cell viability and Akt activation in a breast cancer in-vitro model
Source: PLoS One. 2022 Apr 12;17(4):e0266073. doi: 10.1371/journal.pone.0266073 (PMC9004774; doi:10.1371/journal.pone.0266073)
Supplement: S1 Fig — A) PI3K/Akt and mTOR signatures of public RPPA and summary of activity scores. B) PI3K/Akt and mTOR signatures correlation of public RPPA. C) Correlation of transcriptional signature (mRNA) associated with PI3K/AKT phosphorylation state. D) Comparison of mRNA and Protein expression pattern in samples with high vs low levels of AktSer473 and AktThr308. (PDF) [file pone.0266073.s002.pdf]

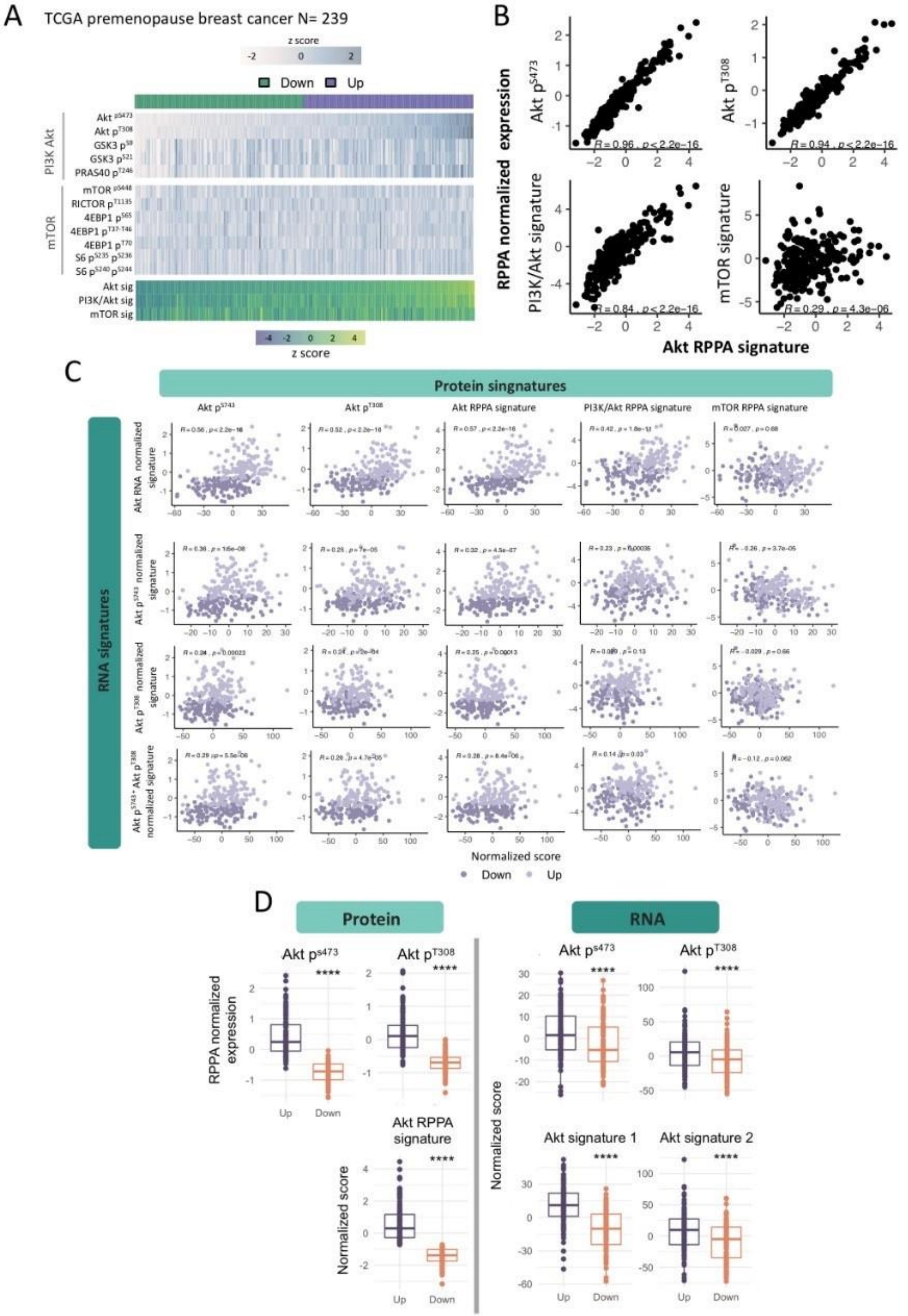
A
TCGA premenopause breast cancer N= 239
z score
Down
Up
PI3K Akt
mTOR
Akt sig
PI3K/Akt sig
mTOR sig
B
RPPA normalized expression
Akt RPPA signature
R = 0.96 , p < 2.2e−16
R = 0.94 , p < 2.2e−16
R = 0.84 , p < 2.2e−16
R = 0.29 , p = 4.3e−06
PI3K/Akt signature
mTOR signature
C
Protein signatures
RNA signatures
Akt RPPA signature
PI3K/Akt RPPA signature
mTOR RPPA signature
Akt RNA normalized signature
Normalized score
Down
Up
D
Protein
RNA
RPPA normalized expression
Akt RPPA signature
Normalized score
Akt signature 1
Akt signature 2
Up
Down
****

Supplementary Figure 1. Comparison of TCGA vs gene expression data set and reverse phase protein matrix data in mammary tumors of premenopausal women. A) PI3K/Akt and mTOR signatures of public RPPA and summary of activity scores. **B)** PI3K/Akt and mTOR signatures correlation of public RPPA. **C)** Correlation of transcriptional signature (mRNA) associated with PI3K/AKT phosphorylation state. **D)** Comparison of mRNA and Protein expression pattern in samples with high vs low levels of AktSer473 and AktThr308
