# Supplementary material for: Sera from women with different metabolic and menopause states differentially regulate cell viability and Akt activation in a breast cancer in-vitro model
Source: PLoS One. 2022 Apr 12;17(4):e0266073. doi: 10.1371/journal.pone.0266073 (PMC9004774; doi:10.1371/journal.pone.0266073)
Supplement: S2 Fig — A) Viability rate of breast cancer cells seeded in sera with or without heat-inactivation. B) Tolerance of breast cancer cell lines to human serum without heat-inactivation. All experiments were performed in triplicate (n = 9). The data shows an average +/- SD (** P <0.05 to Ctr). (PDF) [file pone.0266073.s003.pdf]

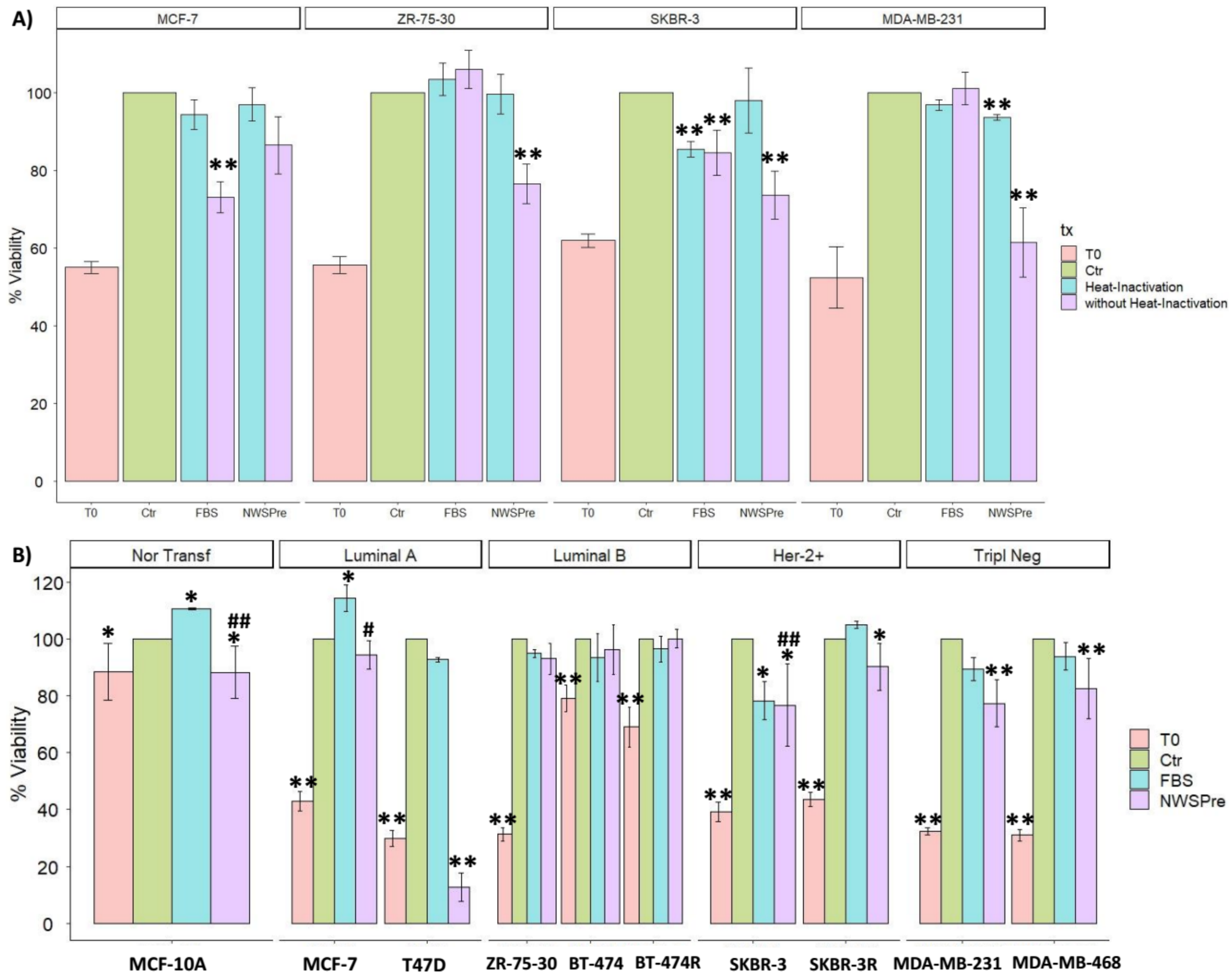

Supplementary Figure 2. Viability rate of breast cancer cells seeded in sera human sera. A) Viability rate of breast cancer cells seeded in sera with or without heat-inactivation. **B)** Tolerance of breast cancer cell lines to human serum without heat-inactivation. All experiments were performed in triplicate (n=9). The data shows an average +/- SD (** P <0.05 to Ctr).
