# Supplementary material for: Sera from women with different metabolic and menopause states differentially regulate cell viability and Akt activation in a breast cancer in-vitro model
Source: PLoS One. 2022 Apr 12;17(4):e0266073. doi: 10.1371/journal.pone.0266073 (PMC9004774; doi:10.1371/journal.pone.0266073)
Supplement: S3 Fig — Cells were stimulated for 10 min with Human Recombinant Insulin (Ins) (0.5 U/ml) (positive control of activation of IR/Akt/p70S6K pathway) or 5% Normal Weight Serum Premenopausal (NWSPre). Western Blot against elements of IR/Akt/p70S6K pathway: Insulin beta receptor (IR-b), Phosphorylated beta insulin receptor in tyrosine residues 1162–1163 (pIR-β Tyr 1162–1163), Total Akt-1 (Akt-1), phosphorylated pan-Akt in Serine 473 residue (pAkt Ser 473), beta actin (β-actin) as a constitutive control. A) Representative Western blot of elements of IR/Akt pathway of MCF-10A cells. B) Representative Western blot of elements of IR/Akt pathway of MCF-7 cells. C) Representative Western blot of elements of IR/Akt pathway of ZR-75-30 cells. D) Representative Western blot of elements of IR/Akt pathway of BT-474 cells. E) Representative Western blot of elements of IR/Akt pathway of BT474R cells. F) Representative Western blot of elements of IR/Akt pathway of SKBR-3. G) Representative Western blot of elements of IR/Akt pathway of SKBR-3R. H) Representative Western blot of elements of IR/Akt pathway of MDA-MB-231. I) Representative Western blot of elements of IR/Akt pathway of MDA-MB-468. (PDF) [file pone.0266073.s004.pdf]

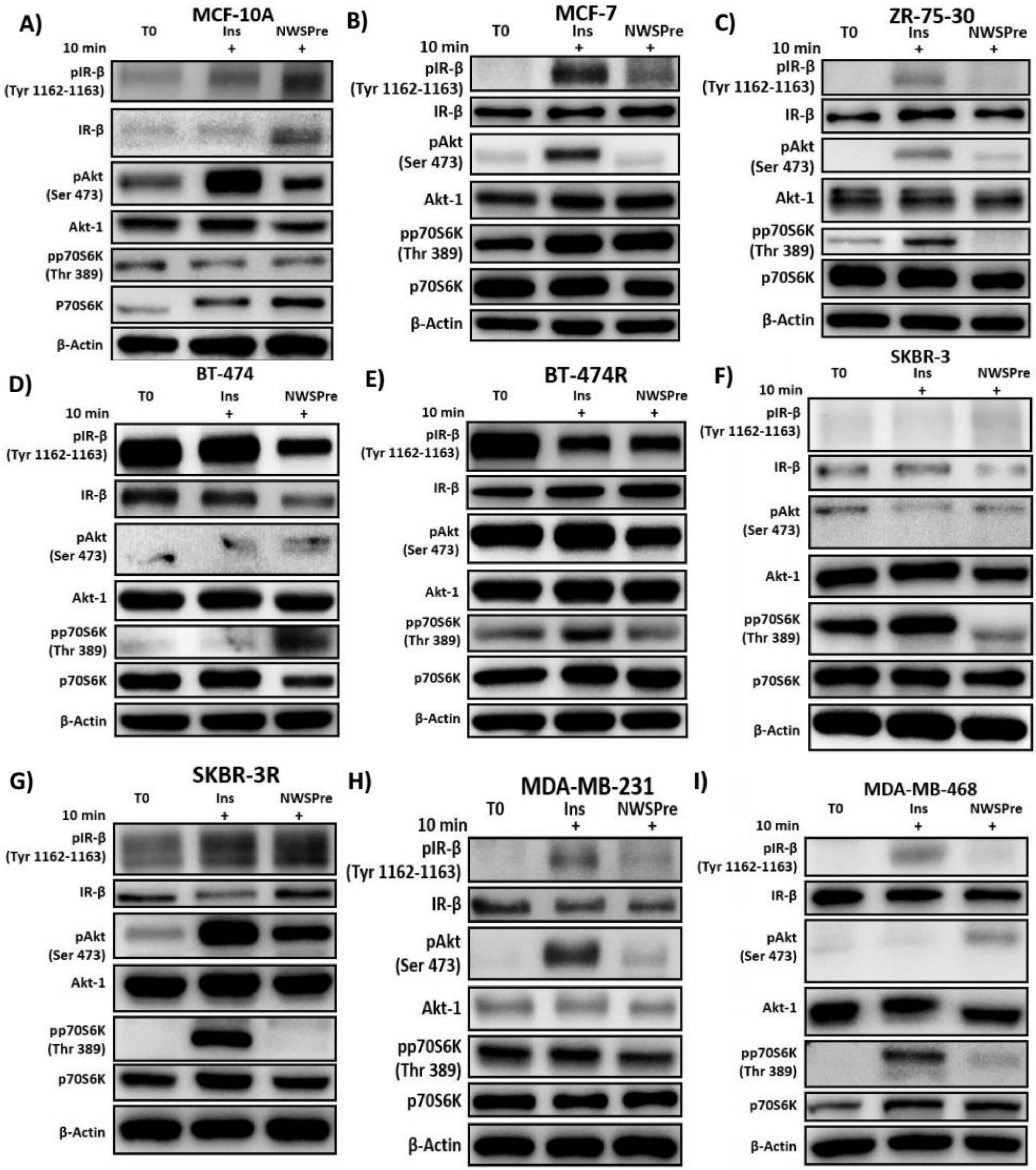
A)
MCF-10A
T0
Ins
NWSPre
10 min
+
+
pIR-β
(Tyr 1162-1163)
IR-β
pAkt
(Ser 473)
Akt-1
pp70S6K
(Thr 389)
P70S6K
β-Actin
B)
MCF-7
T0
Ins
NWSPre
10 min
+
+
pIR-β
(Tyr 1162-1163)
IR-β
pAkt
(Ser 473)
Akt-1
pp70S6K
(Thr 389)
p70S6K
β-Actin
C)
ZR-75-30
T0
Ins
NWSPre
10 min
+
+
pIR-β
(Tyr 1162-1163)
IR-β
pAkt
(Ser 473)
Akt-1
pp70S6K
(Thr 389)
p70S6K
β-Actin
D)
BT-474
T0
Ins
NWSPre
10 min
+
+
pIR-β
(Tyr 1162-1163)
IR-β
pAkt
(Ser 473)
Akt-1
pp70S6K
(Thr 389)
p70S6K
β-Actin
E)
BT-474R
T0
Ins
NWSPre
10 min
+
+
pIR-β
(Tyr 1162-1163)
IR-β
pAkt
(Ser 473)
Akt-1
pp70S6K
(Thr 389)
p70S6K
β-Actin
F)
SKBR-3
T0
Ins
NWSPre
10 min
+
+
pIR-β
(Tyr 1162-1163)
IR-β
pAkt
(Ser 473)
Akt-1
pp70S6K
(Thr 389)
p70S6K
β-Actin
G)
SKBR-3R
T0
Ins
NWSPre
10 min
+
+
pIR-β
(Tyr 1162-1163)
IR-β
pAkt
(Ser 473)
Akt-1
pp70S6K
(Thr 389)
p70S6K
β-Actin
H)
MDA-MB-231
T0
Ins
NWSPre
10 min
+
+
pIR-β
(Tyr 1162-1163)
IR-β
pAkt
(Ser 473)
Akt-1
pp70S6K
(Thr 389)
p70S6K
β-Actin
I)
MDA-MB-468
T0
Ins
NWSPre
10 min
+
+
pIR-β
(Tyr 1162-1163)
IR-β
pAkt
(Ser 473)
Akt-1
pp70S6K
(Thr 389)
p70S6K
β-Actin

Supplementary Figure 3. Activation of IR/Akt/p70S6K pathway by stimulation with Normal Weight Serum Premenopausal in breast cancer cell lines. Cells were stimulated for 10 min with Human Recombinant Insulin (Ins) (0.5 U/ml) (positive control of activation of IR/Akt/p70S6K pathway) or 5 % Normal Weight Serum Premenopausal (NWSPre). Western Blot against elements of IR/Akt/p70S6K pathway: Insulin beta receptor (IR-b), Phosphorylated beta insulin receptor in tyrosine residues 1162-1163 (pIR-β Tyr 1162-1163), Total Akt-1 (Akt-1) , phosphorylated pan-Akt in Serine 473 residue (pAkt Ser 473), beta actin (β-actin) as a constitutive control. **A)** Representative Western blot of elements of IR/Akt pathway of MCF-10A cells. **B)** Representative Western blot of elements of IR/Akt pathway of MCF-7 cells. **C)** Representative Western blot of elements of IR/Akt pathway of ZR-75-30 cells. **D)** Representative Western blot of elements of IR/Akt pathway of BT-474 cells. **E)** Representative Western blot of elements of IR/Akt pathway of BT474R cells. **F)** Representative Western blot of elements of IR/Akt pathway of SKBR-3. **G)** Representative Western blot of elements of IR/Akt pathway of SKBR-3R. **H)** Representative Western blot of elements of IR/Akt pathway of MDA-MB-231. **I)** Representative Western blot of elements of IR/Akt pathway of MDA-MB-468
