# Supplementary material for: Sera from women with different metabolic and menopause states differentially regulate cell viability and Akt activation in a breast cancer in-vitro model
Source: PLoS One. 2022 Apr 12;17(4):e0266073. doi: 10.1371/journal.pone.0266073 (PMC9004774; doi:10.1371/journal.pone.0266073)
Supplement: S4 Fig — Breast cancer cells were treated for 48 hours with heat-inactivated fetal bovine serum supplemented with 10% (Ctr), 5% FBS or 5% sera from normal-weight premenopausal women (NWSPre) or sera from obese premenopausal women (OSPre). Boxplot shows the group analysis of cell viability effect on A) MCF-7, B) MCF-10A, C) ZR75-30, D) SKBR-3 and E) MDA-MB-231 lines seeded with NWSPre or OSPre. T0 corresponds to viability at time the different sera were added. Viability was determined by violet crystal technique and normalizing against Ctr. All experiments were performed by triplicate (n = 9) for each serum evaluated. ** P <0.05. (PDF) [file pone.0266073.s005.pdf]

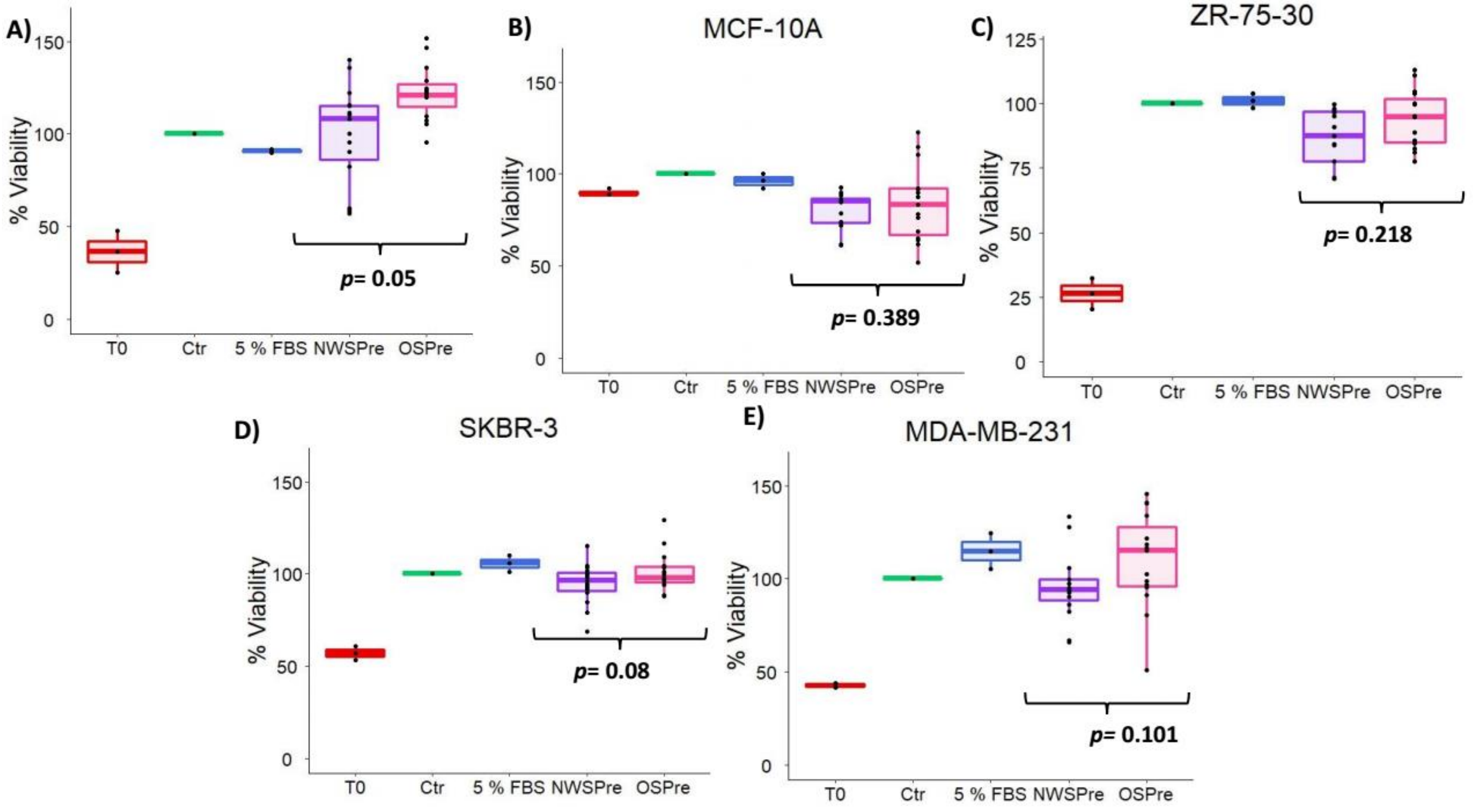

Supplementary Figure 4. Effect of human sera with different metabolic characteristics on viability in breast cancer cells. Breast cancer cells were treated for 48 hours with heatinactivated fetal bovine serum supplemented with 10% (Ctr), 5% FBS or 5% sera from normal-weight premenopausal women (NWSPre) or sera from obese premenopausal women (OSPre). Boxplot shows the group analysis of cell viability effect on **A)** MCF-7, **B)** MCF-10A, **C)** ZR75-30, **D)** SKBR-3 and **E)** MDA-MB-231 lines seeded with NWSPre or OSPre. T0 corresponds to viability at time the different sera were added. Viability was determined by violet crystal technique and normalizing against Ctr. All experiments were performed by triplicate (n = 9) for each serum evaluated. ** P <0.05.
