# Supplementary material for: Sera from women with different metabolic and menopause states differentially regulate cell viability and Akt activation in a breast cancer in-vitro model
Source: PLoS One. 2022 Apr 12;17(4):e0266073. doi: 10.1371/journal.pone.0266073 (PMC9004774; doi:10.1371/journal.pone.0266073)
Supplement: S5 Fig — MCF-7 cells stimulated for 10 minutes with recombinant human insulin (Ins) (0.5 U/ml) (positive control of activation of IR/Akt pathway) and 5% NWSPre or OSPre or OSPost or NWSPost after 10 min. RT-PCR for Akt isoforms: Akt isoform 1 (Akt-1), Akt isoform 2 (Akt-2), Akt isoform 3 (Akt-3) and Peptidylprolyl isomerase A (PPIA) as constitutive control. (PDF) [file pone.0266073.s006.pdf]

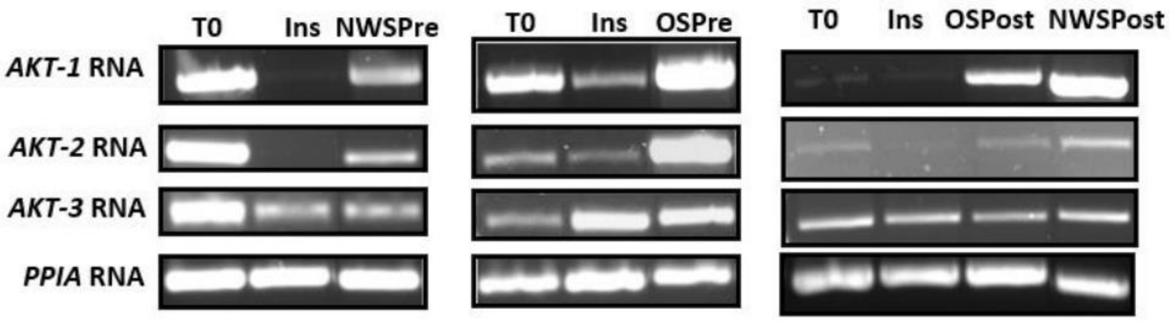


Supplementary Figure 5. Expression levels of Akt Isoforms in MCF-7 cells by stimulation of with human sera. MCF-7 cells stimulated for 10 minutes with recombinant human insulin (Ins) (0.5 U/ml) (positive control of activation of IR/Akt pathway) and 5 % NWSPre or OSPre or OSPost or NWSPost after 10 min. RT-PCR for Akt isoforms: Akt isoform 1 (Akt-1), Akt isoform 2 (Akt-2), Akt isoform 3 (Akt-3) and Peptidylprolyl isomerase A (PPIA) as constitutive control.
