# Supplementary material for: Sera from women with different metabolic and menopause states differentially regulate cell viability and Akt activation in a breast cancer in-vitro model
Source: PLoS One. 2022 Apr 12;17(4):e0266073. doi: 10.1371/journal.pone.0266073 (PMC9004774; doi:10.1371/journal.pone.0266073)
Supplement: S6 Fig — The viability and phosphorylation levels of MCF-7 cells exposed to OSPost sera with Metformin treatment at 0 and 10 weeks were evaluated. A) Viability of MCF-7 cells. B) Western Blot Densitometry of pAkt (Ser473) of MCF-7 cells. C) Akt Isoforms expression levels on MCF-7 cells stimulated with OSPost at 0 and 10 weeks of Metformin treatment. D) Determination of serum molecule levels in postmenopausal obese women treated at 0 and 10 weeks of treatment with Metformin. The plotted data correspond to three independent experiments (n = 3) for each serum evaluated. ** P<0.005. (PDF) [file pone.0266073.s007.pdf]

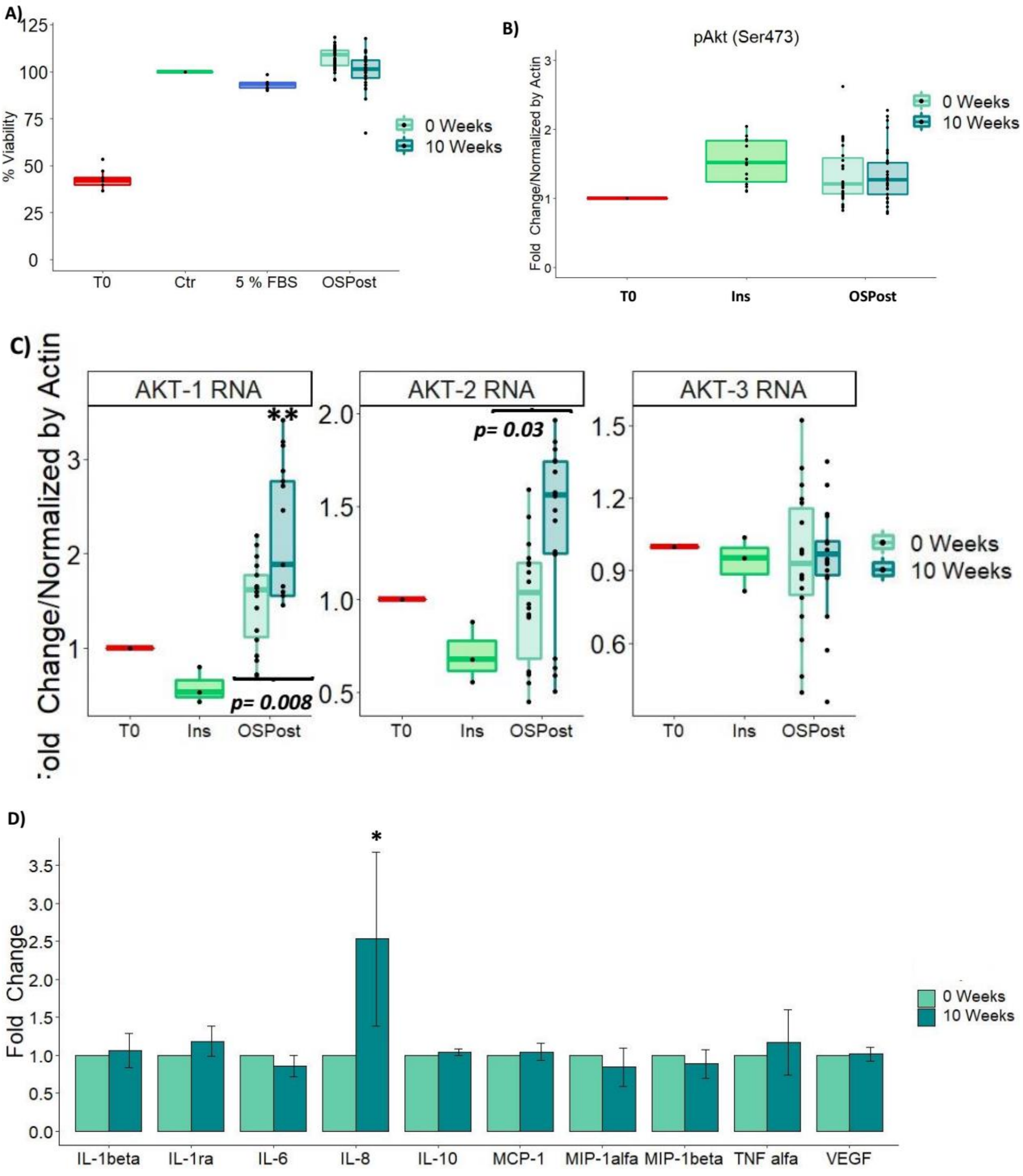

A)
% Viability
125
100
75
50
25
0
T0
Ctr
5 % FBS
OSPost
0 Weeks
10 Weeks
B)
pAkt (Ser473)
Fold Change/Normalized by Actin
3
2
1
0
T0
Ins
OSPost
0 Weeks
10 Weeks
C)
Fold Change/Normalized by Actin
AKT-1 RNA
**
p= 0.008
3
2
1
T0
Ins
OSPost
AKT-2 RNA
p= 0.03
2.0
1.5
1.0
0.5
T0
Ins
OSPost
AKT-3 RNA
1.5
1.2
0.9
0.6
T0
Ins
OSPost
0 Weeks
10 Weeks
D)
Fold Change
*
3.5
3.0
2.5
2.0
1.5
1.0
0.5
0.0
IL-1beta
IL-1ra
IL-6
IL-8
IL-10
MCP-1
MIP-1alfa
MIP-1beta
TNF alfa
VEGF
0 Weeks
10 Weeks

Supplementary Figure 6. Effect of serum from postmenopausal obese women with Metformin treated on MCF-7 cells. The viability and phosphorylation levels of MCF-7 cells exposed to OSPost sera with metformin treatment at 0 and 10 weeks were evaluated. A) Viability of MCF-7 cells. B) Western Blot Densitometry of pAkt (Ser473) of MCF-7 cells. C) Akt Isoforms expression levels in MCF-7 cells stimulated with OSPost at 0 and 10 weeks of Metformin treatment. D) Determination of serum molecule levels in postmenopausal obese women treated at 0 and 10 weeks of treatment with metformin. The plotted data correspond to three independent experiments (n = 3) for each serum evaluated. ** P<0.005.
