# Supplementary material for: Sera from women with different metabolic and menopause states differentially regulate cell viability and Akt activation in a breast cancer in-vitro model
Source: PLoS One. 2022 Apr 12;17(4):e0266073. doi: 10.1371/journal.pone.0266073 (PMC9004774; doi:10.1371/journal.pone.0266073)
Supplement: S7 Fig — A) MCP-1 levels at T0 and T10 weeks of Metformin treatment in women with grade I obesity. B) MCP-1 levels at T0 and T10 weeks of Metformin treatment in women with grade III obesity. C) MIP-1beta levels at T0 and T10 weeks of Metformin treatment in women with grade I obesity. D) MIP-1beta levels at T0 and T10 weeks of Metformin treatment in women with grade III obesity. E) TNFalpha levels at T0 and T10 weeks of treatment with Metformin in women with grade I obesity. F) TNFalpha levels at T0 and T10 weeks of treatment with Metformin in women with grade III obesity. G) VEGF levels at T0 and T10 weeks of Metformin treatment in women with grade I obesity. H) VEGF levels at T0 and T10 weeks of Metformin treatment in women with grade III obesity. (PDF) [file pone.0266073.s008.pdf]

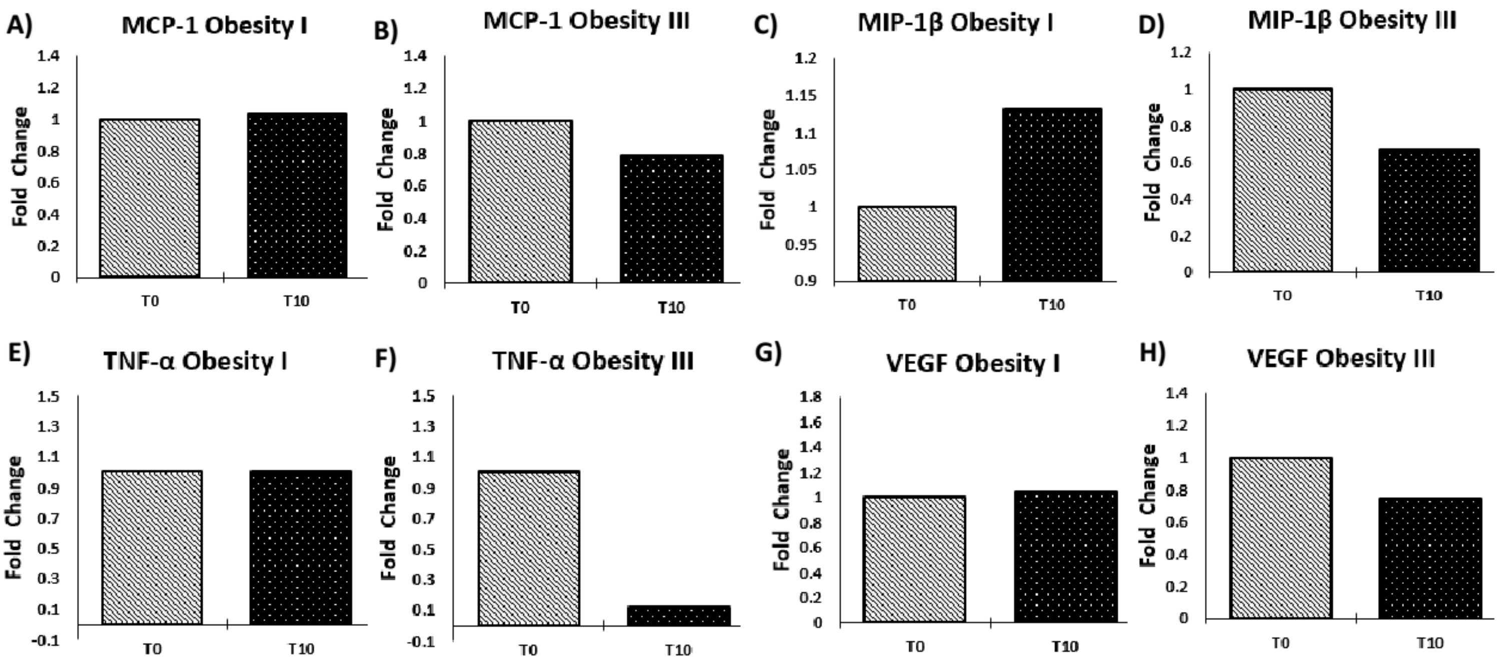

Supplementary Figure 7. Effect of the grade of obesity on the expression of serum molecules after treatment with metformin. A) MCP-1 levels at T0 and T10 weeks of metformin treatment in women with grade I obesity. **B)** A) MCP-1 levels at T0 and T10 weeks of metformin treatment in women with grade III obesity. **C)** MIP-1beta levels at T0 and T10 weeks of metformin treatment in women with grade I obesity. **D)** MIP-1beta levels at T0 and T10 weeks of metformin treatment in women with grade III obesity. **E)** TNFalpha levels at T0 and T10 weeks of treatment with metformin in women with grade I obesity. **F)** TNFalpha levels at T0 and T10 weeks of treatment with metformin in women with grade III obesity. **G)** VEGF levels at T0 and T10 weeks of metformin treatment in women with grade I obesity. **H)** VEGF levels at T0 and T10 weeks of metformin treatment in women with grade III obesity.
