# Supplementary material for: Sera from women with different metabolic and menopause states differentially regulate cell viability and Akt activation in a breast cancer in-vitro model
Source: PLoS One. 2022 Apr 12;17(4):e0266073. doi: 10.1371/journal.pone.0266073 (PMC9004774; doi:10.1371/journal.pone.0266073)
Supplement: S8 Fig — (PDF) [file pone.0266073.s009.pdf]

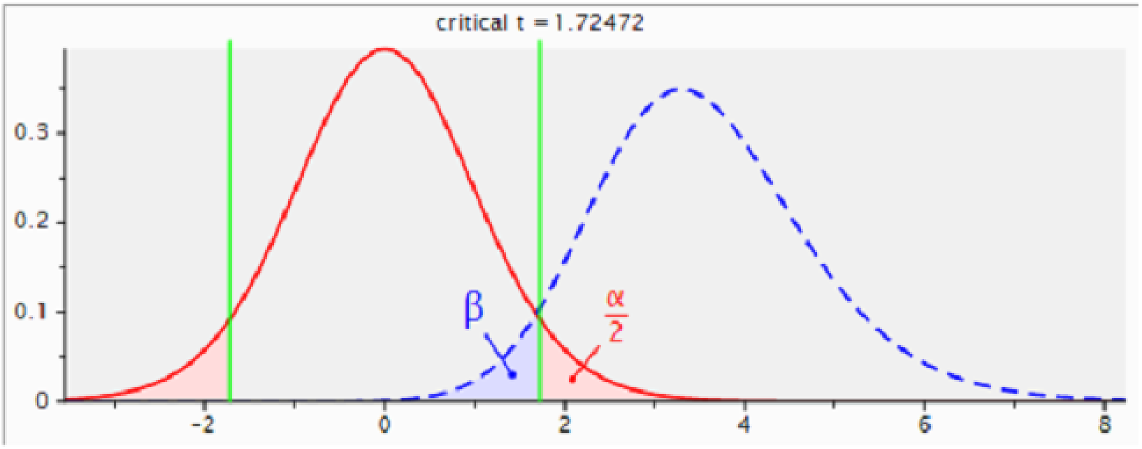

t tests – Means: Difference between two independent means (two groups)

Analysis: A priori: Compute required sample size

Input: Tail(s) = Two
Effect size d = 1.4539158
α err prob = 0.10
Power (1−β err prob) = 0.95
Allocation ratio N2/N1 = 1

Output: Noncentrality parameter δ = 3.4097348
Critical t = 1.7247182
Df = 20
Sample size group 1 = 11
Sample size group 2 = 11
Total sample size = 22
Actual power = 0.9501405

Supplementary Figure 8. Sample size calculation by G Power program.
