# Supplementary material for: Sera from women with different metabolic and menopause states differentially regulate cell viability and Akt activation in a breast cancer in-vitro model
Source: PLoS One. 2022 Apr 12;17(4):e0266073. doi: 10.1371/journal.pone.0266073 (PMC9004774; doi:10.1371/journal.pone.0266073)

Figure 2B

NWSPre sera

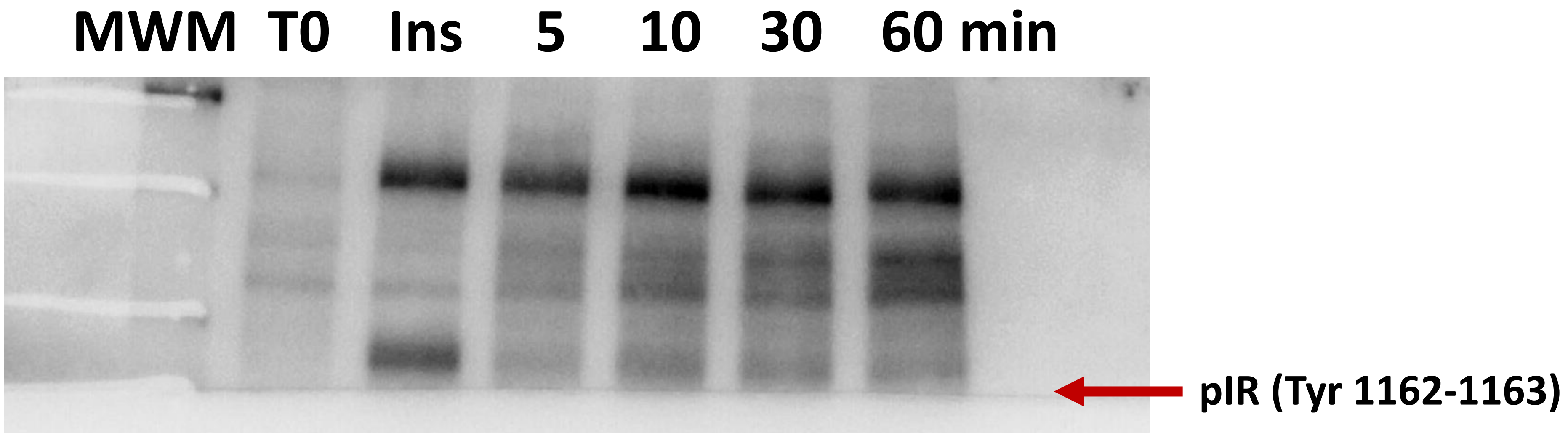

Figure 2B

NWSPre sera

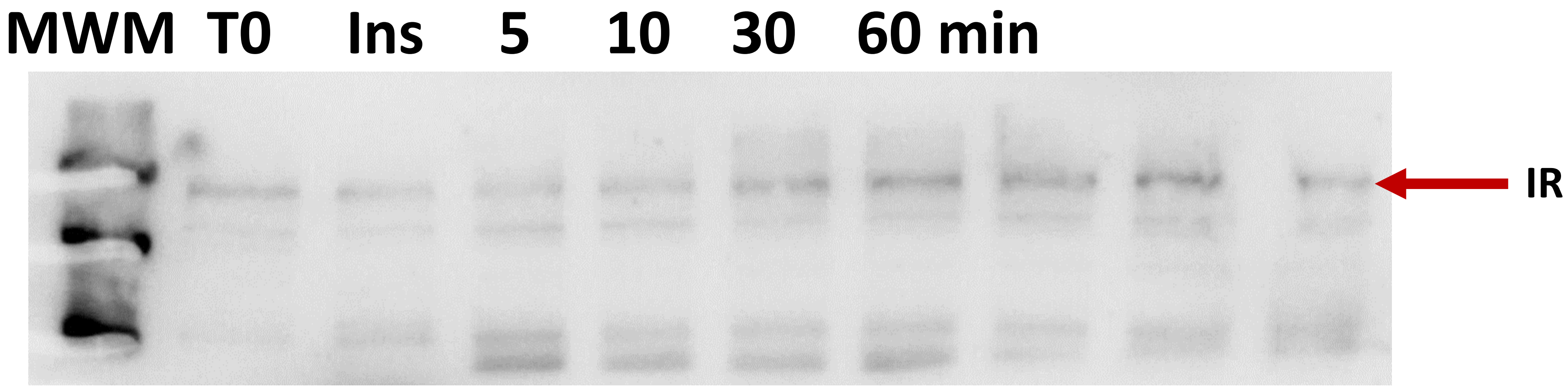

Figure 2B

NWSPre sera

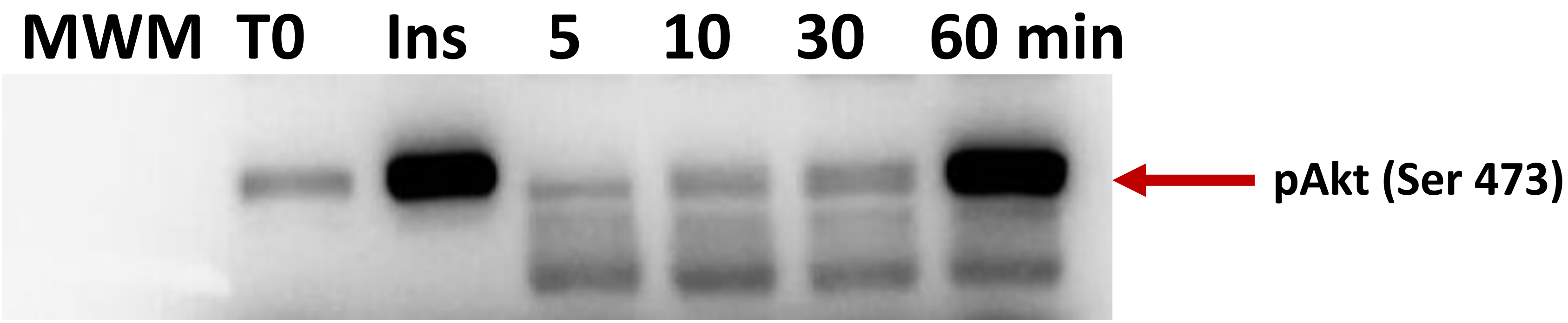

Figure 2B

NWSPre sera

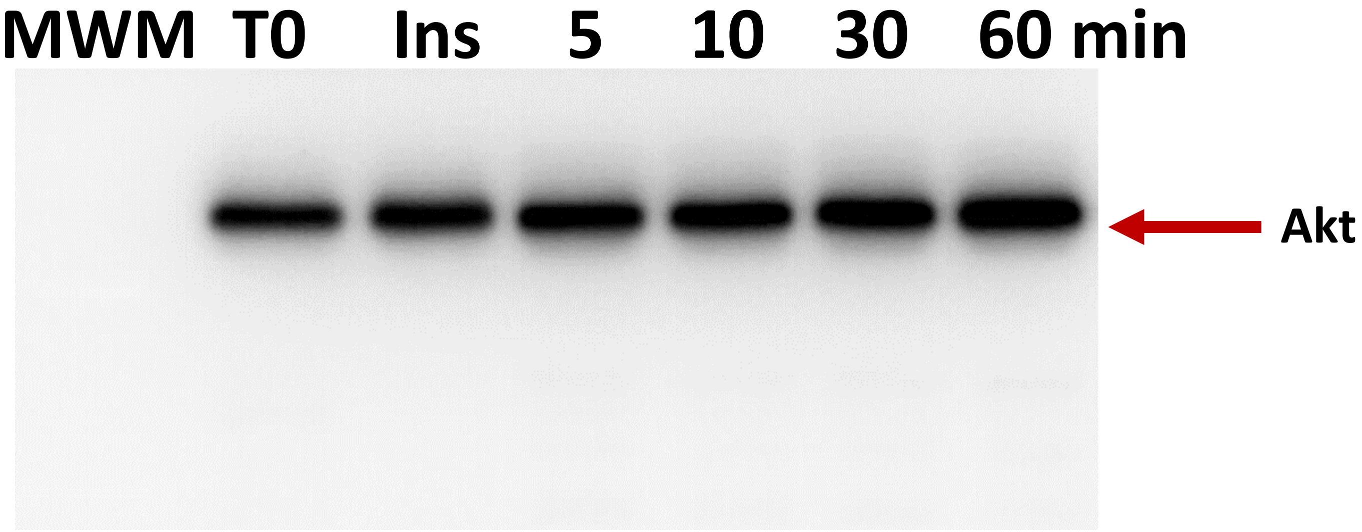

Figure 2B

NWSPre sera

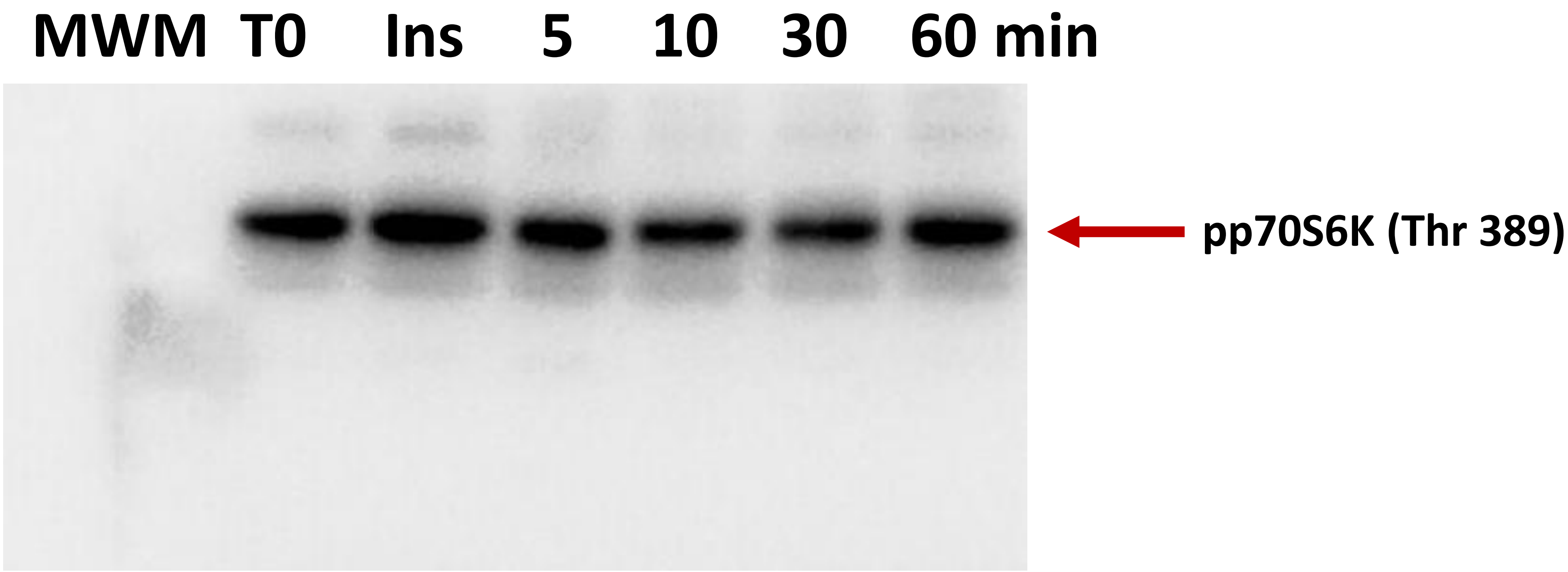

Figure 2B

NWSPre sera

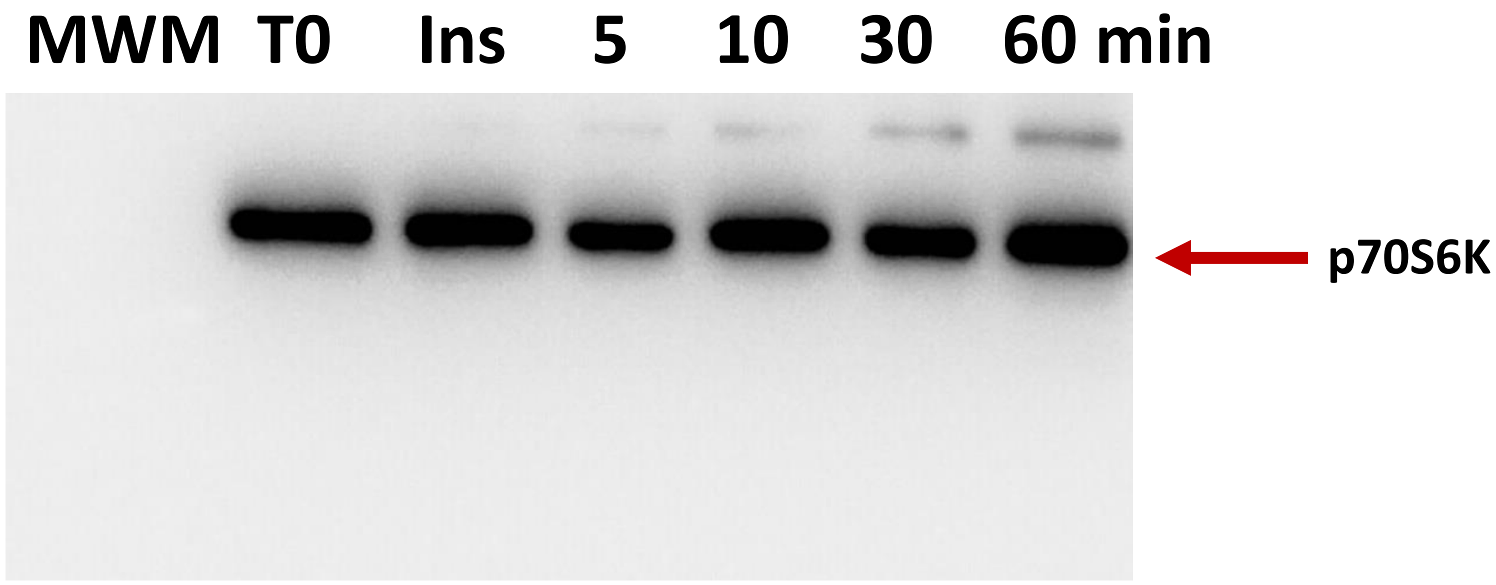

Figure 2B

NWSPre sera

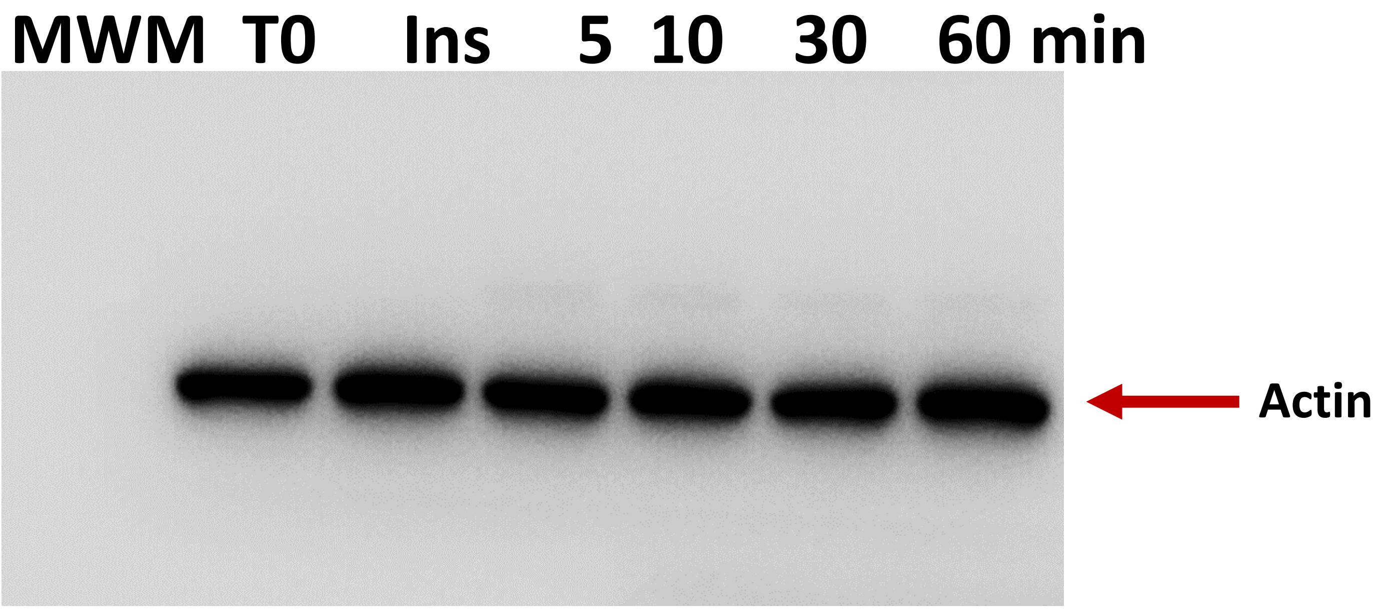

Figure 2B

OSPre sera

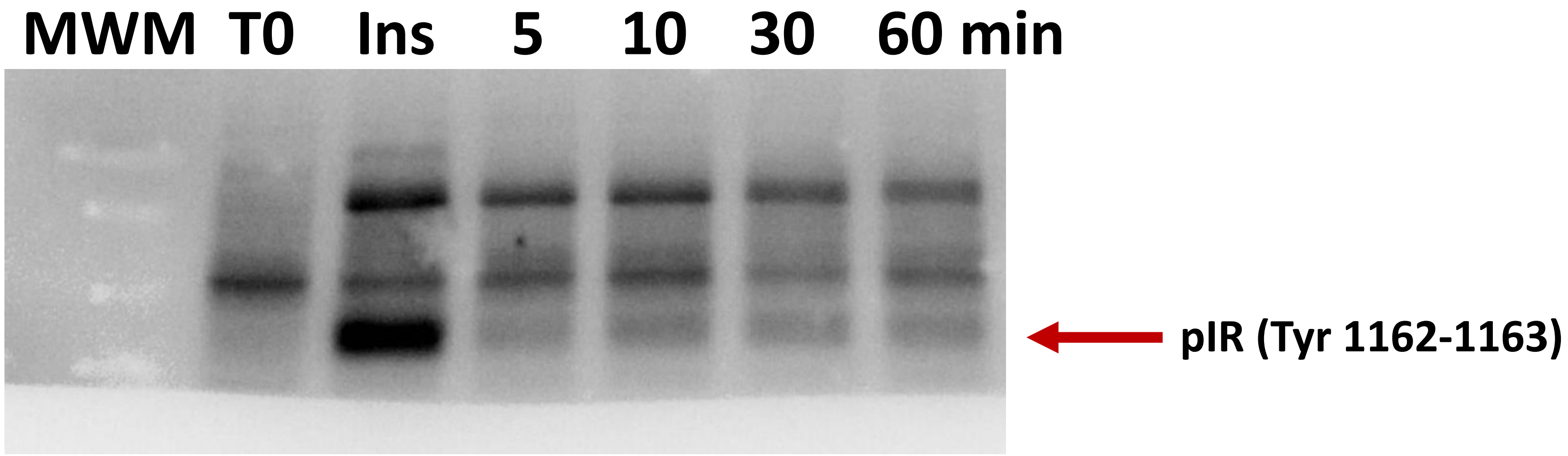

Figure 2B

OSPre sera

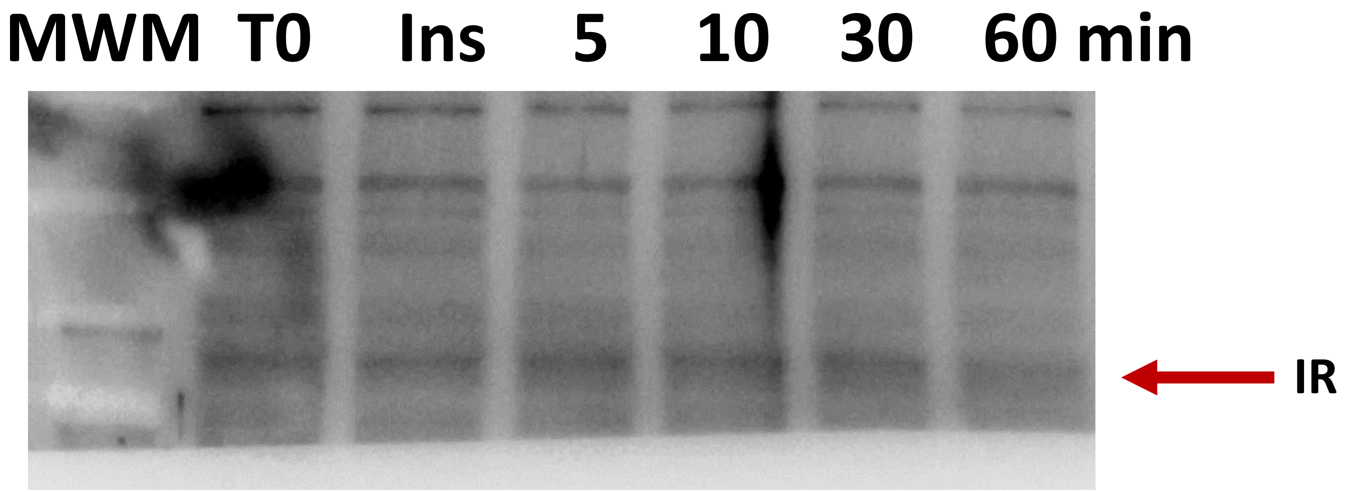

Figure 2B

OSPre sera

MWM T0 Ins 5 10 30 60 min

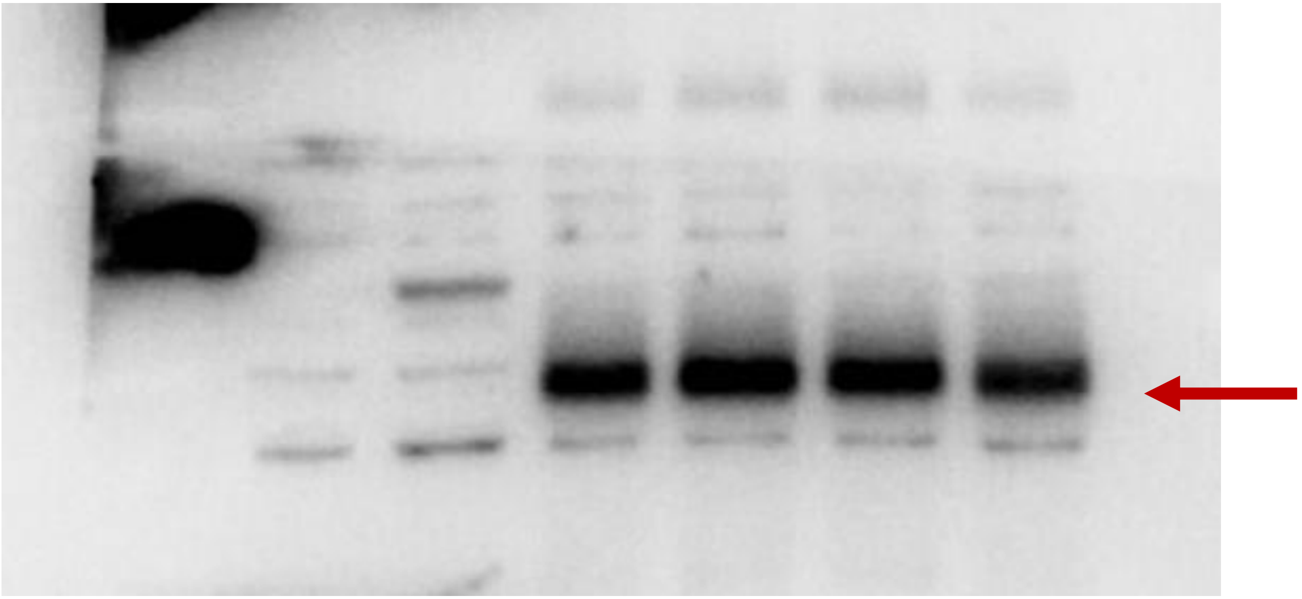

pAkt (Ser 473)

Figure 2B

OSPre sera

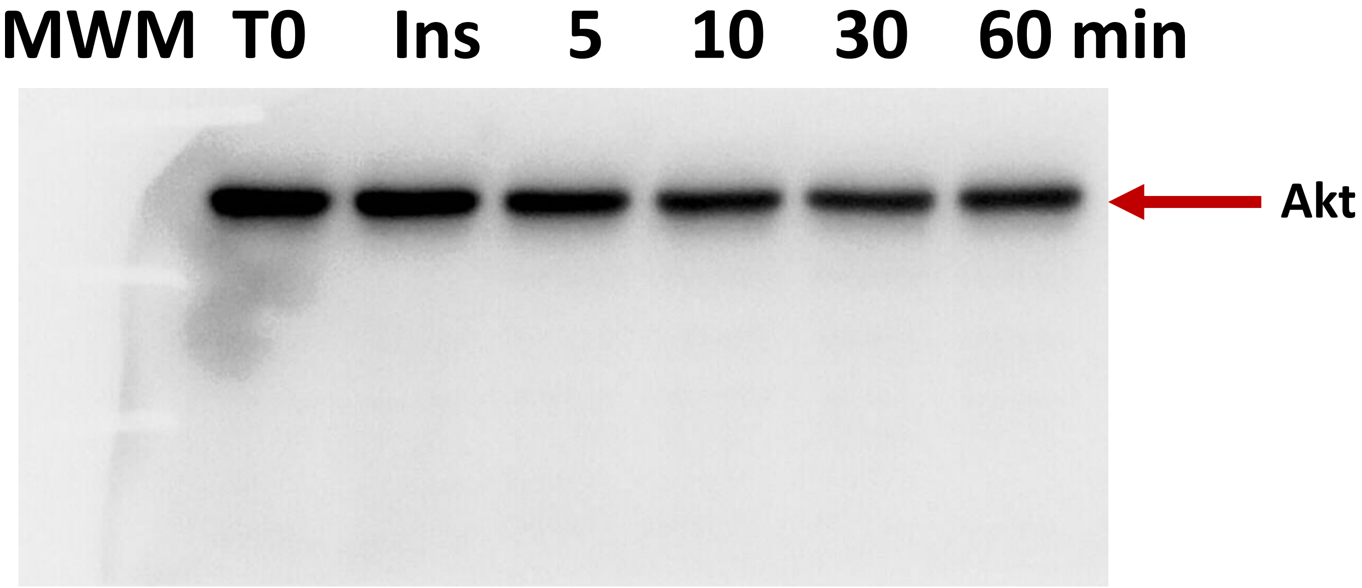

Figure 2B

OSPre sera

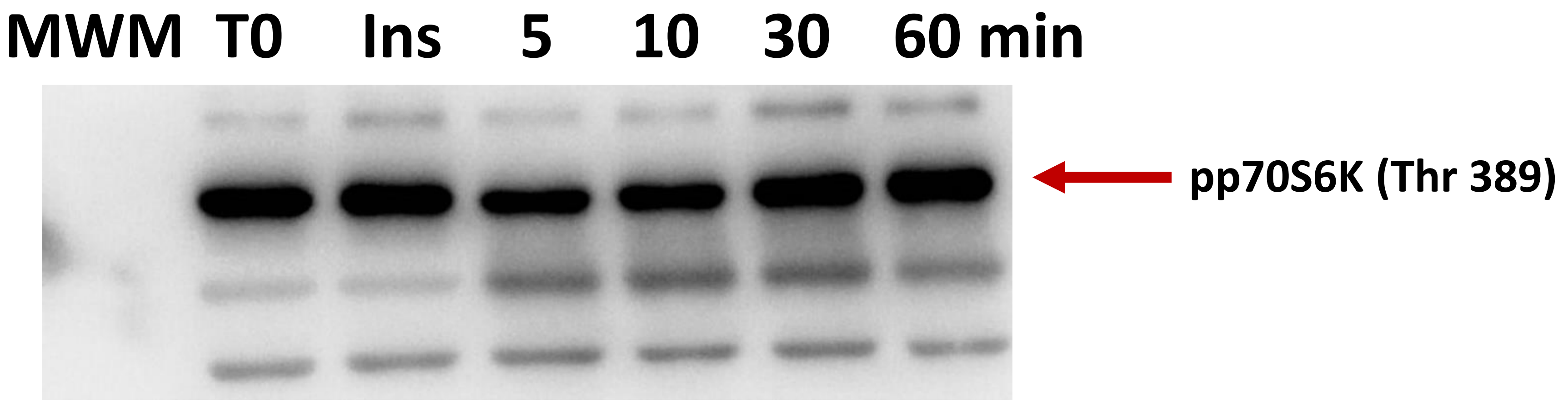

Figure 2B

OSPre sera

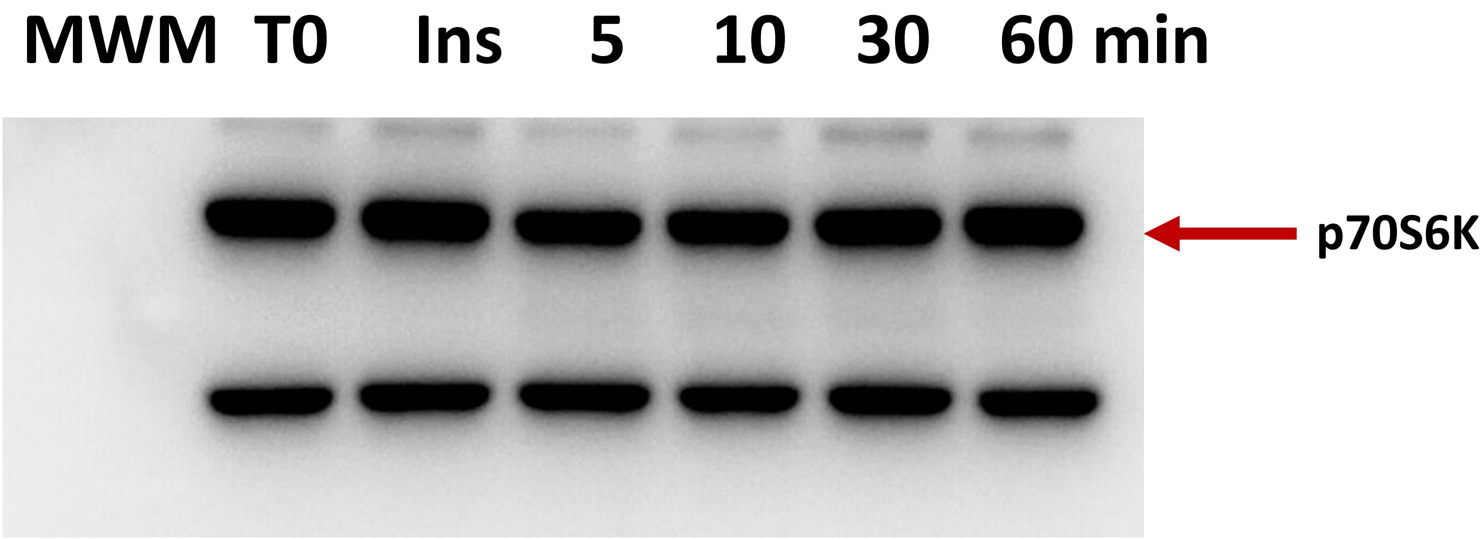

Figure 2B

OSPre sera

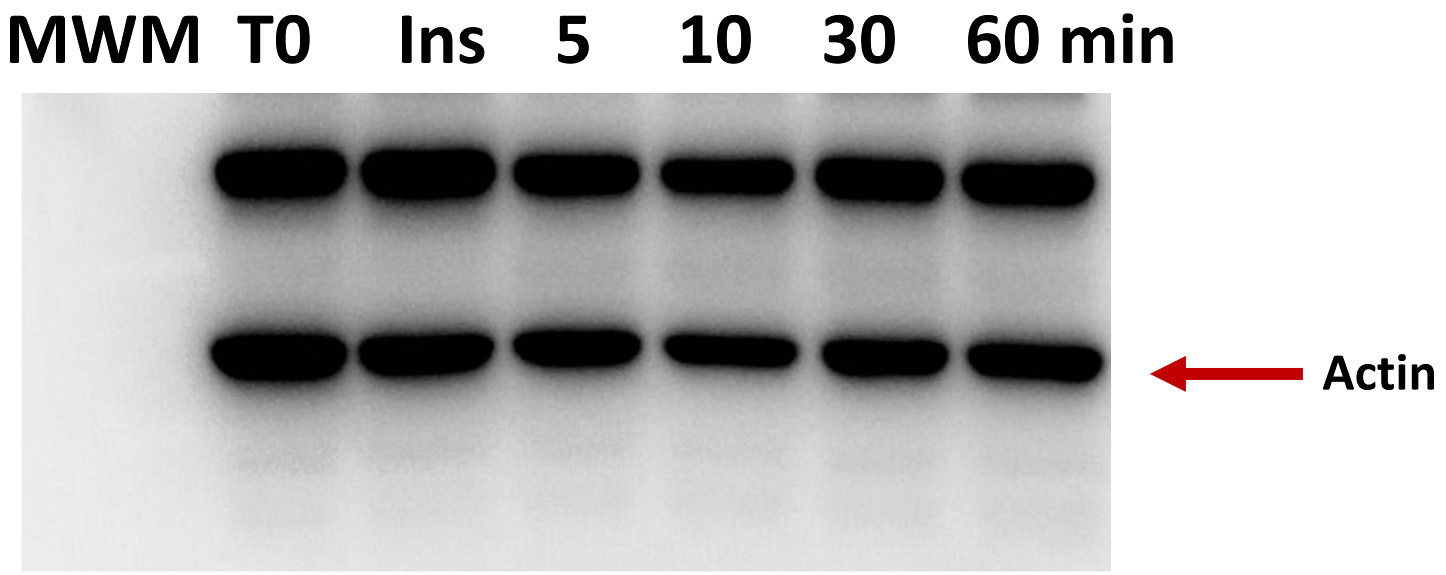

Figure 2F

NWSPre sera

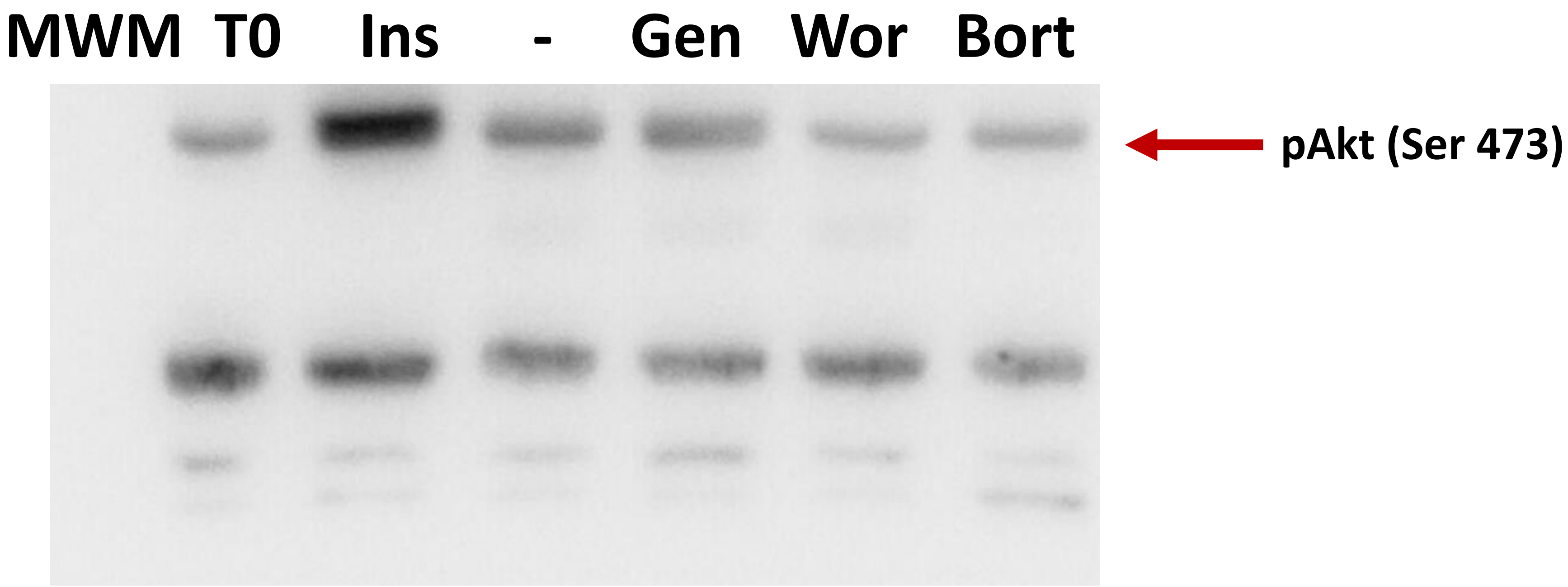

Figure 2F

NWSPre sera

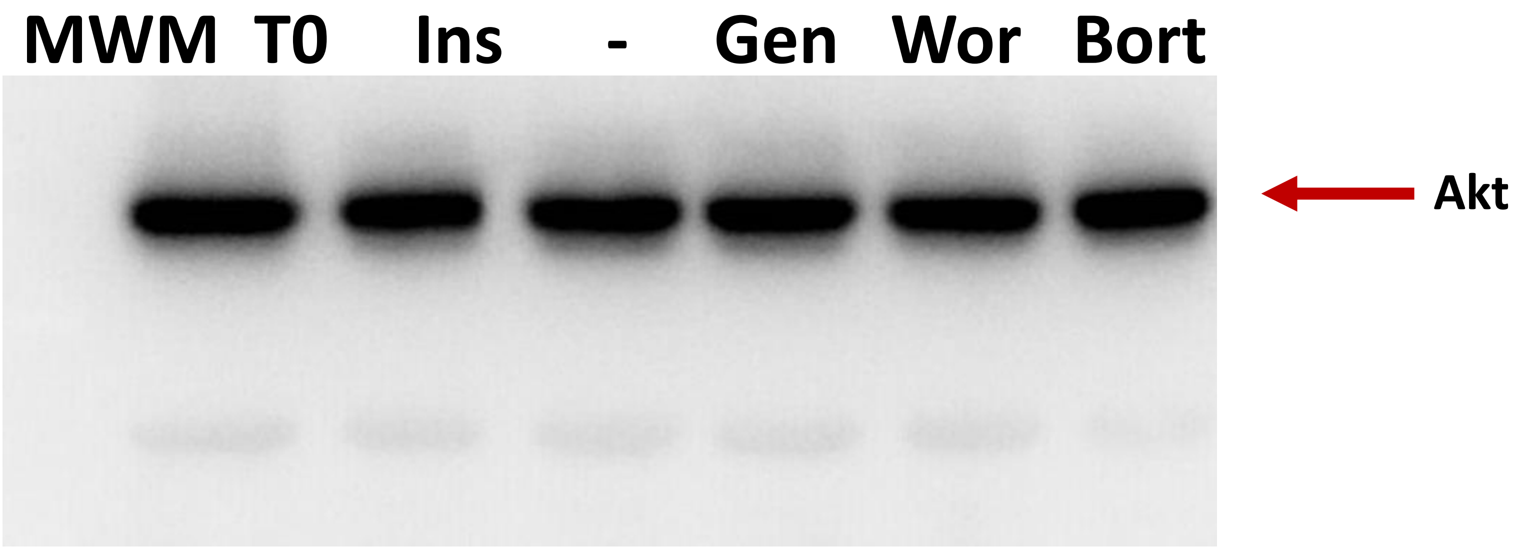

Figure 2F

NWSPre sera

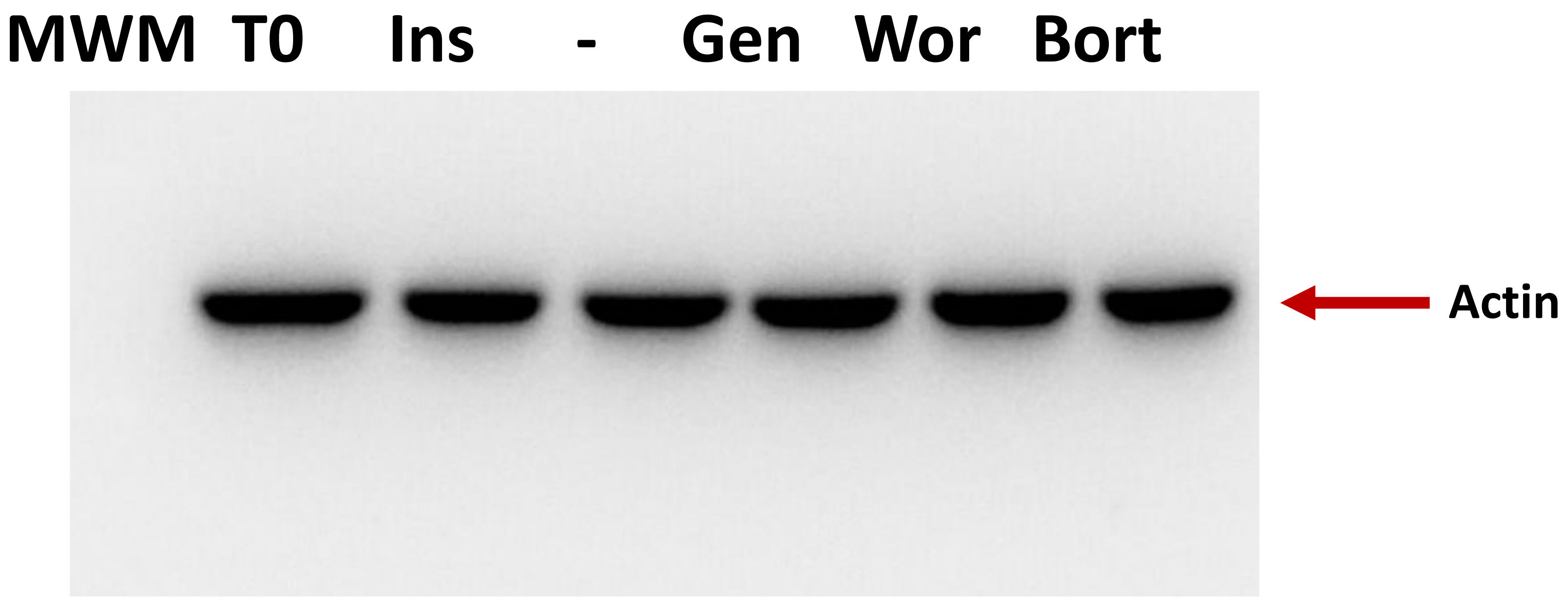

Figure 2F

OSPre sera

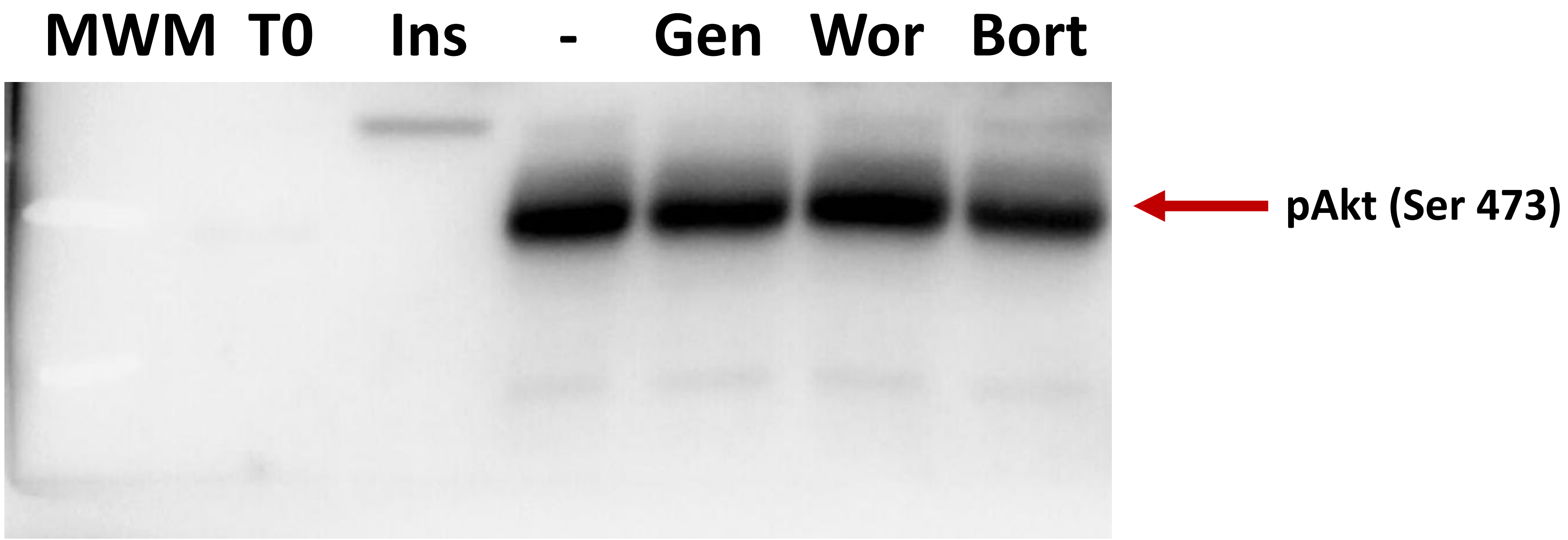

Figure 2F

OSPre sera

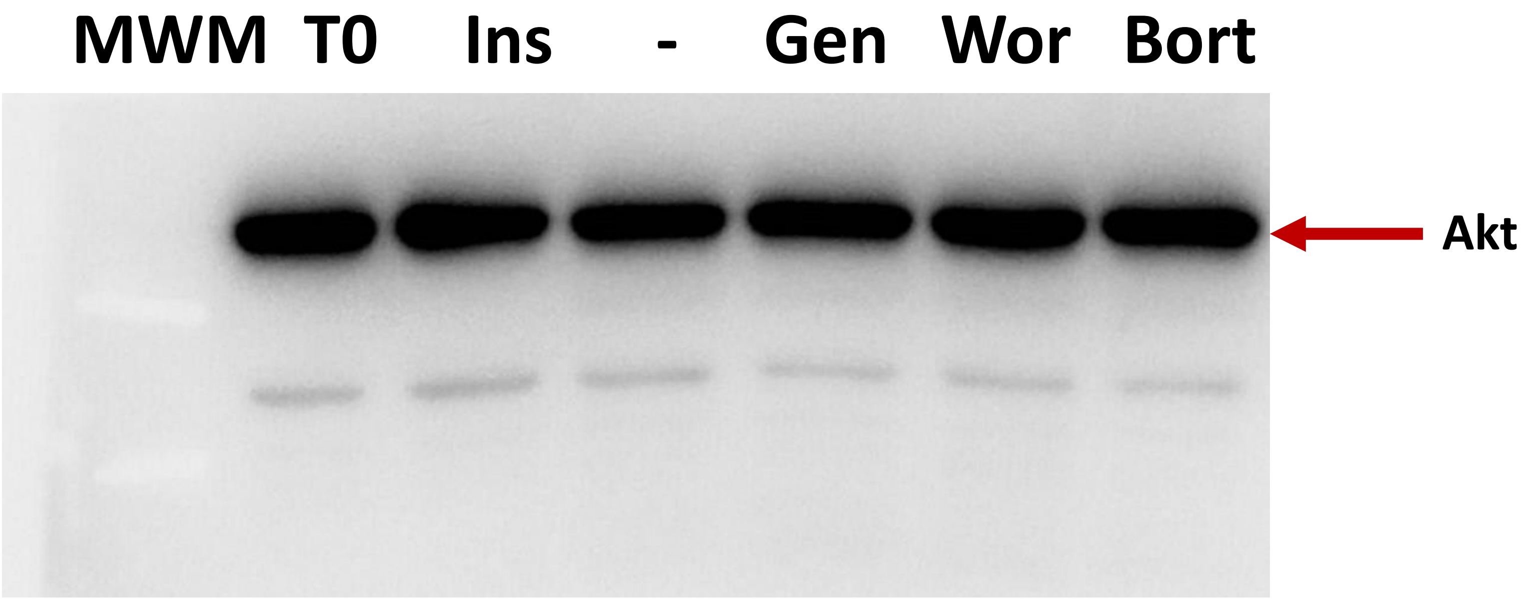

Figure 2F

OSPre sera

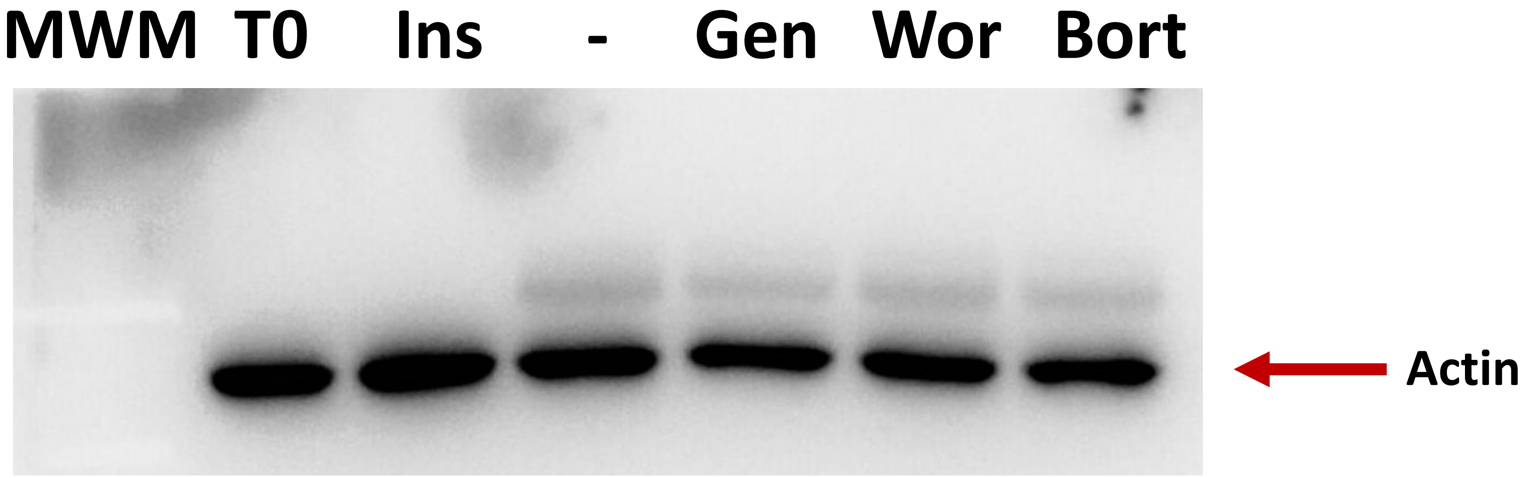

Figure 3B

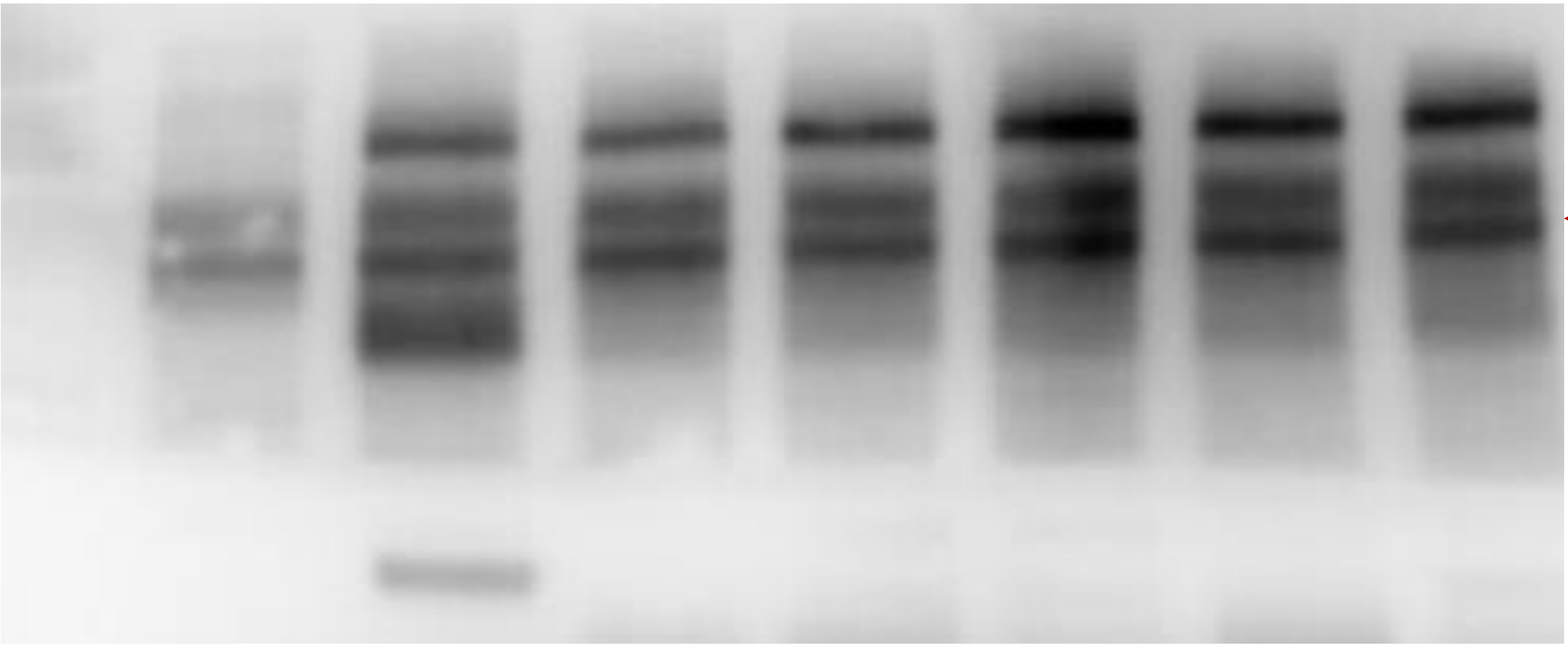

Figure 3B

NWSPre sera

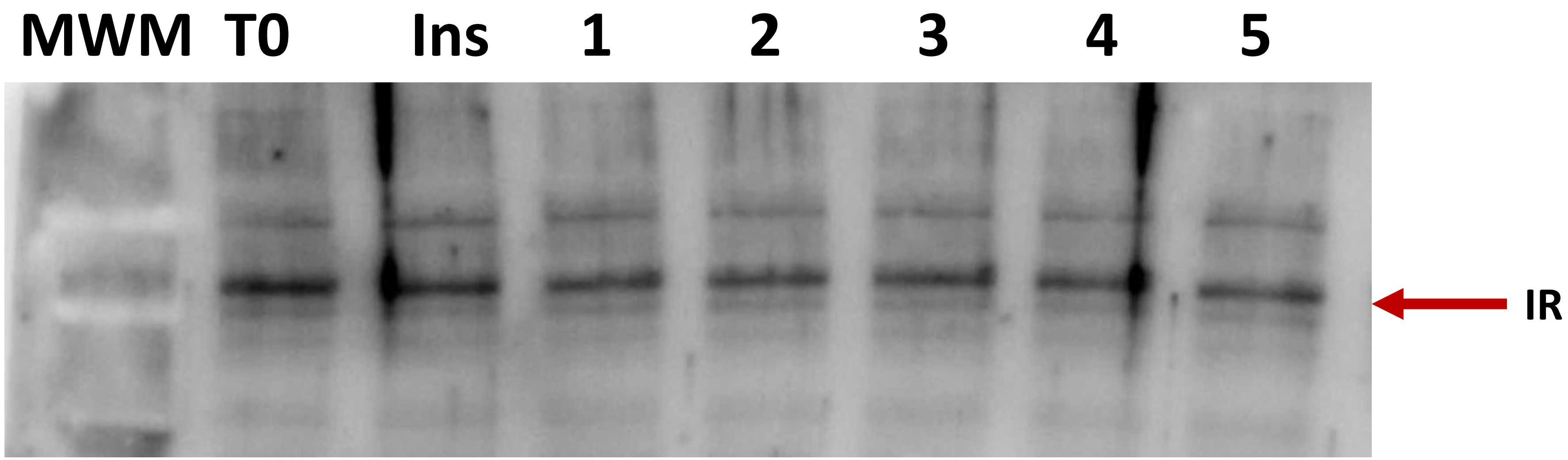

Figure 3B

NWSPre sera

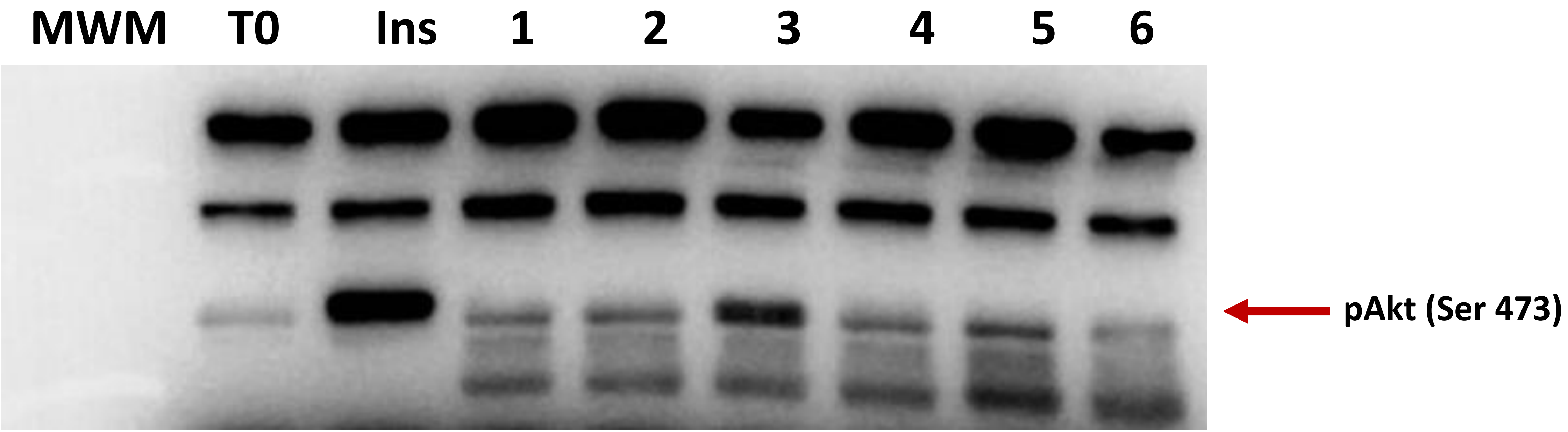

Figure 3B

NWSPre sera

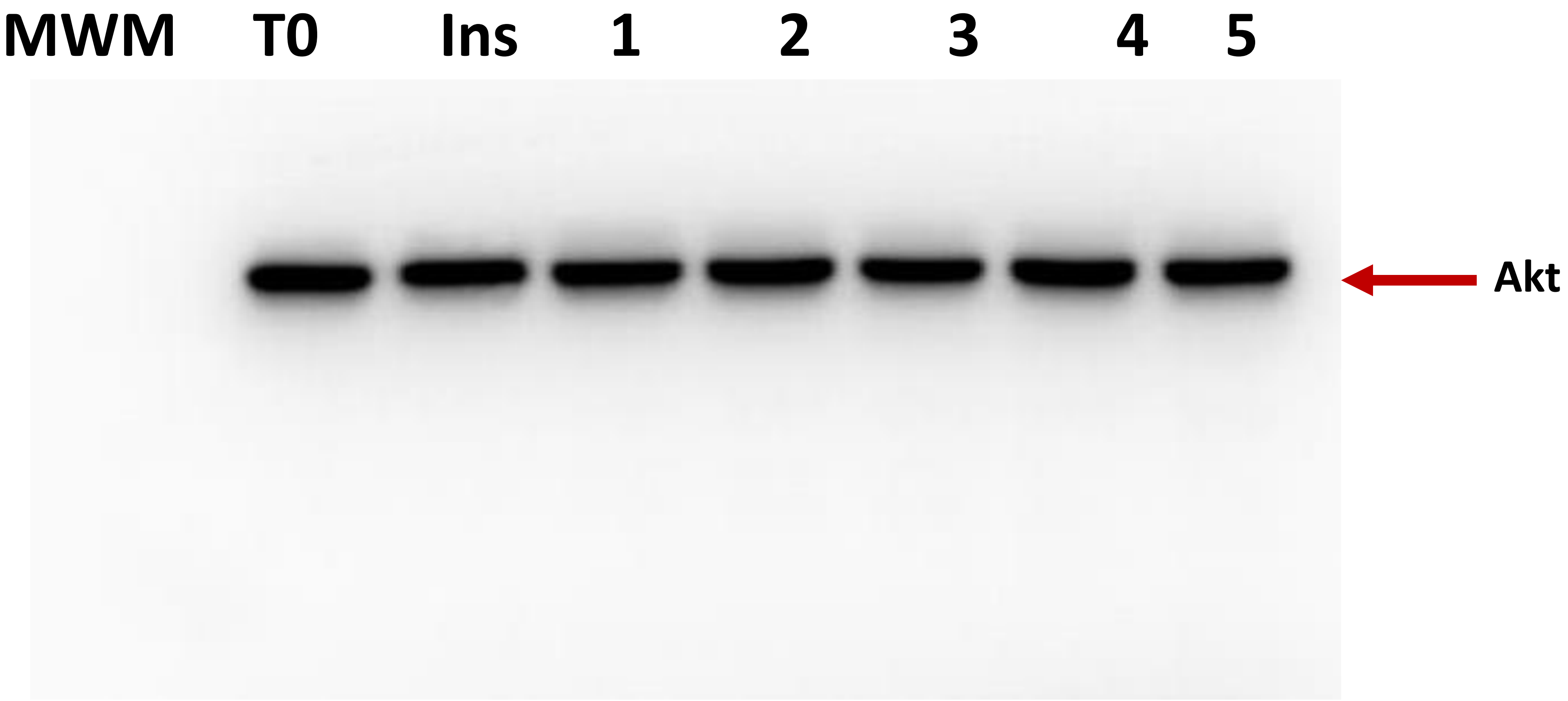

Figure 3B

NWSPre sera

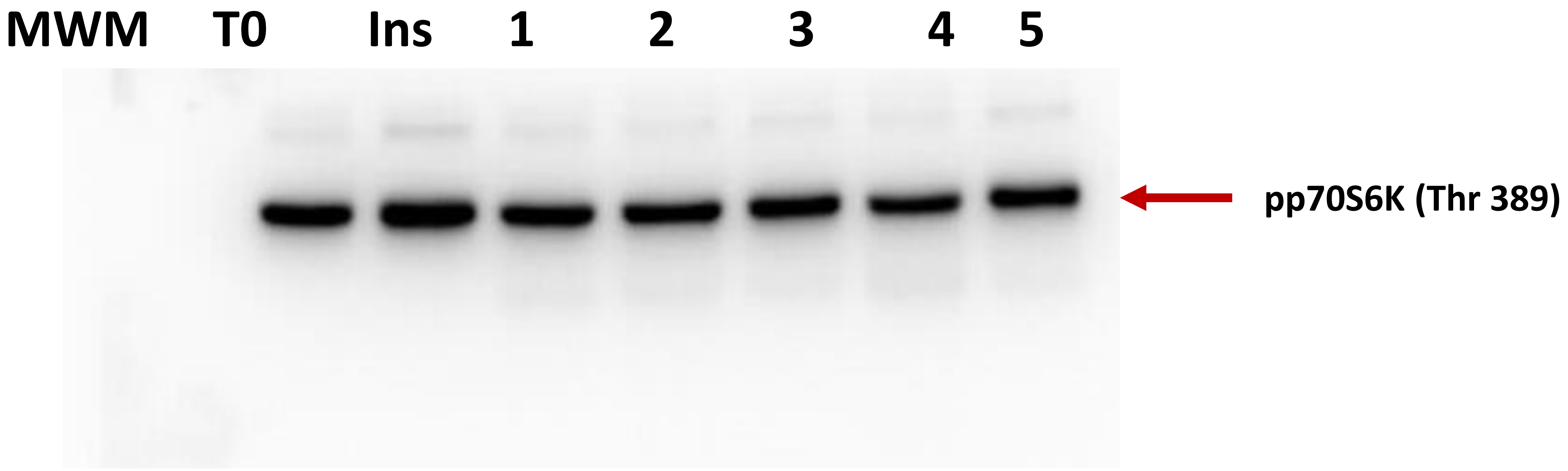

Figure 3B

NWSPre sera

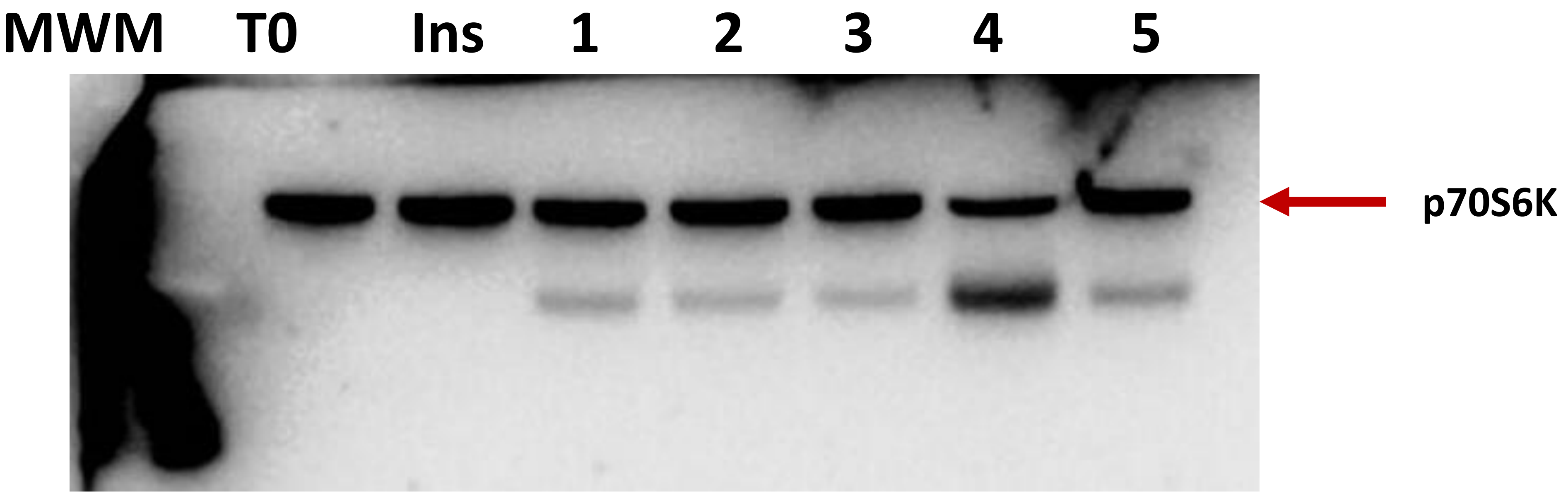

Figure 3B

NWSPre sera

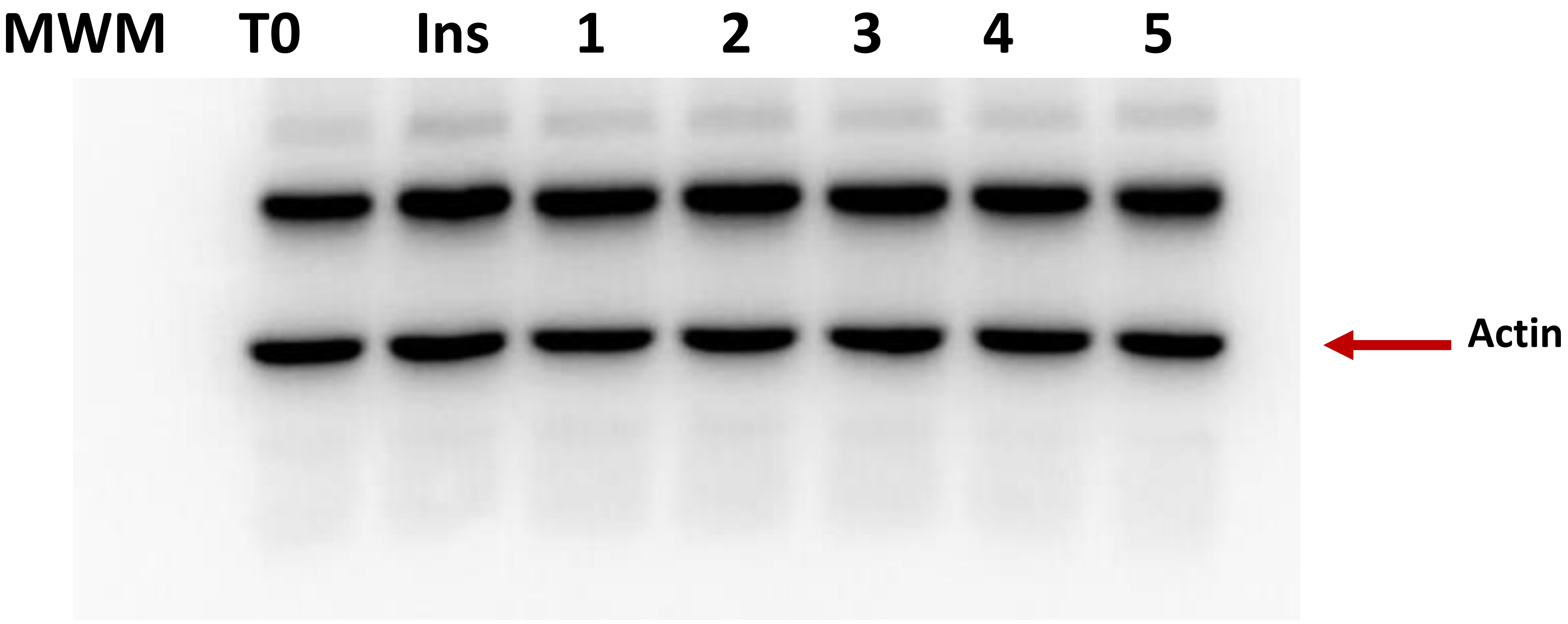

Figure 3B

OSPre sera

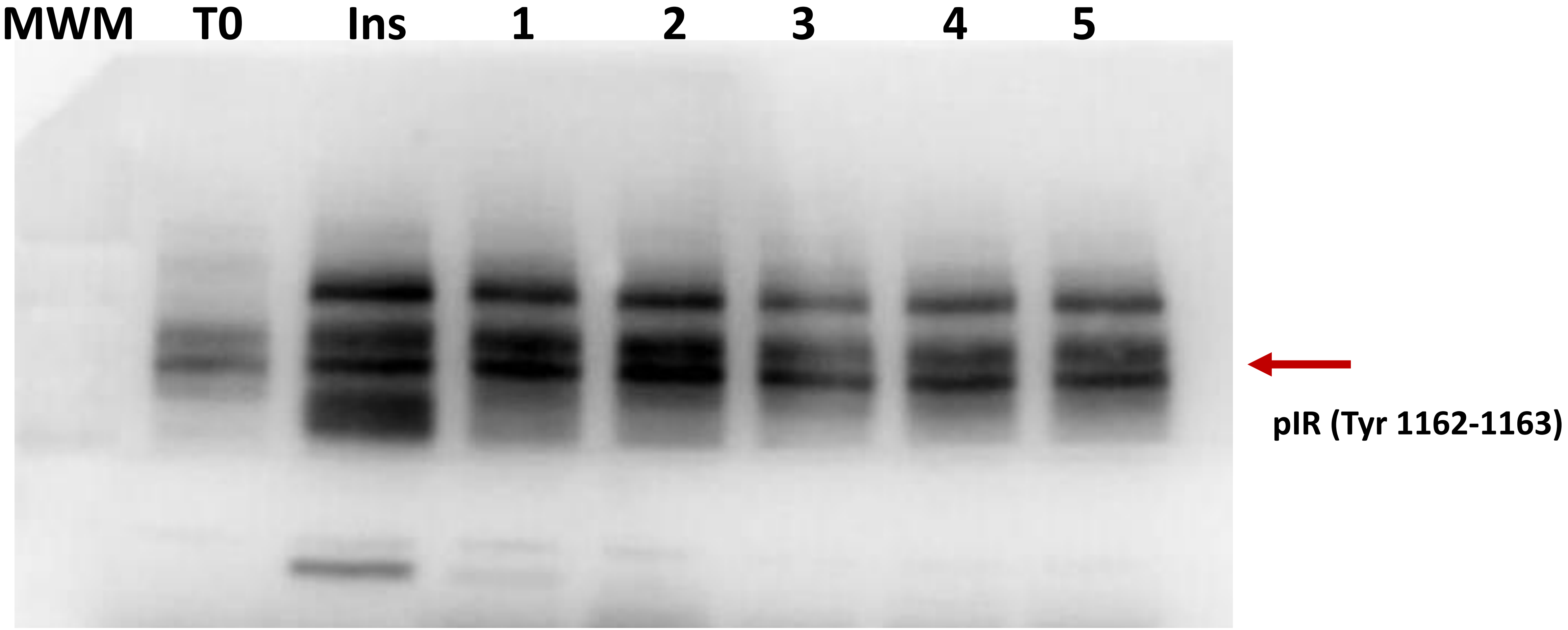

Figure 3B

OSPre sera

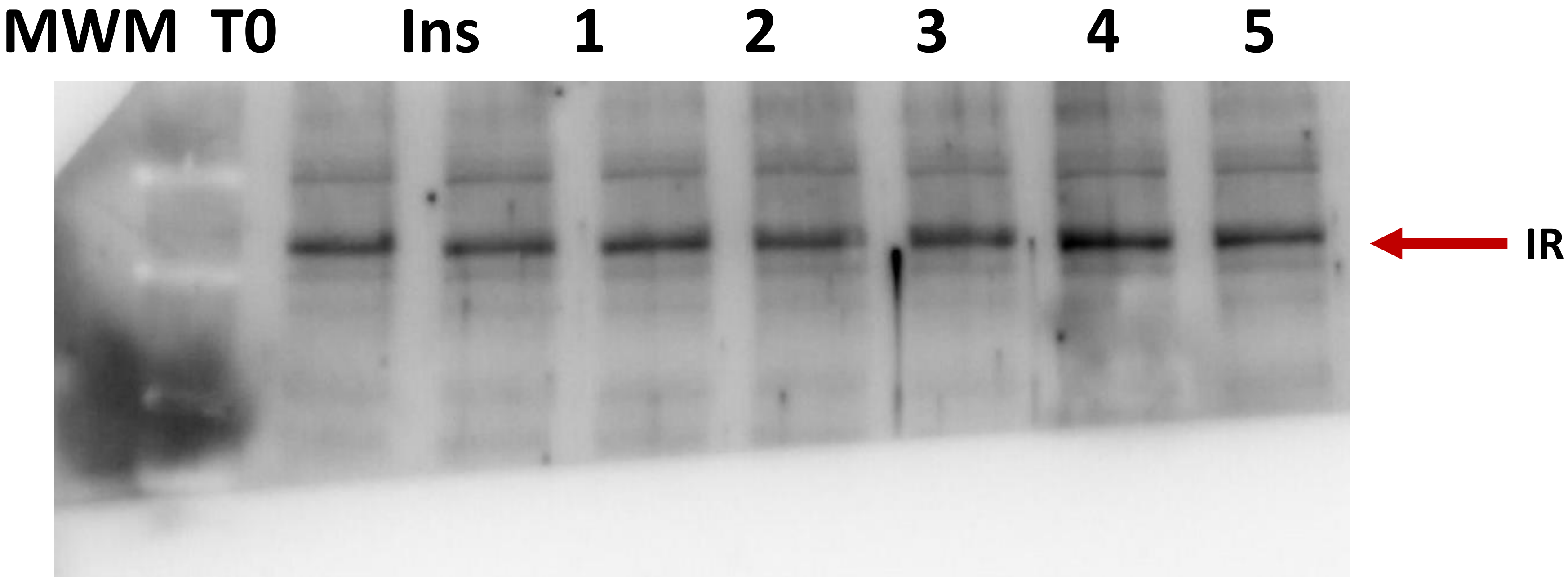

Figure 3B

OSPre sera

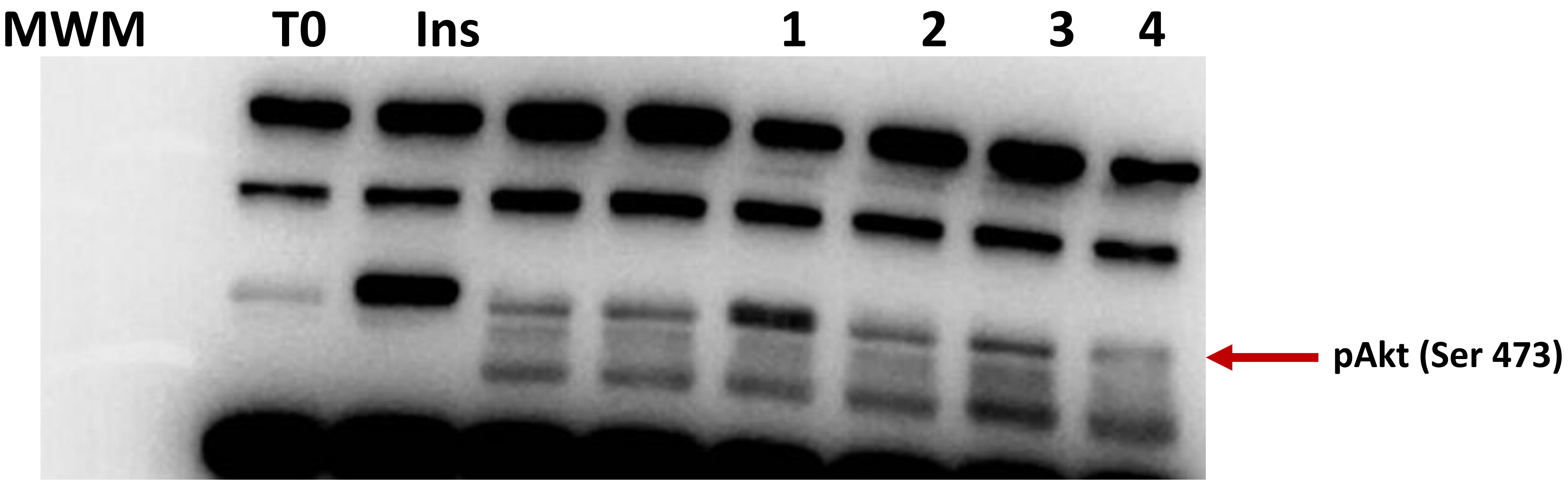

Figure 3B

OSPre sera

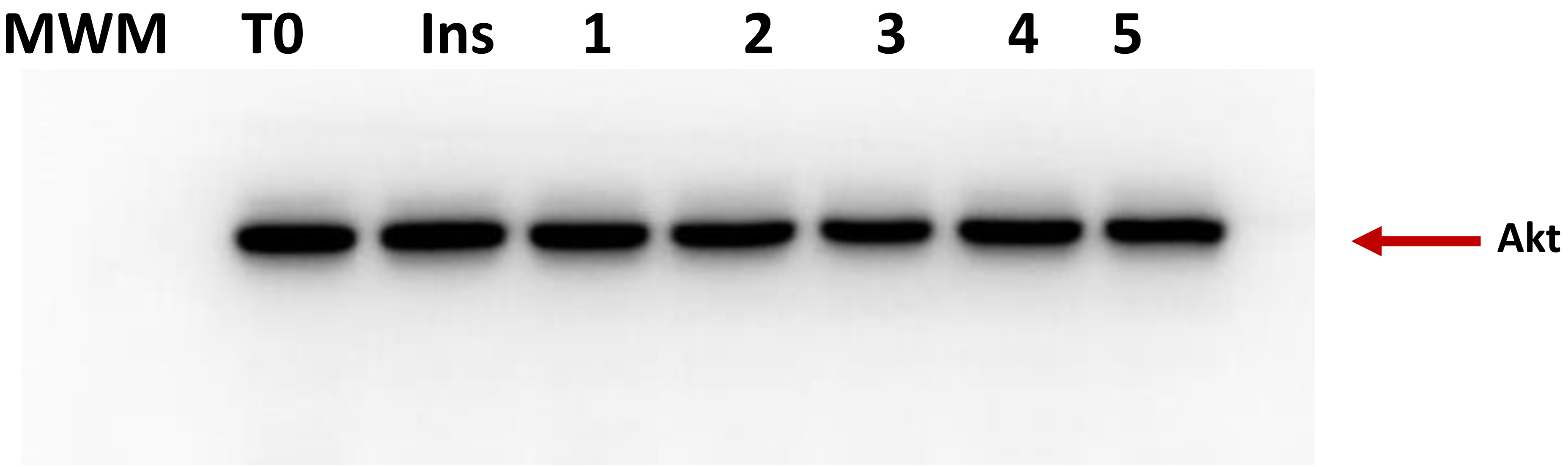

Figure 3B

OSPre sera

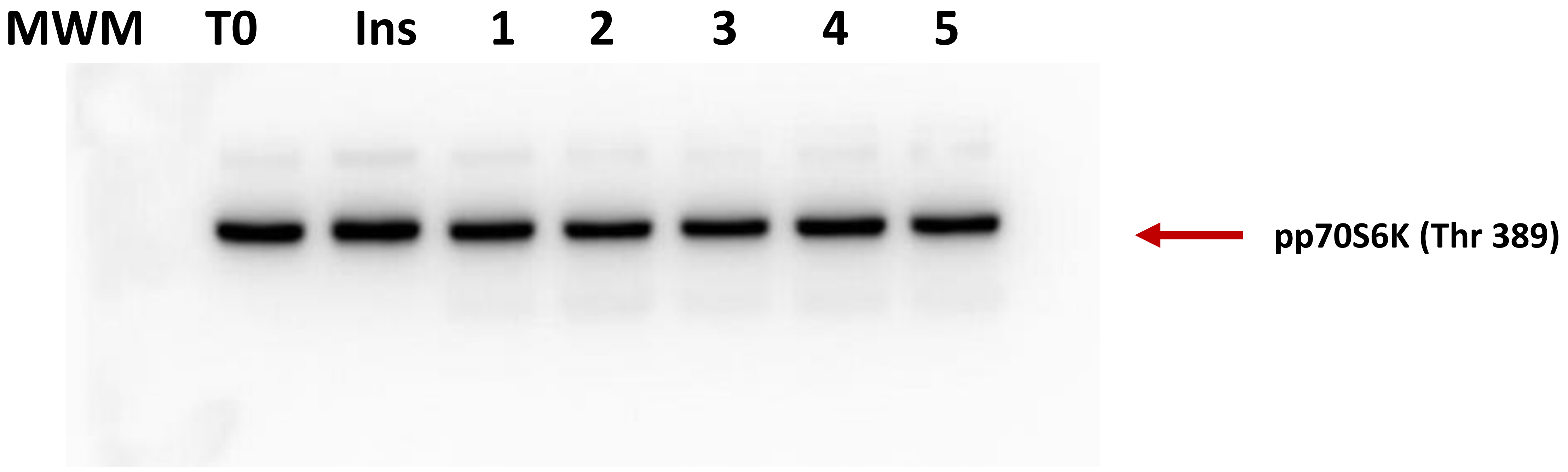

Figure 3B

OSPre sera

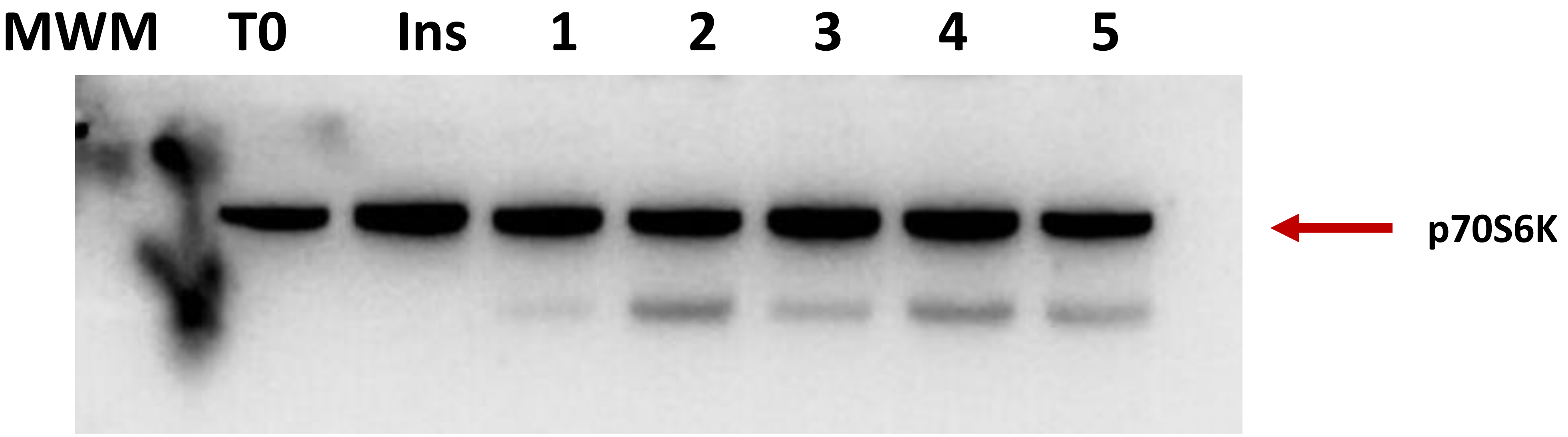

Figure 3B

OSPre sera

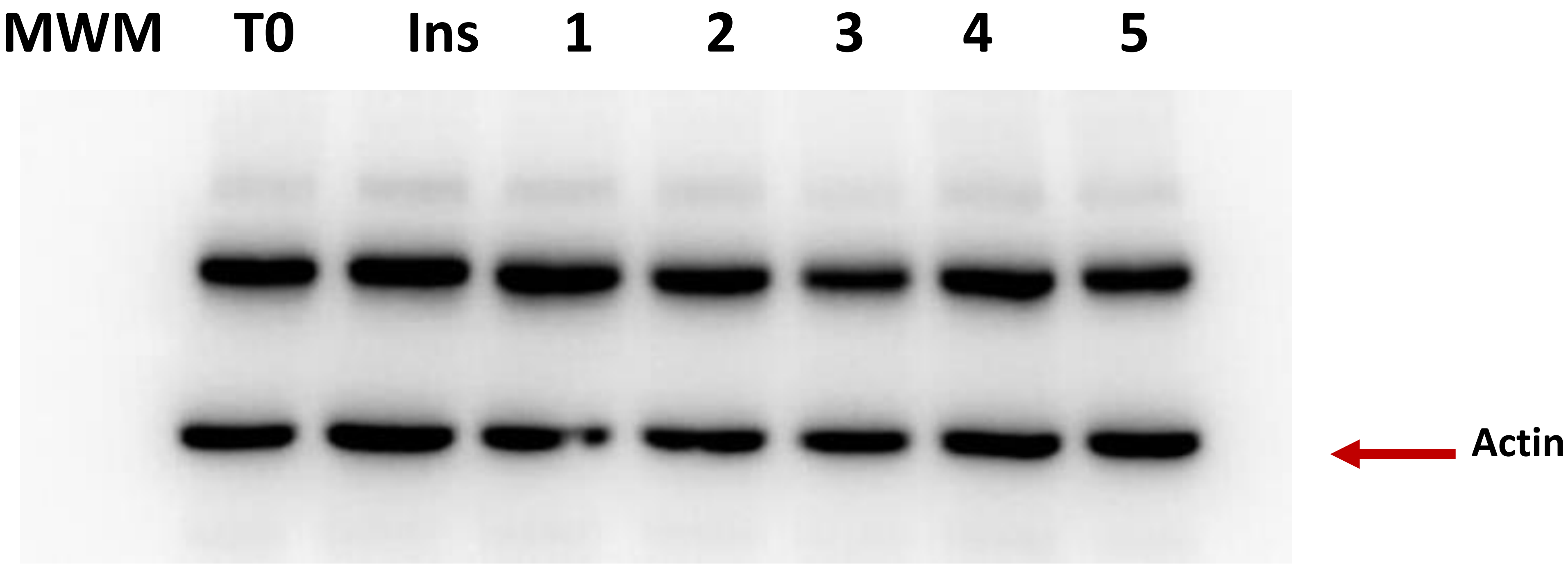

Figure 3B

OSPost and NWSPost sera

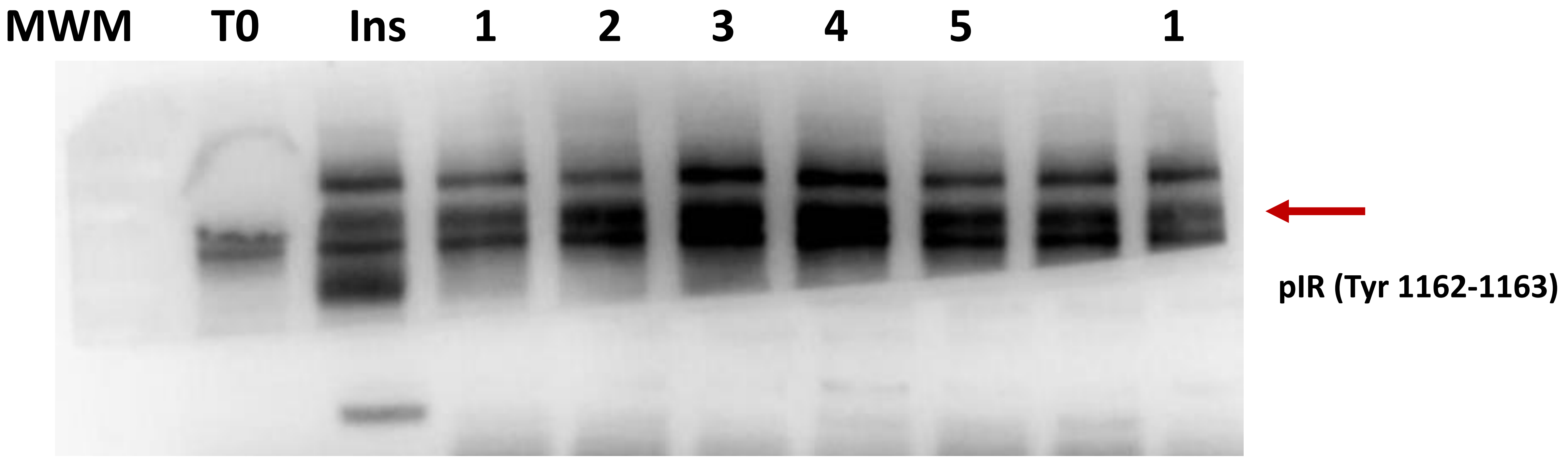

Figure 3B

OSPost and NWSPost sera

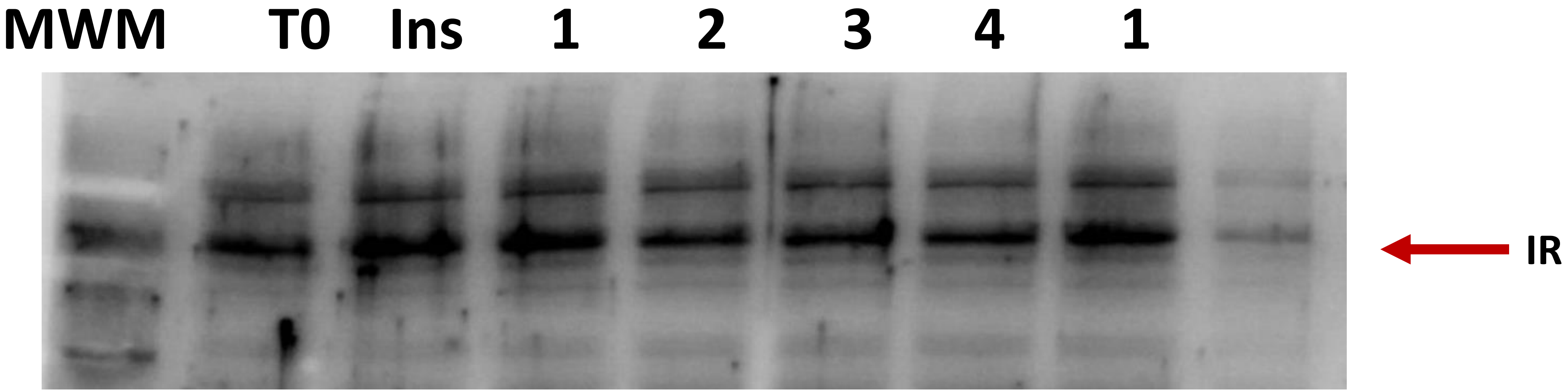

Figure 3B

OSPost and NWSPost sera

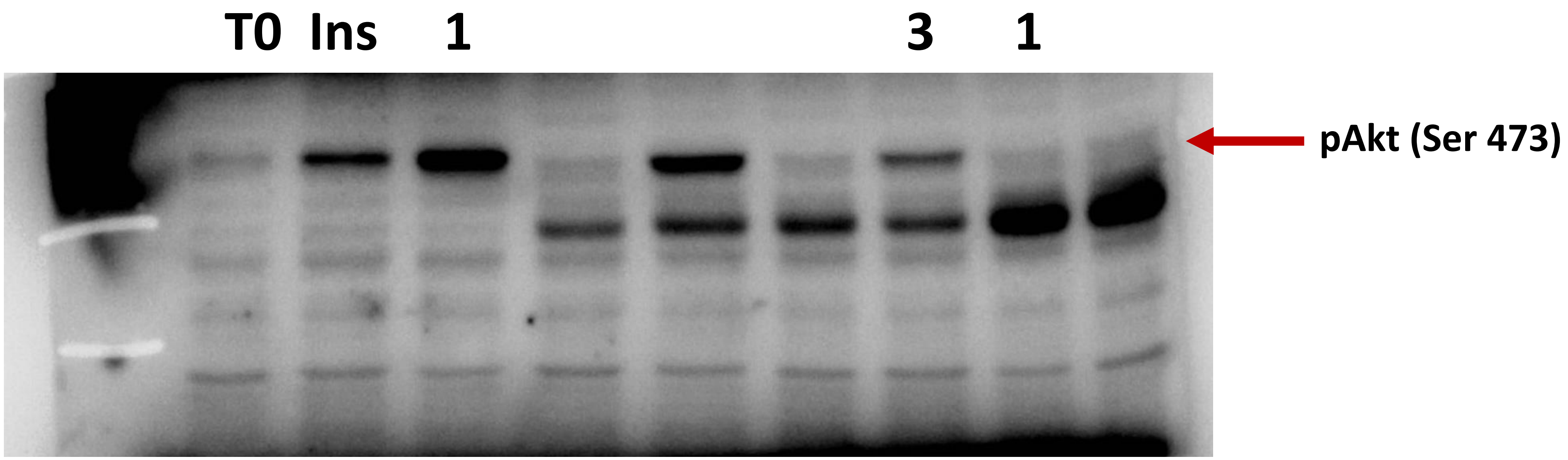

Figure 3B

OSPost and NWSPost sera

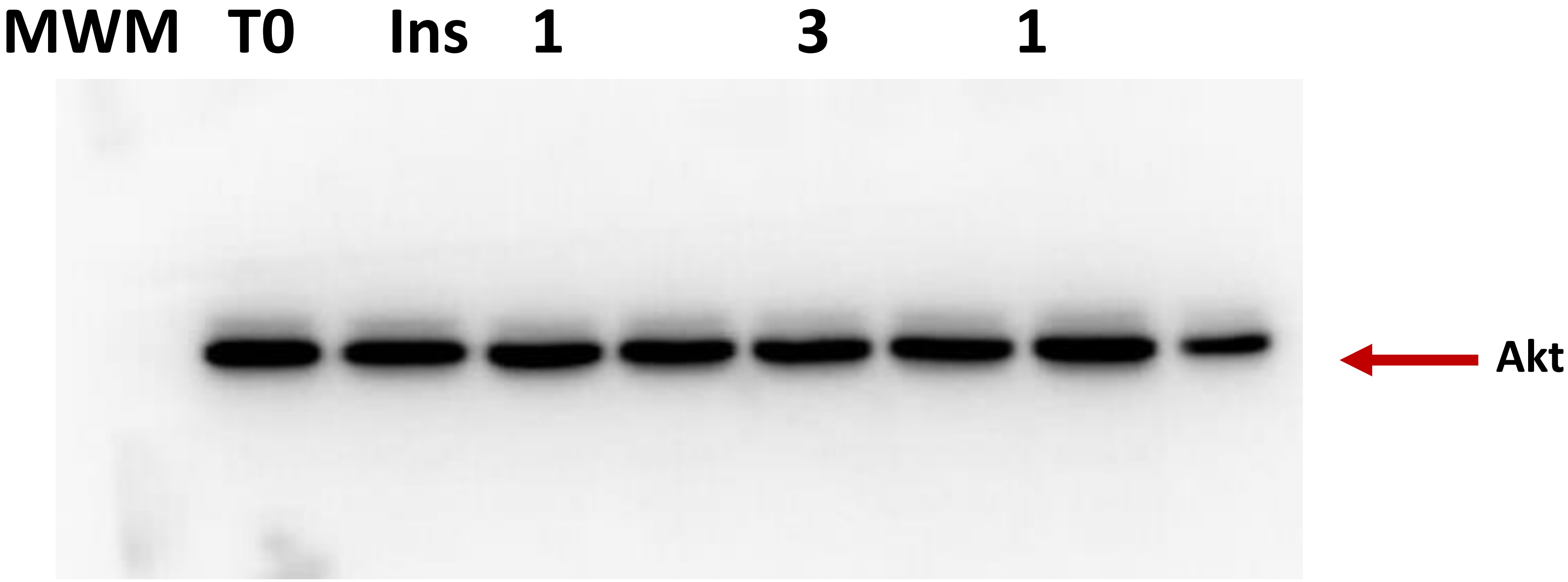

Figure 3B

OSPost and NWSPost sera

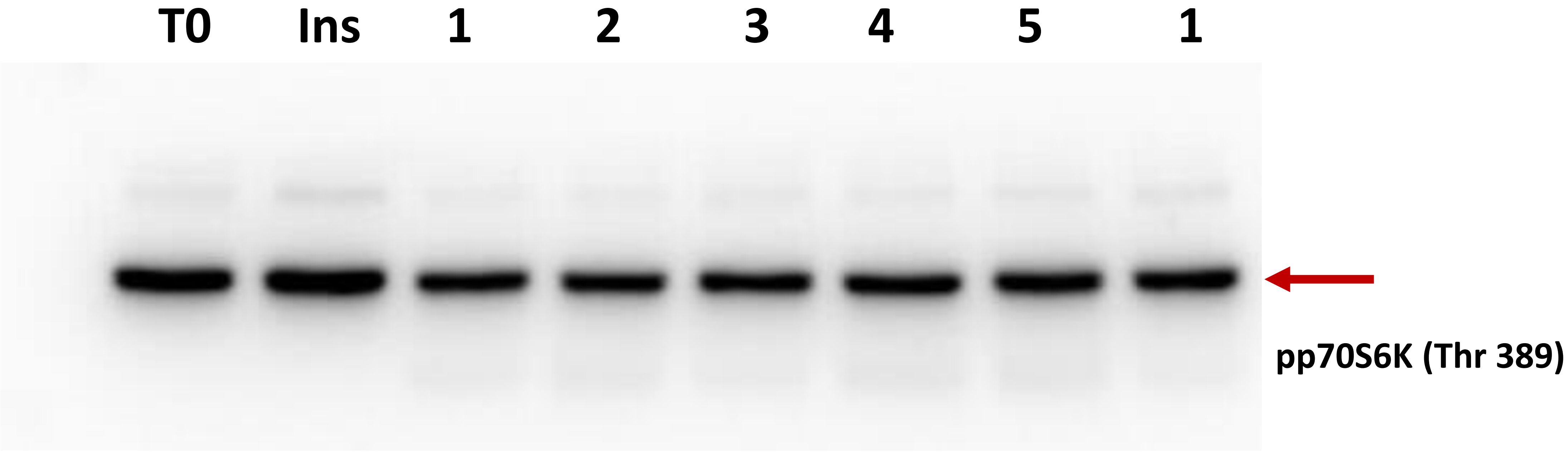

Figure 3B

OSPost and NWSPost sera

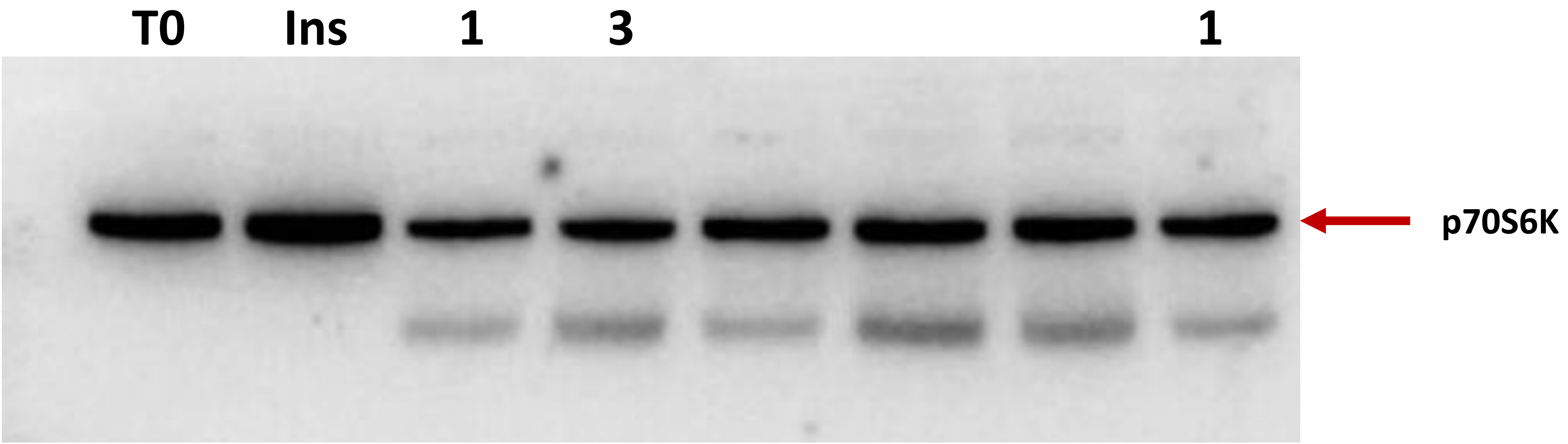

Figure 3B

OSPost and NWSPost sera

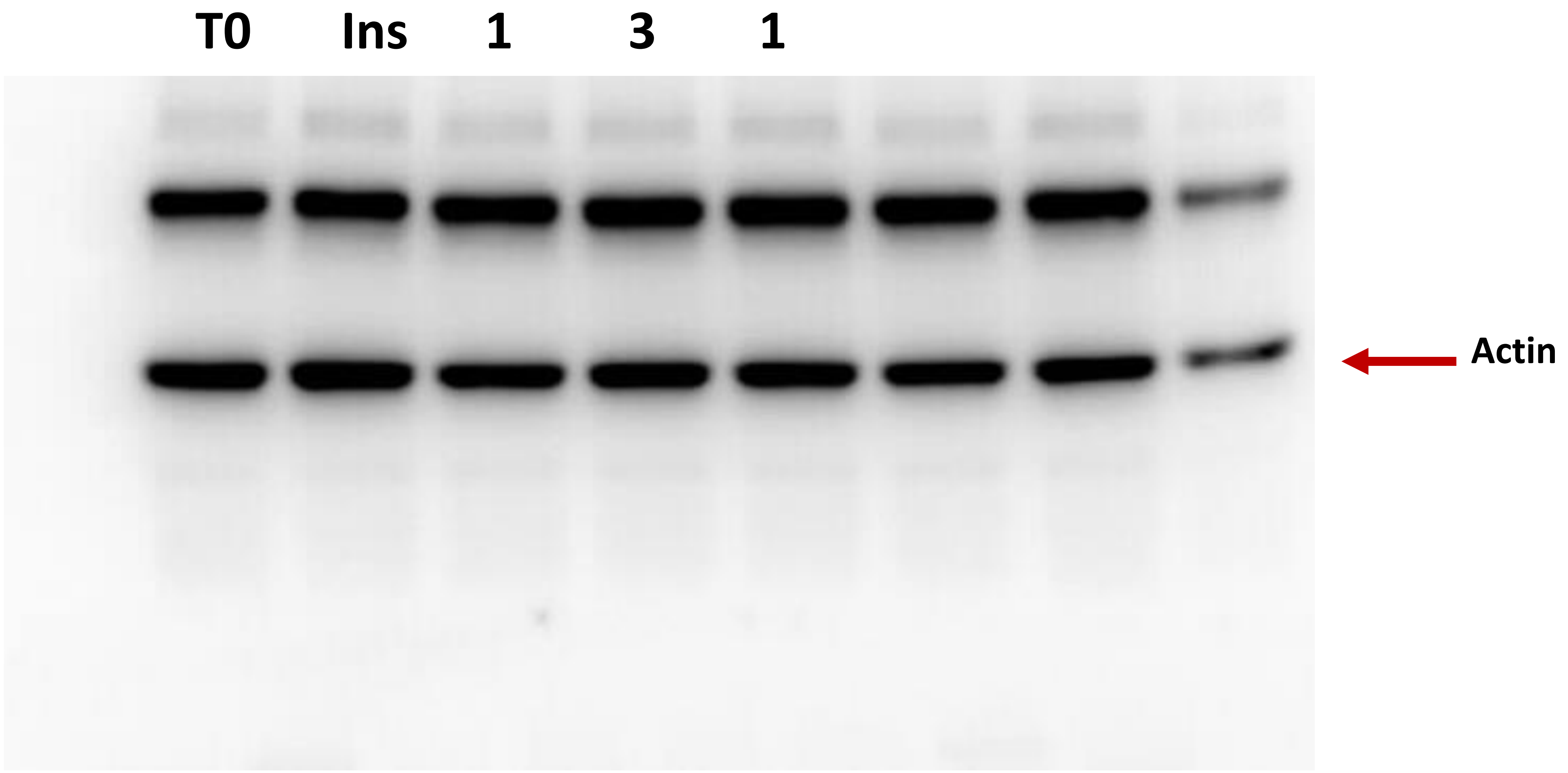

Figure 4B

OSPost sera

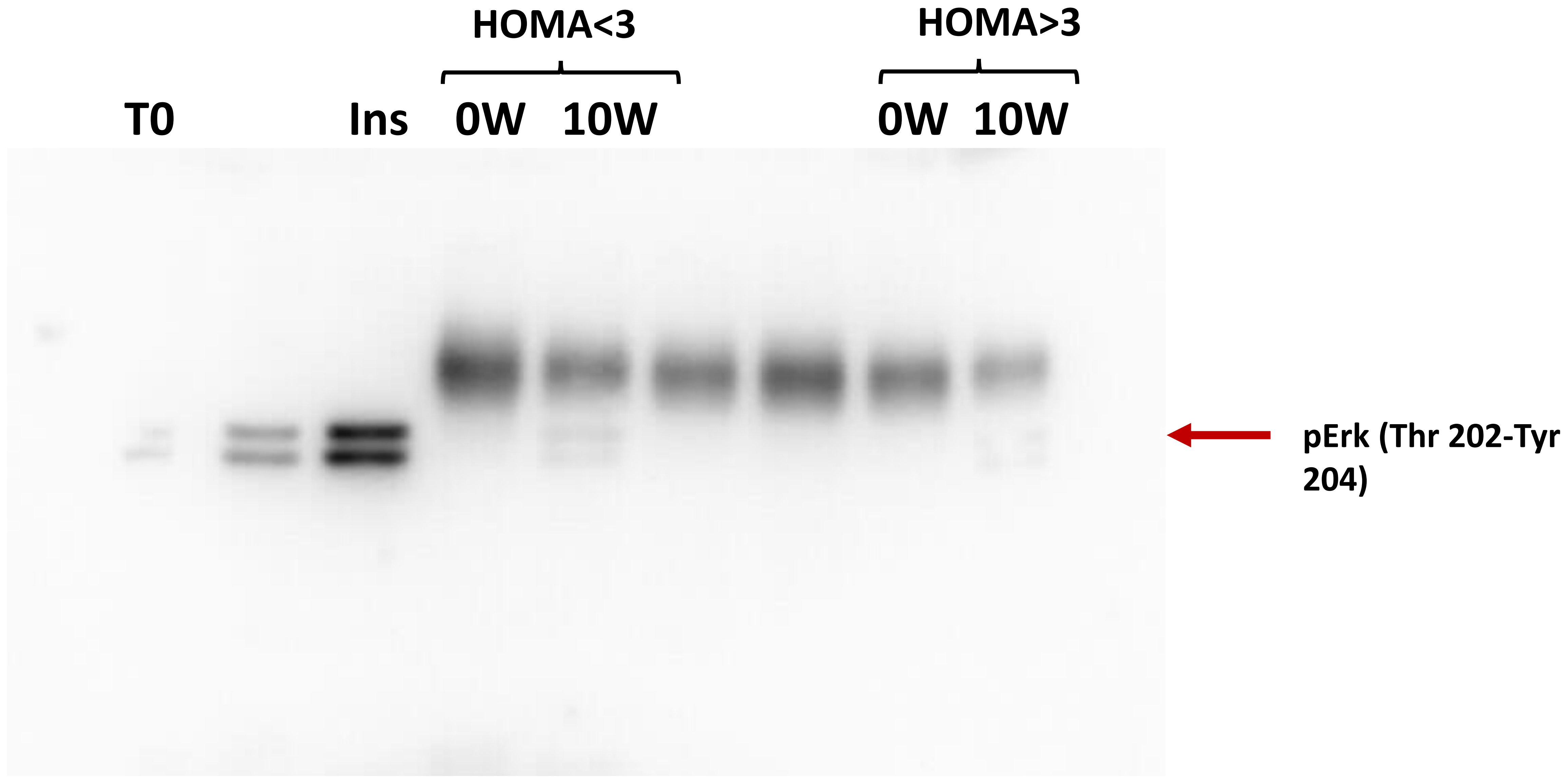

Figure 4B

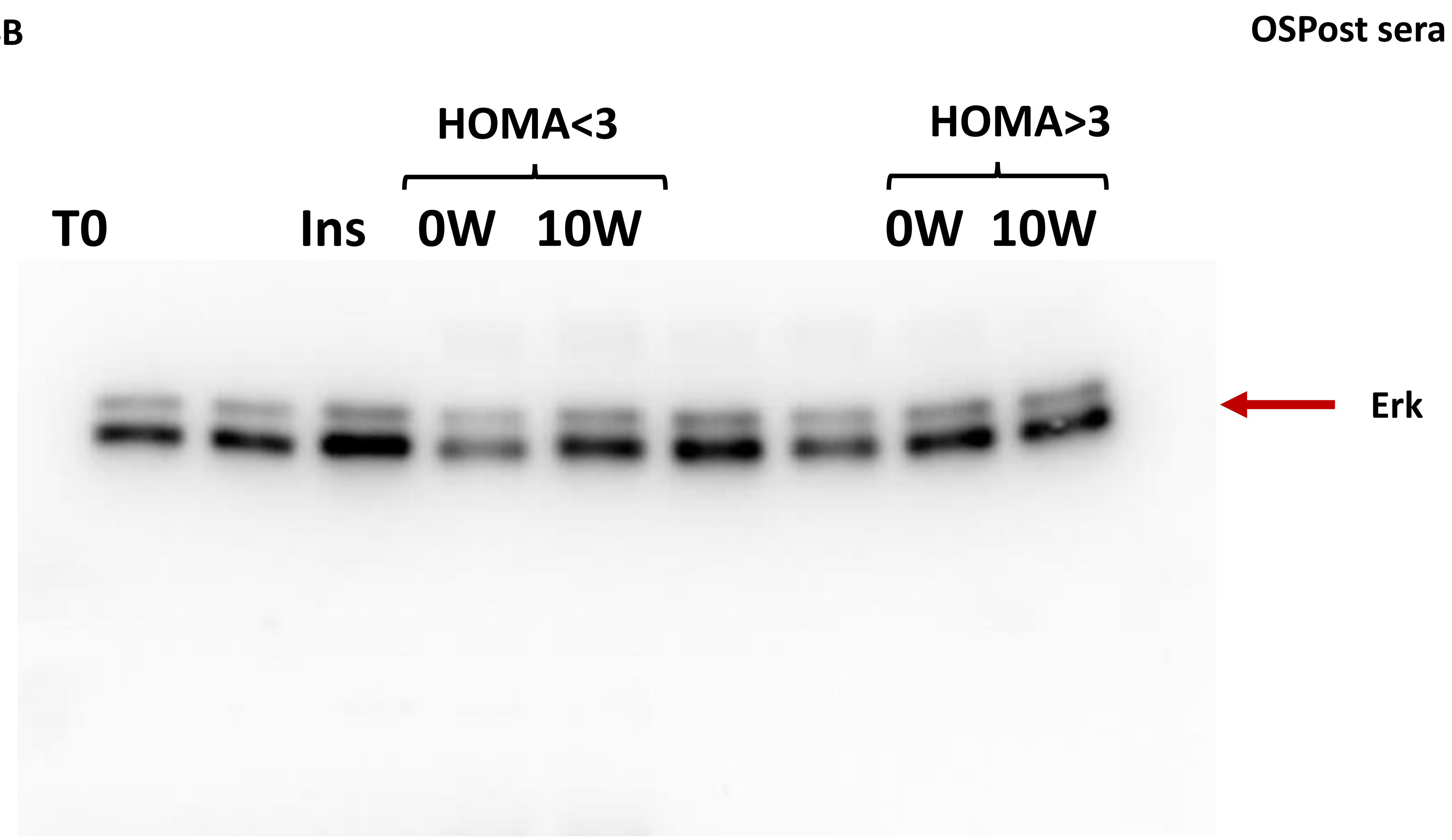

Figure 4B

OSPost sera

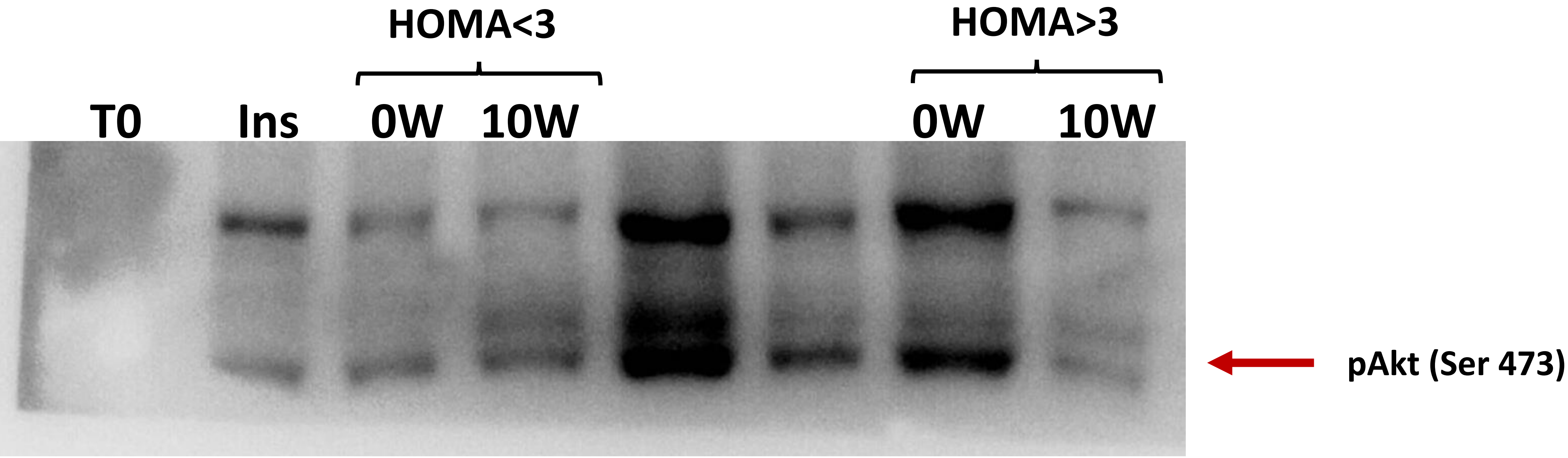

Figure 4B

OSPost sera

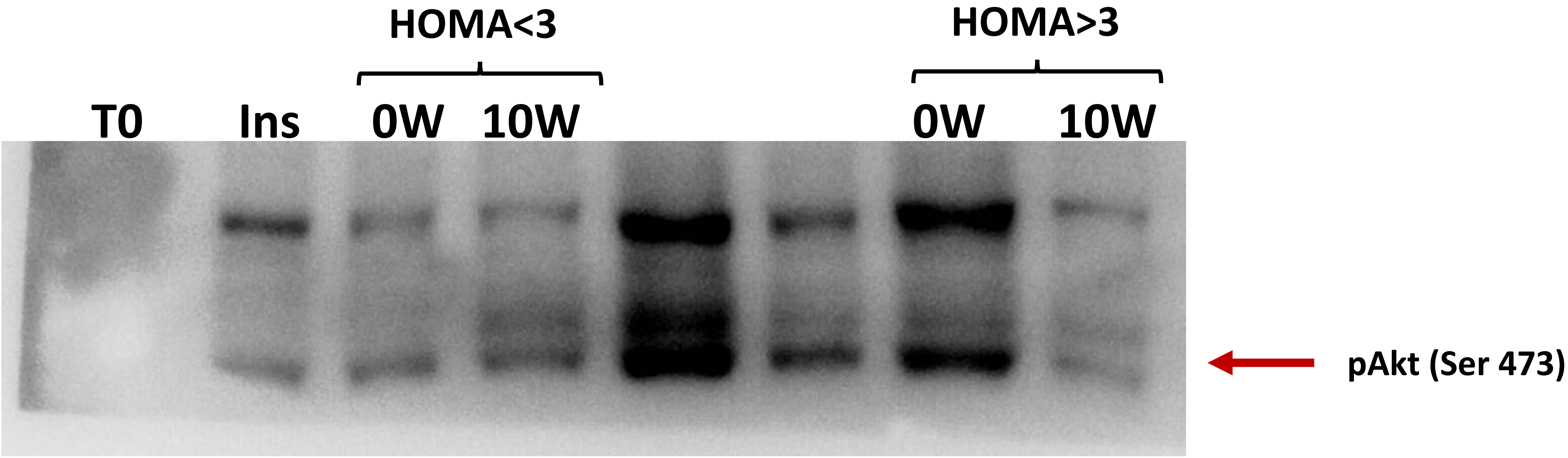

Figure 4B

OSPost sera

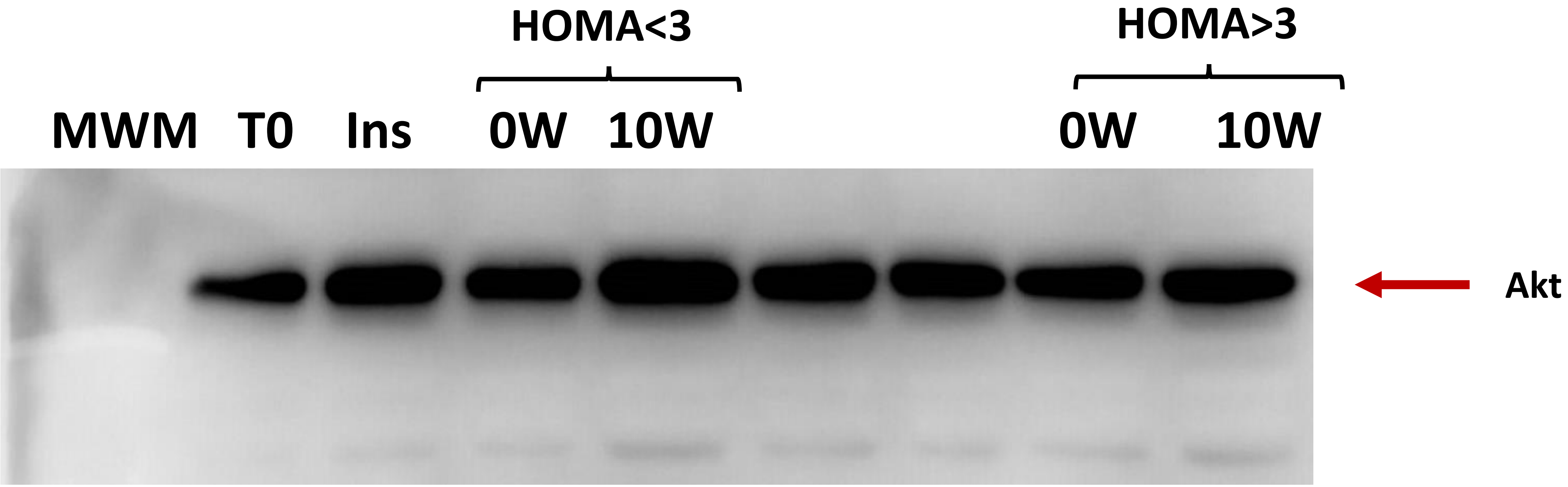

Figure 4B

OSPost sera

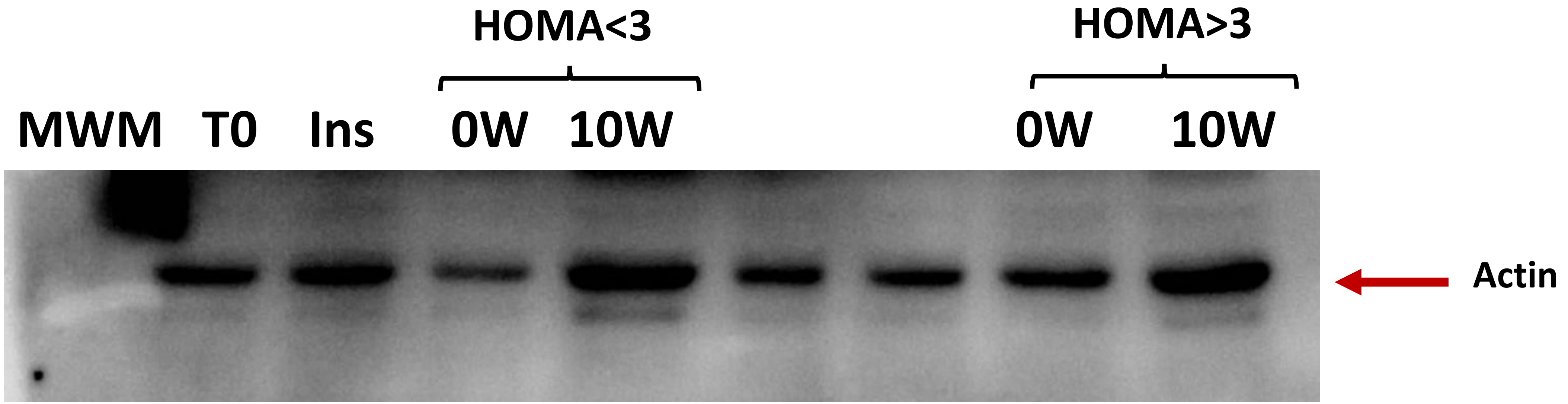

Figure 5A 10 min OSPost sera

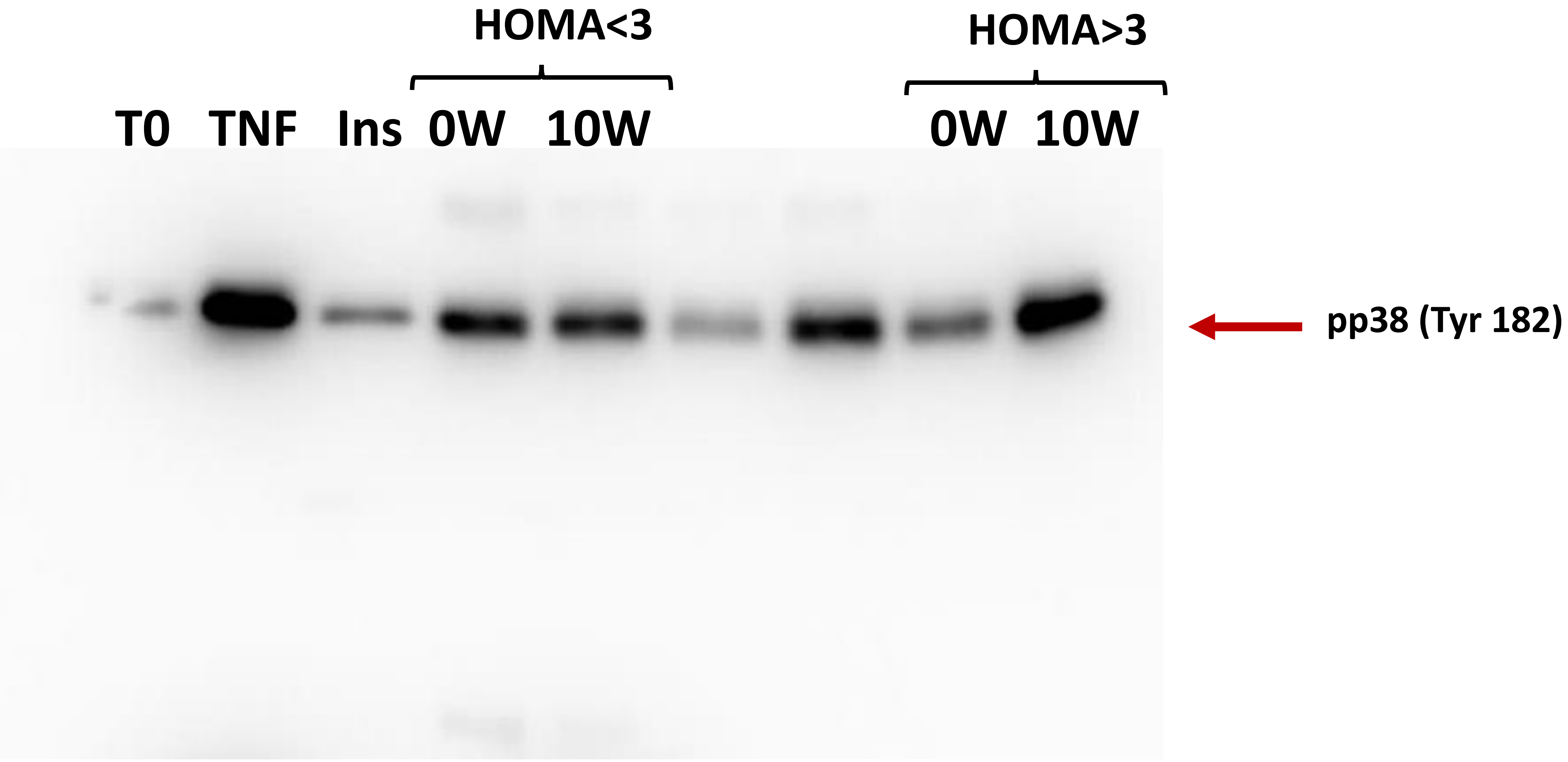

Figure 5A 10 min OSPost sera

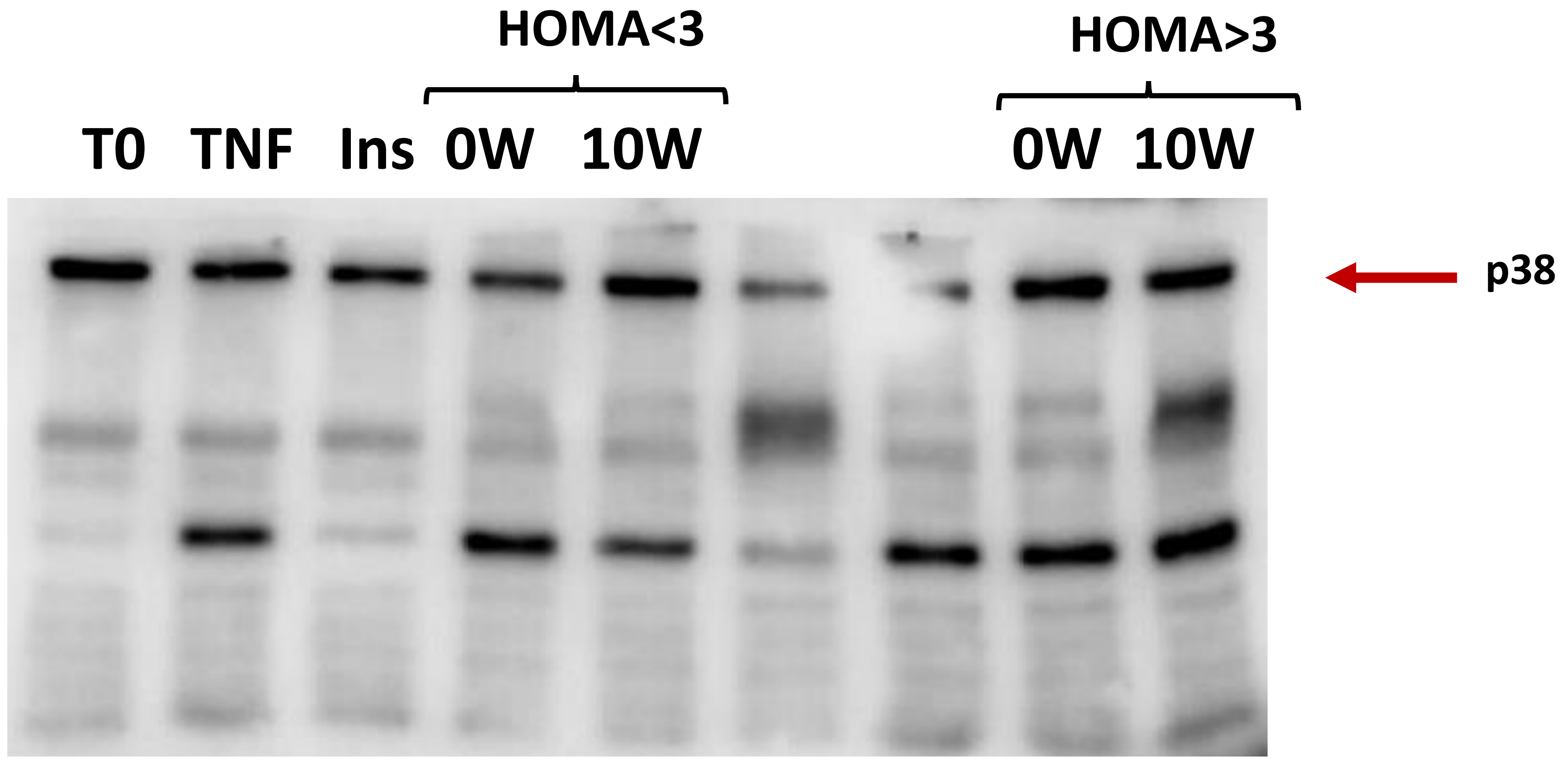

Figure 5A 10 min OSPost sera

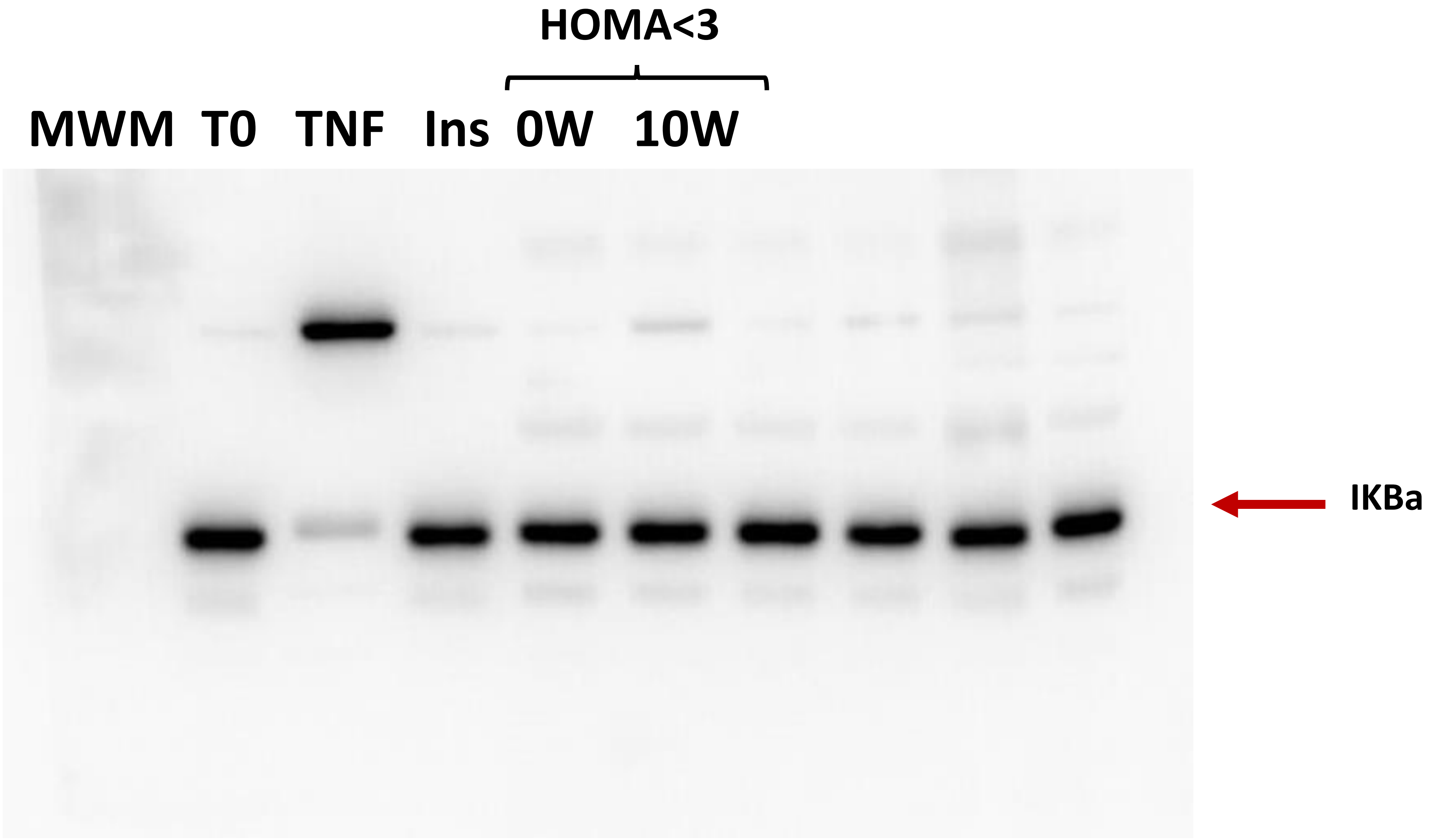

Figure 5A 10 min OSPost sera

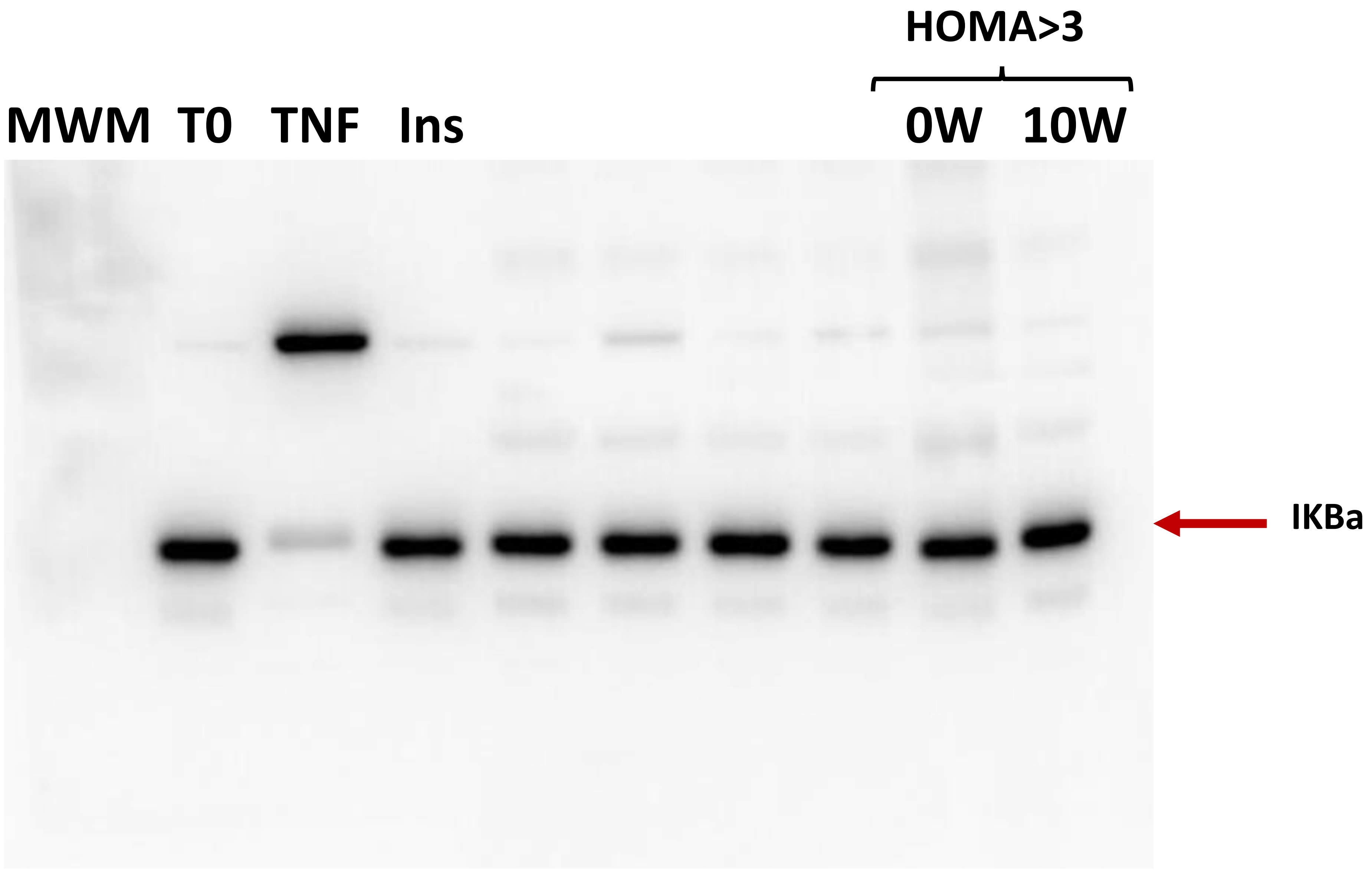

Figure 5A 10 min OSPost sera

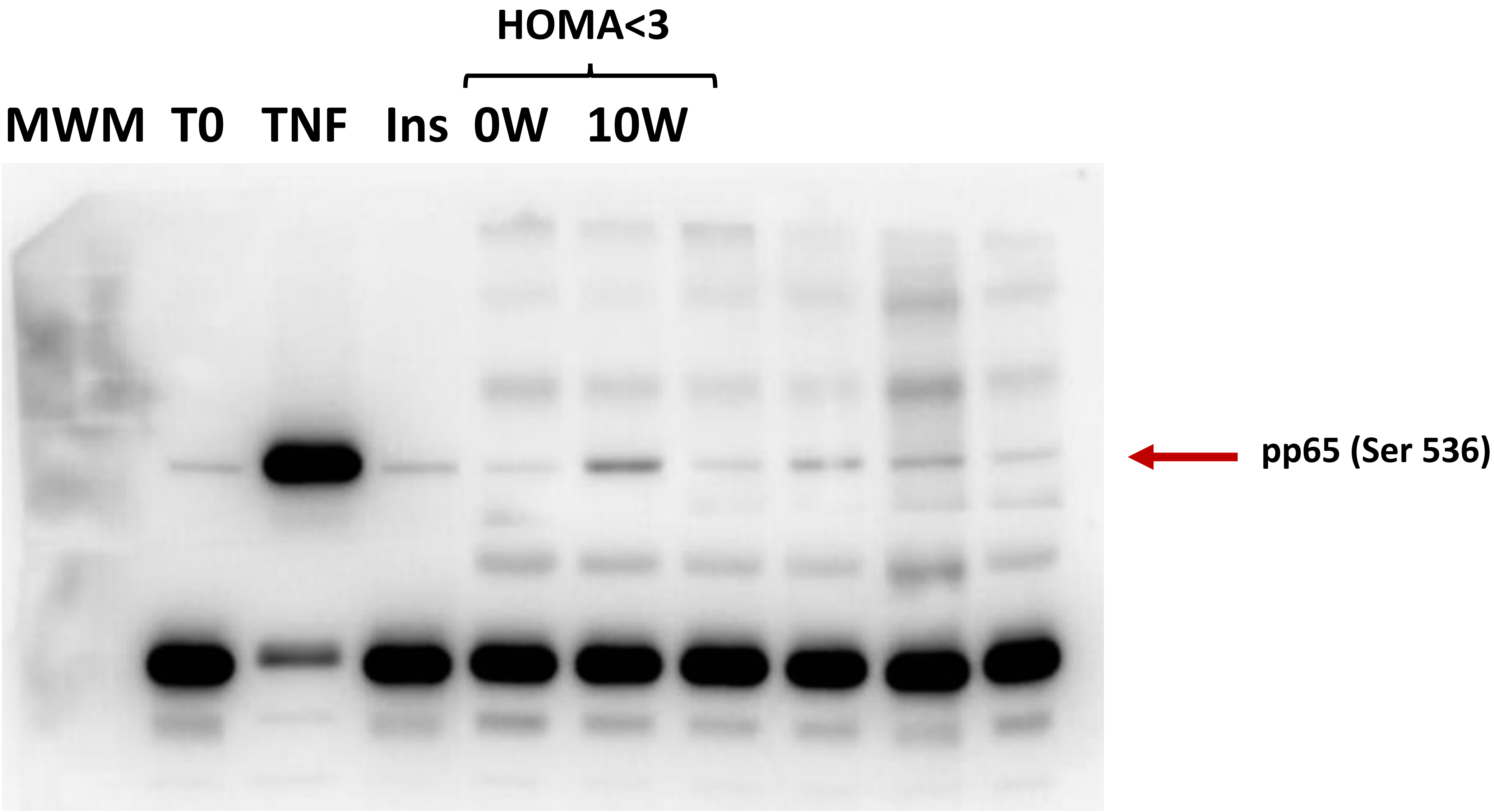

Figure 5A 10 min OSPost sera

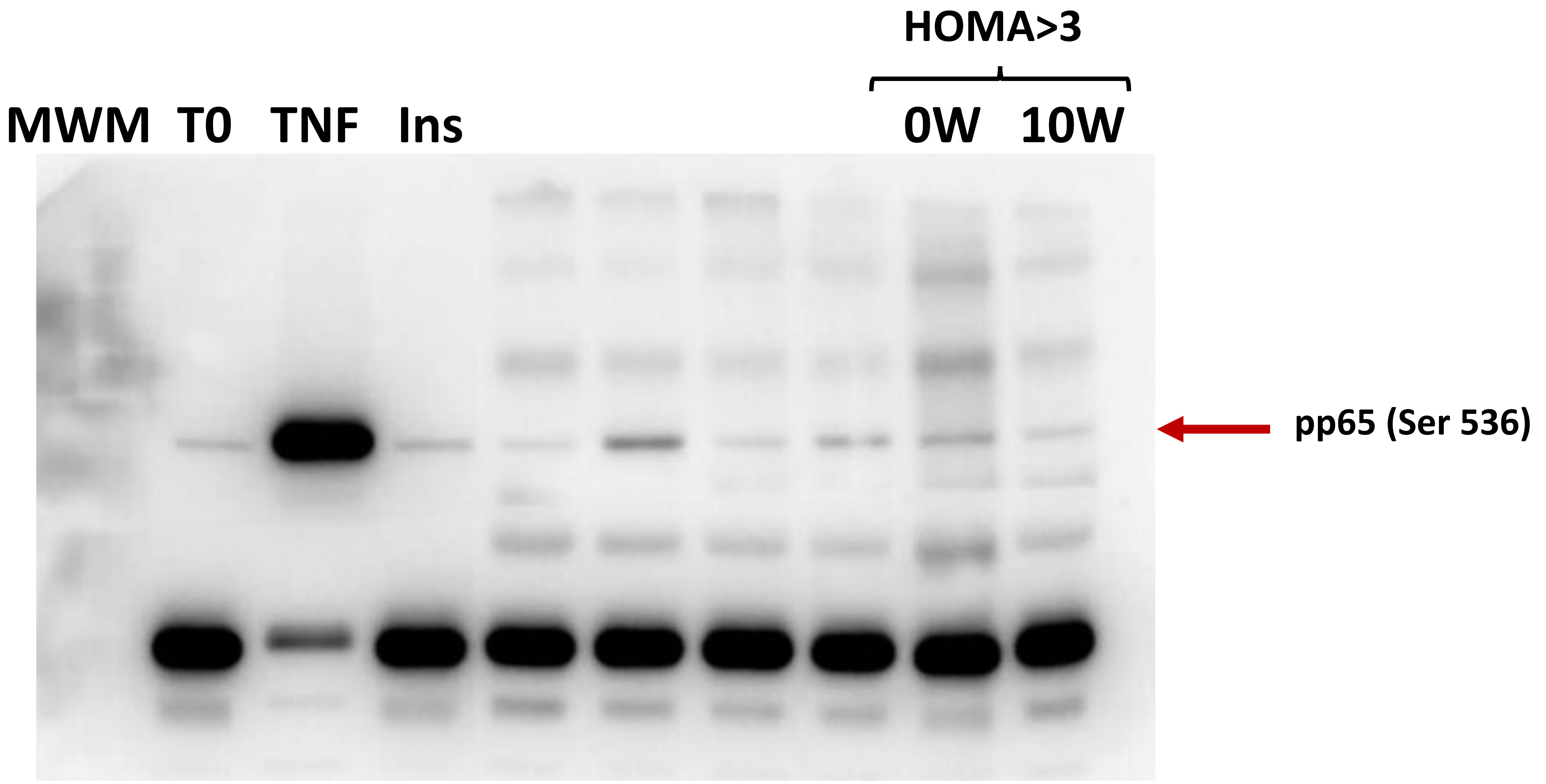

Figure 5A 10 min OSPost sera

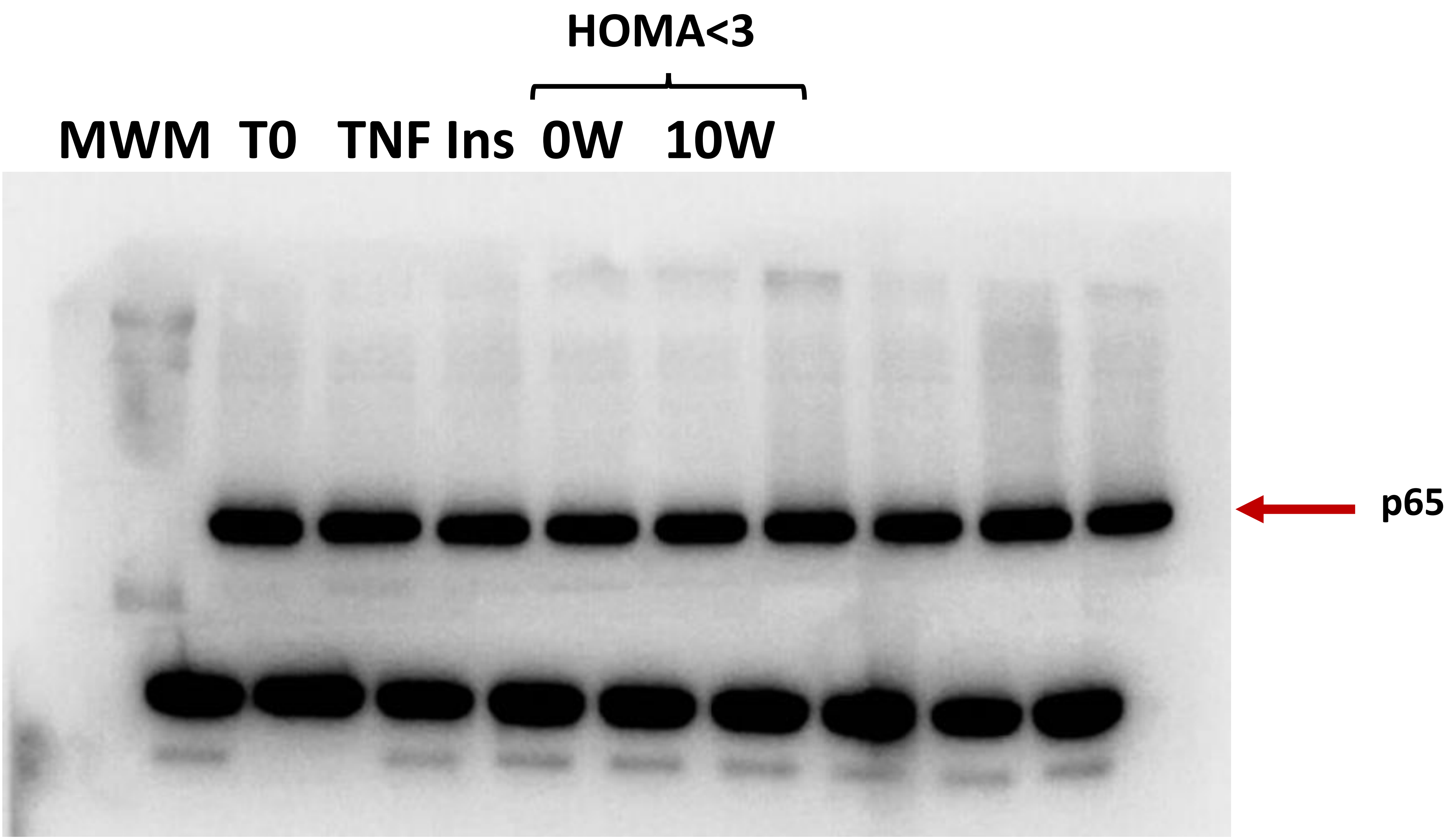

Figure 5A 10 min OSPost sera

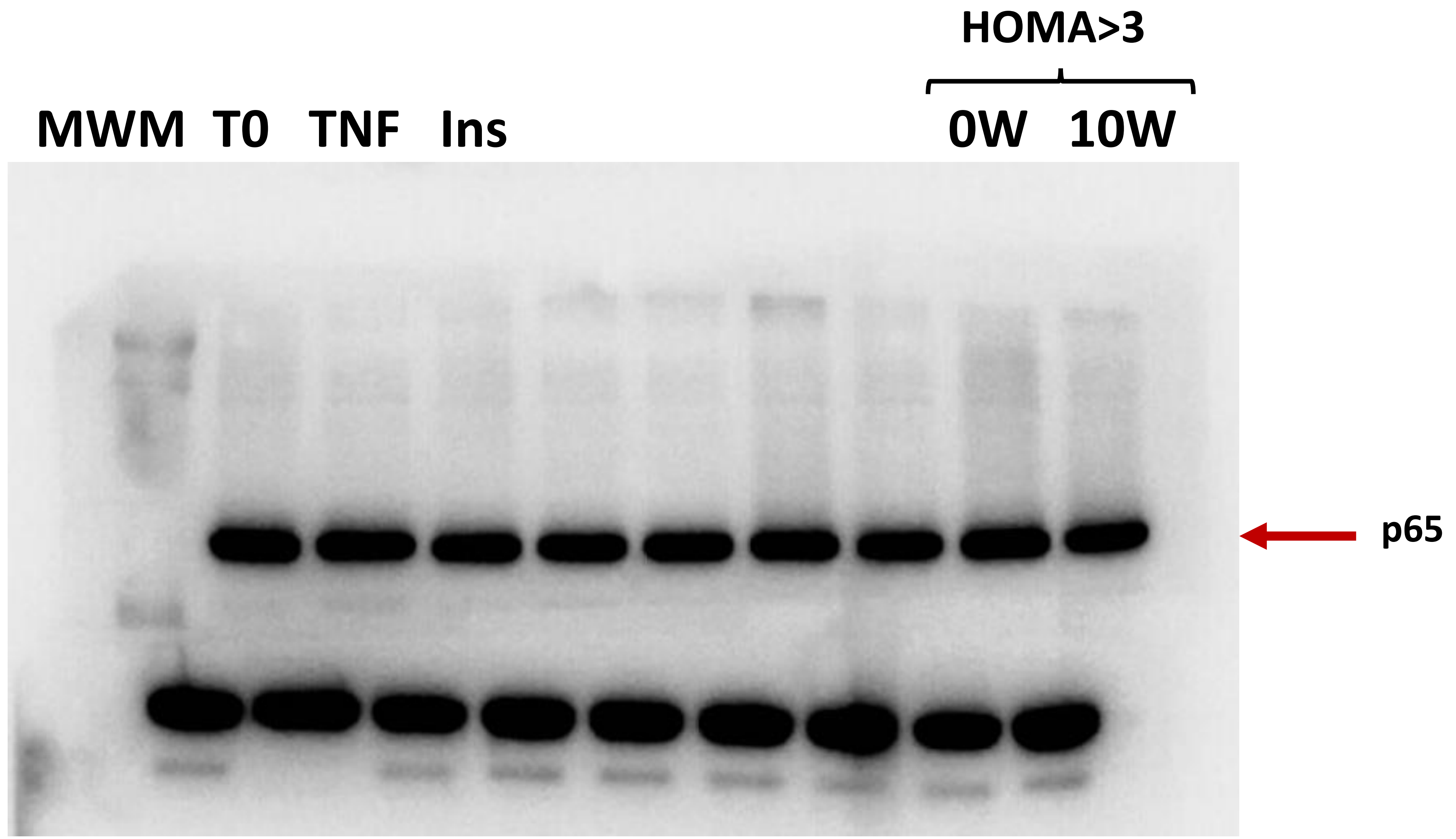

Figure 5A 10 min OSPost sera

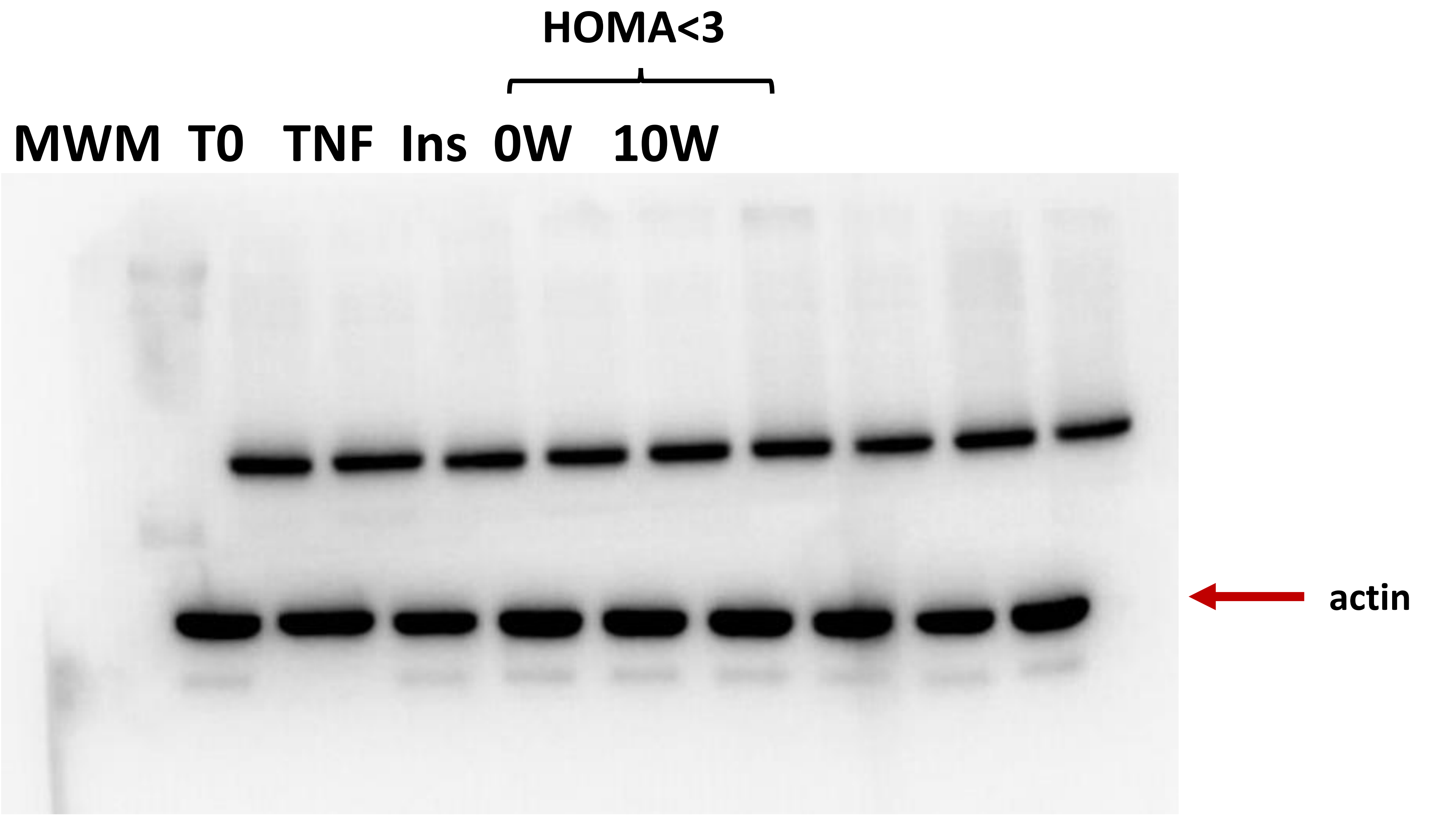

Figure 5A 10 min OSPost sera

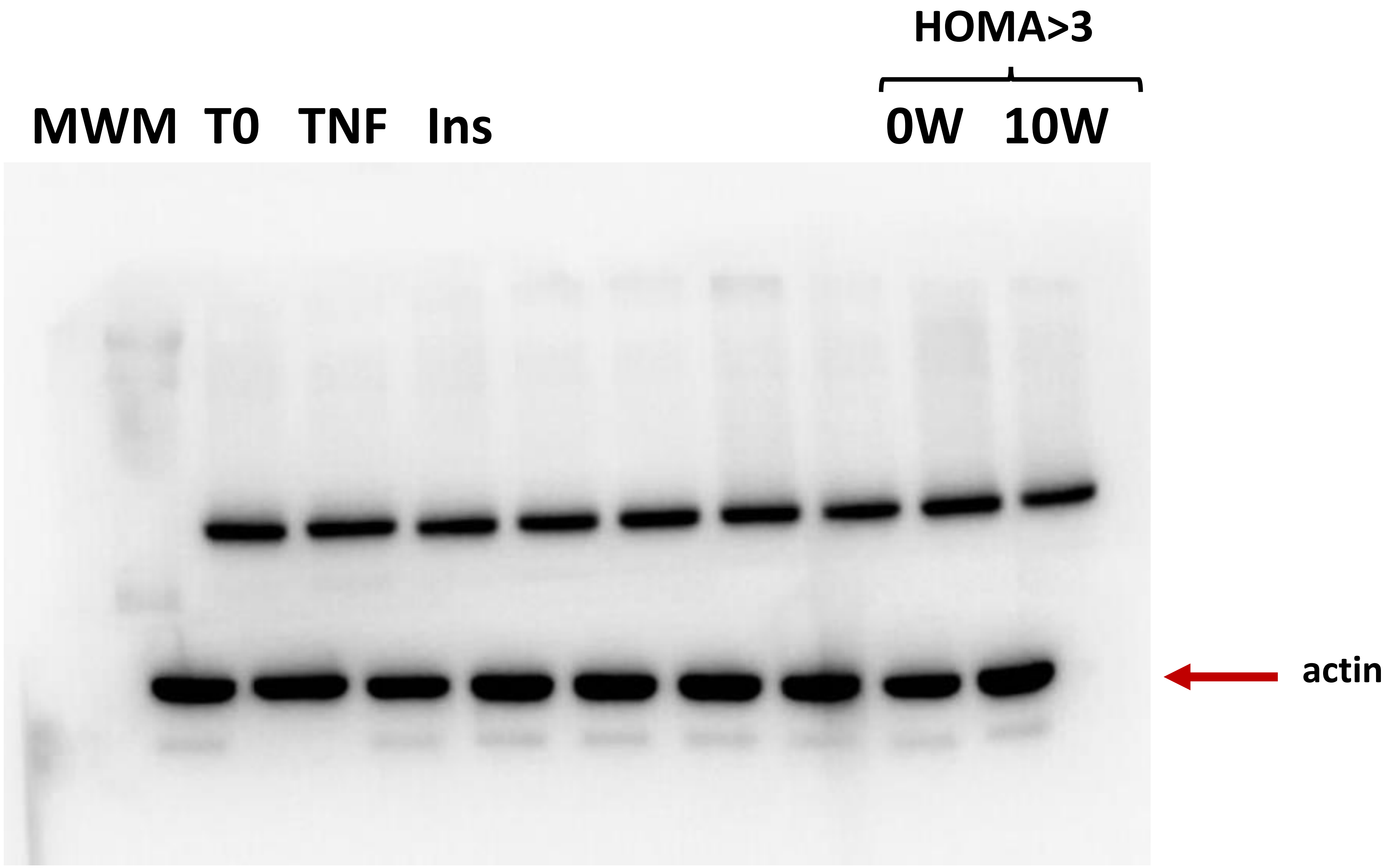

Figure 5A 10 min OSPost sera

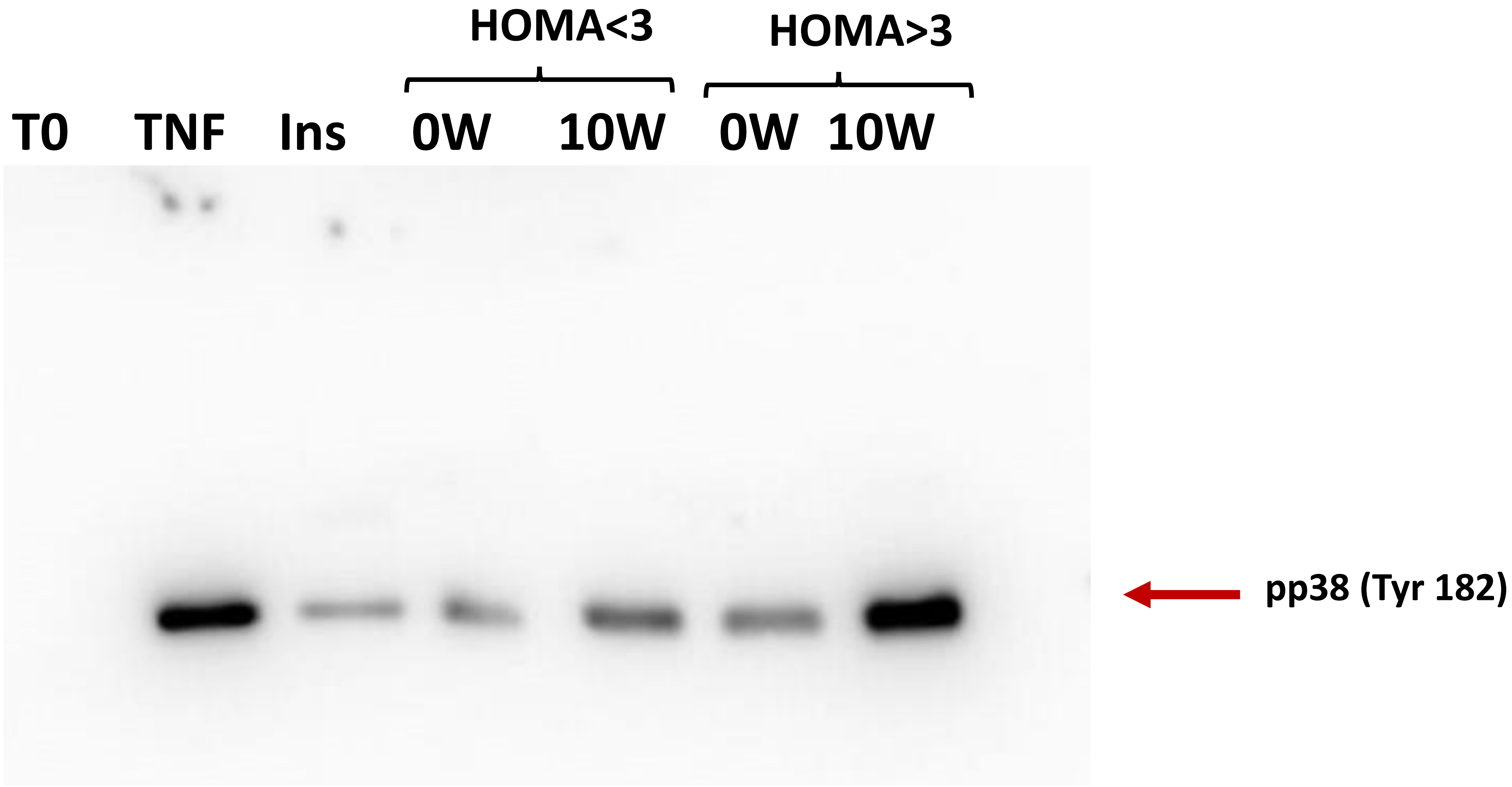

Figure 5A 10 min OSPost sera

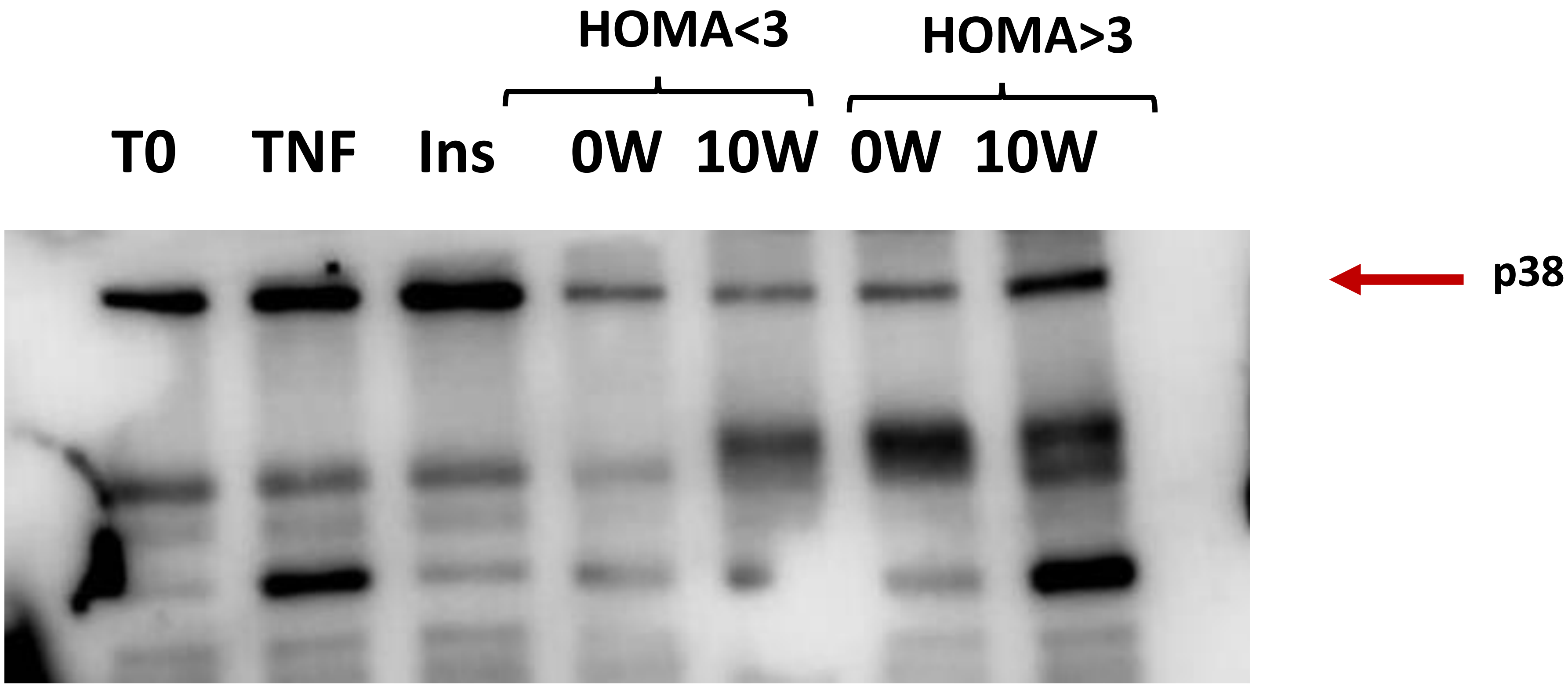

Figure 5A **30 min** **OSPost sera**

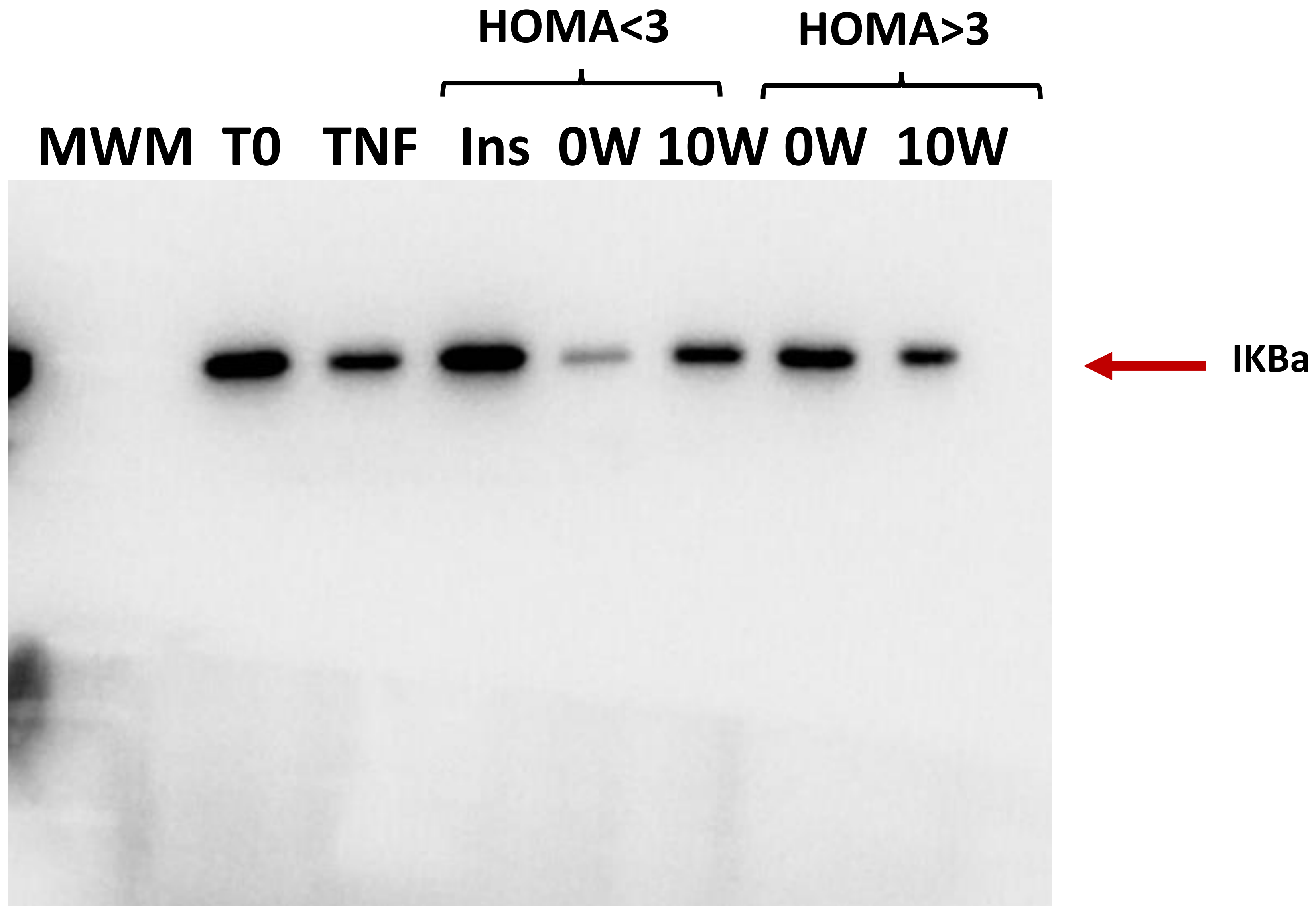

Figure 5A 30 min OSPost sera

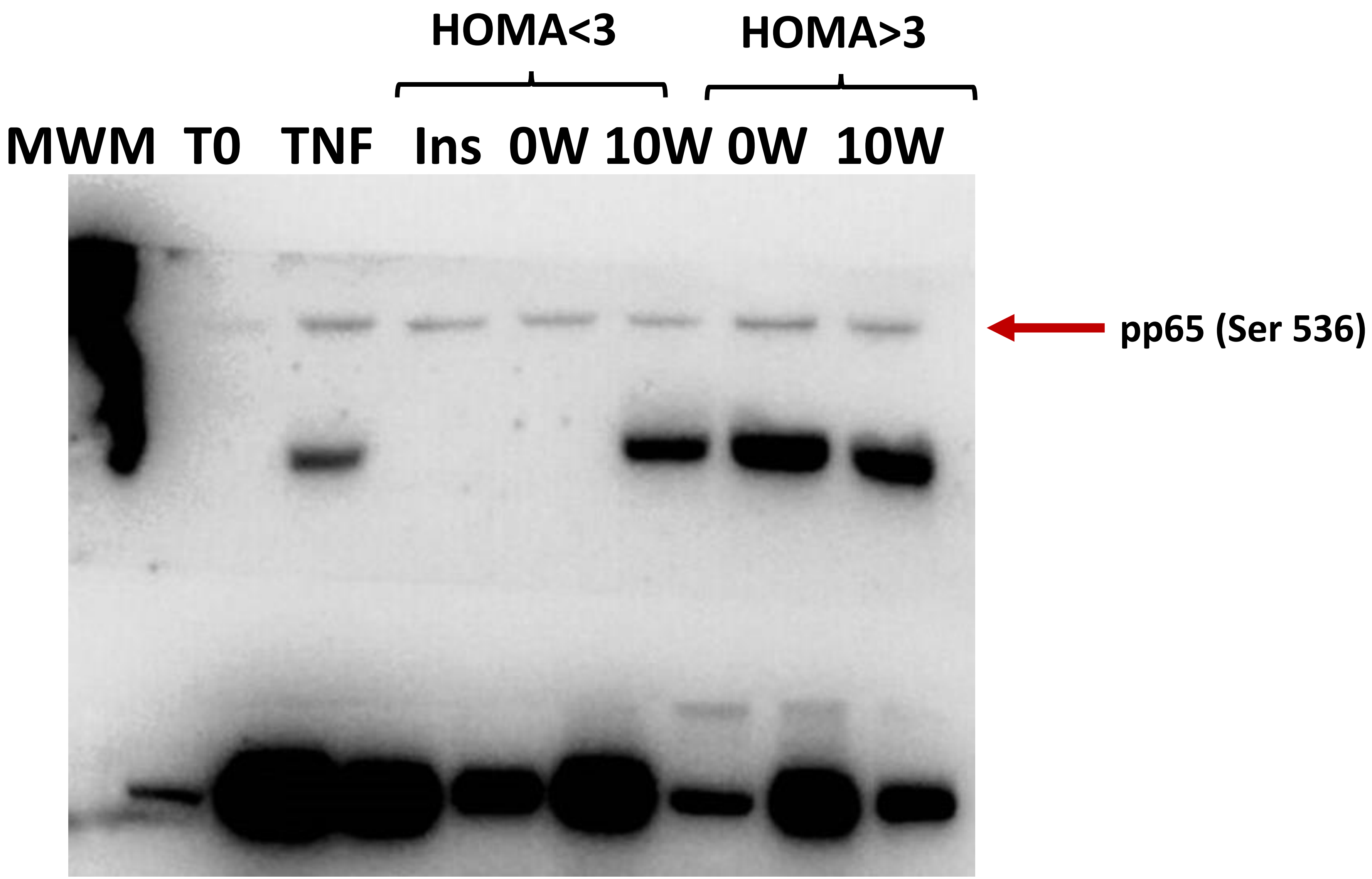

Figure 5A

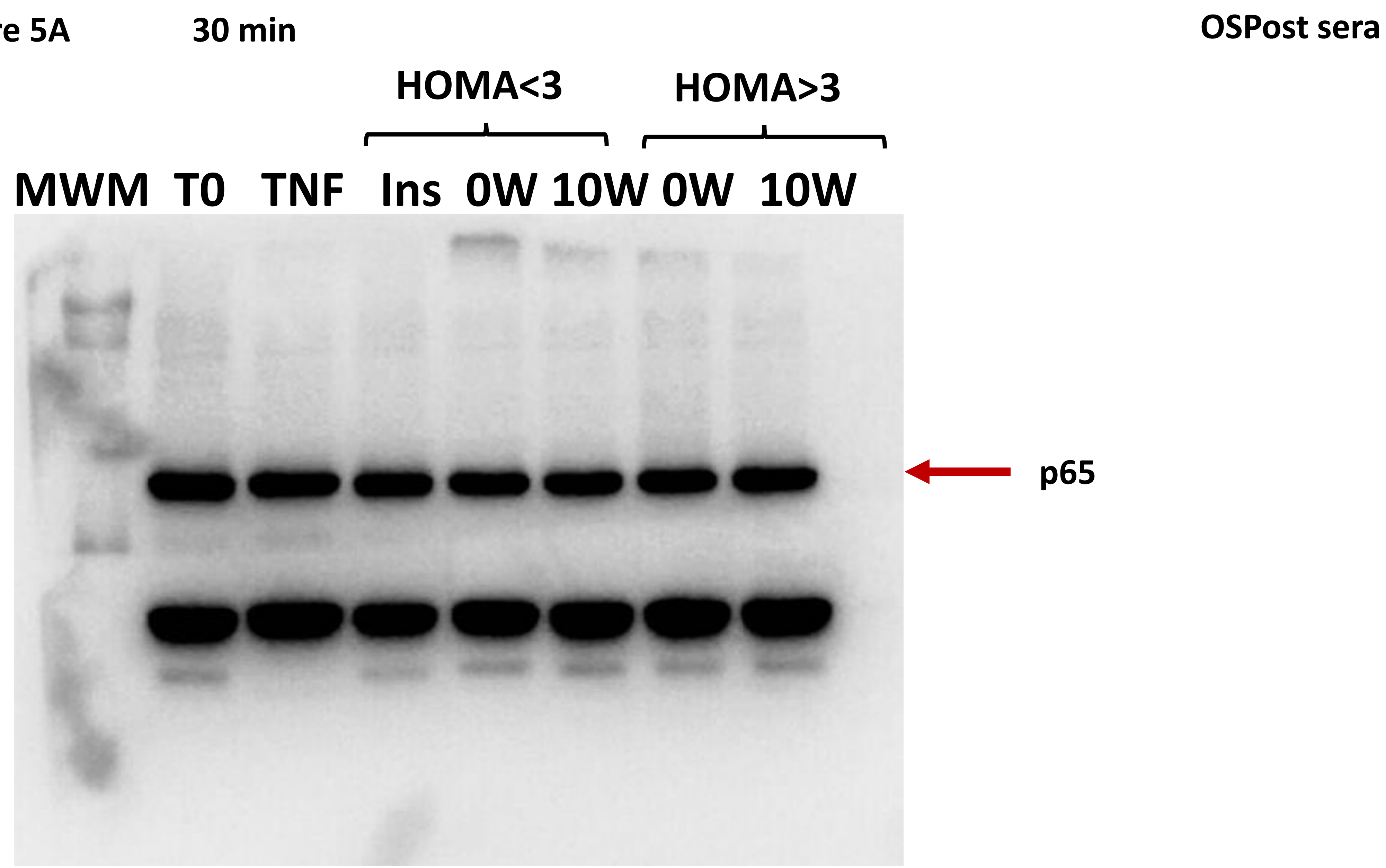

Figure 5A 30 min OSPost sera

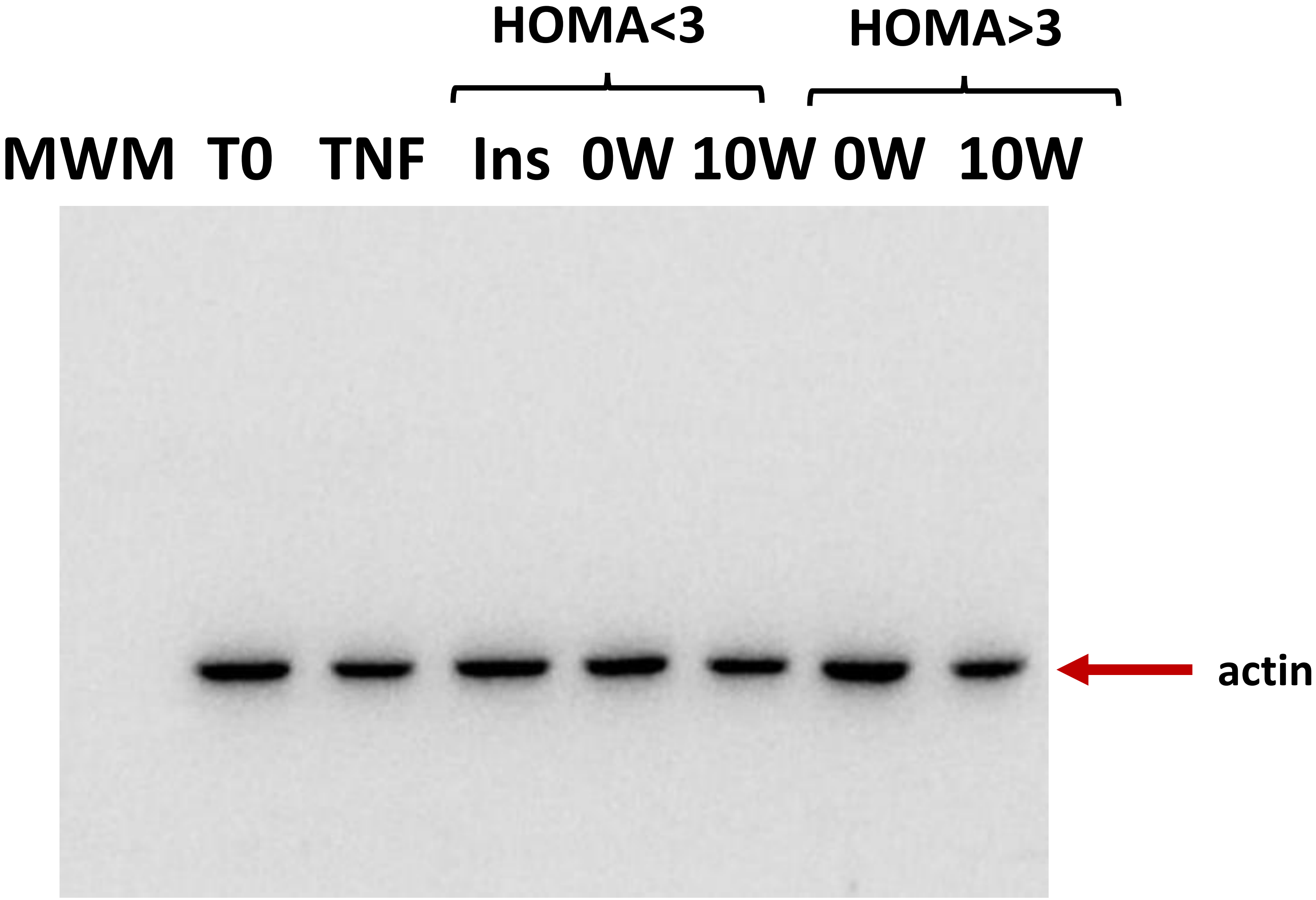

Supplementary Figure 3A

MCF-10A pIR

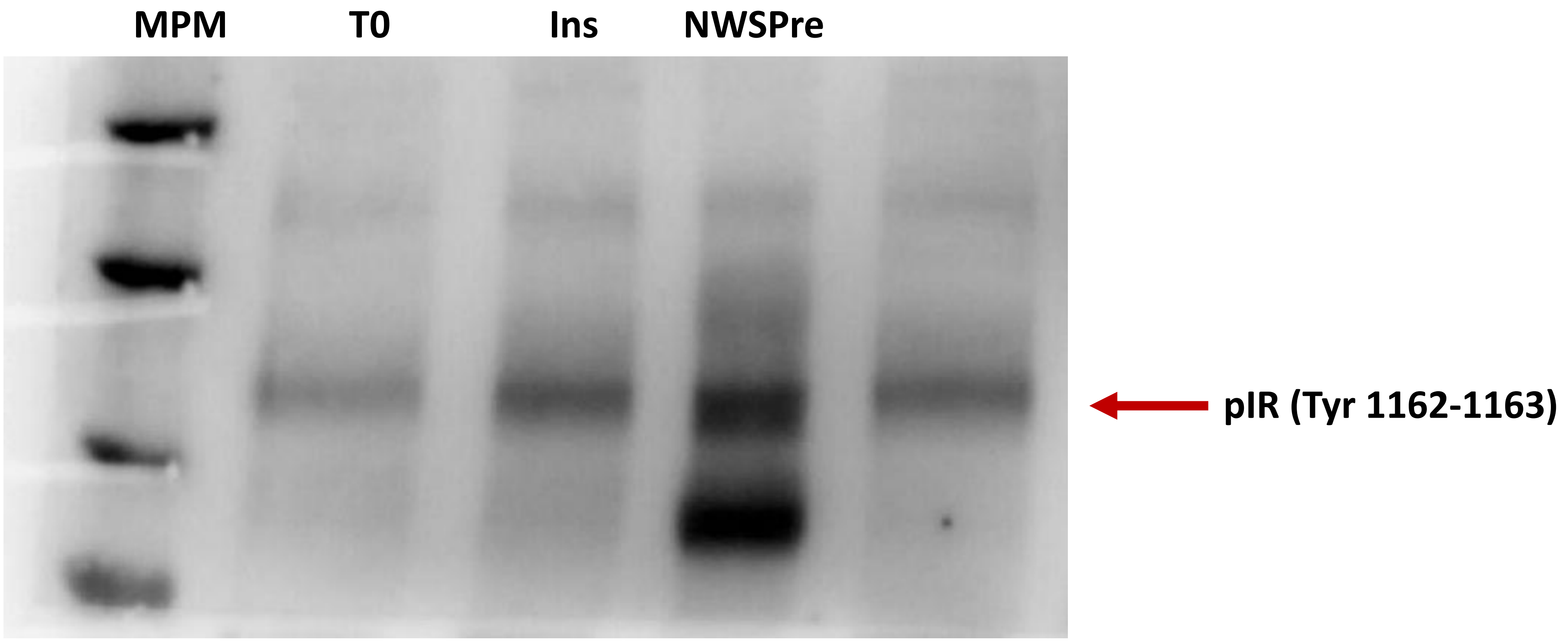

Supplementary Figure 3A

MCF-10A IR

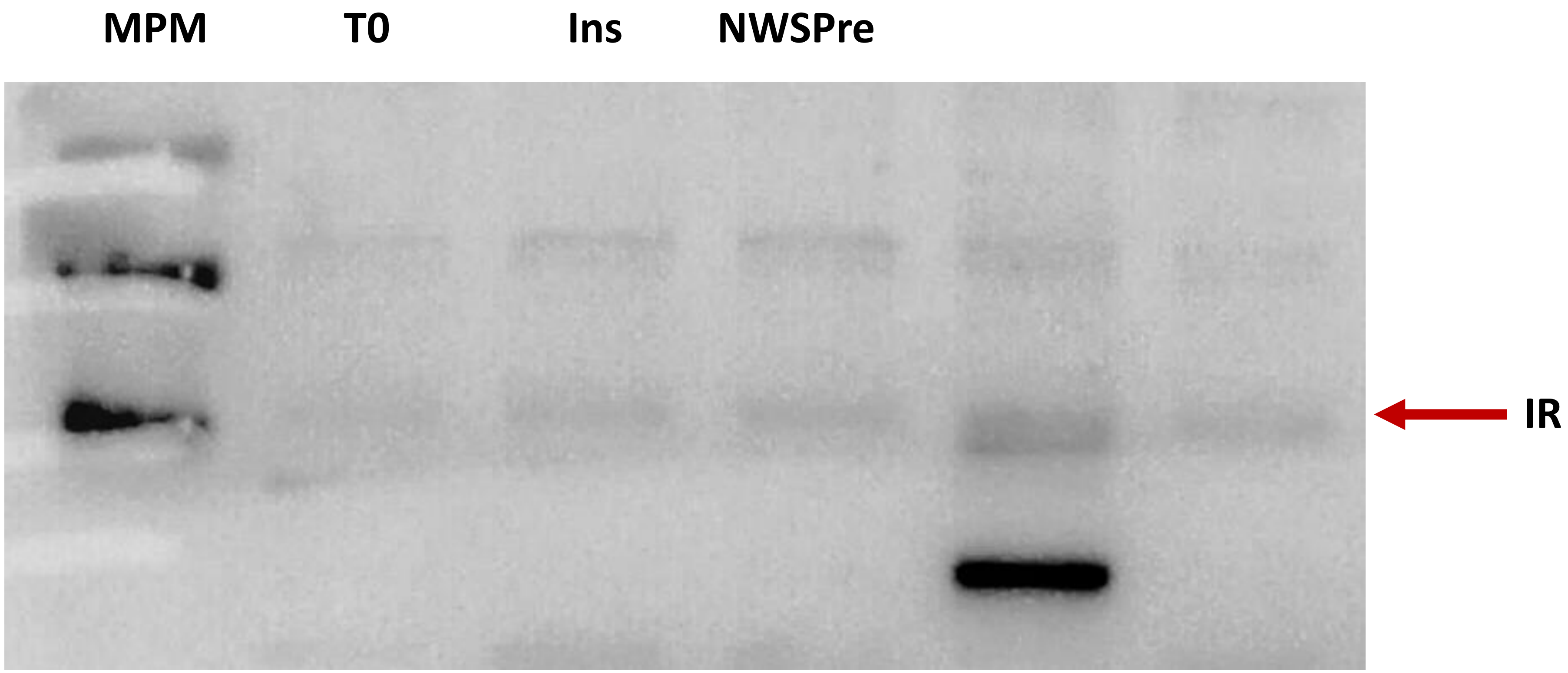

Supplementary Figure 3A

MCF-10A pAkt(Ser 473)

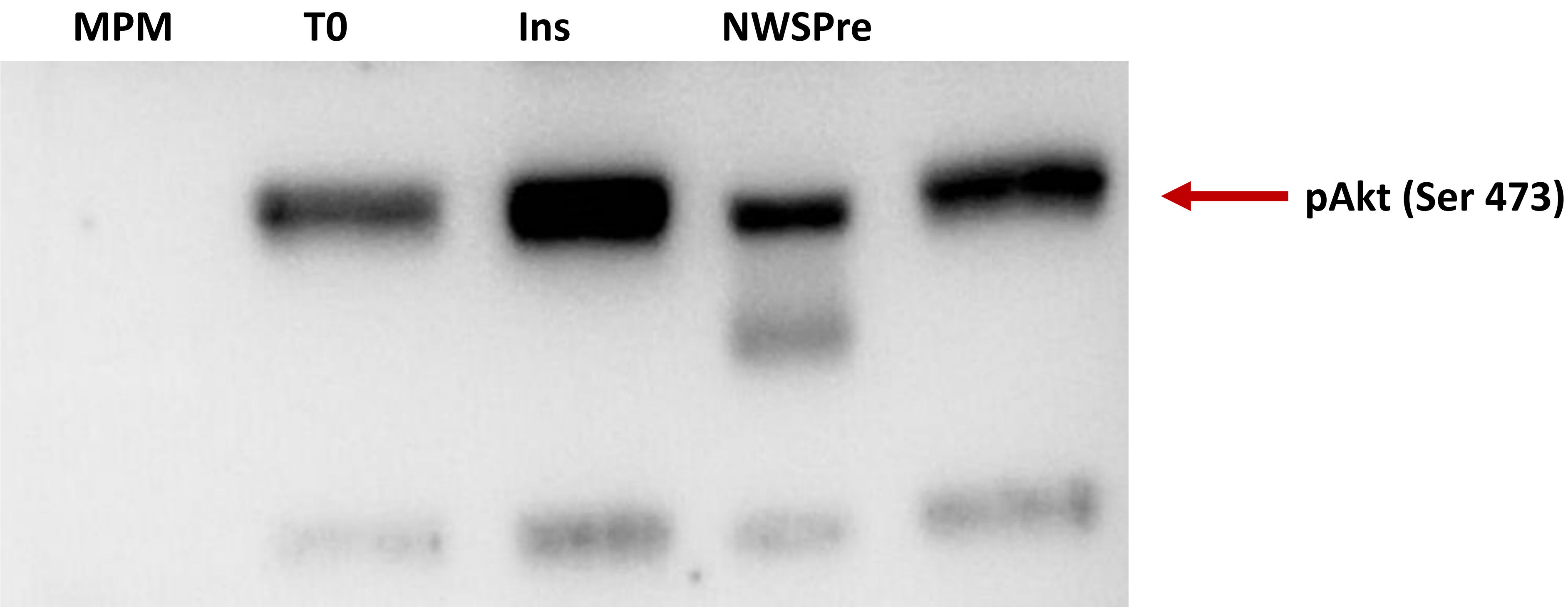

Supplementary Figure 3A

MCF-10A Akt

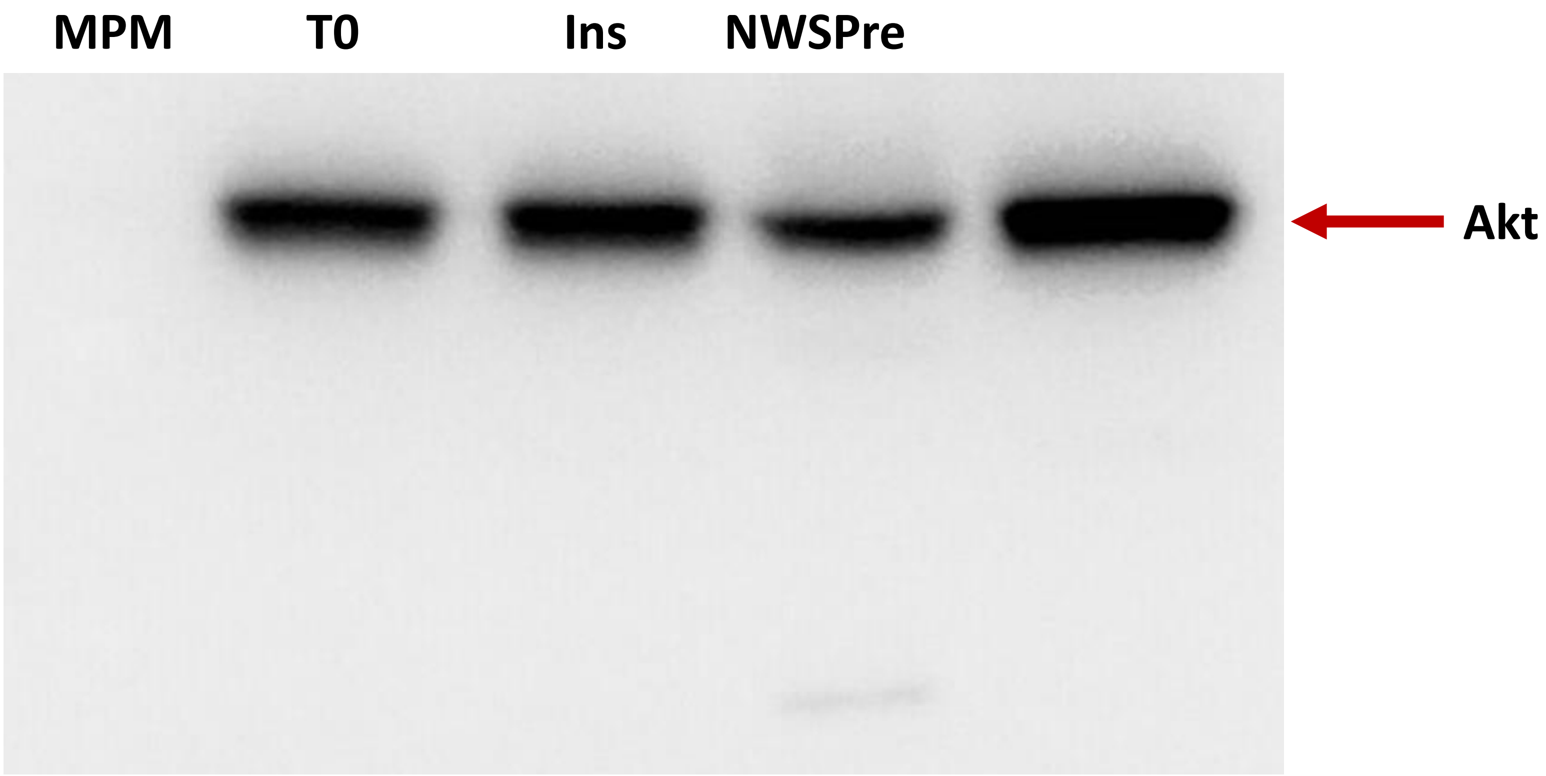

Supplementary Figure 3A

MCF-10A pp70S6K(Thr 389)

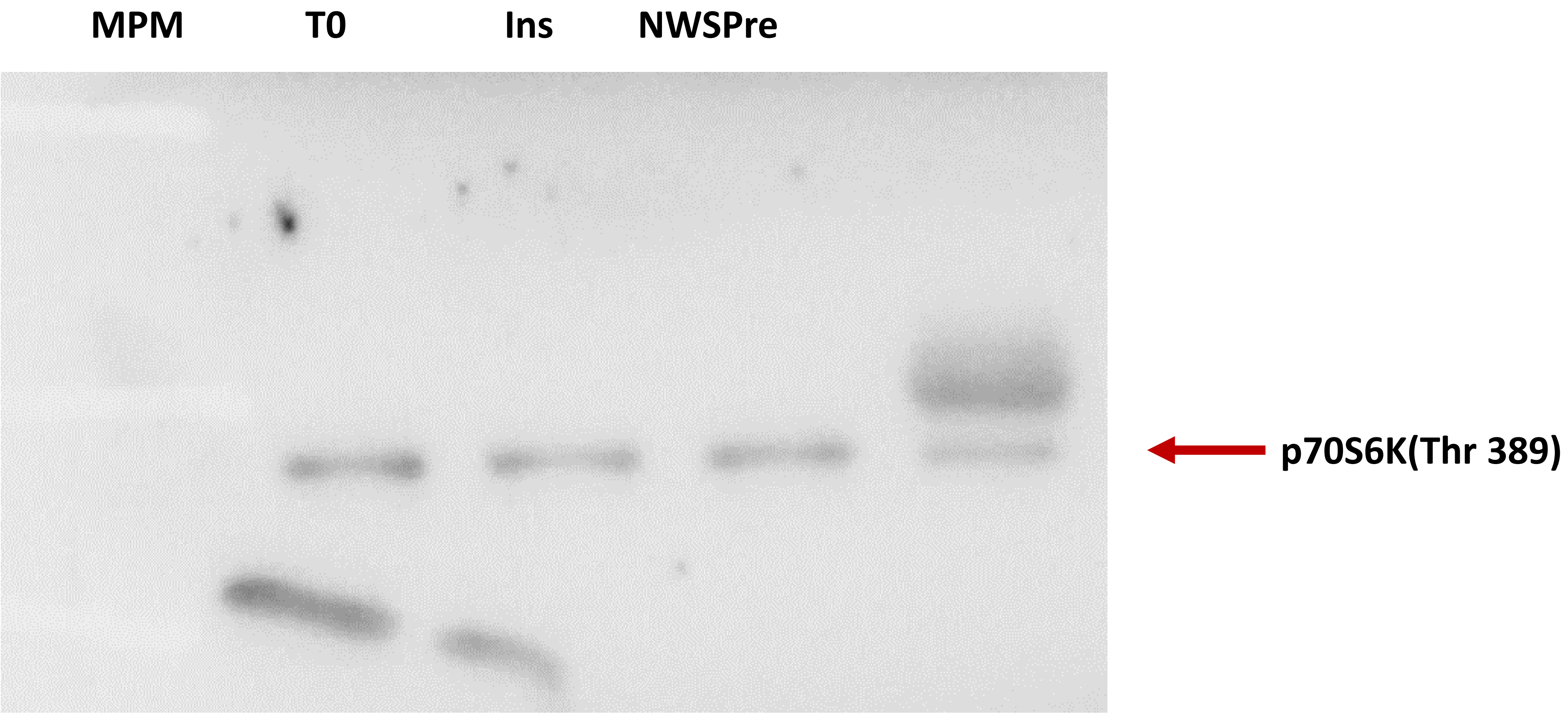

Supplementary Figure 3A

MCF-10A p70S6K

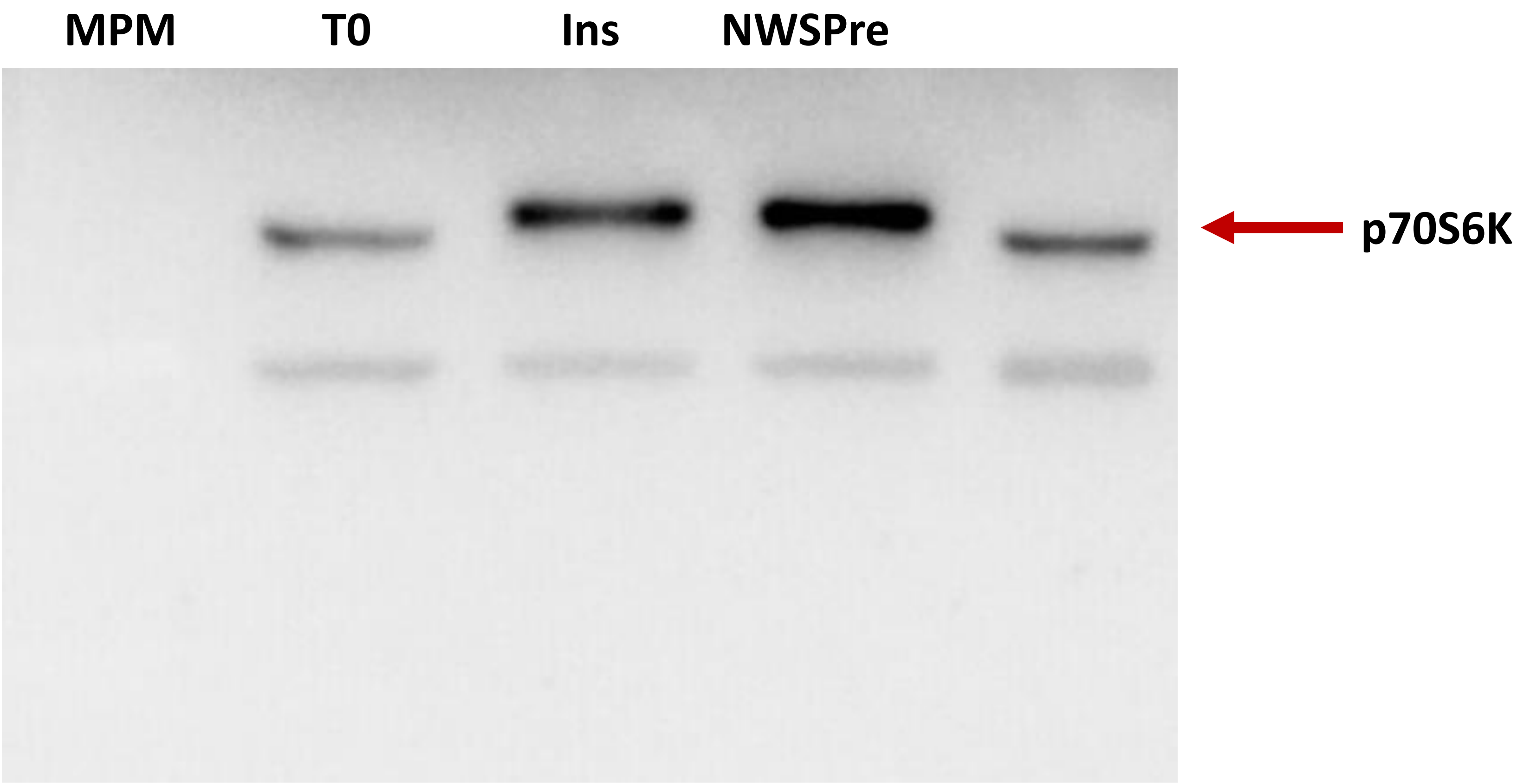

Supplementary Figure 3A

MCF-10A actin

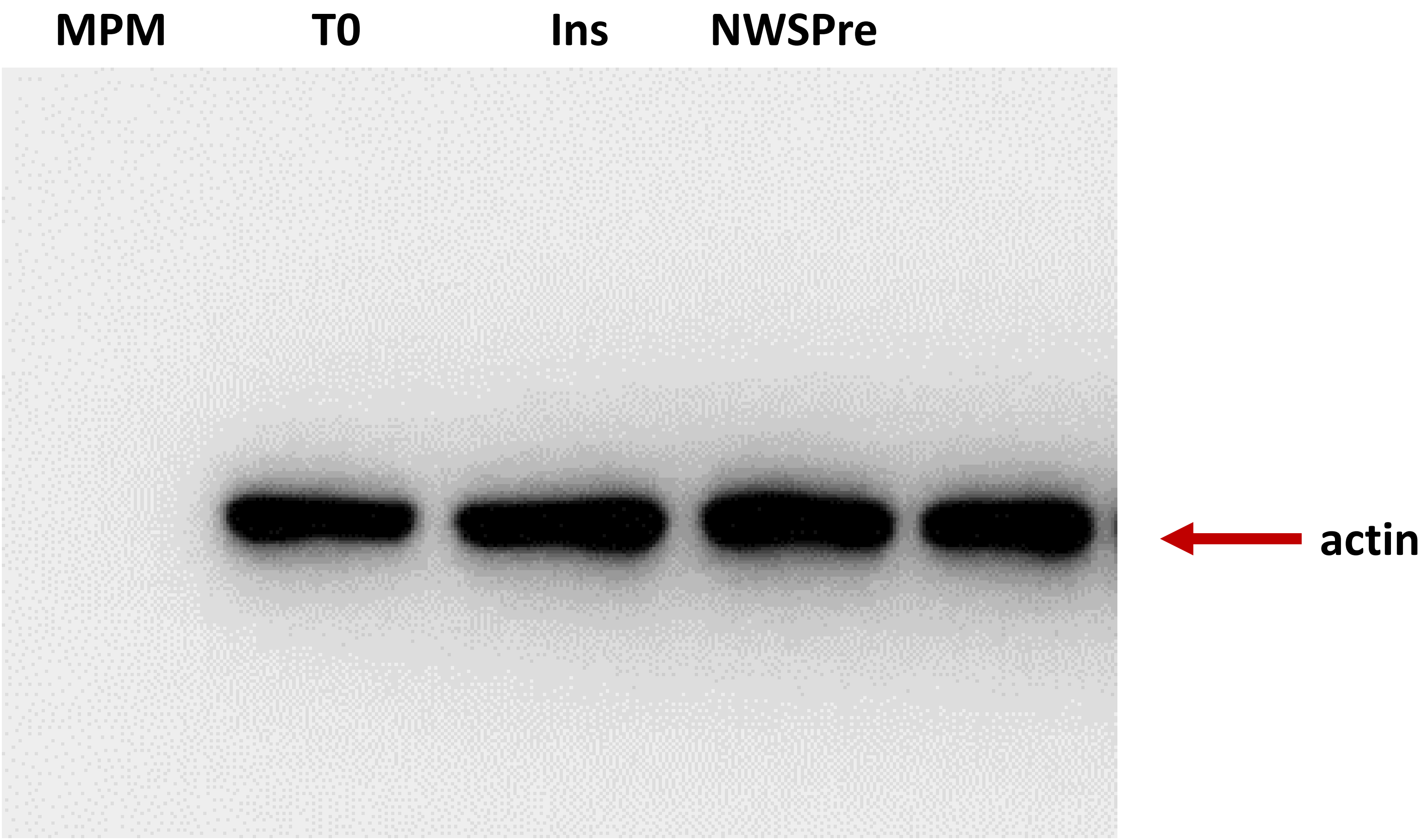

Supplementary Figure 3B

MCF-7 pIR

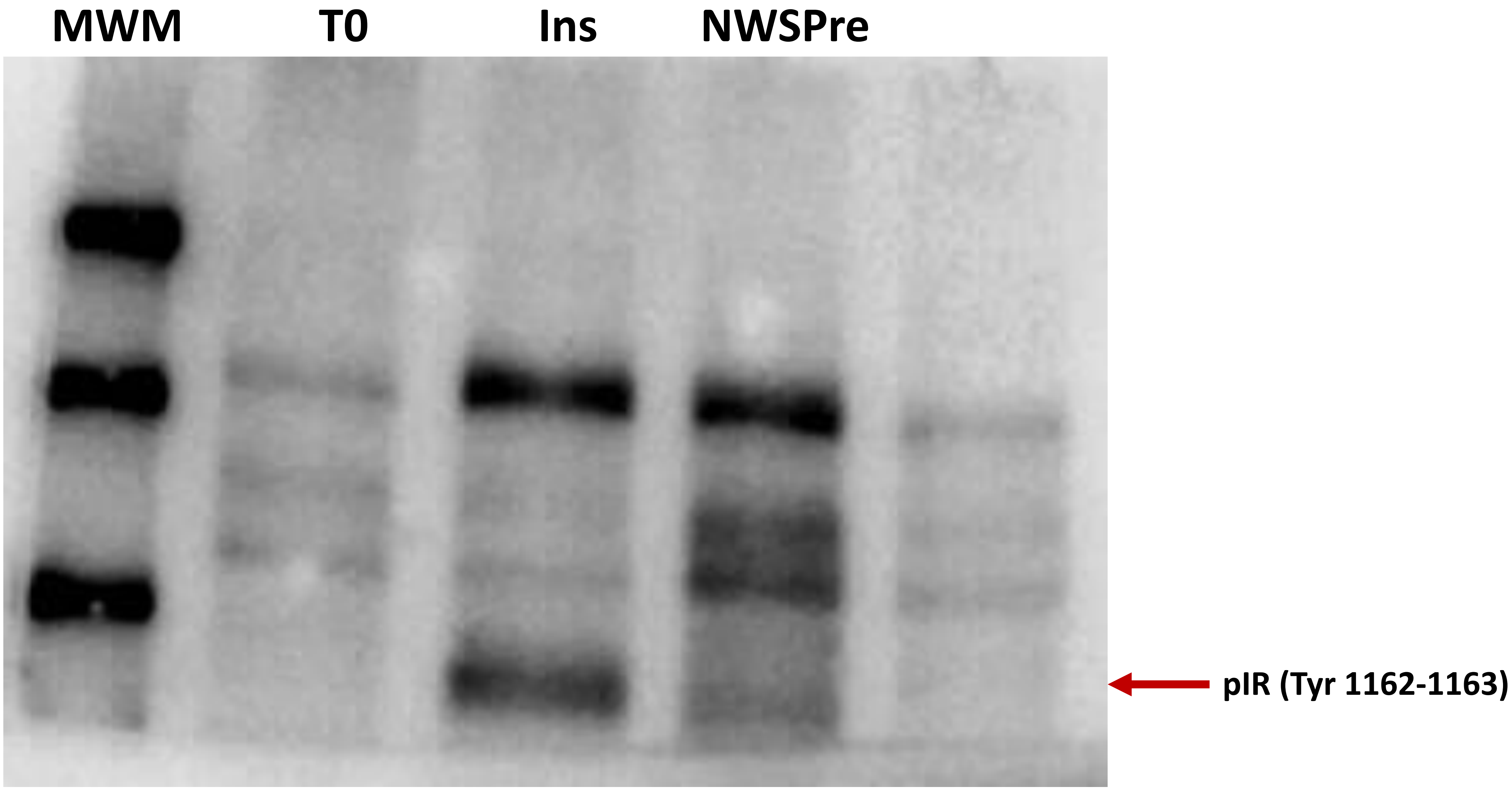

Supplementary Figure 3B

MCF-7 IR

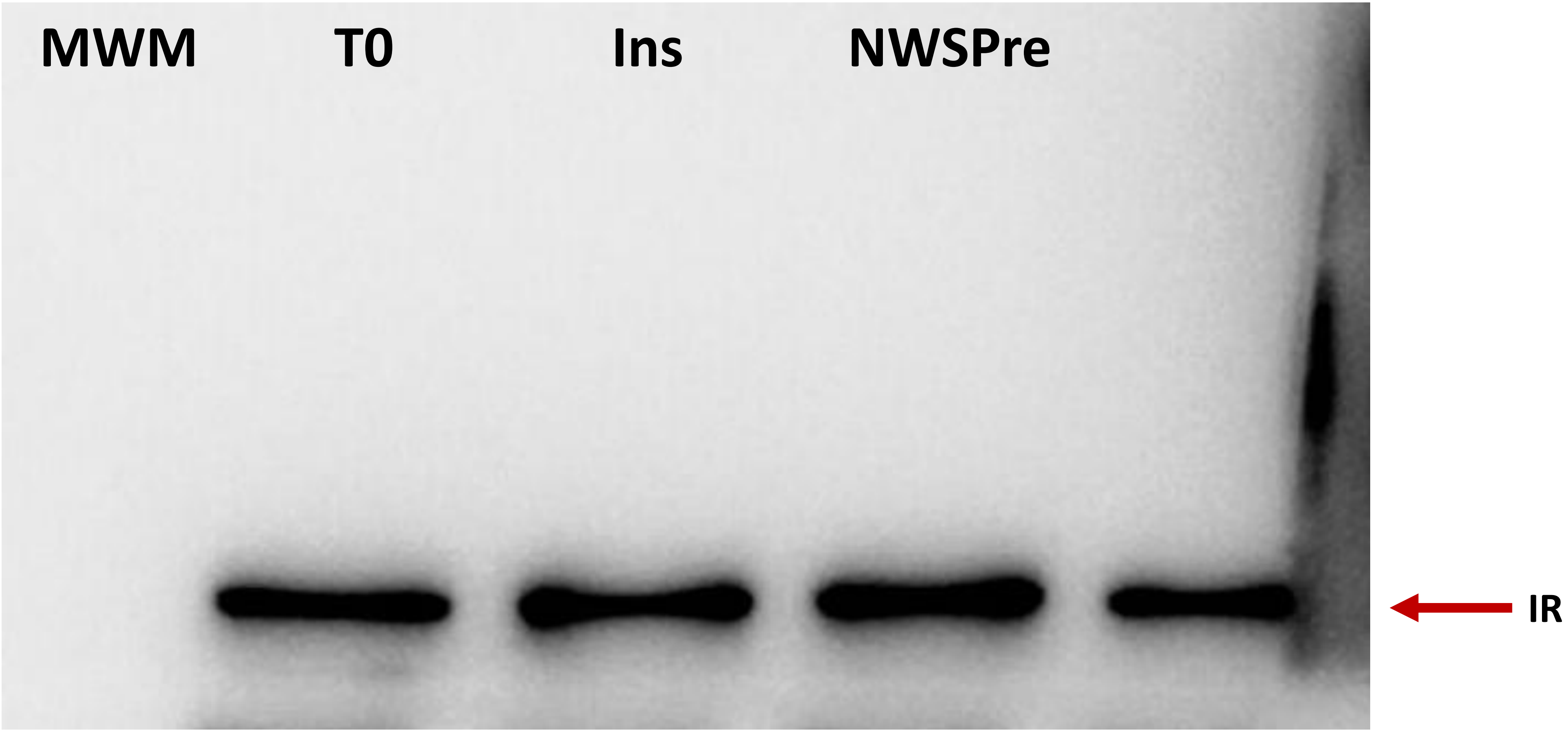

Figure 3A

MCF-7 pAkt(Ser 473)

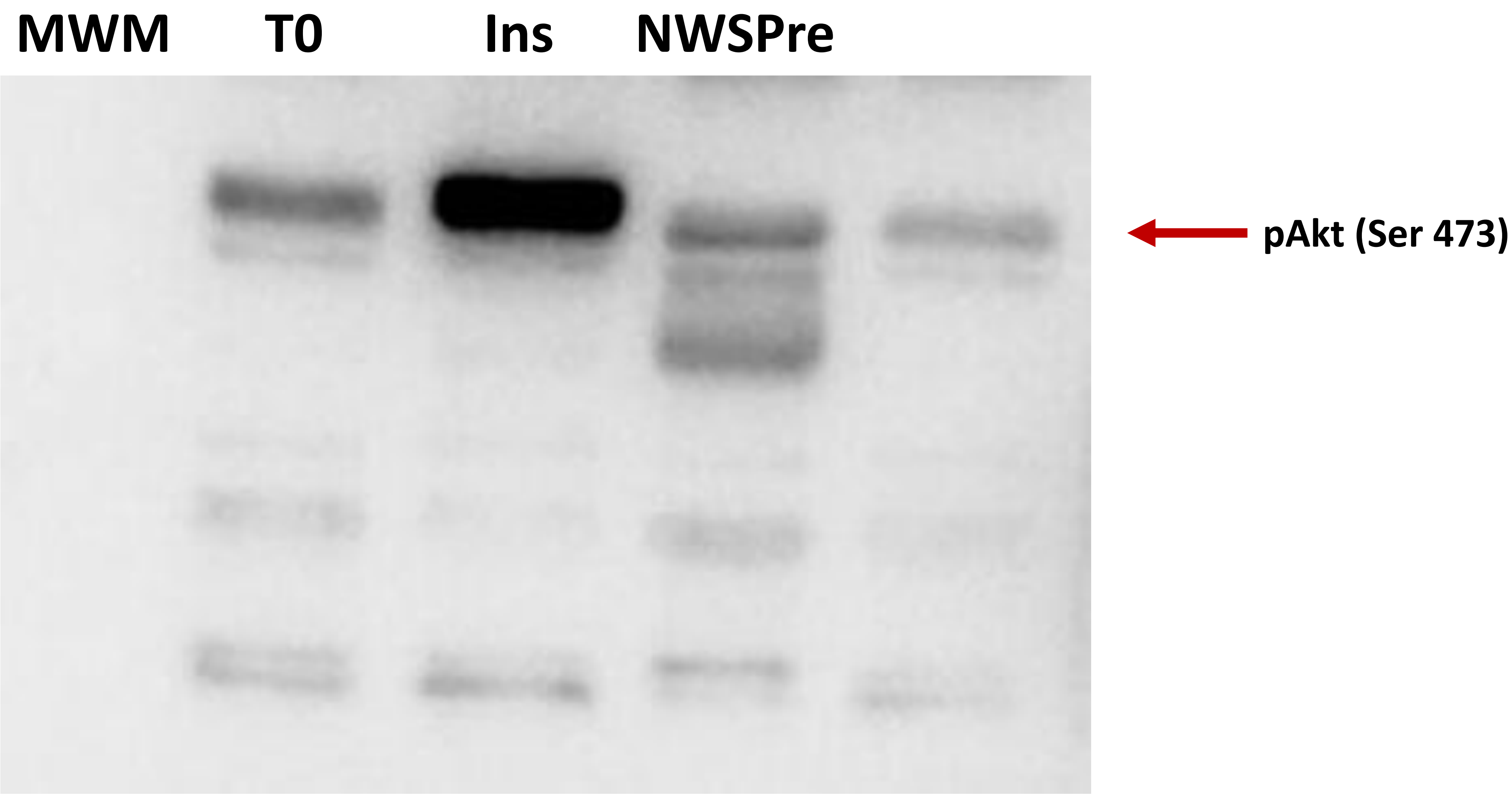

Supplementary Figure 3B

MCF-7 Akt

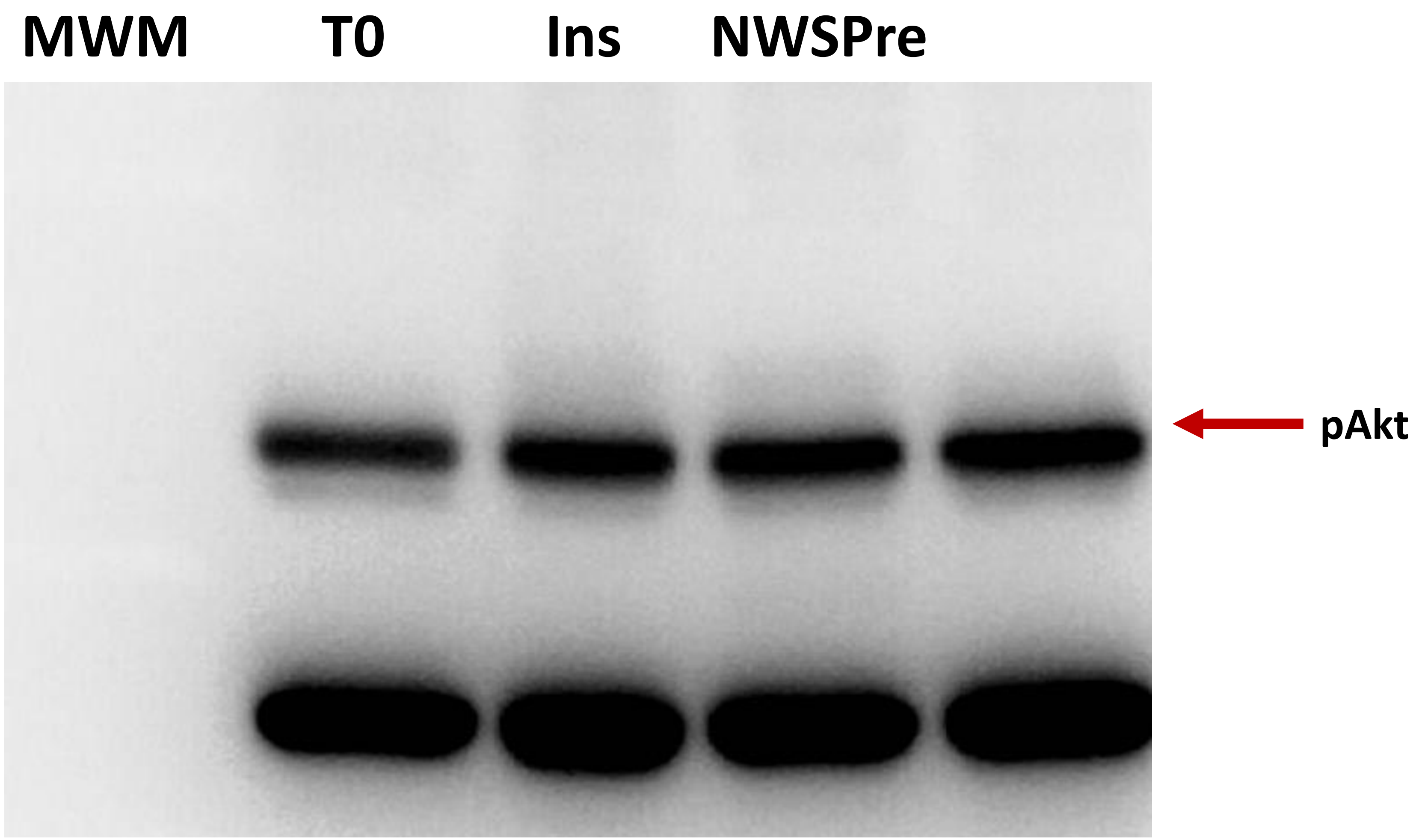

Supplementary Figure 3B

MCF-7 p70S6K

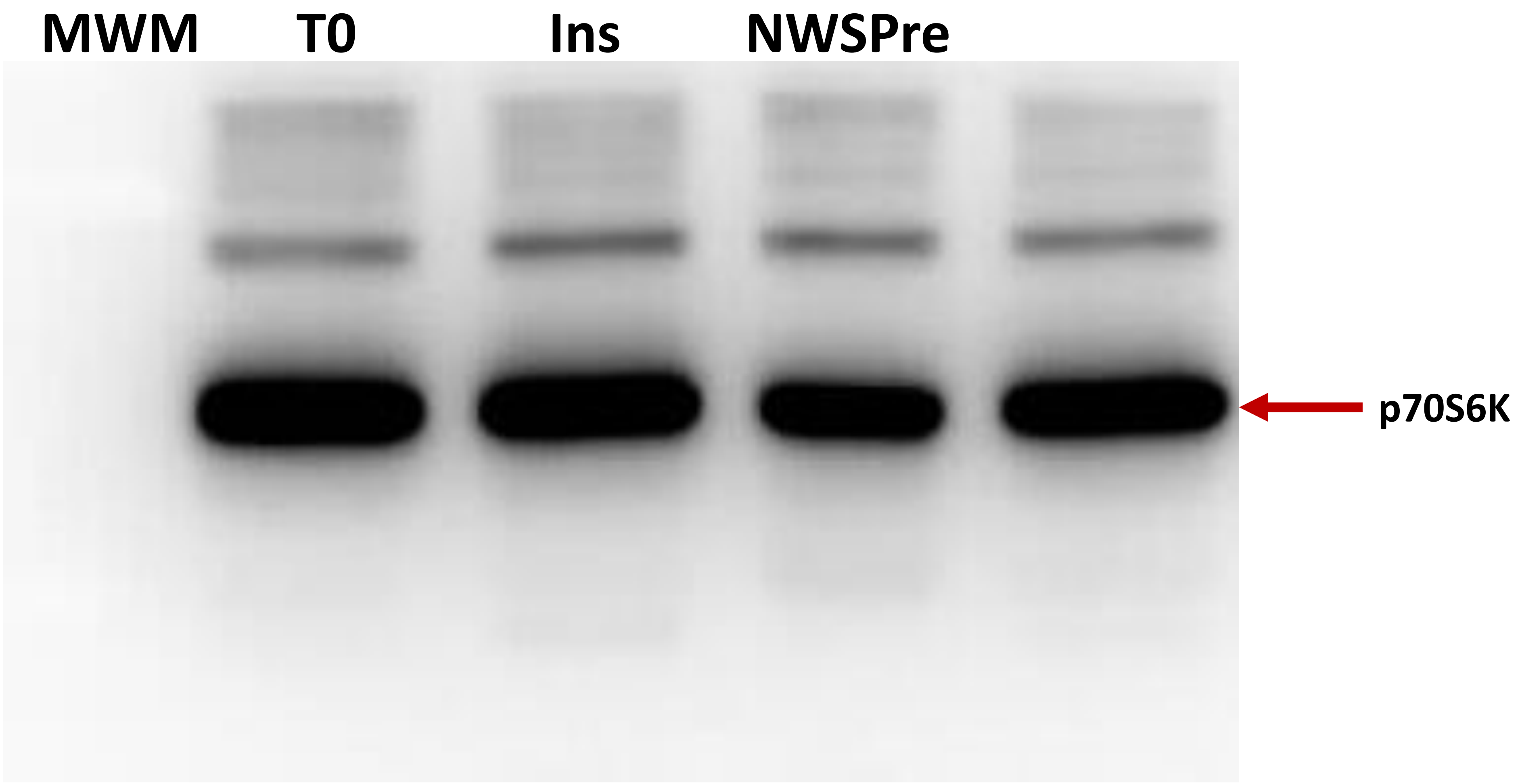

Supplementary Figure 3B

MCF-7 p70S6K(Thr 389)

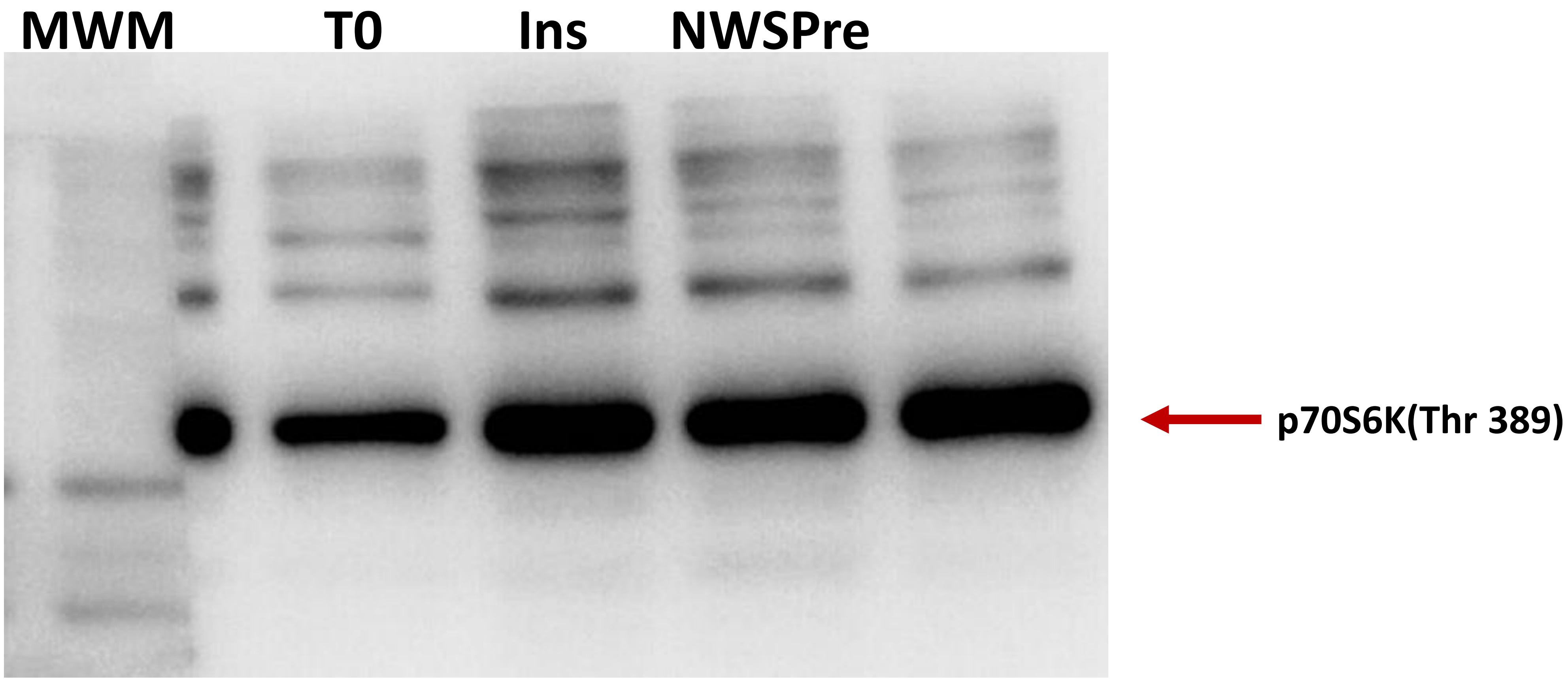

Supplementary Figure 3B

MCF-7 actin

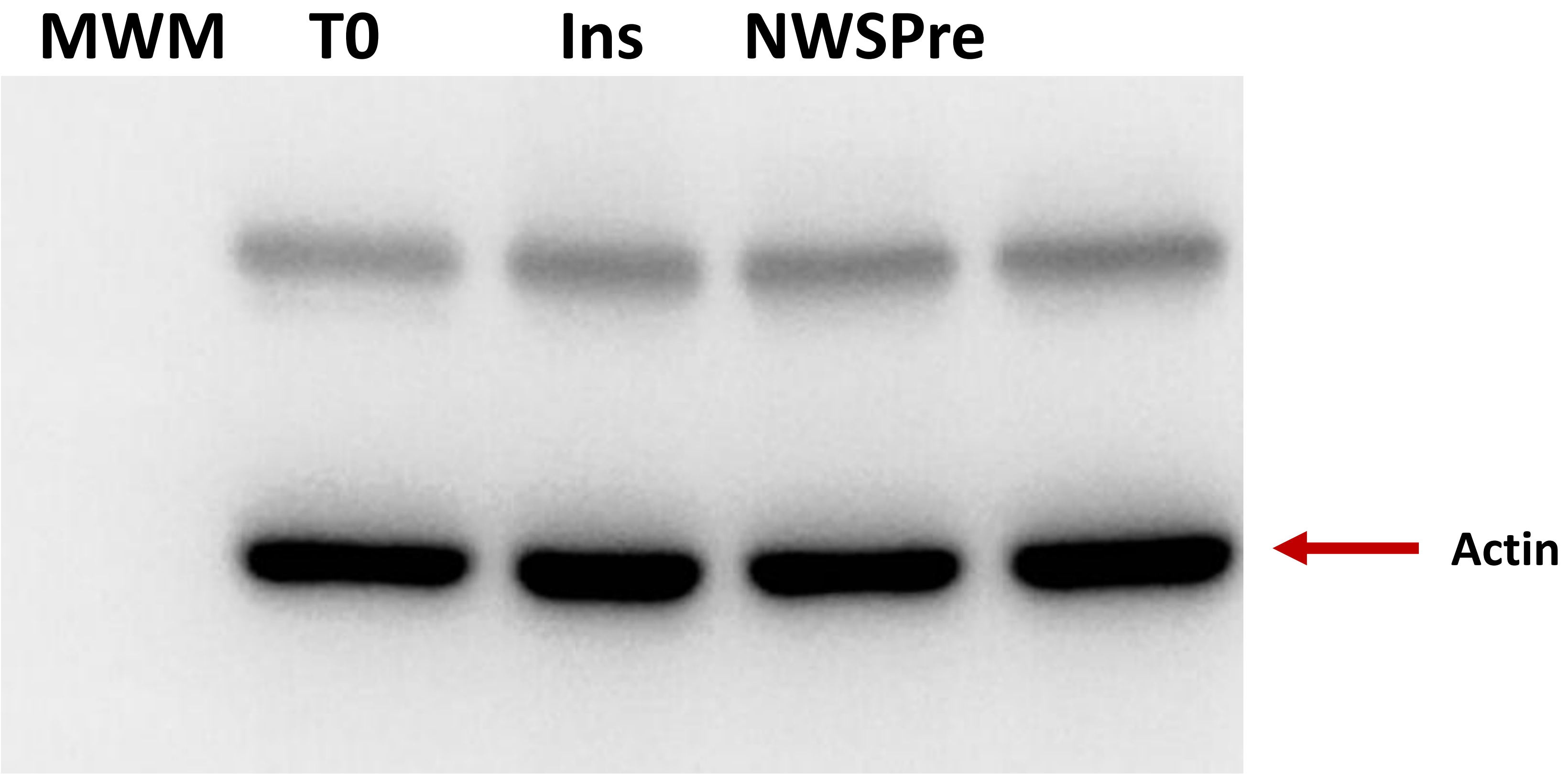

Supplementary Figure 3C

ZR-75-30 pIR

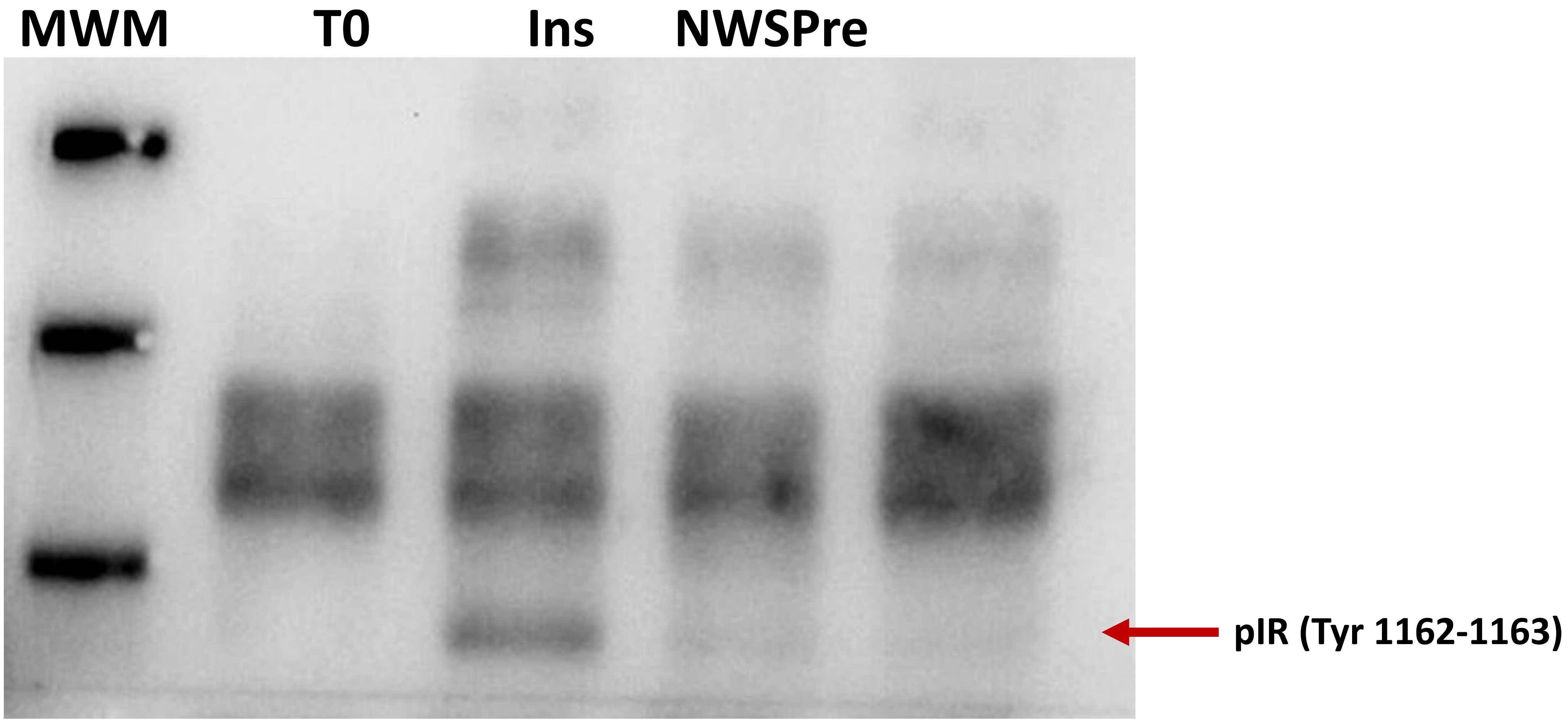

Supplementary Figure 3C

ZR-75-30 IR

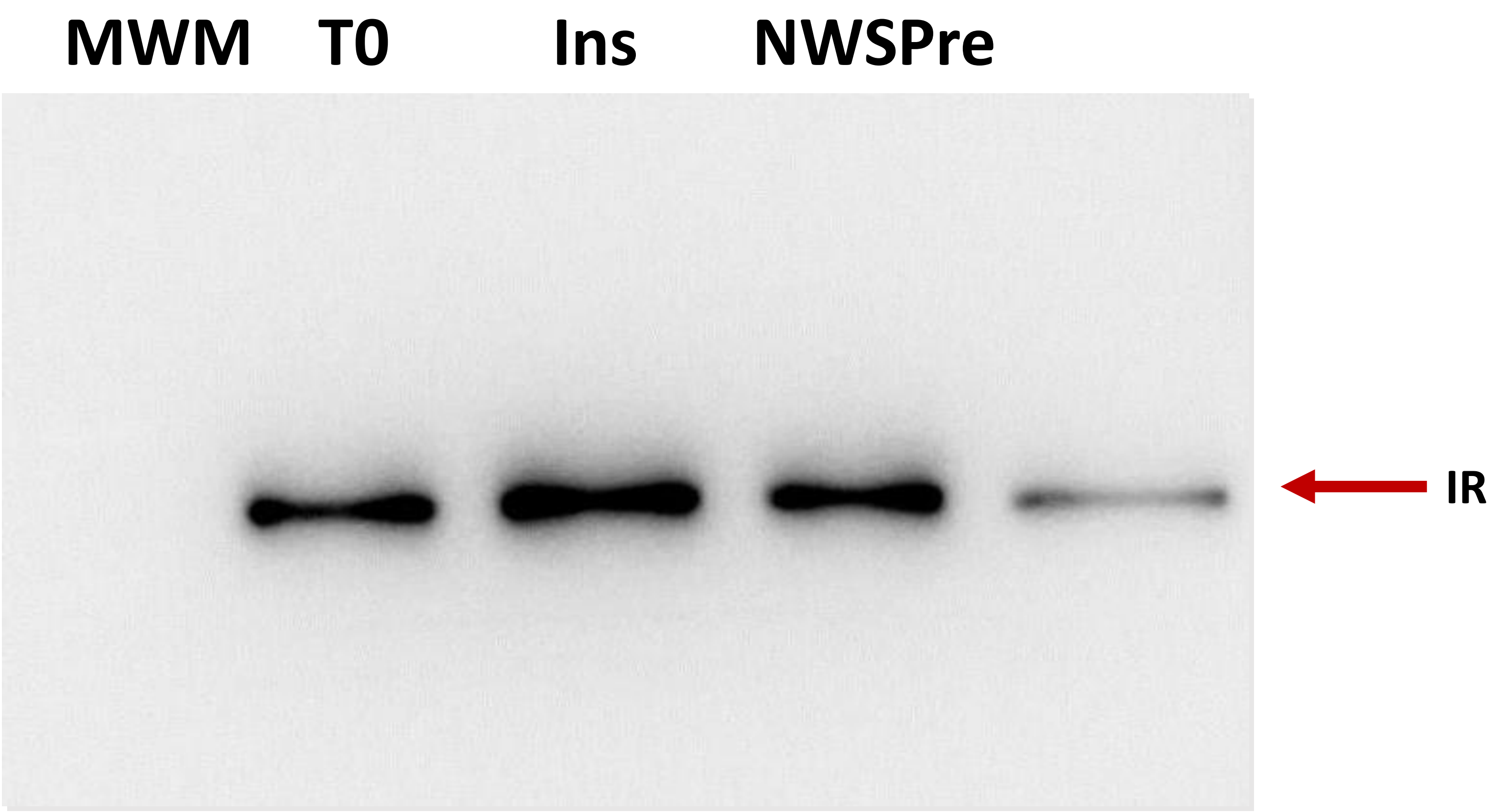

Supplementary Figure 3C

ZR-75-30 pAkt(Ser 473)

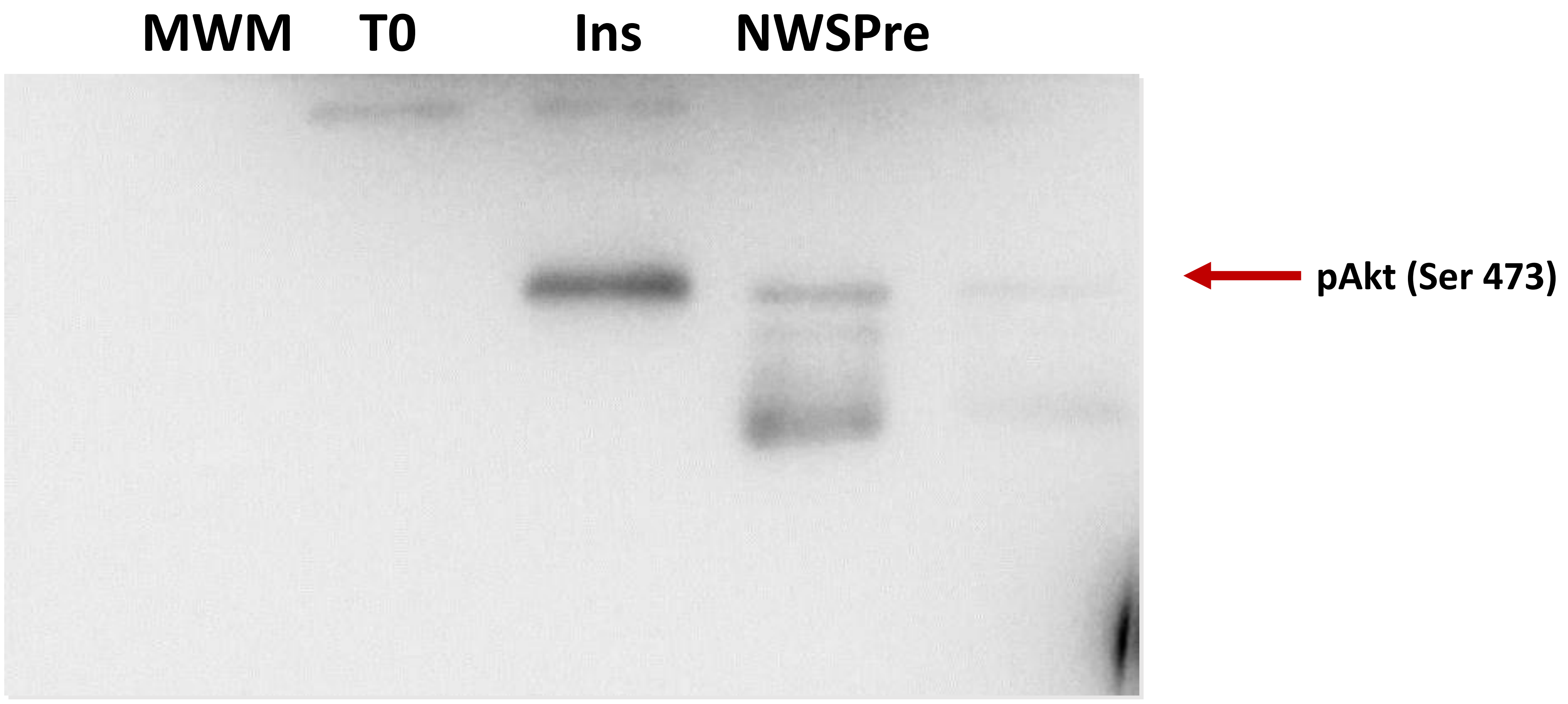

Supplementary Figure 3C

ZR-75-30 Akt

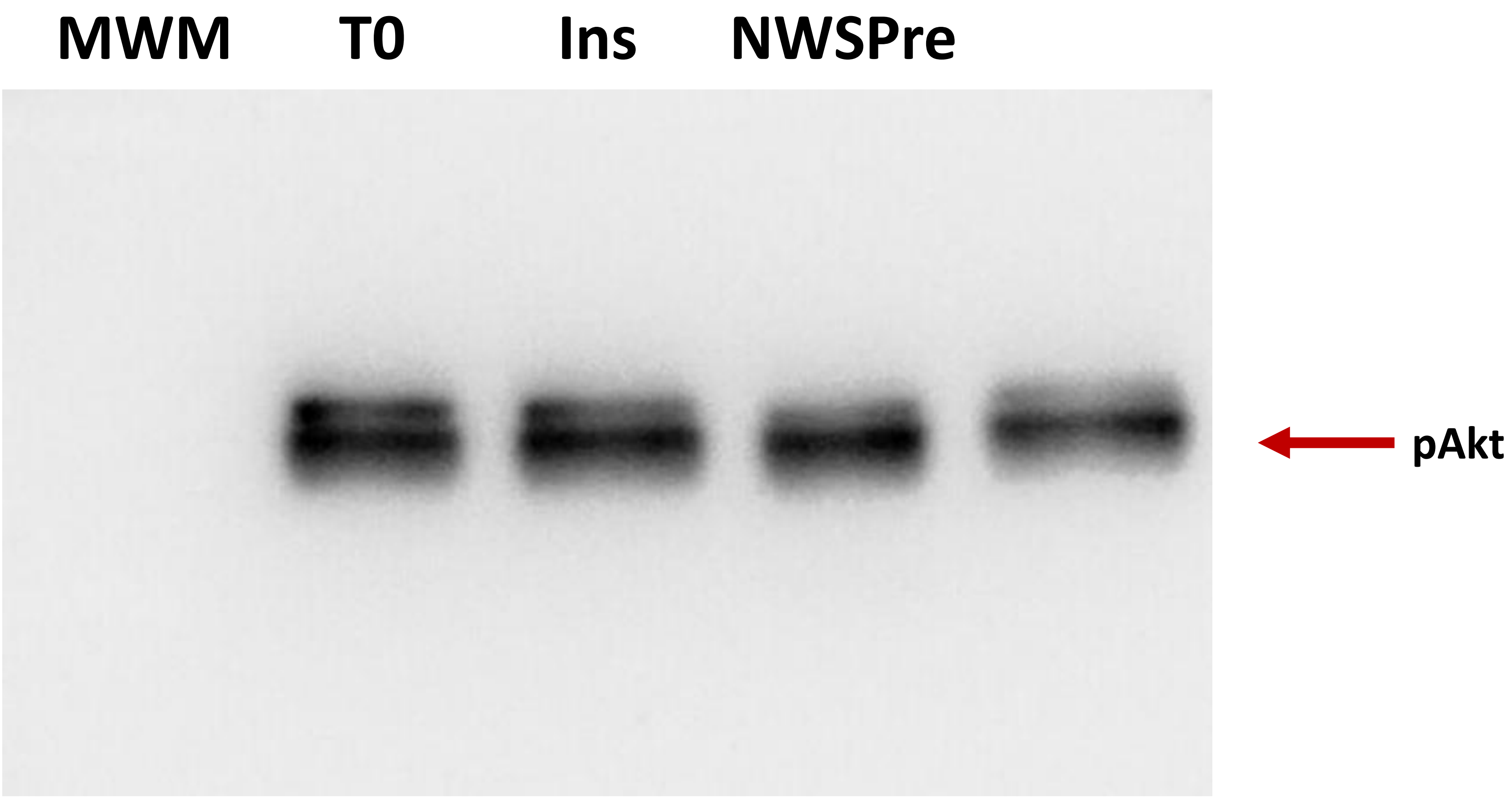

Supplementary Figure 3C

ZR-75-30 p70S6K

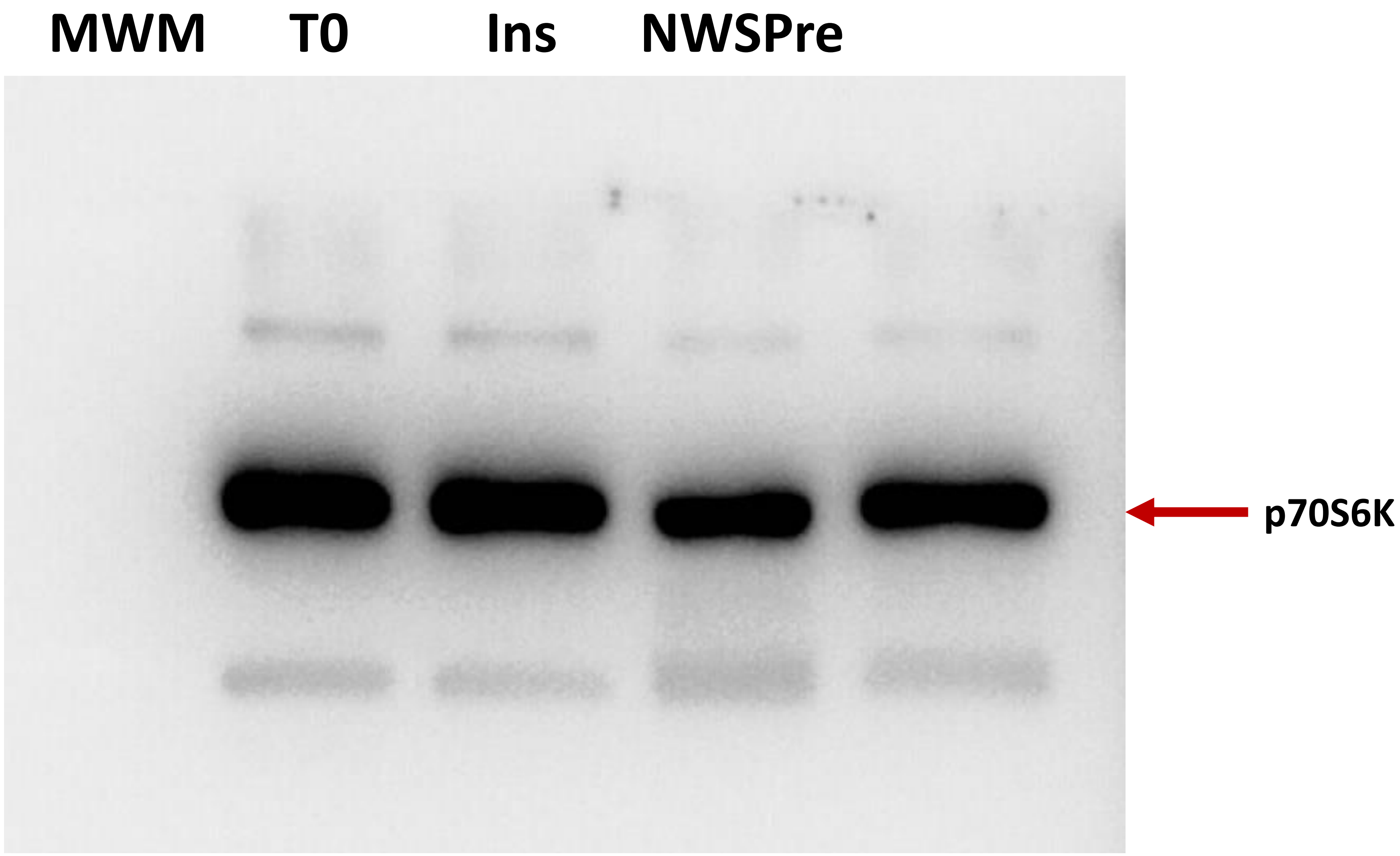

Supplementary Figure 3C

ZR-75-30 p70S6K(Thr 389)

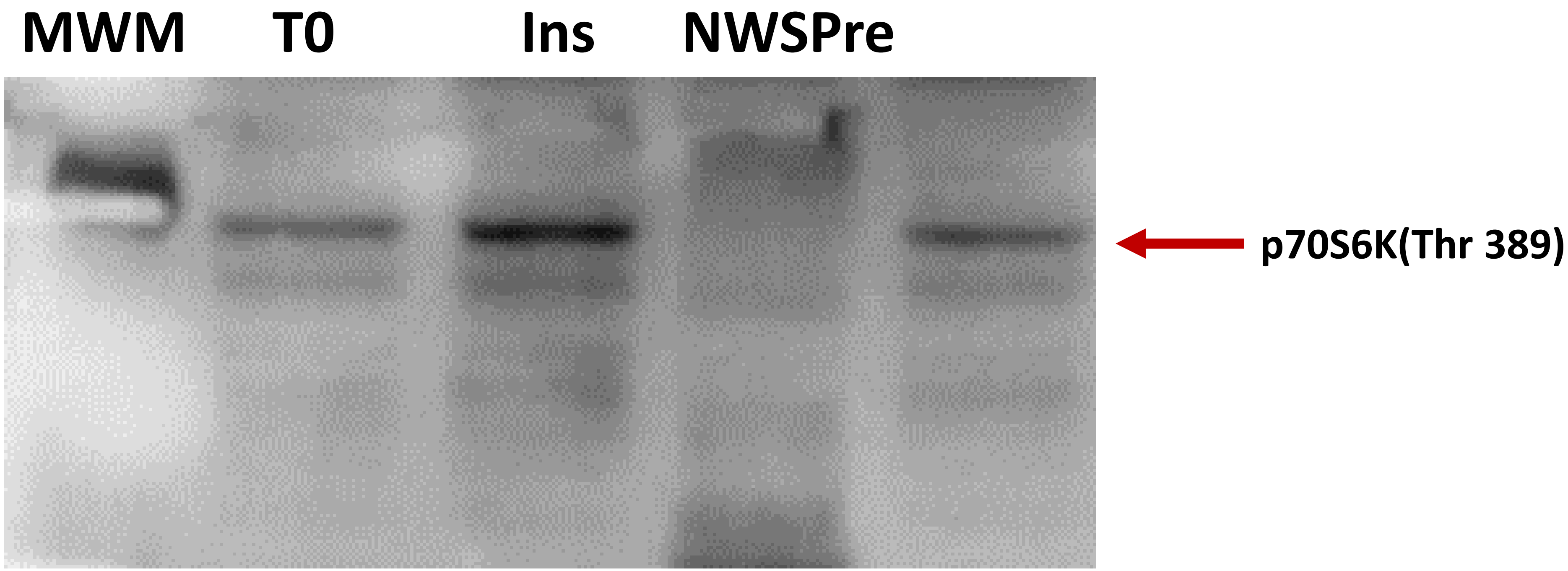

Supplementary Figure 3C

ZR-75-30 actin

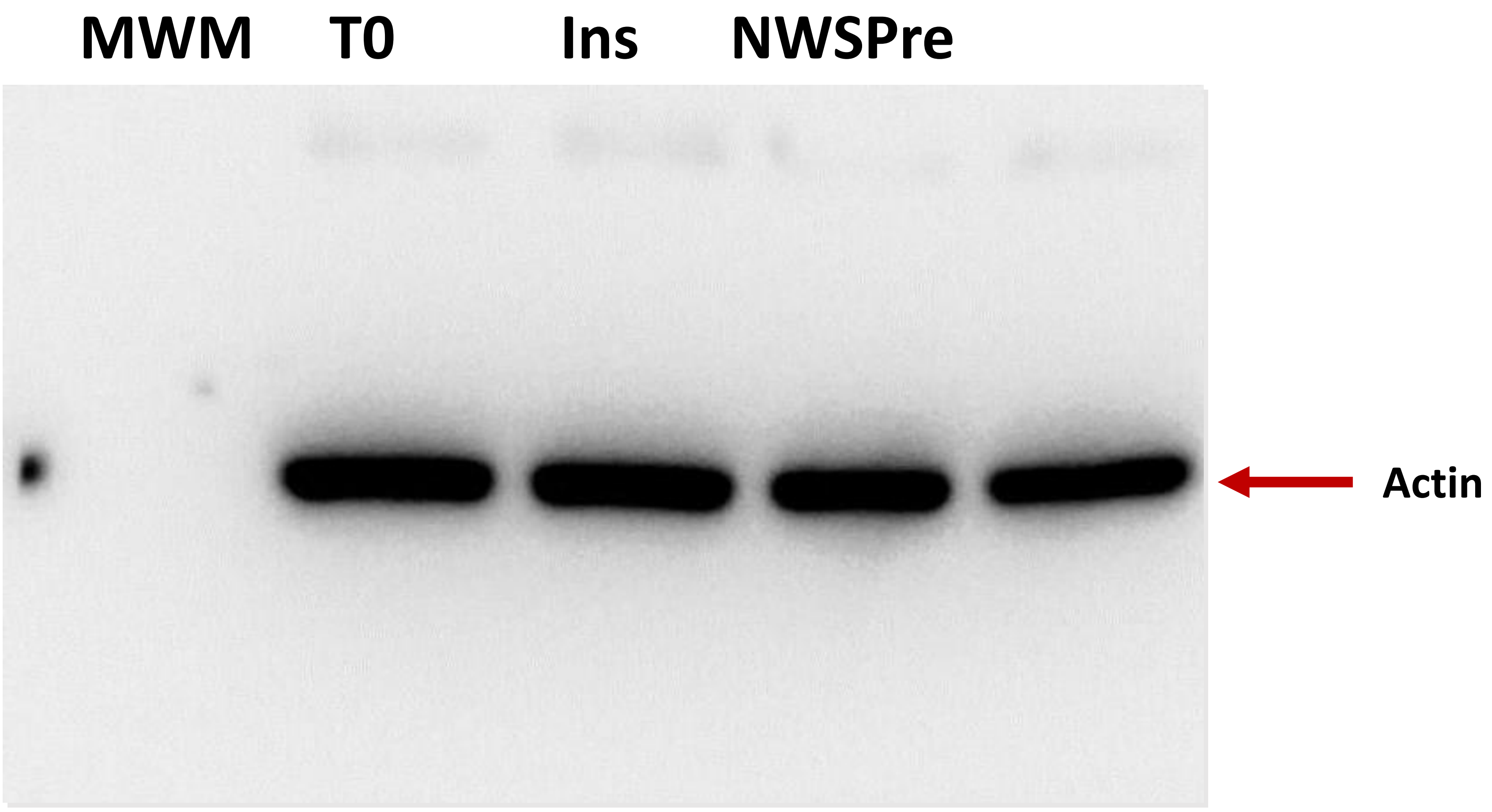

Supplementary Figure 3D

BT-474 pIR

MWM T0 Ins NWSPre

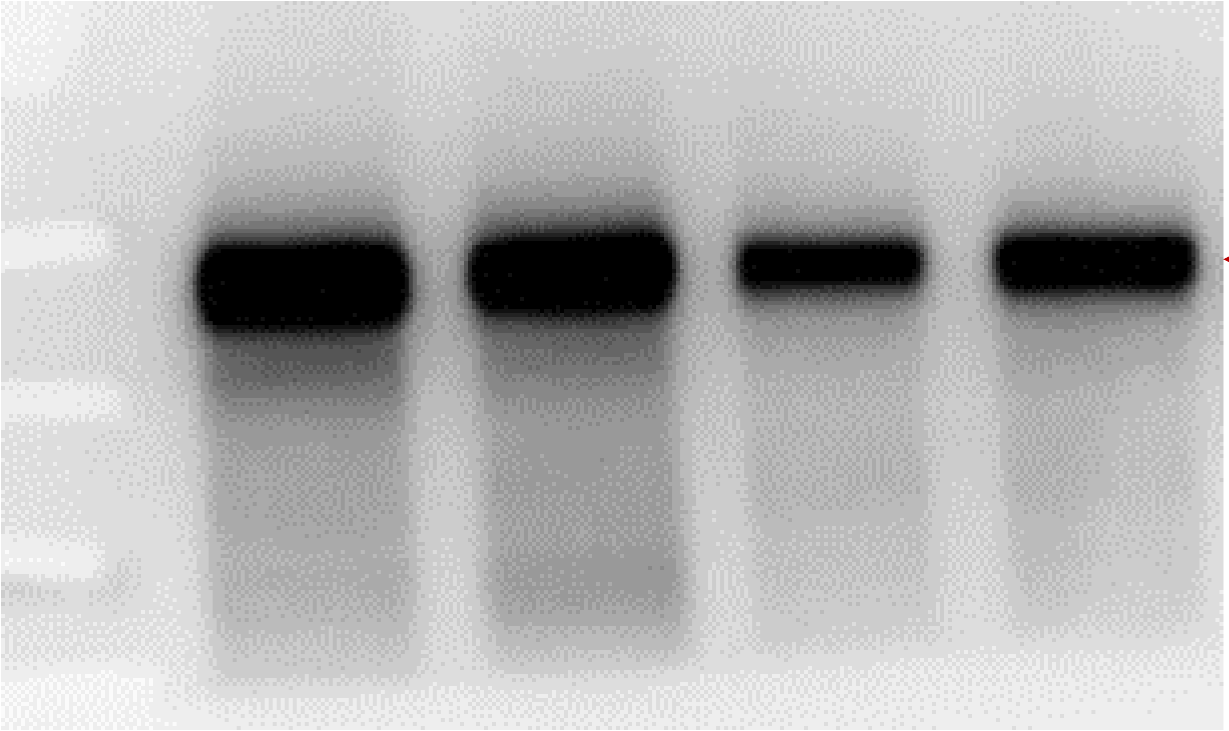

pIR (Tyr 1162-1163)

Supplementary Figure 3D

BT-474 IR

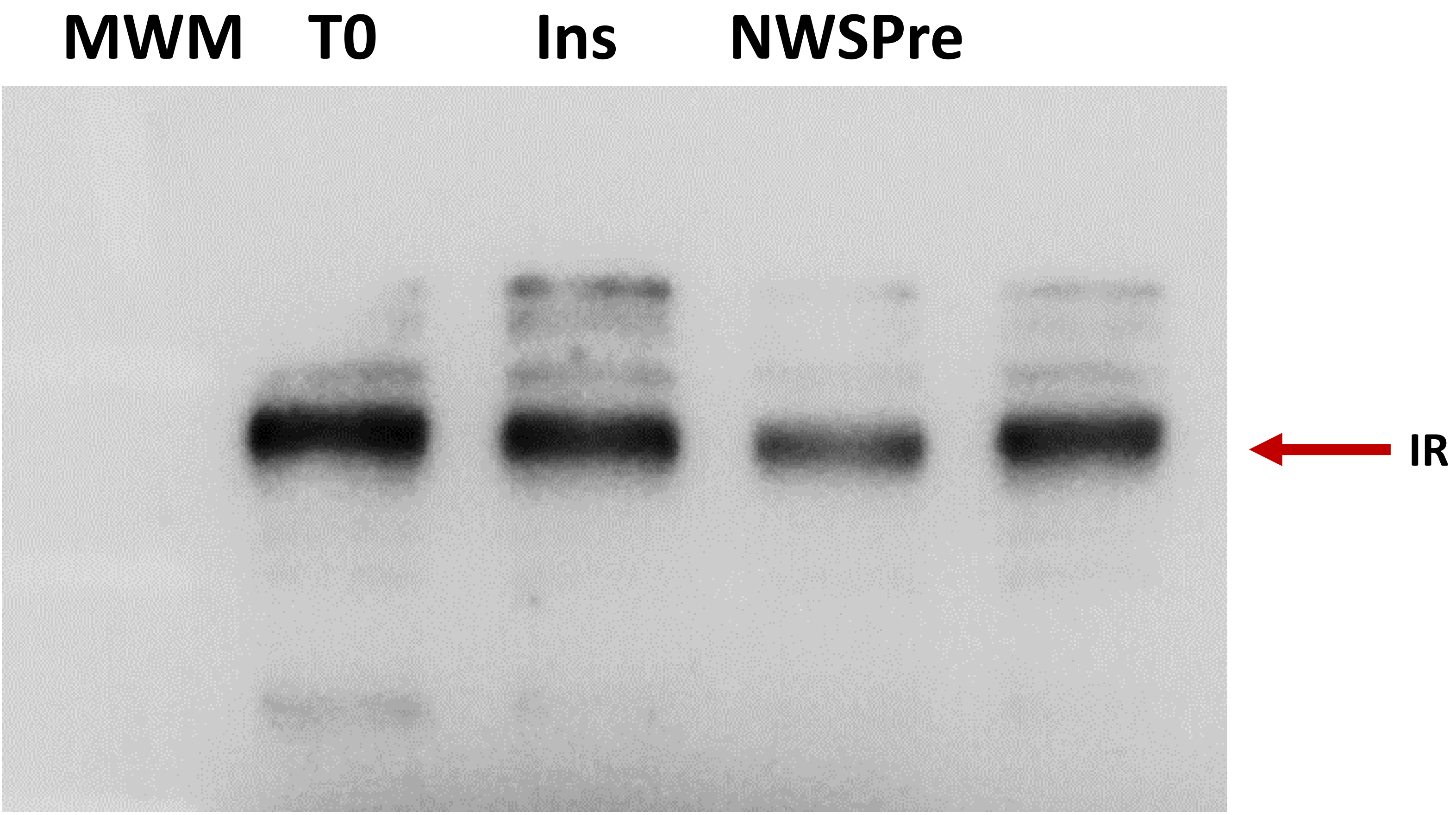

Supplementary Figure 3D

BT-474 pAkt(Ser 473)

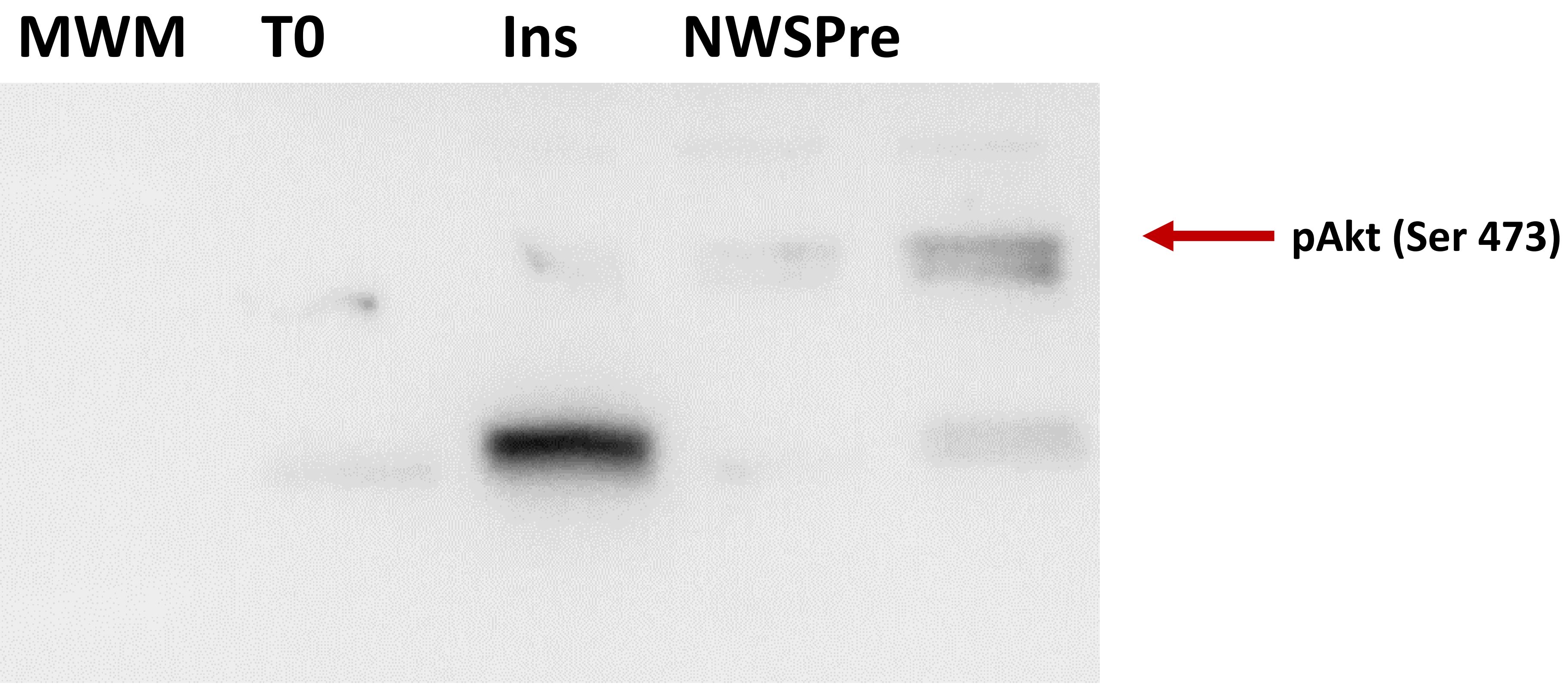

Supplementary Figure 3D

BT-474 Akt

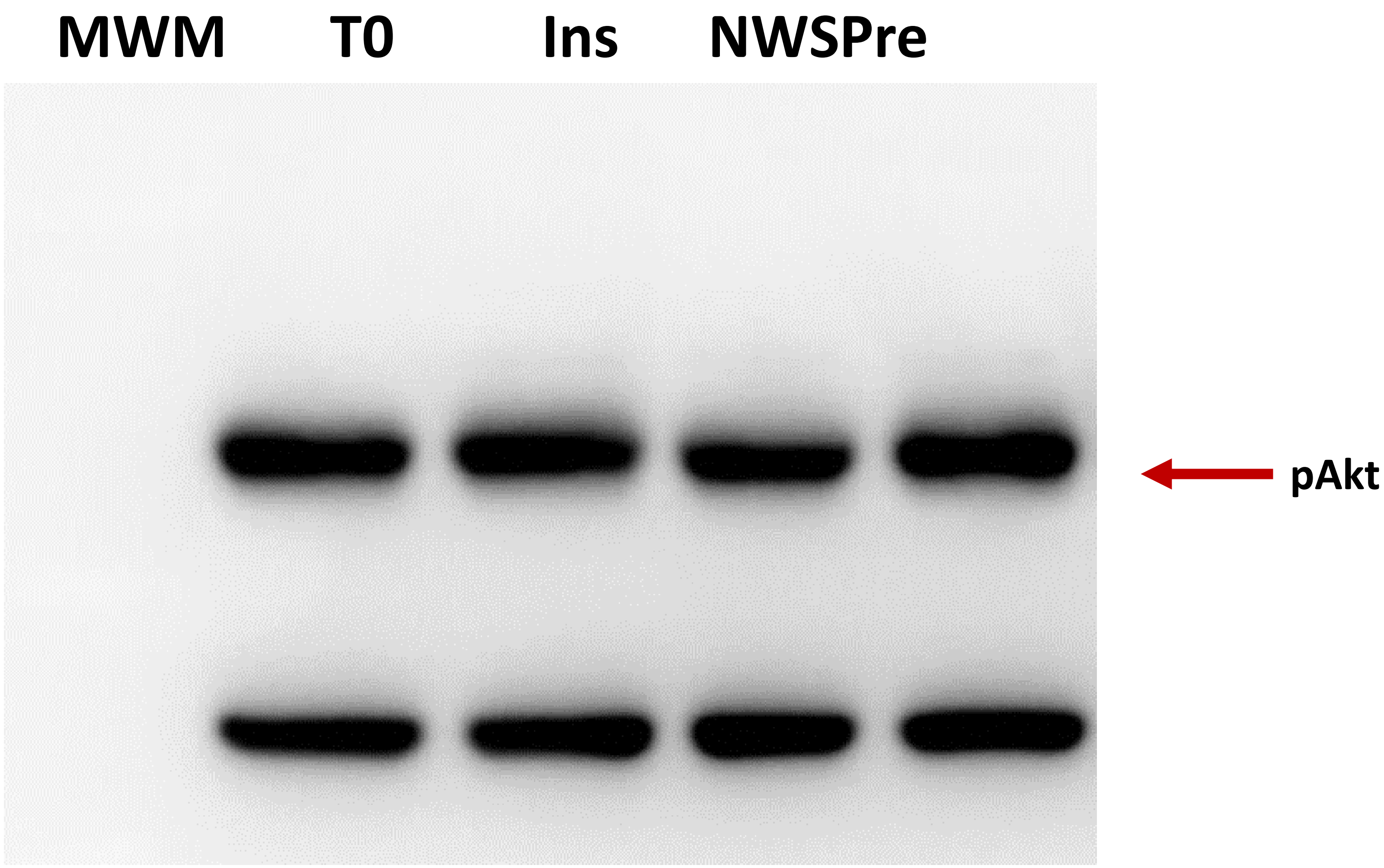

Supplementary Figure 3D

BT-474 p70S6K

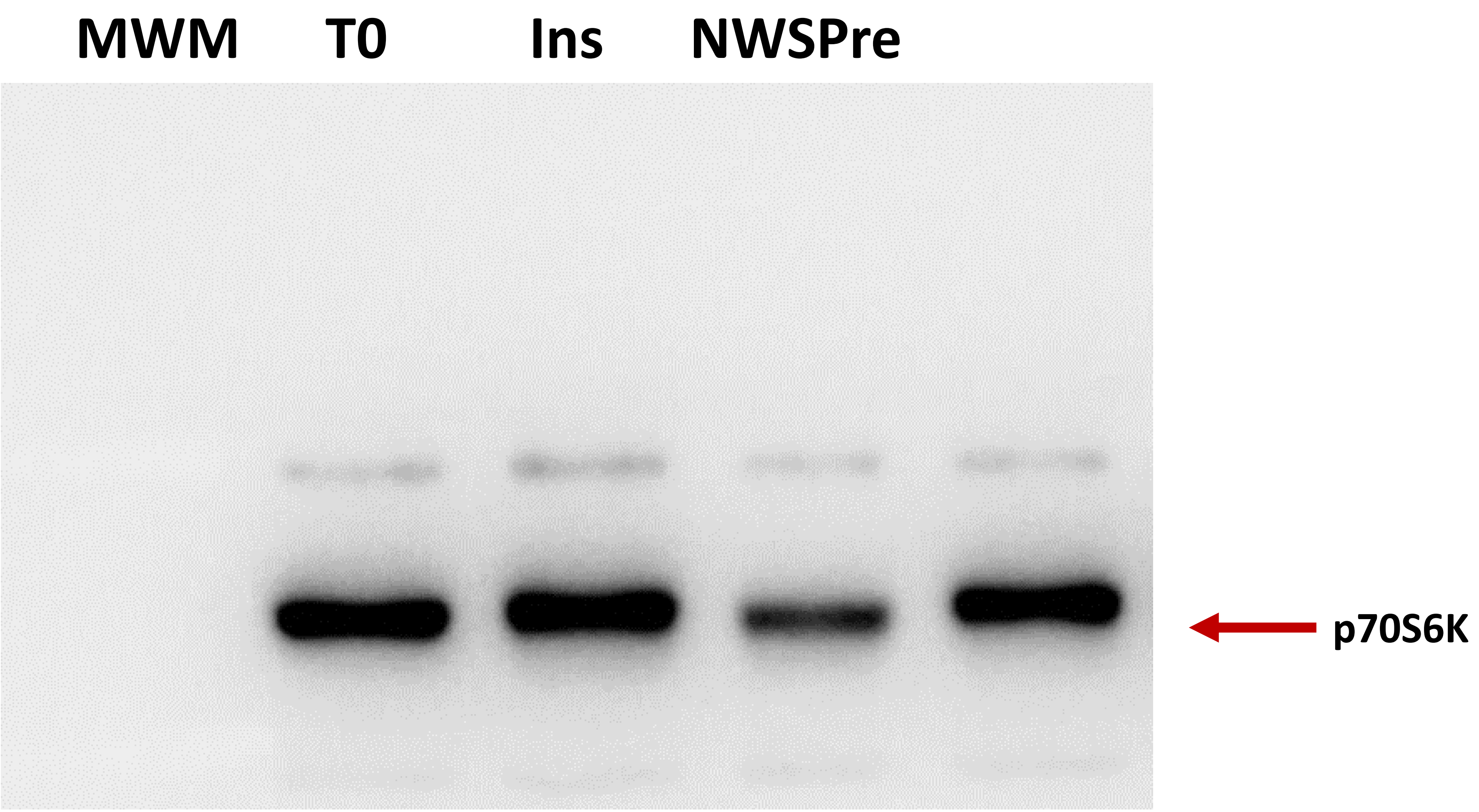

Supplementary Figure 3D

BT-474 p70S6K(Thr 389)

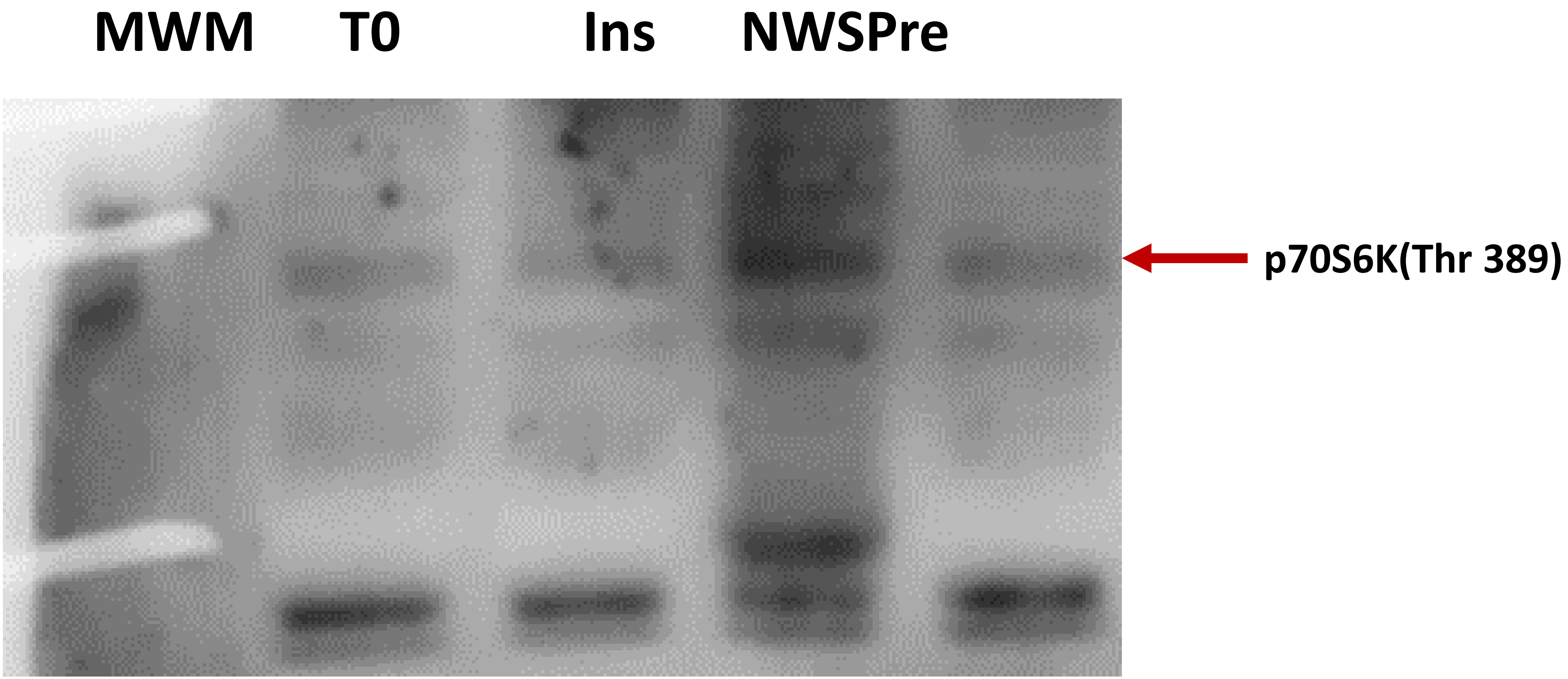

Supplementary Figure 3D

BT-474 actin

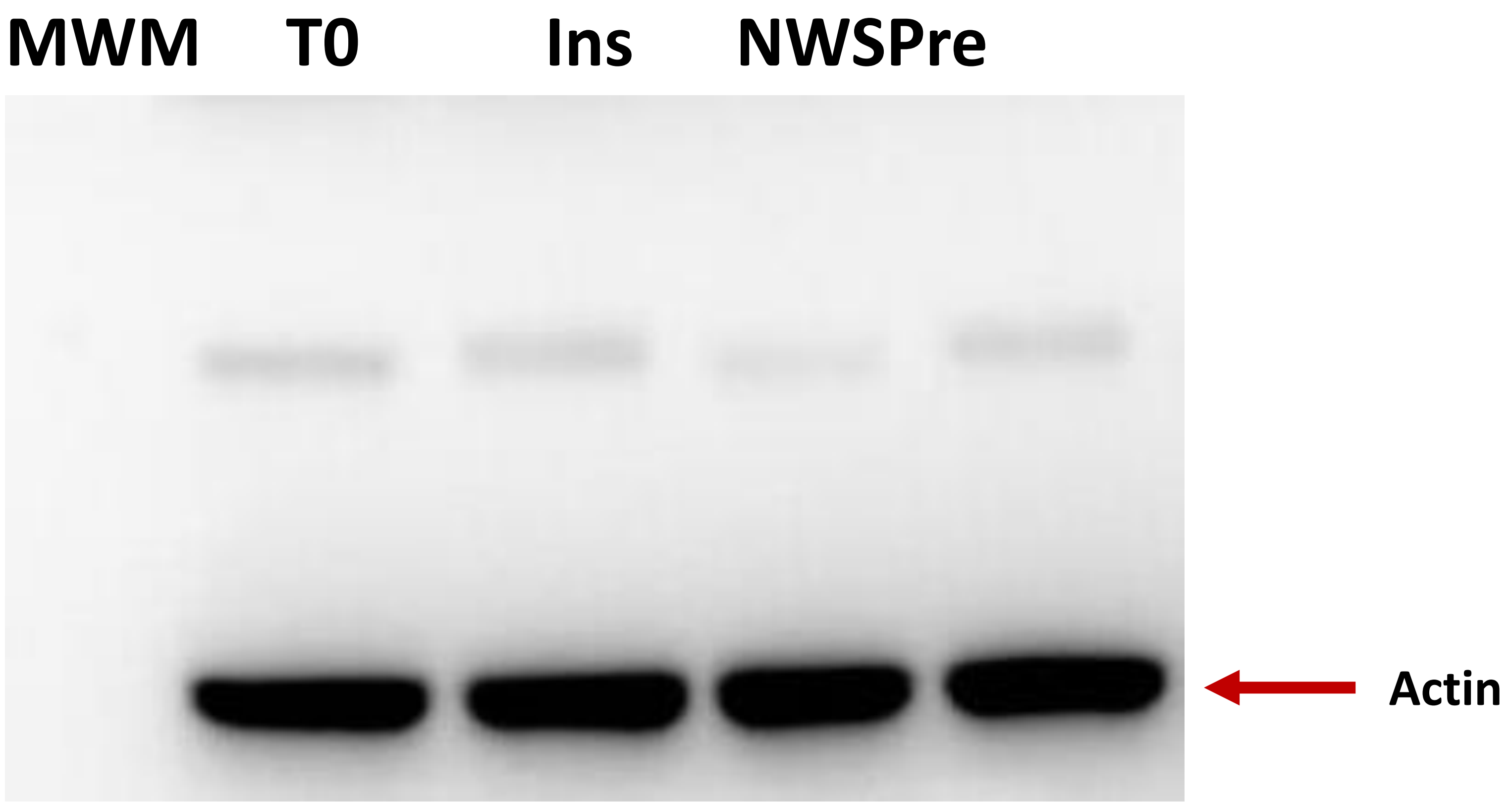

Supplementary Figure 3E

BT-474R pIR

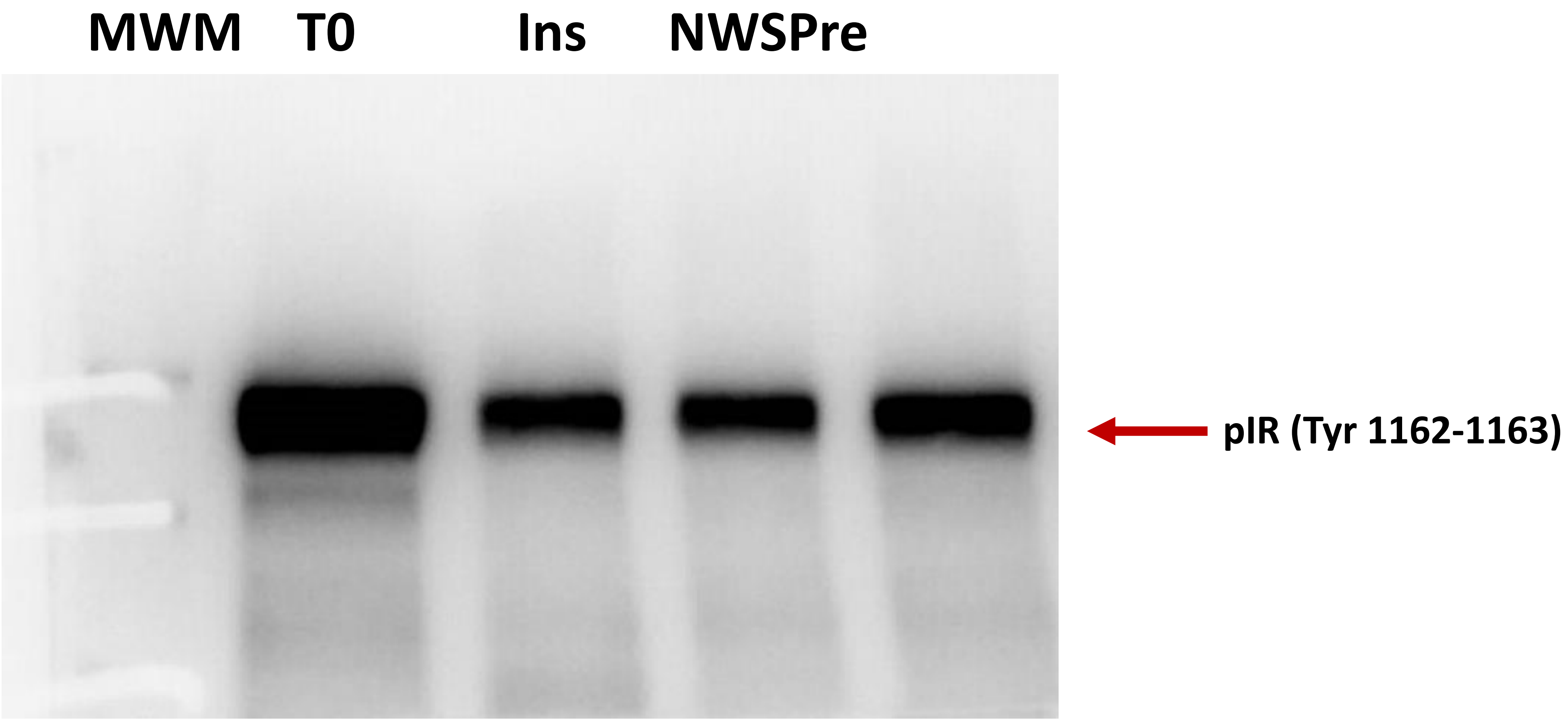

Supplementary Figure 3E

BT-474R IR

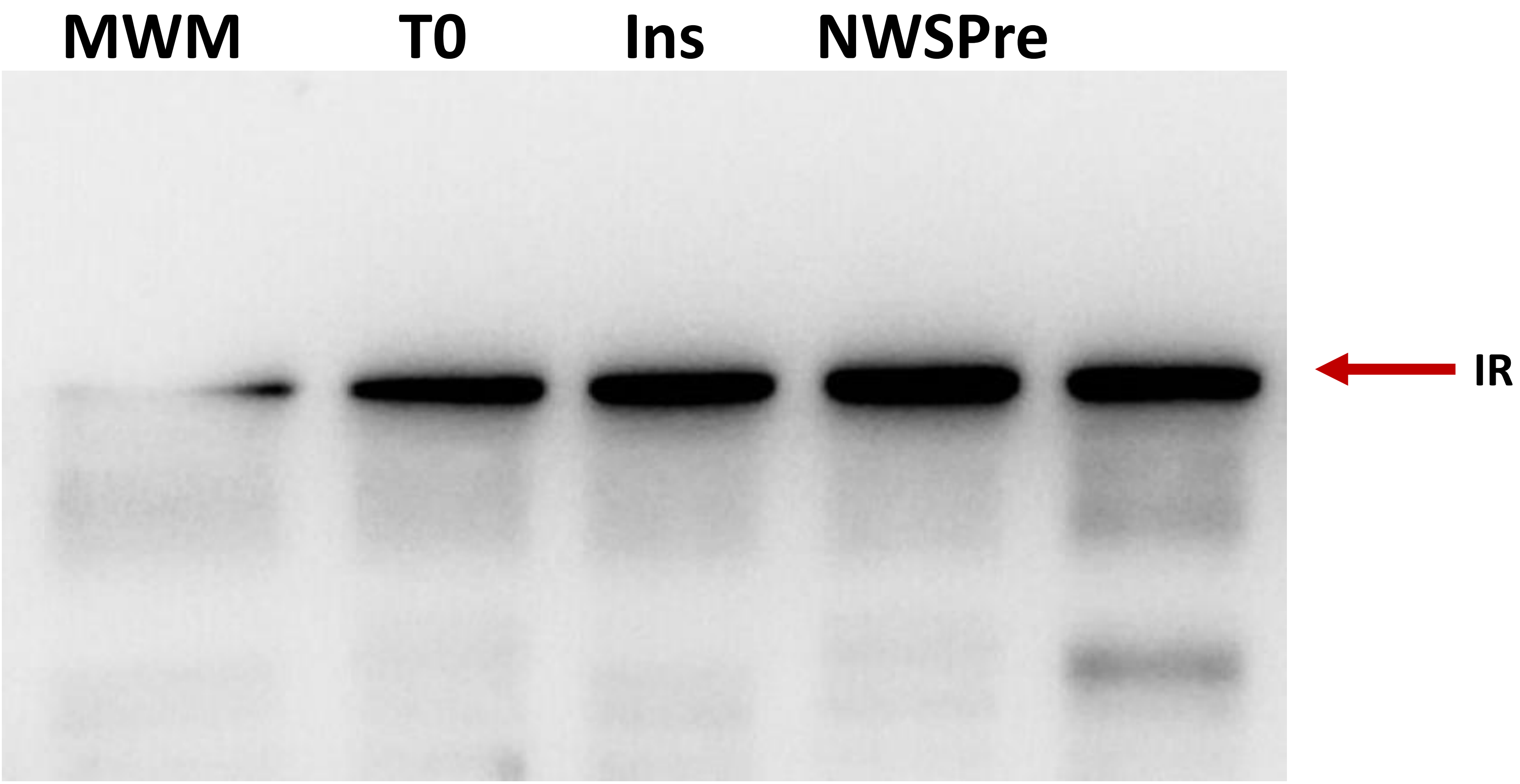

Supplementary Figure 3E

BT-474R pAkt(Ser 473)

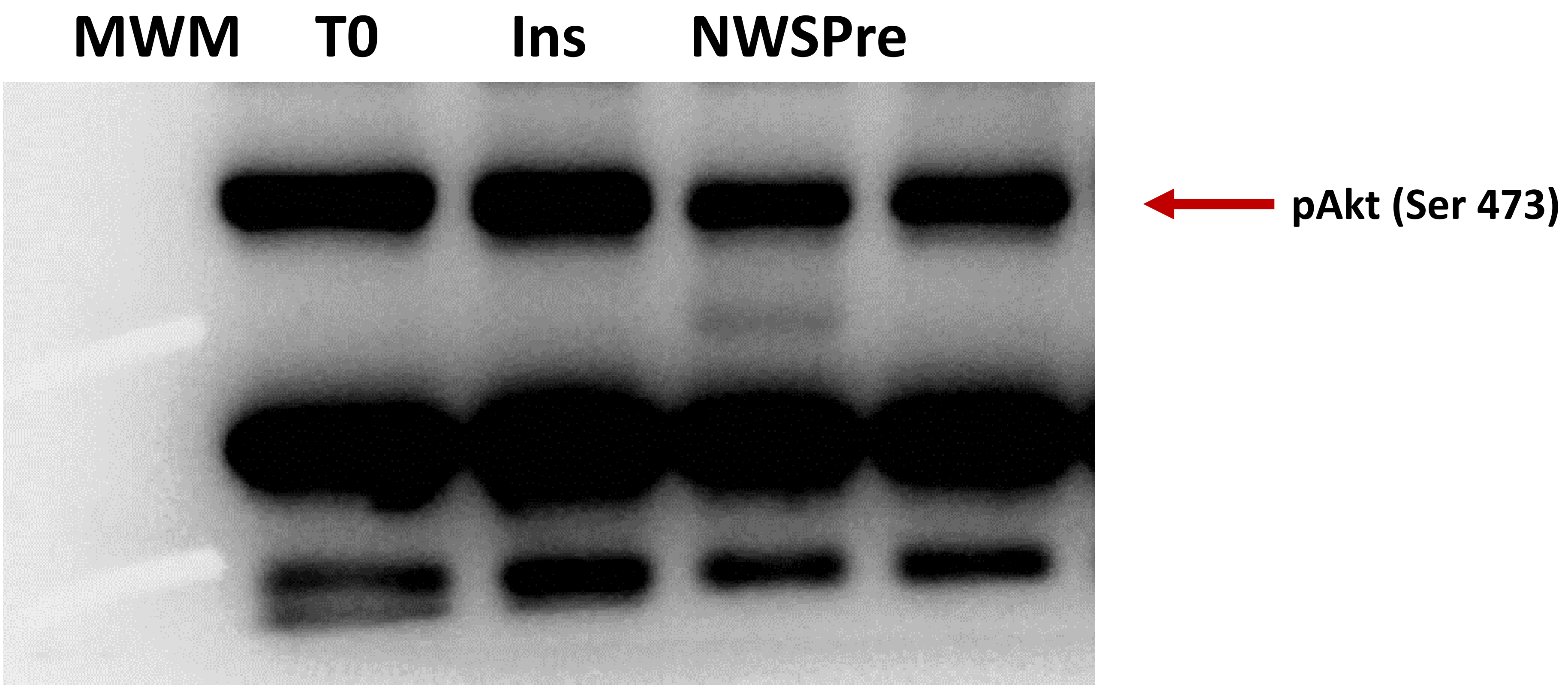

Supplementary Figure 3E

BT-474R Akt

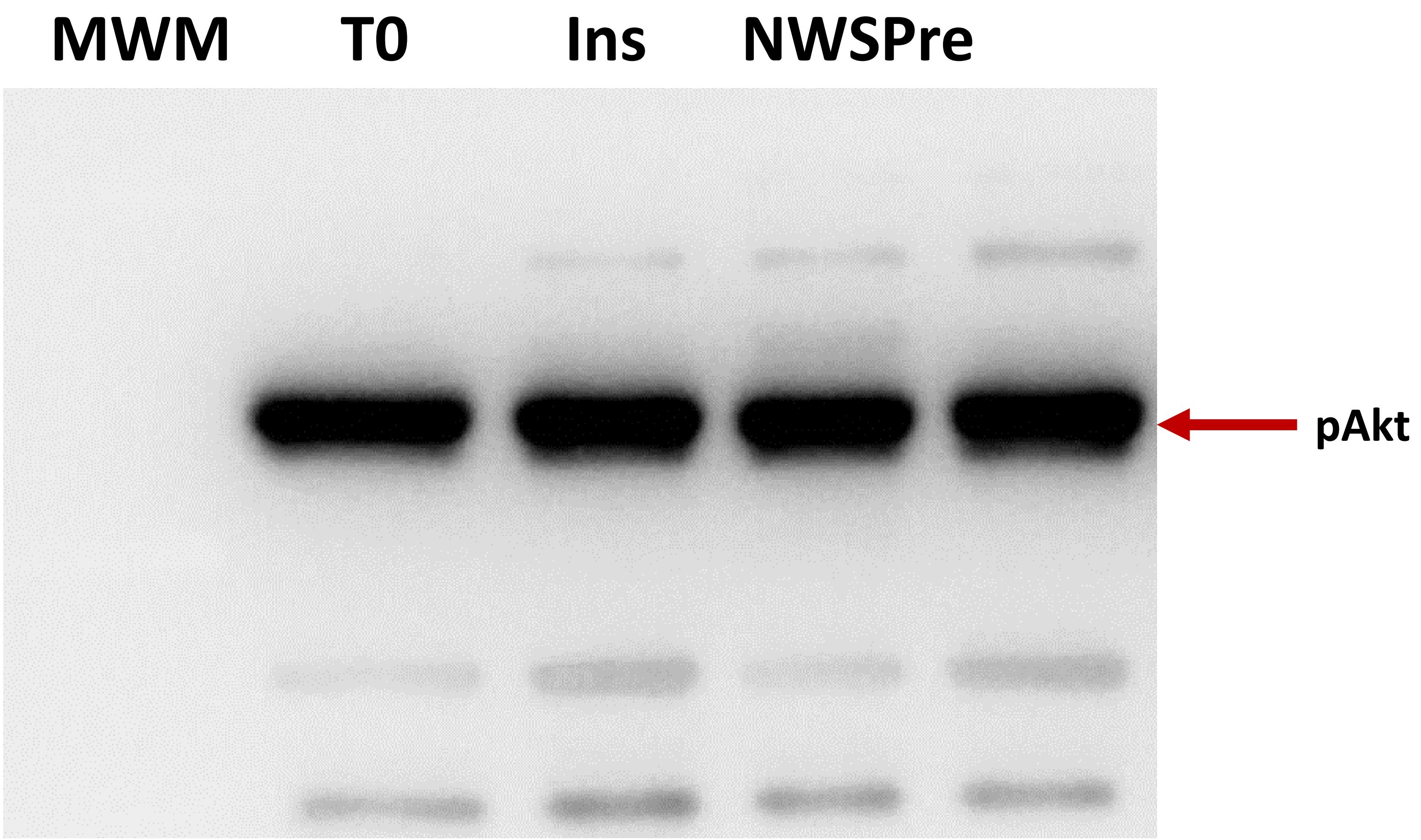

Supplementary Figure 3E

BT-474R p70S6K

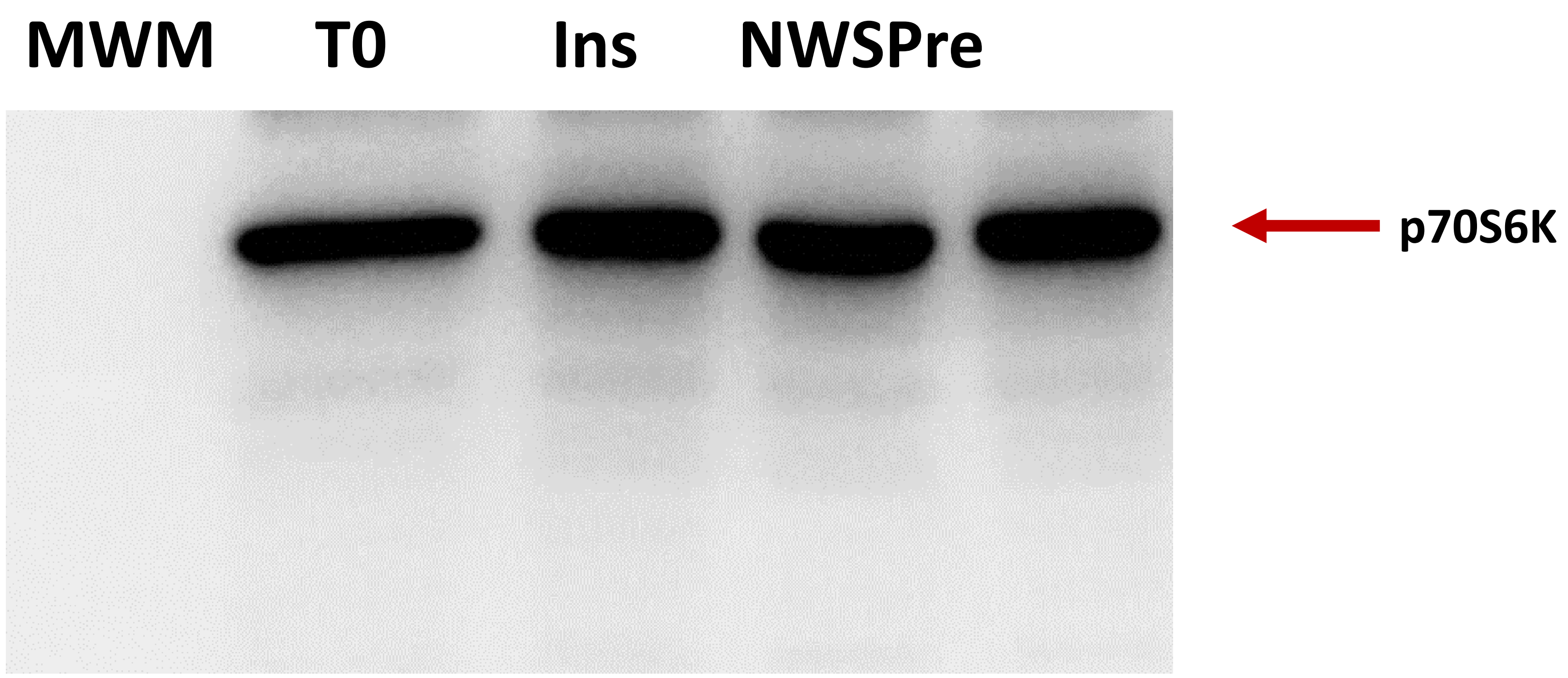

Supplementary Figure 3E

BT-474R p70S6K(Thr 389)

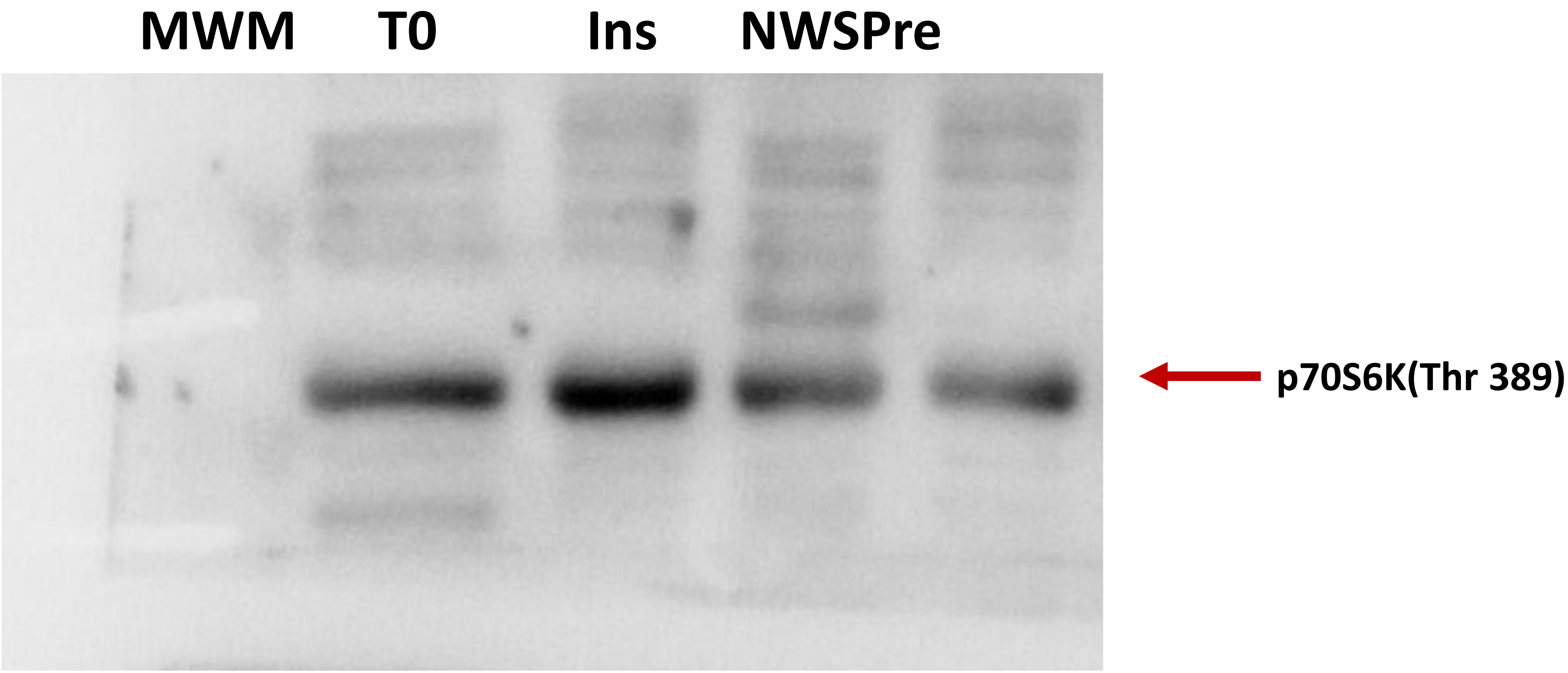

Supplementary Figure 3E

BT-474R actin

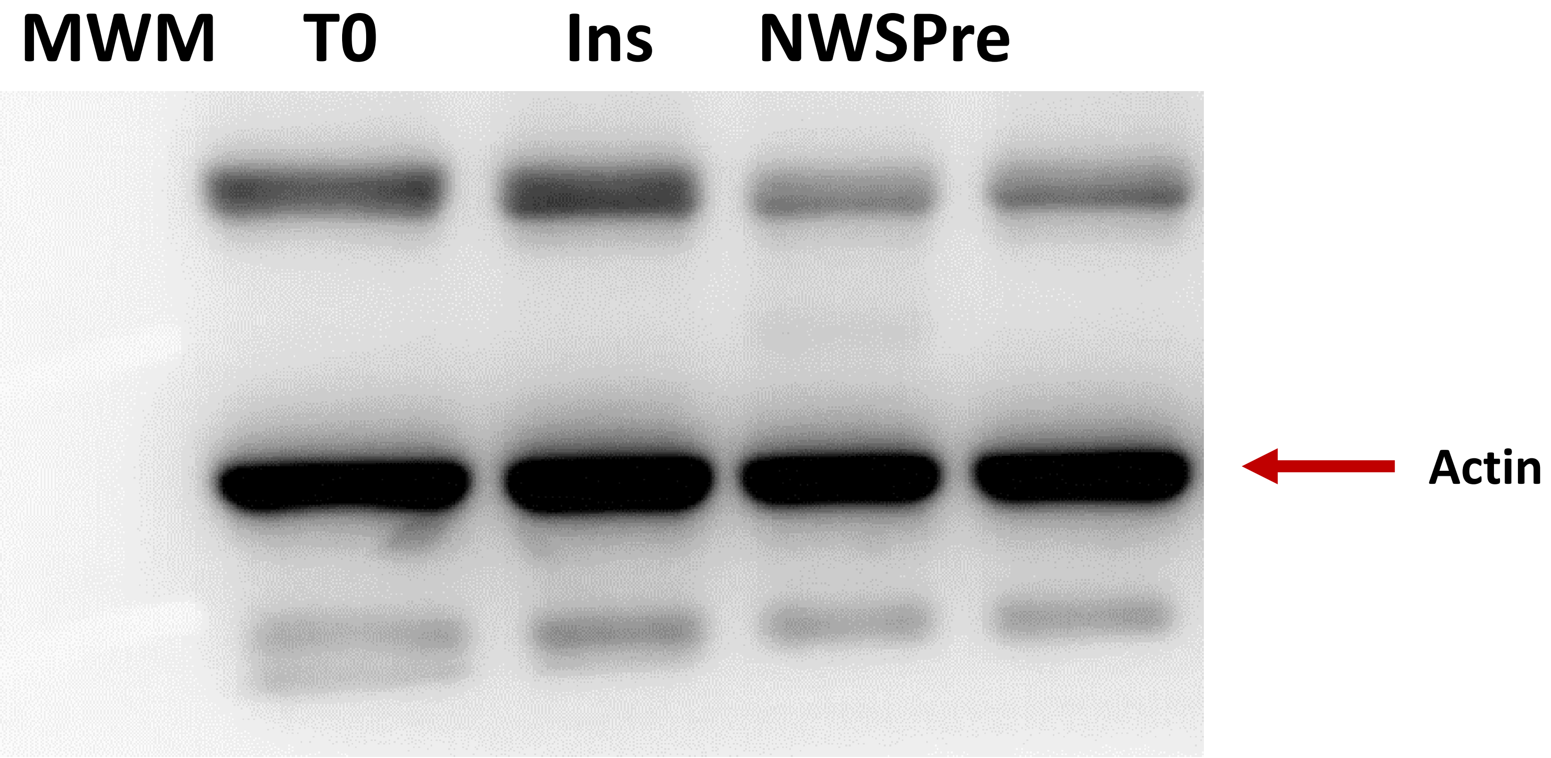

Supplementary Figure 3F

SKBR-3 pIR

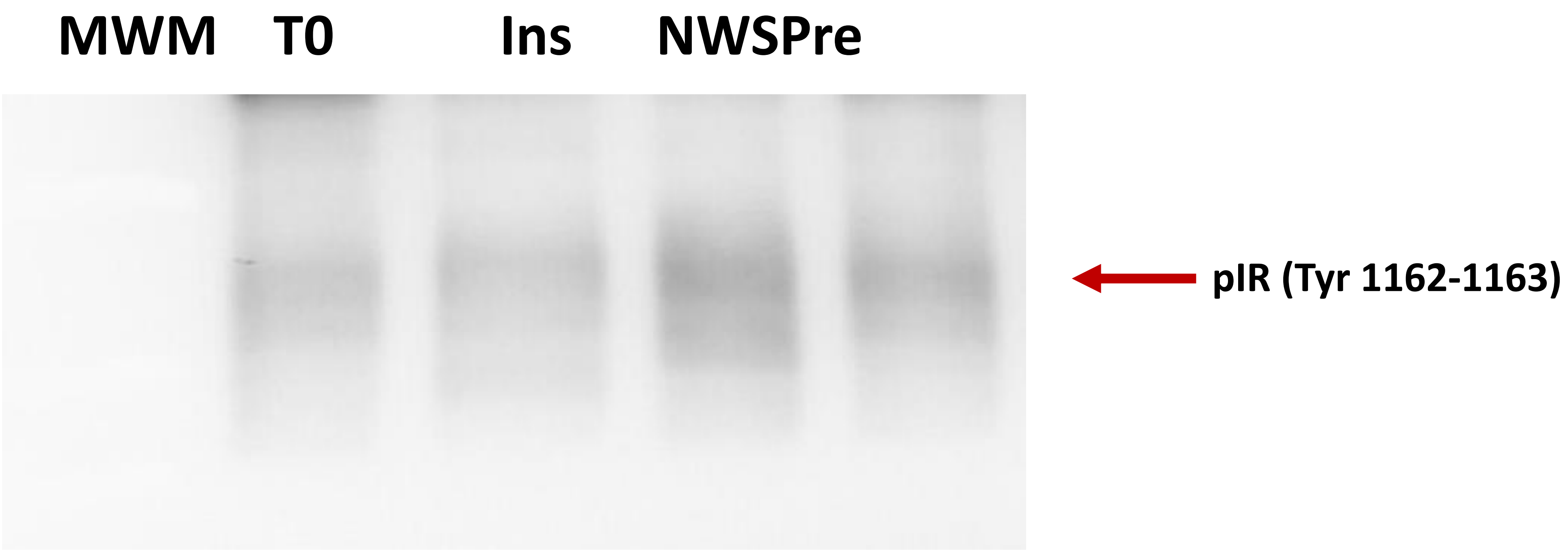

Supplementary Figure 3F

SKBR-3 IR

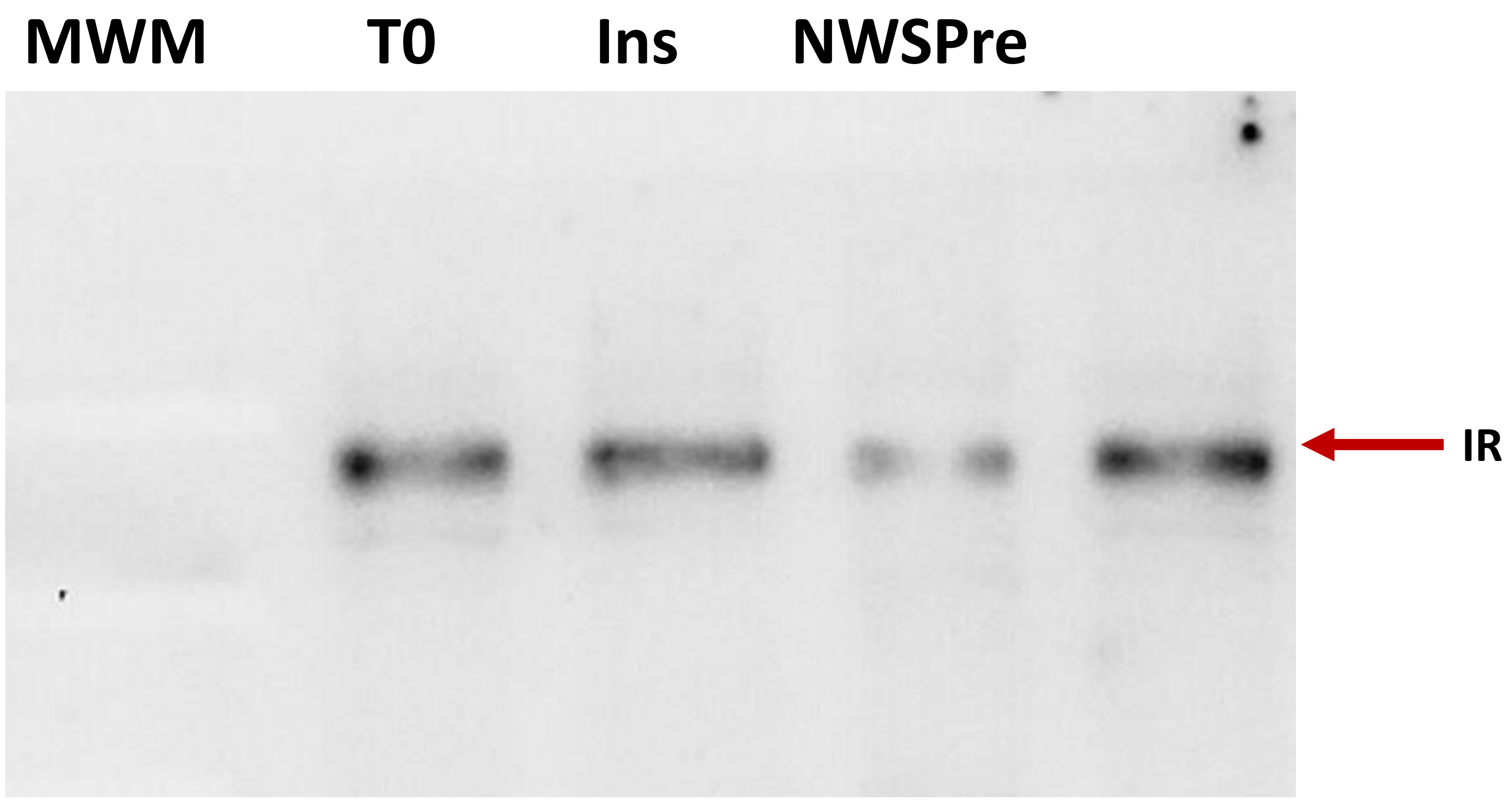

Supplementary Figure 3F

SKBR-3 pAkt(Ser 473)

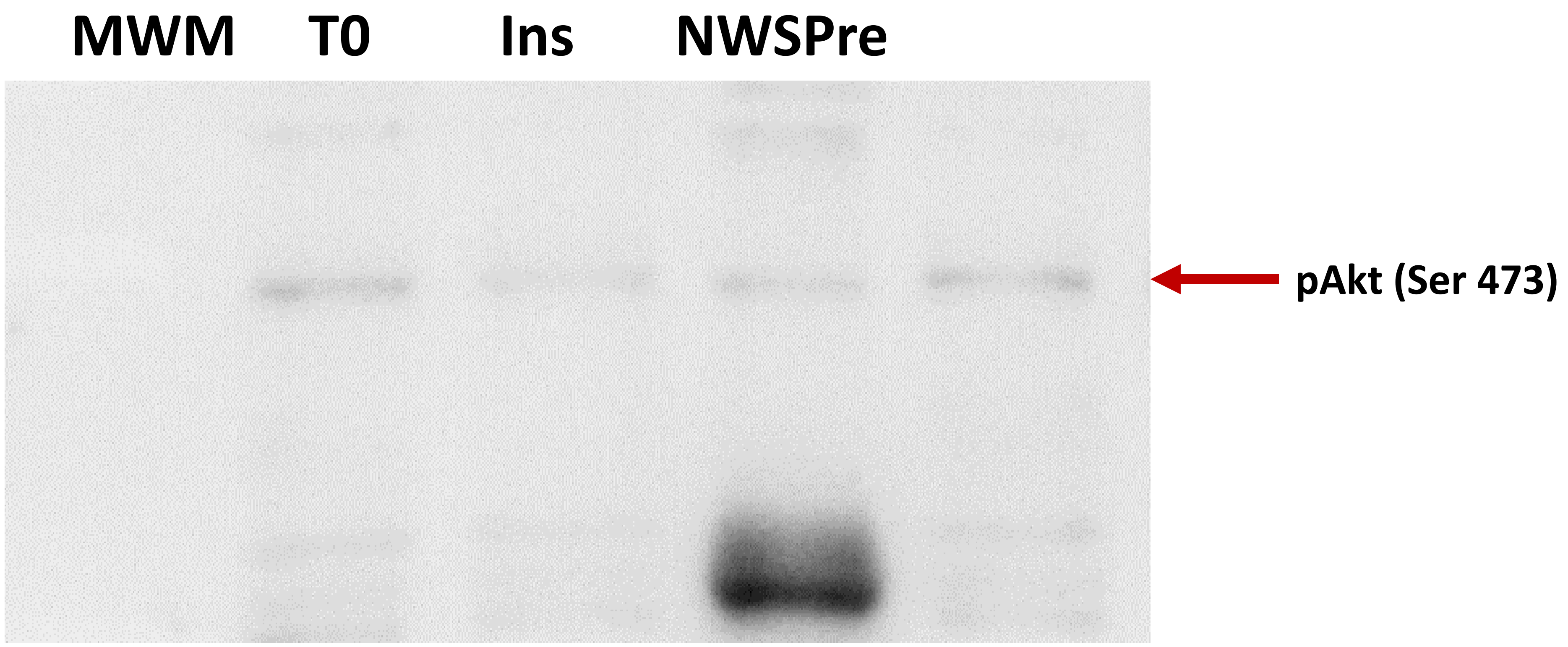

Supplementary Figure 3F

SKBR-3 Akt

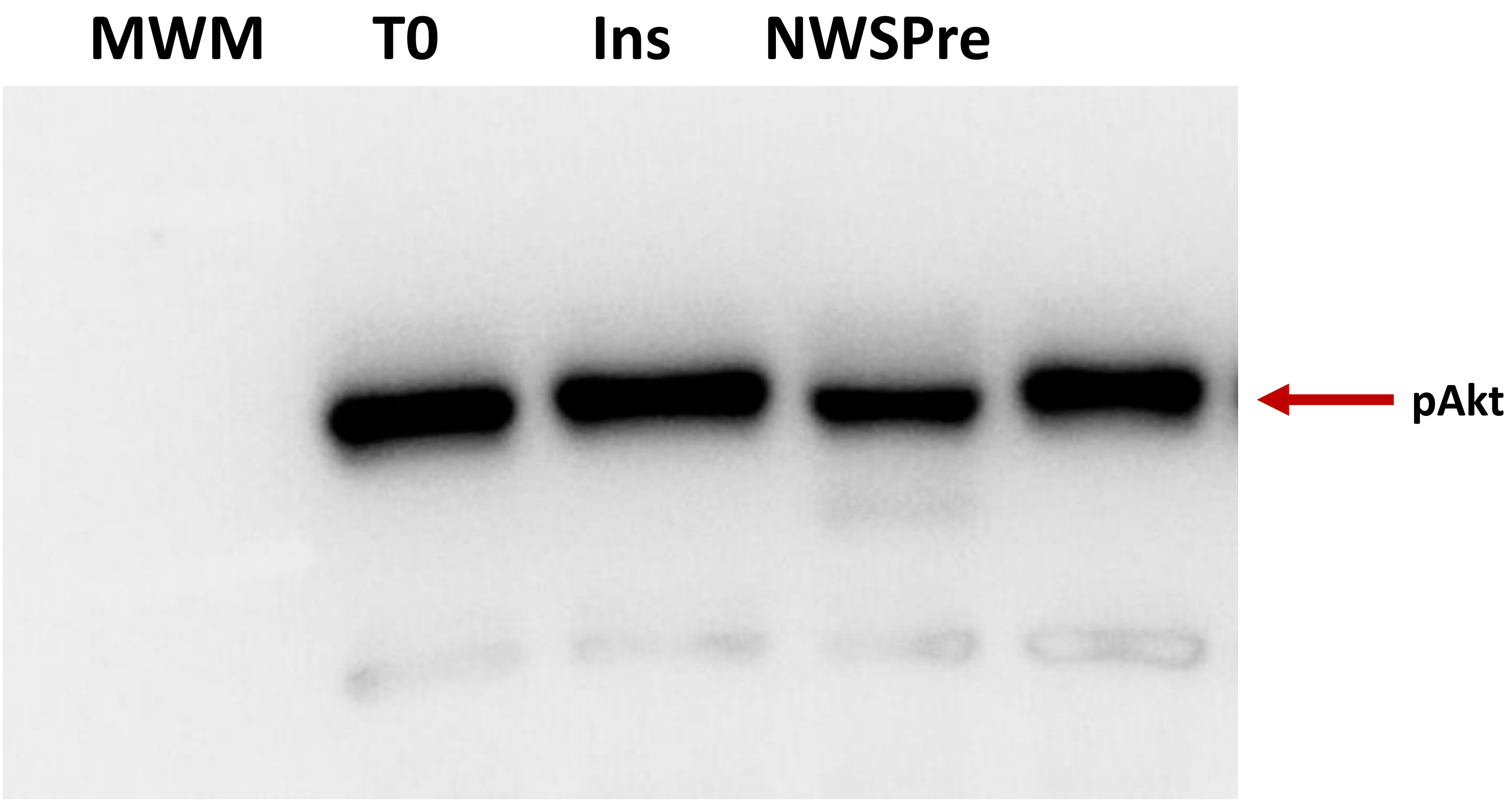

Supplementary Figure 3F

SKBR-3 p70S6K

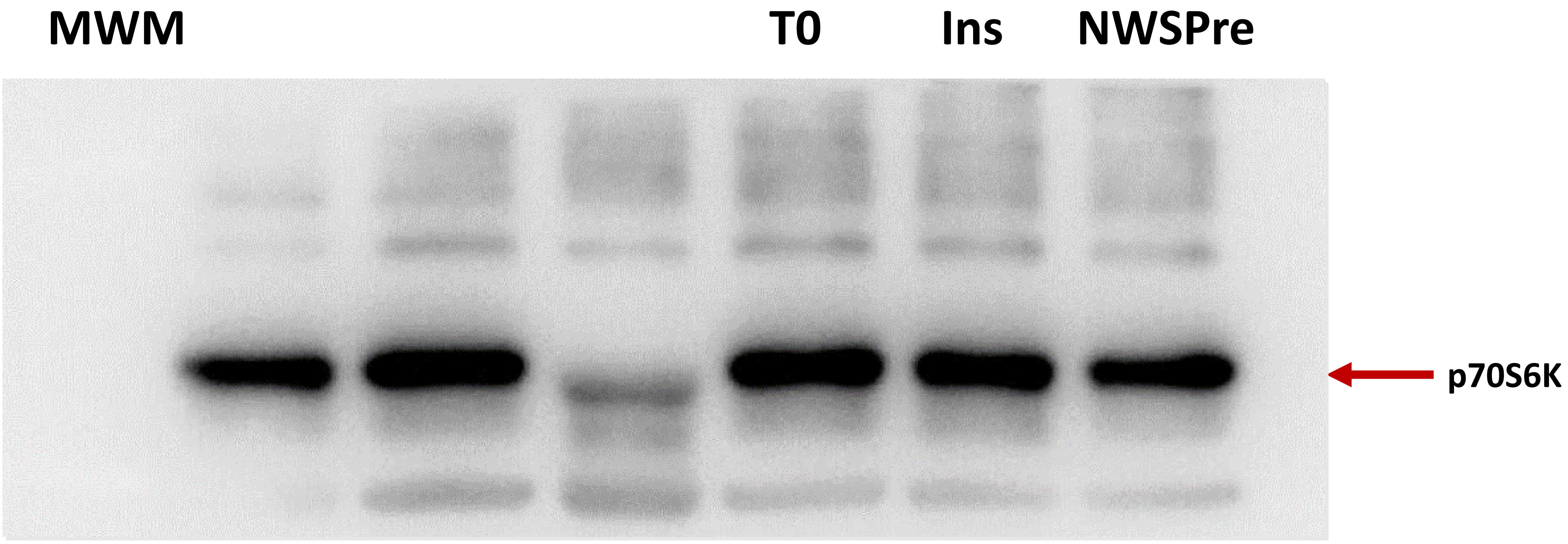

Supplementary Figure 3F

SKBR-3 p70S6K(Thr 389)

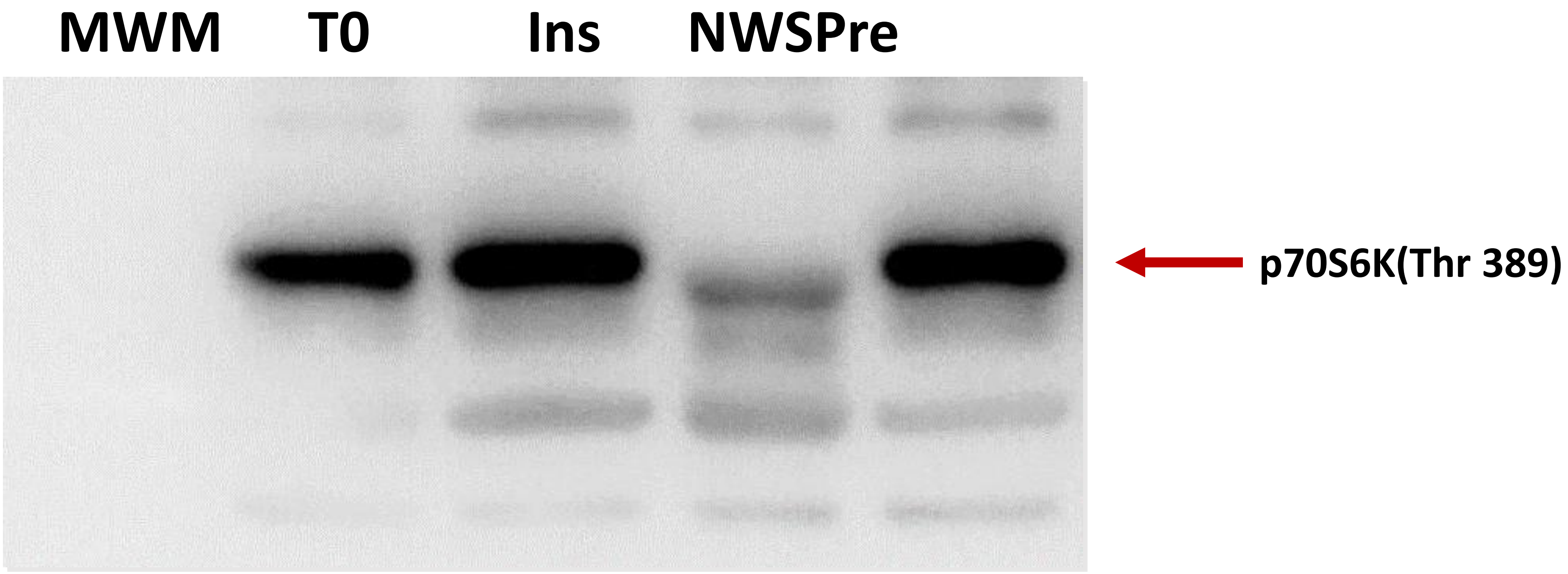

Supplementary Figure 3F

SKBR-3 actin

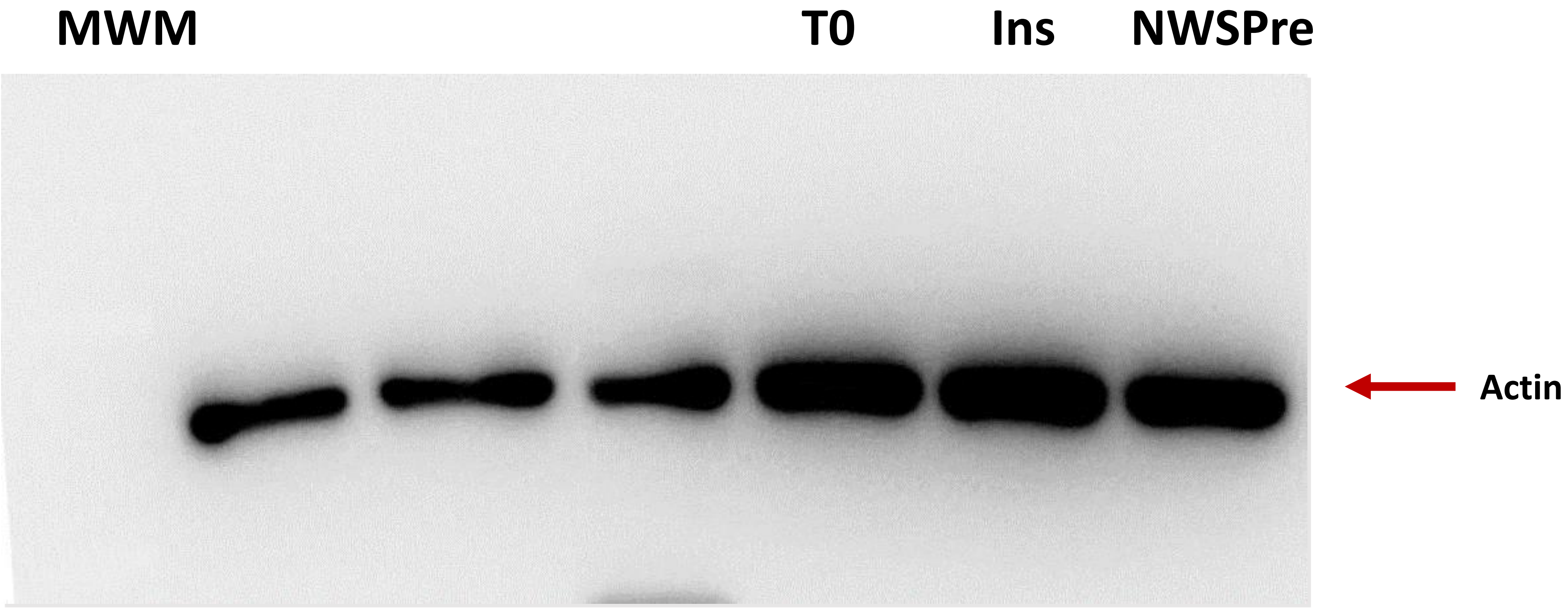

Supplementary Figure 3G

SKBR-3R pIR

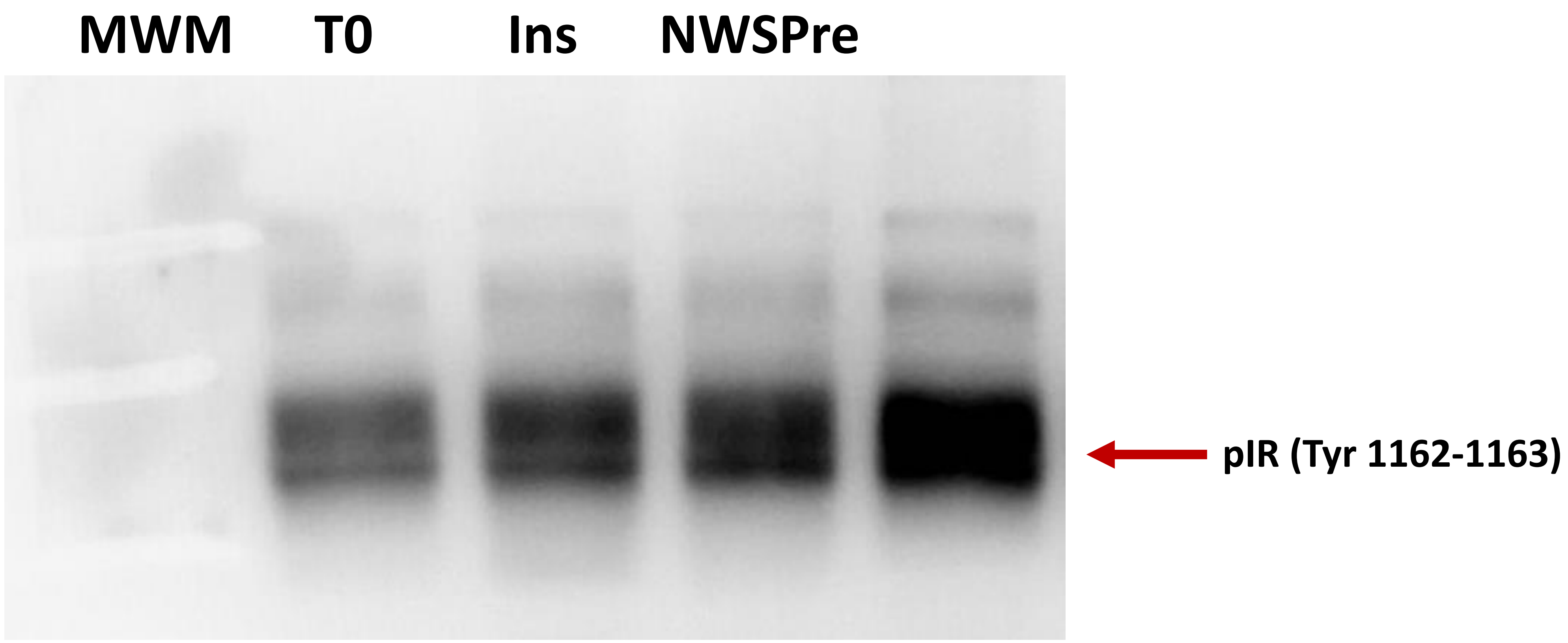

Supplementary Figure 3G

SKBR-3R IR

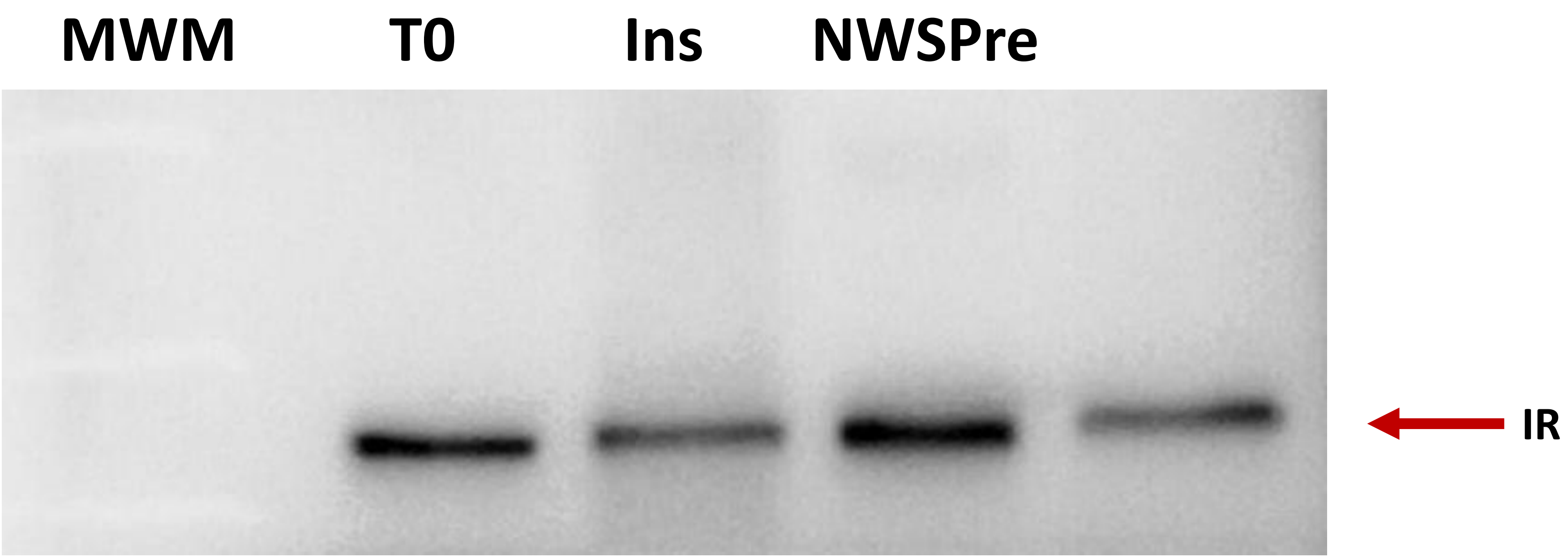

Supplementary Figure 3G

SKBR-3R pAkt(Ser 473)

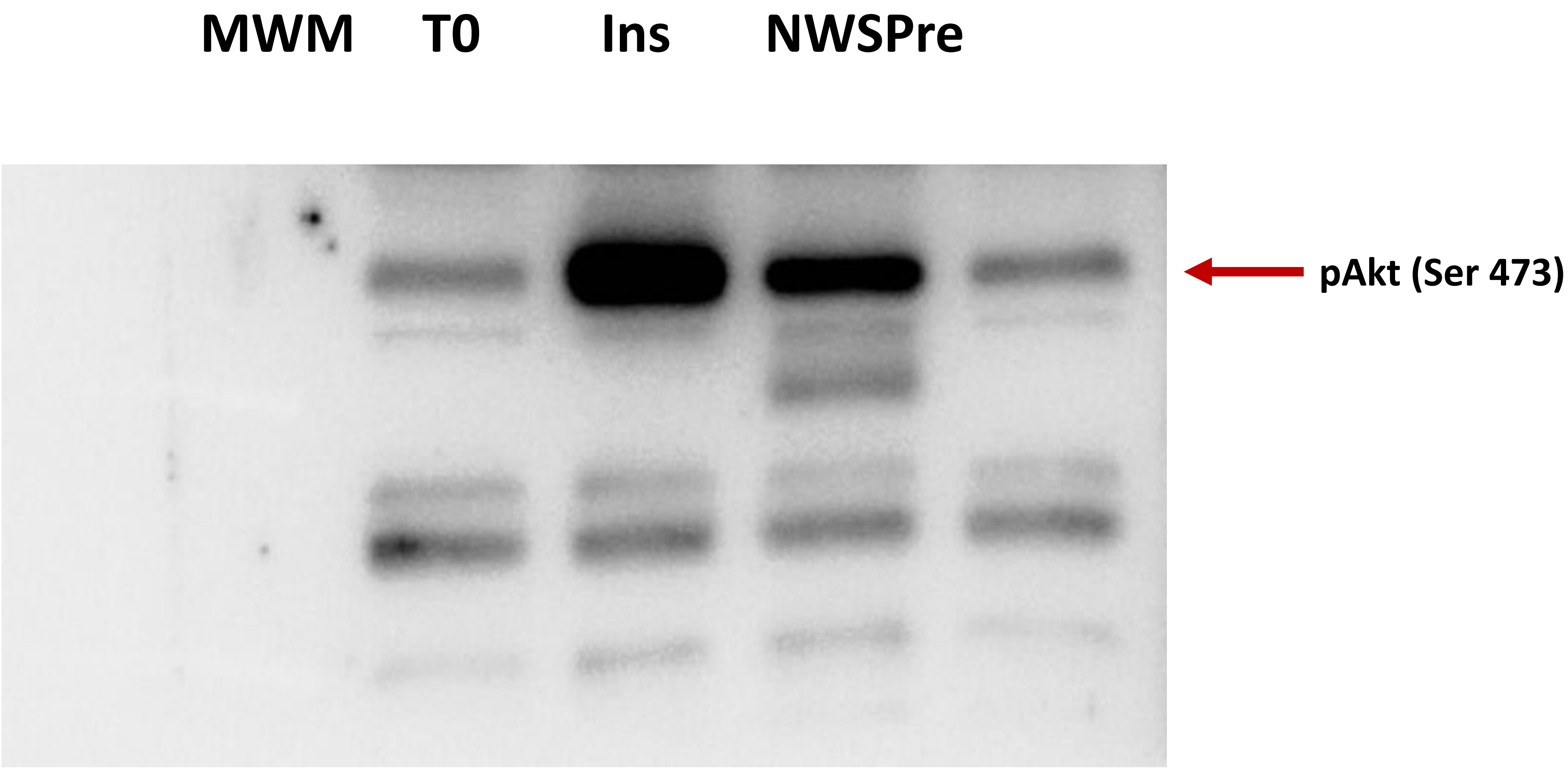

Supplementary Figure 3G

SKBR-3R Akt

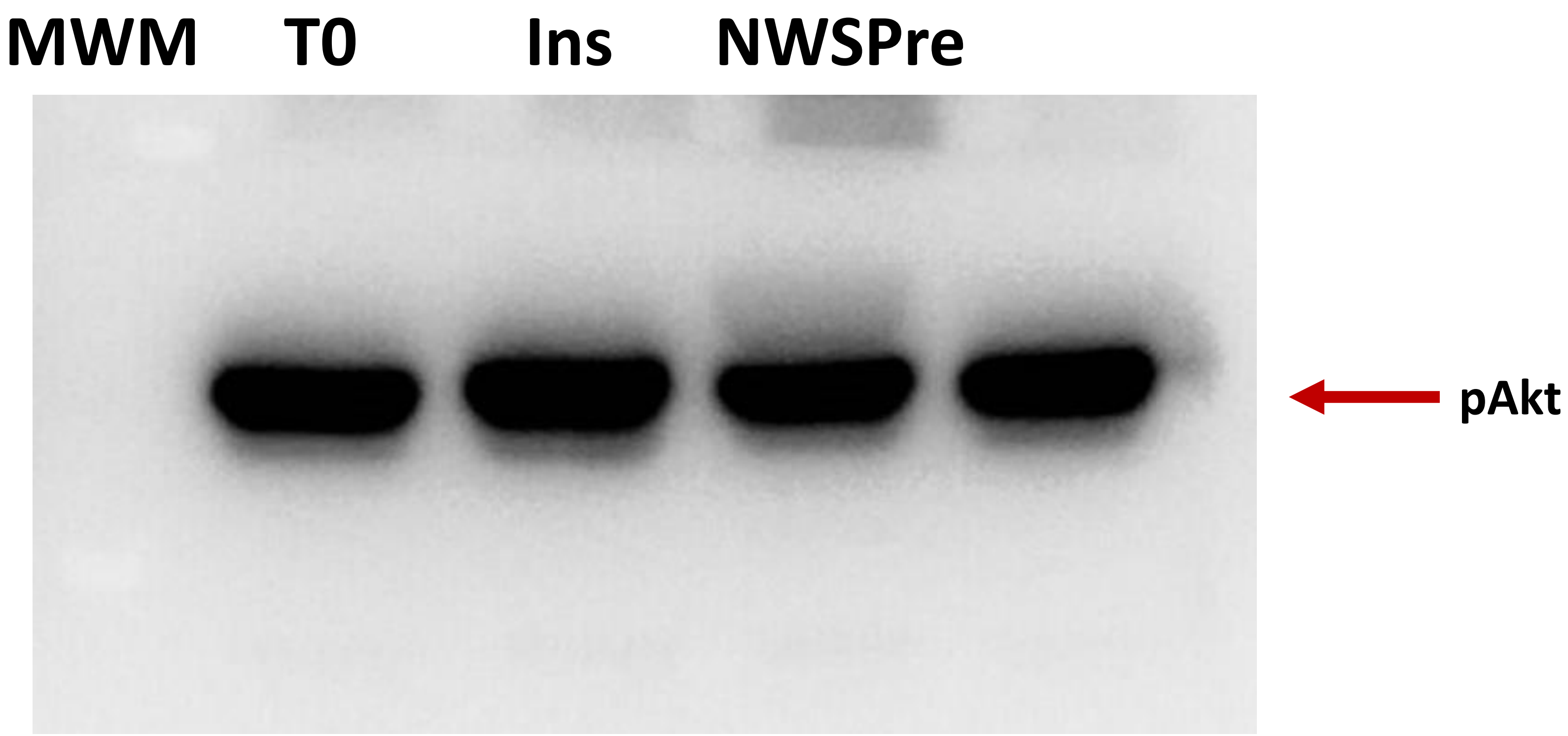

Supplementary Figure 3G

SKBR-3R p70S6K

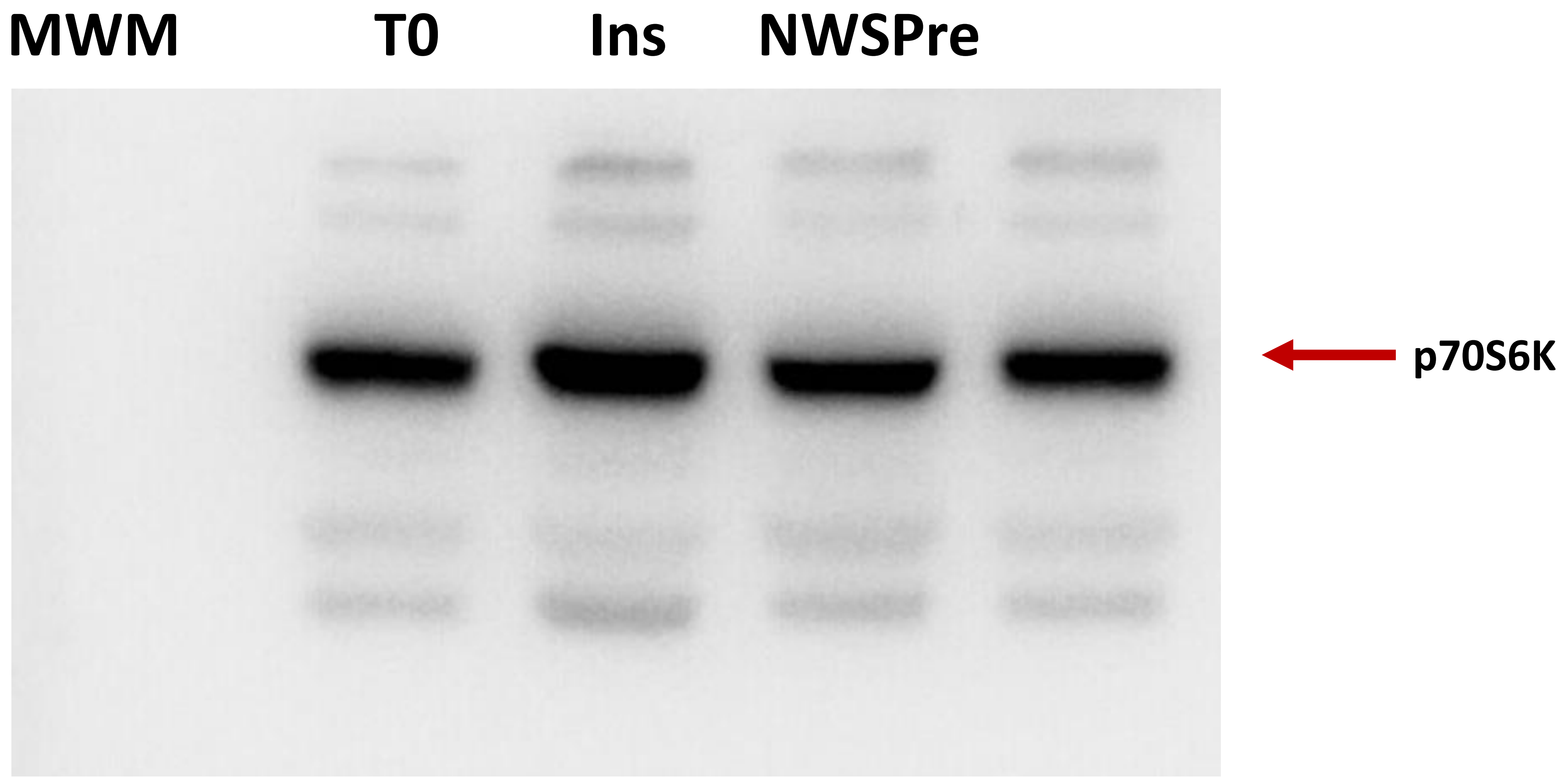

Supplementary Figure 3G

SKBR-3R p70S6K(Thr 389)

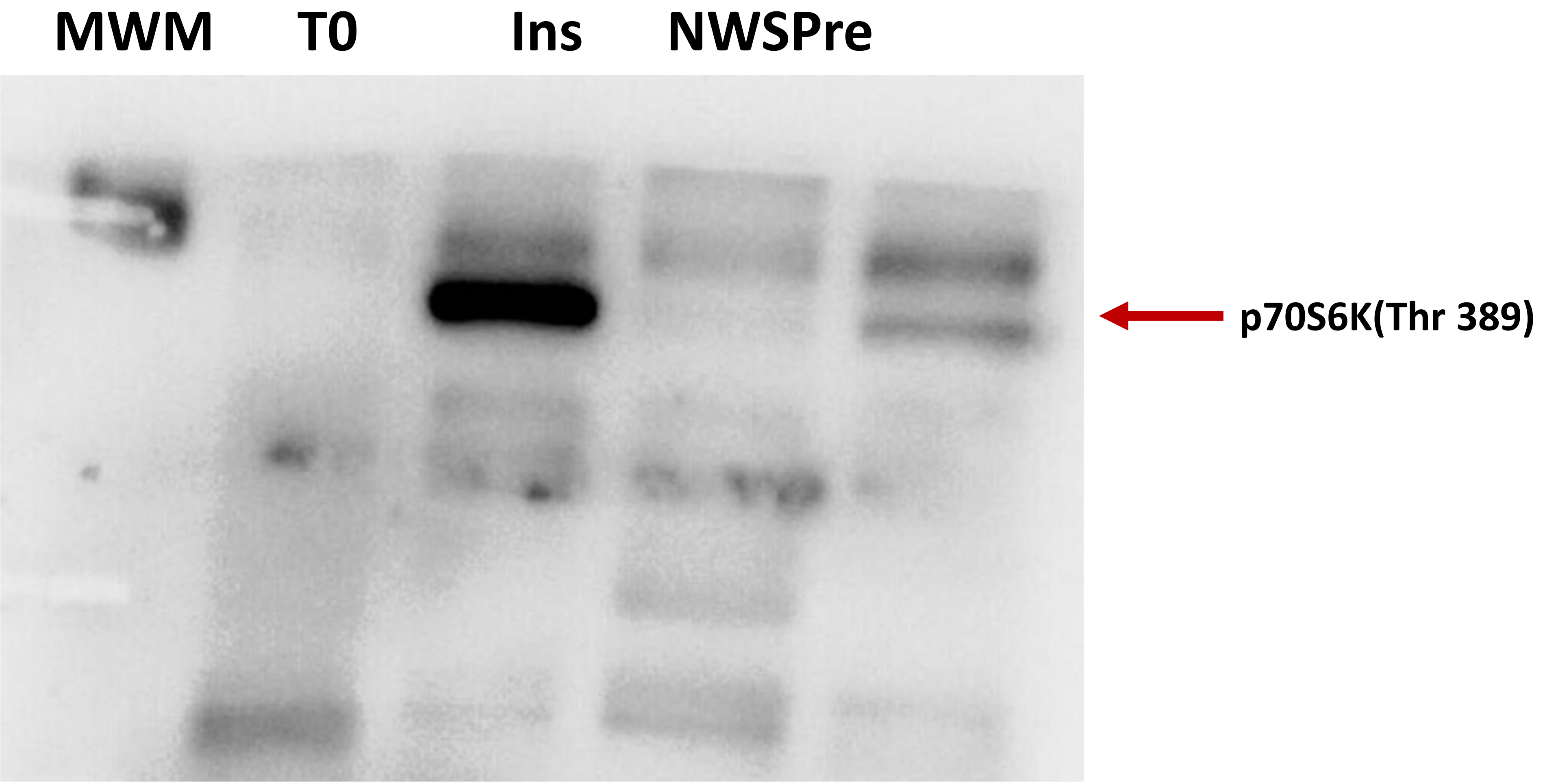

Supplementary Figure 3G

SKBR-3R actin

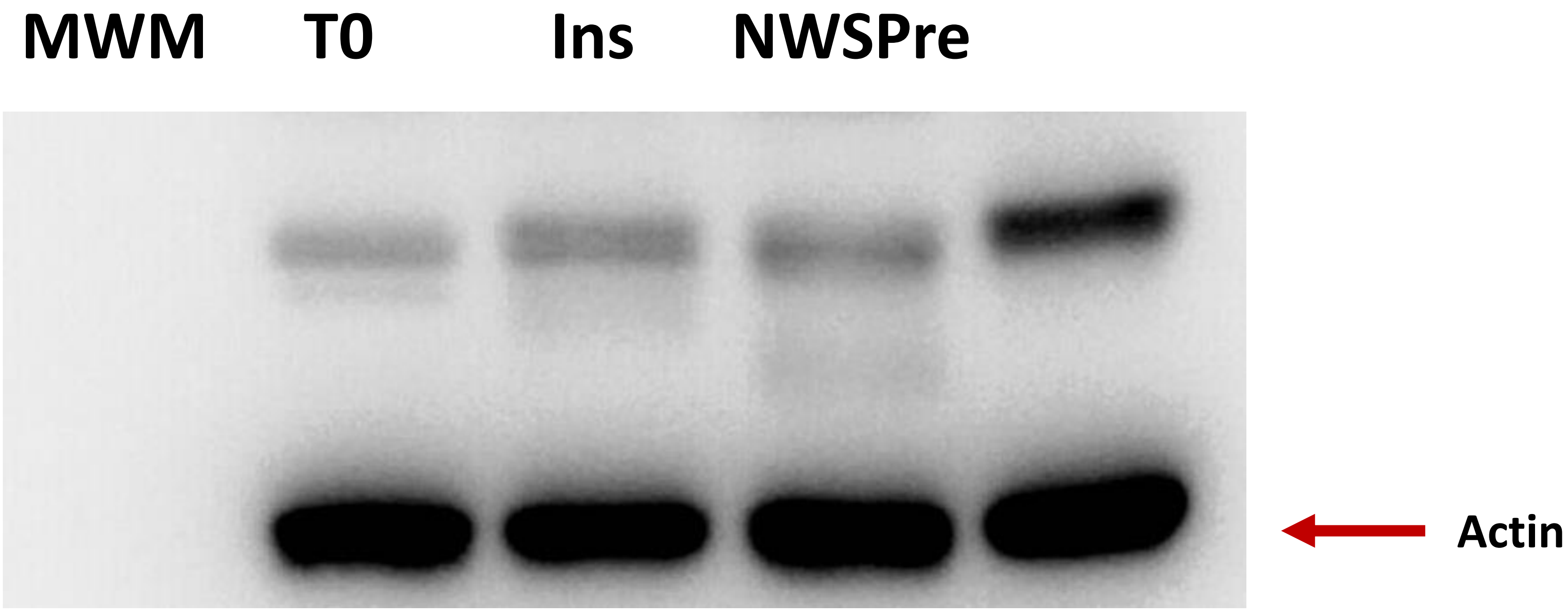

Supplementary Figure 3H

MDA-MB-231 pIR

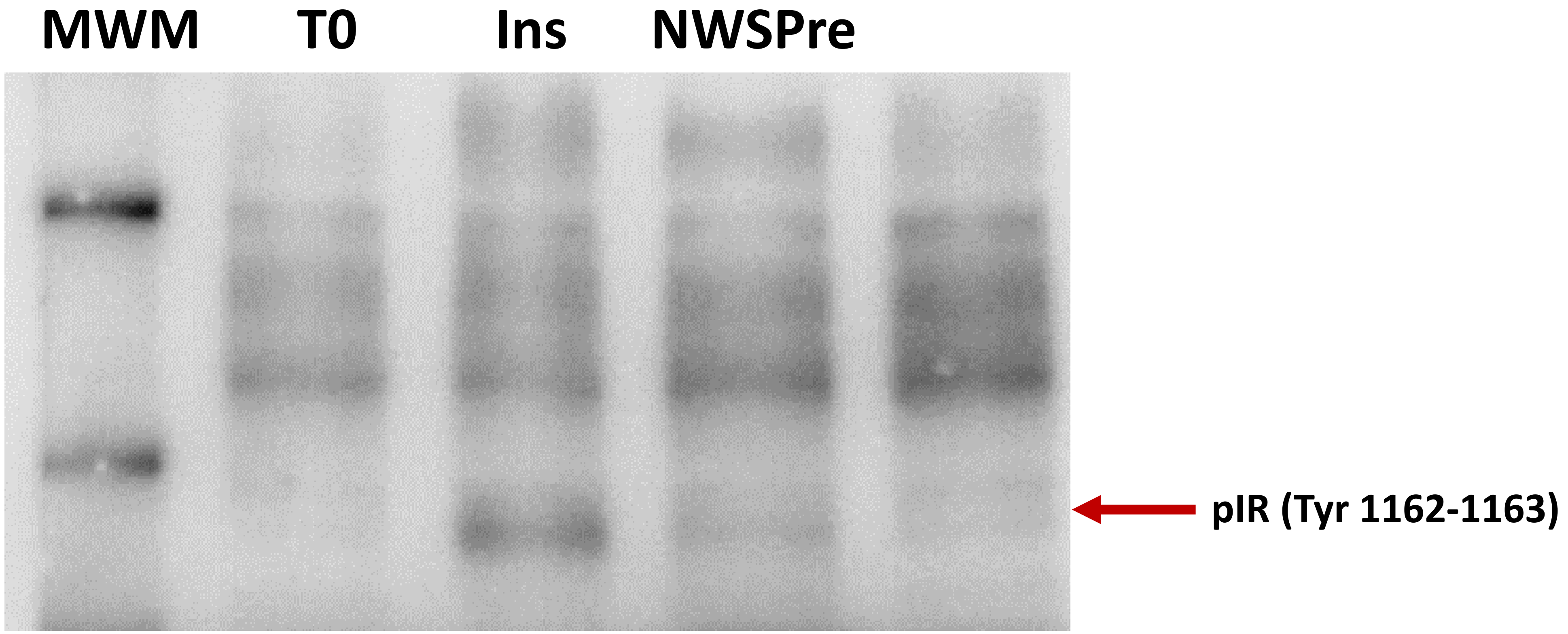

Supplementary Figure 3H

MDA-MB-231 IR

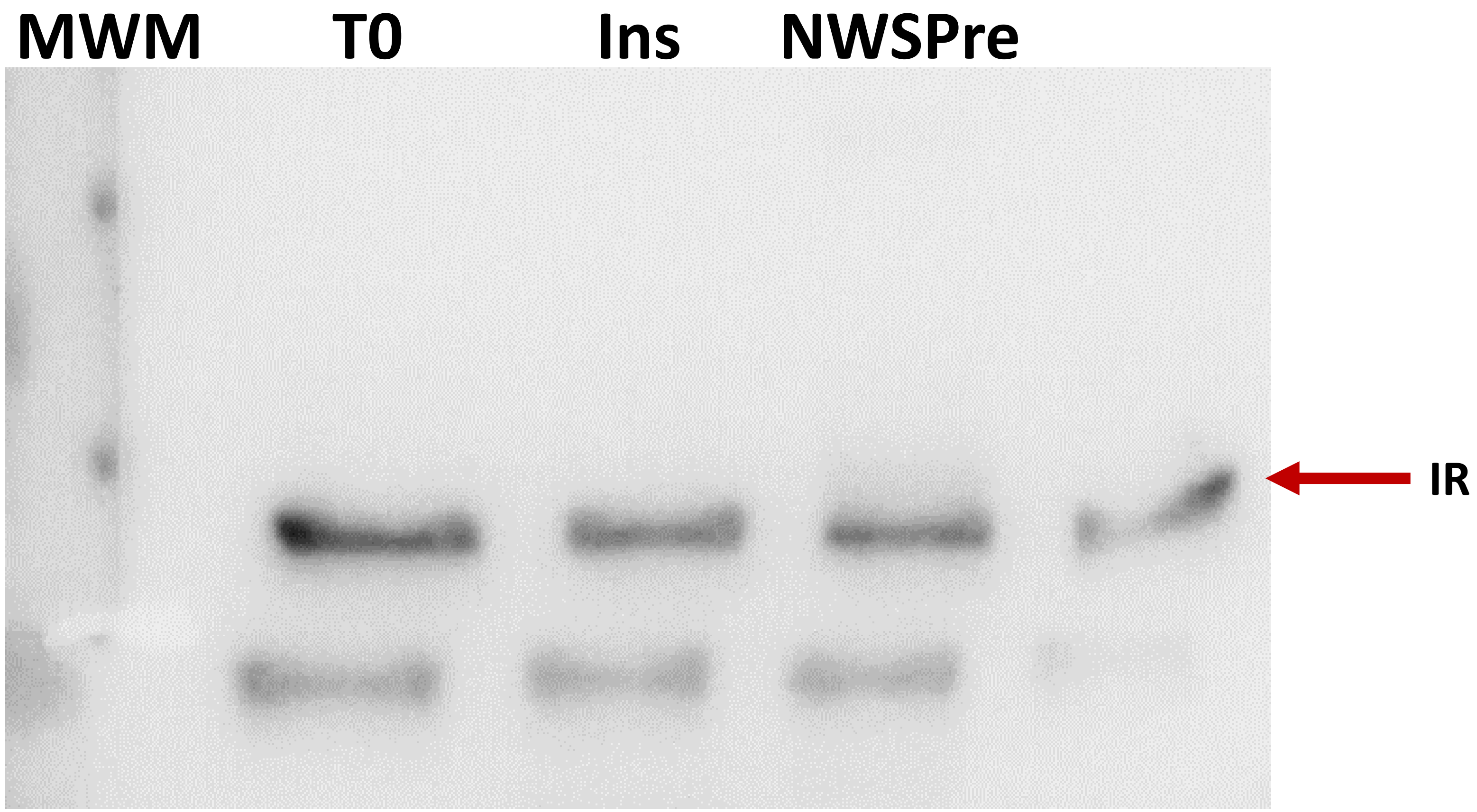

Supplementary Figure 3H

MDA-MB-231 pAkt(Ser 473)

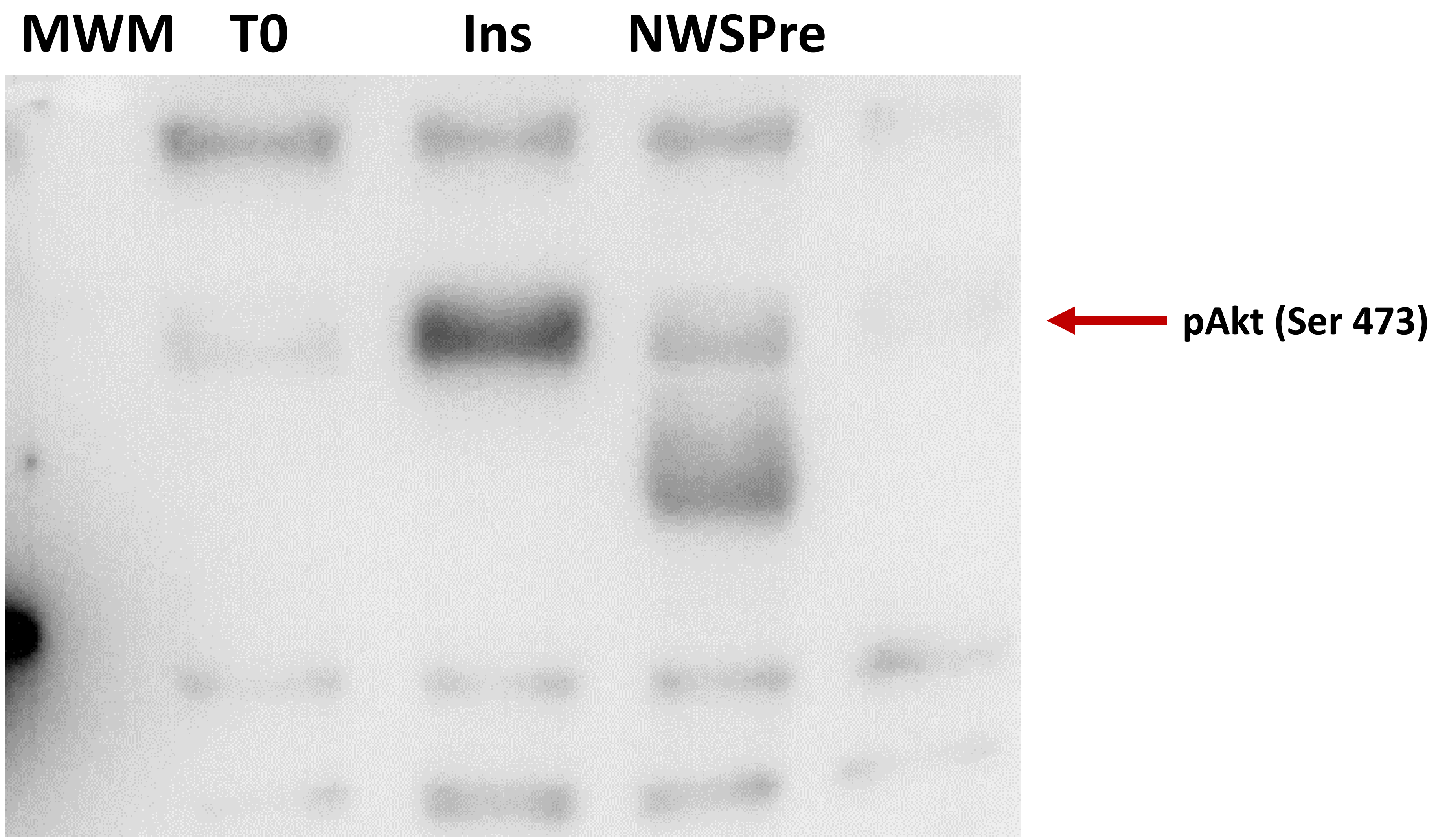

Supplementary Figure 3H

MDA-MB-231 Akt

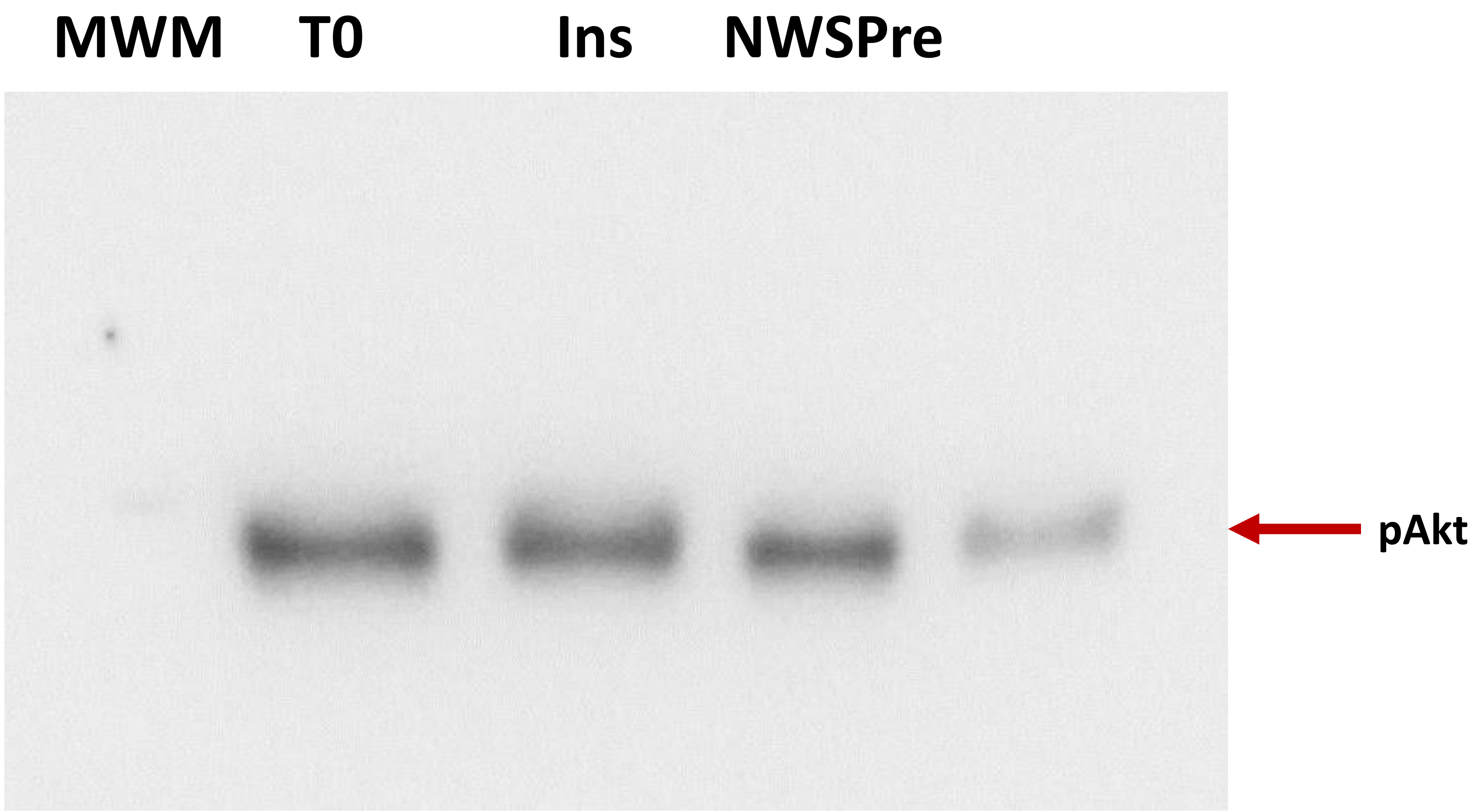

Supplementary Figure 3H

MDA-MB-231 p70S6K(Thr 389)

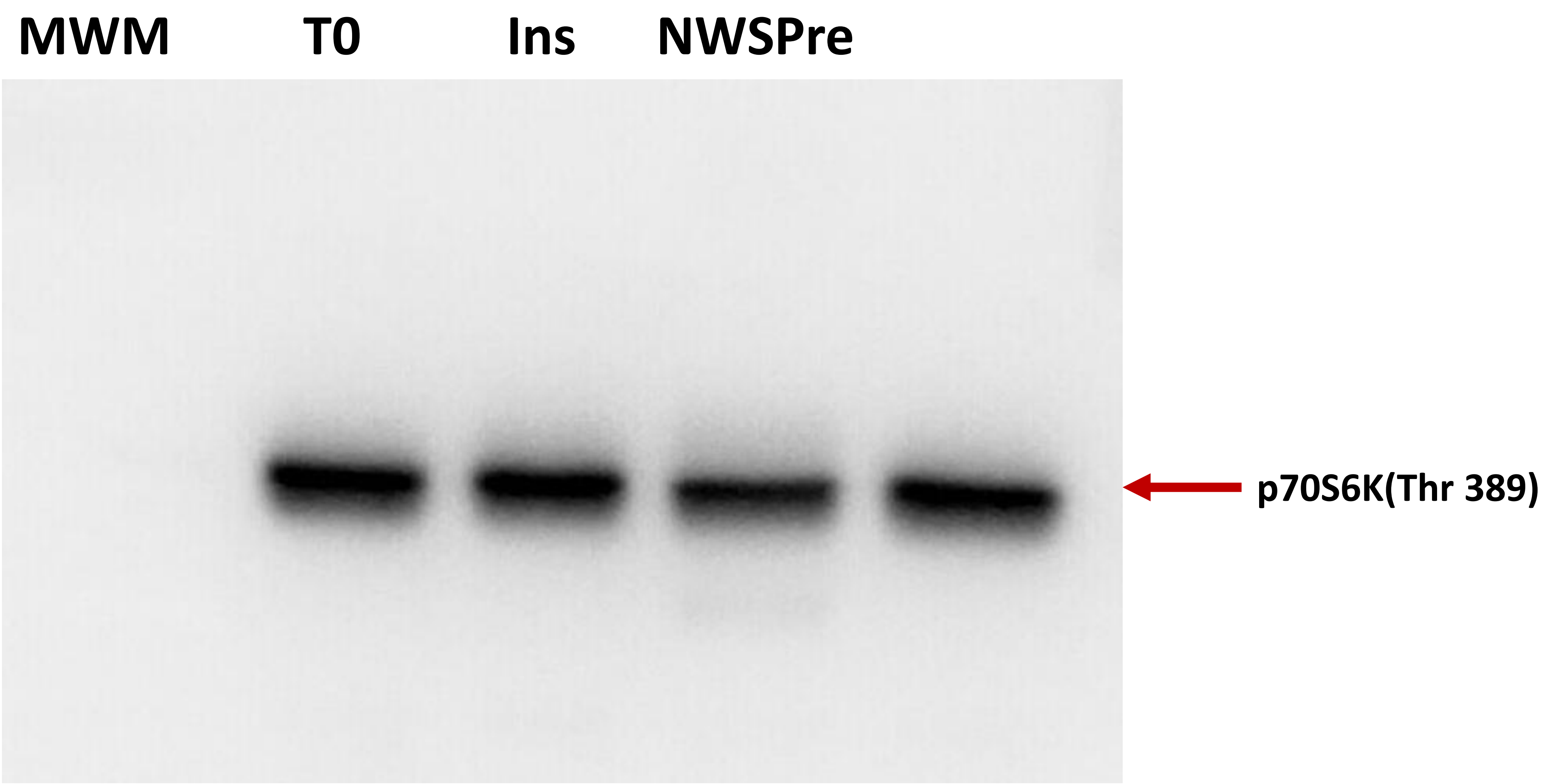

Supplementary Figure 3H

MDA-MB-231 p70S6K

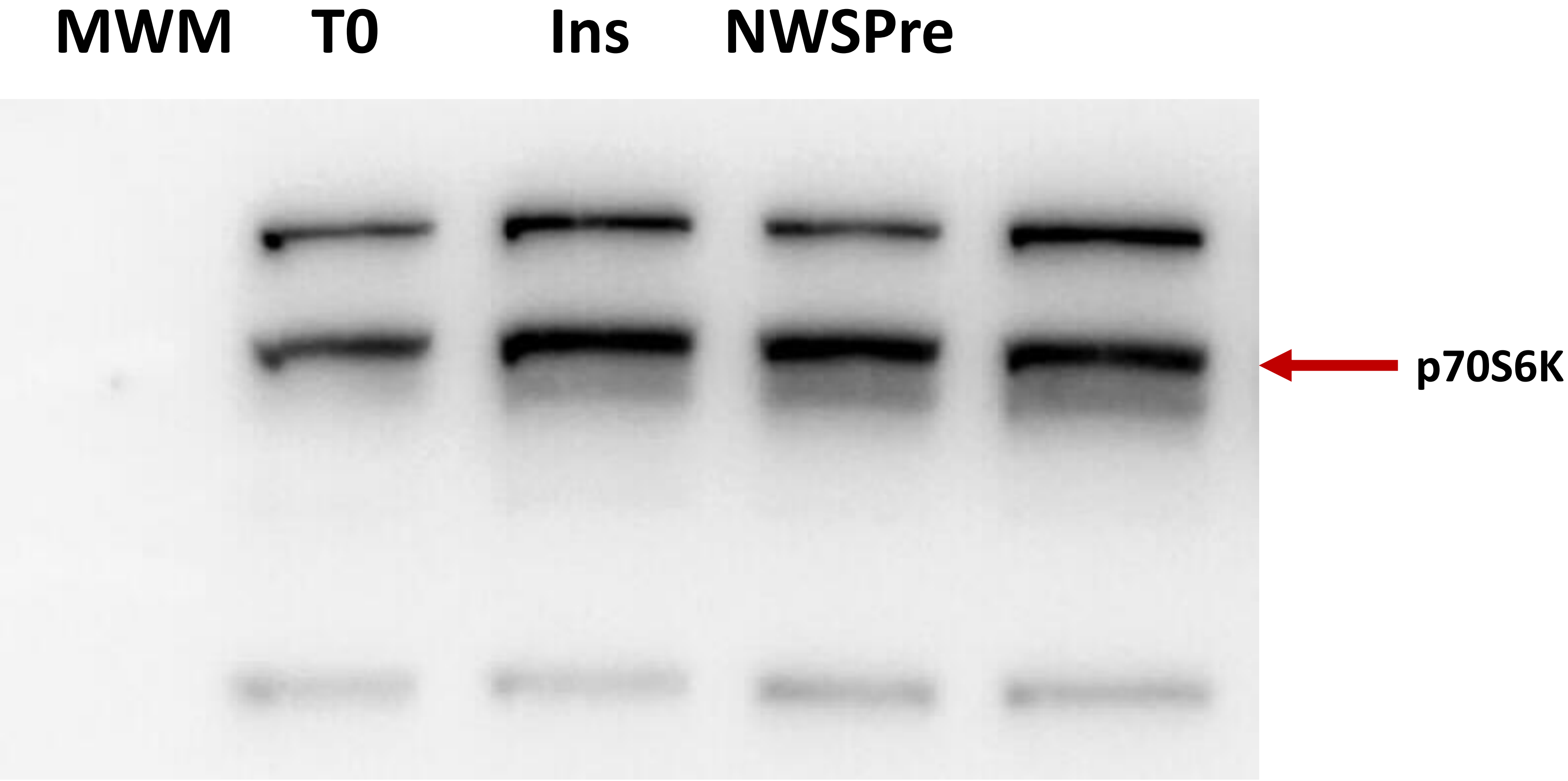

Supplementary Figure 3H

MDA-MB-231 actin

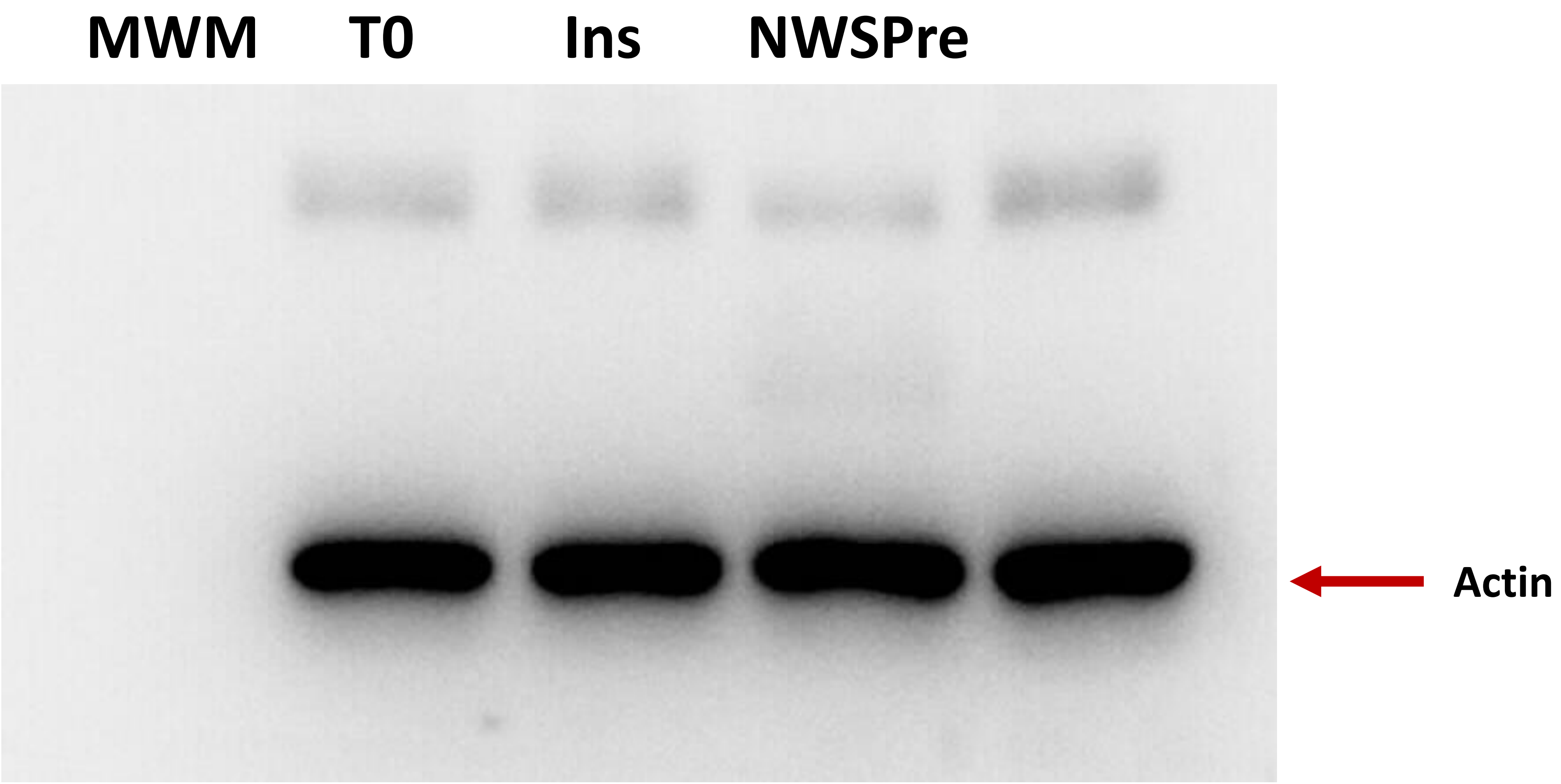

Supplementary Figure 3I

MDA-MB-468 pIR

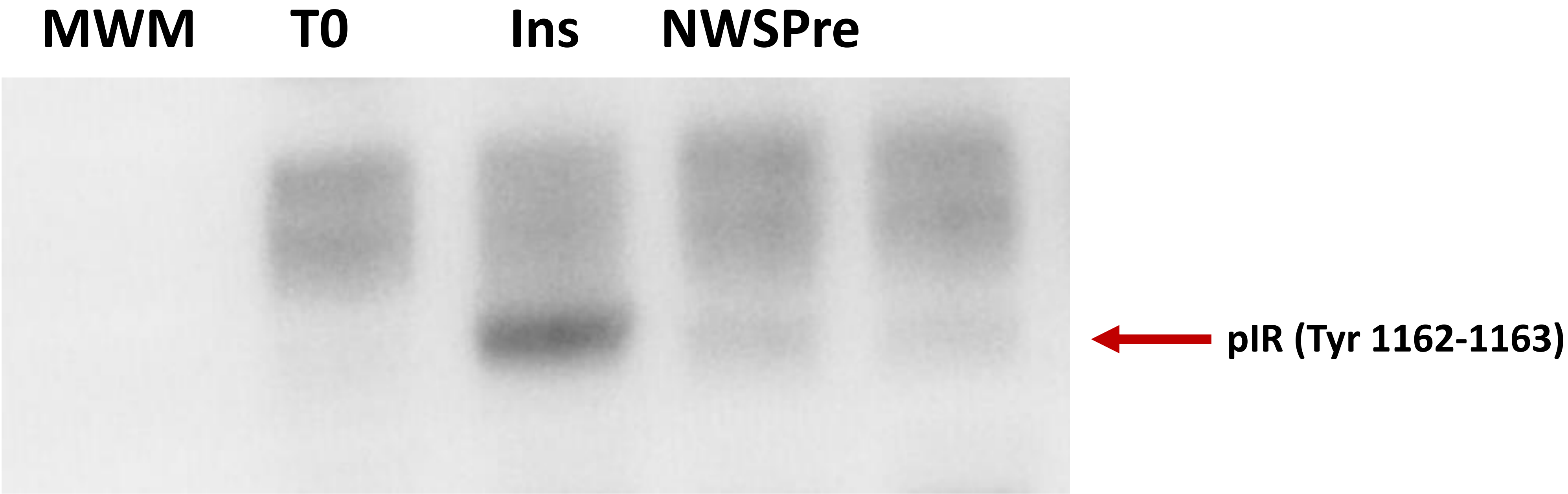

Supplementary Figure 3I

MDA-MB-468 IR

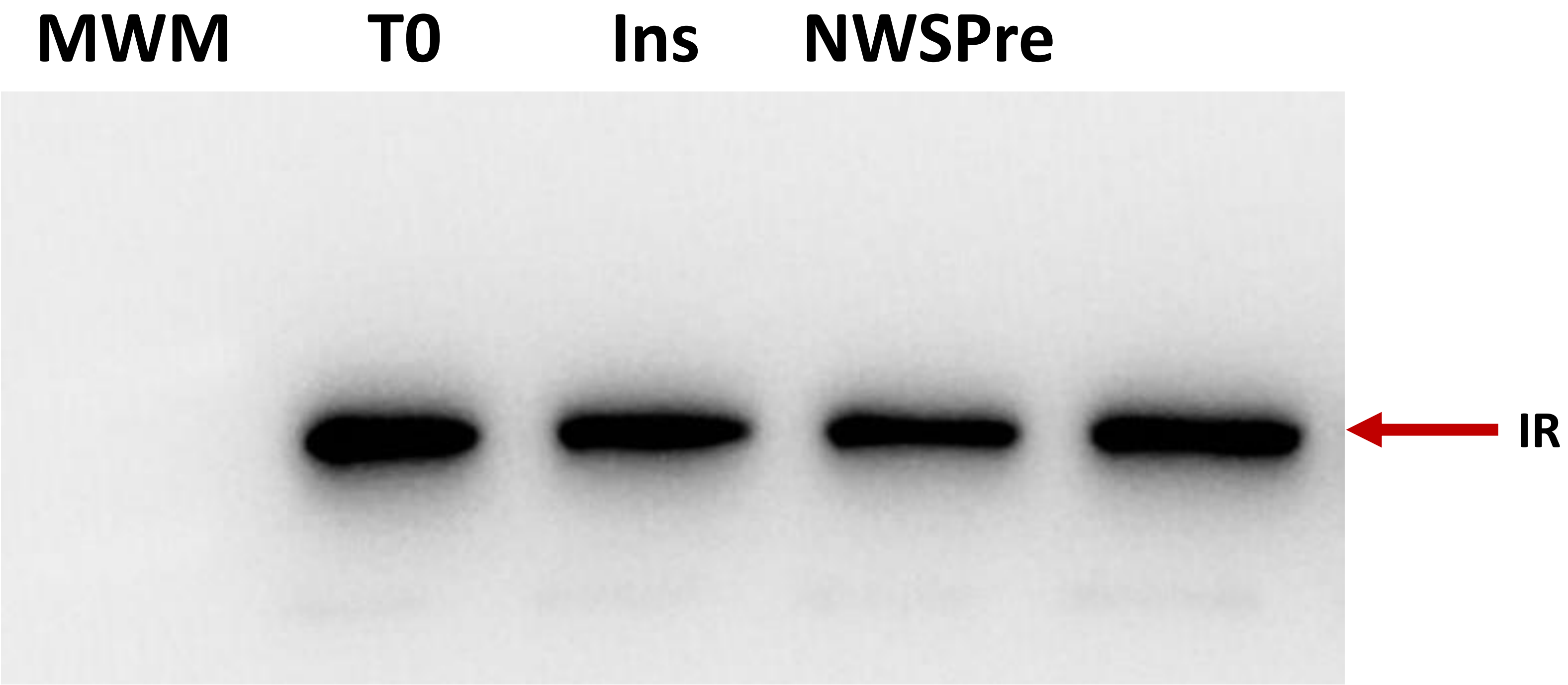

Supplementary Figure 3I

MDA-MB-468 pAkt(Ser 473)

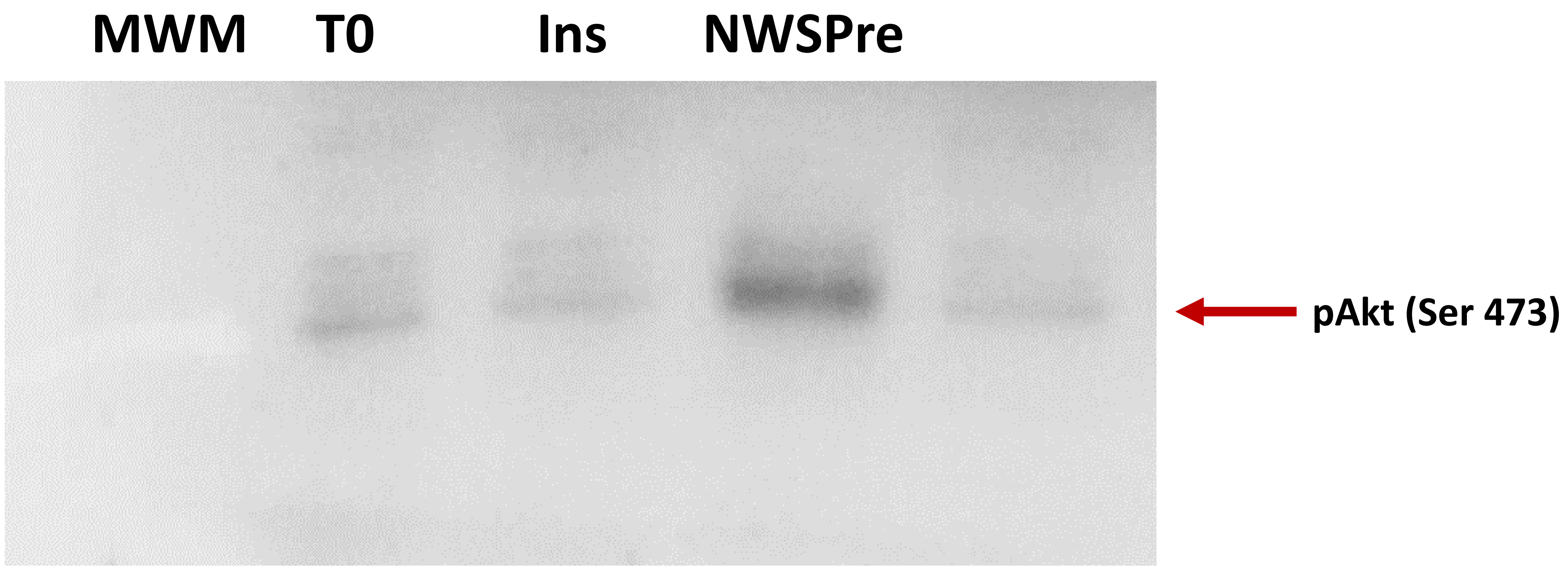

Supplementary Figure 3I

MDA-MB-468 Akt

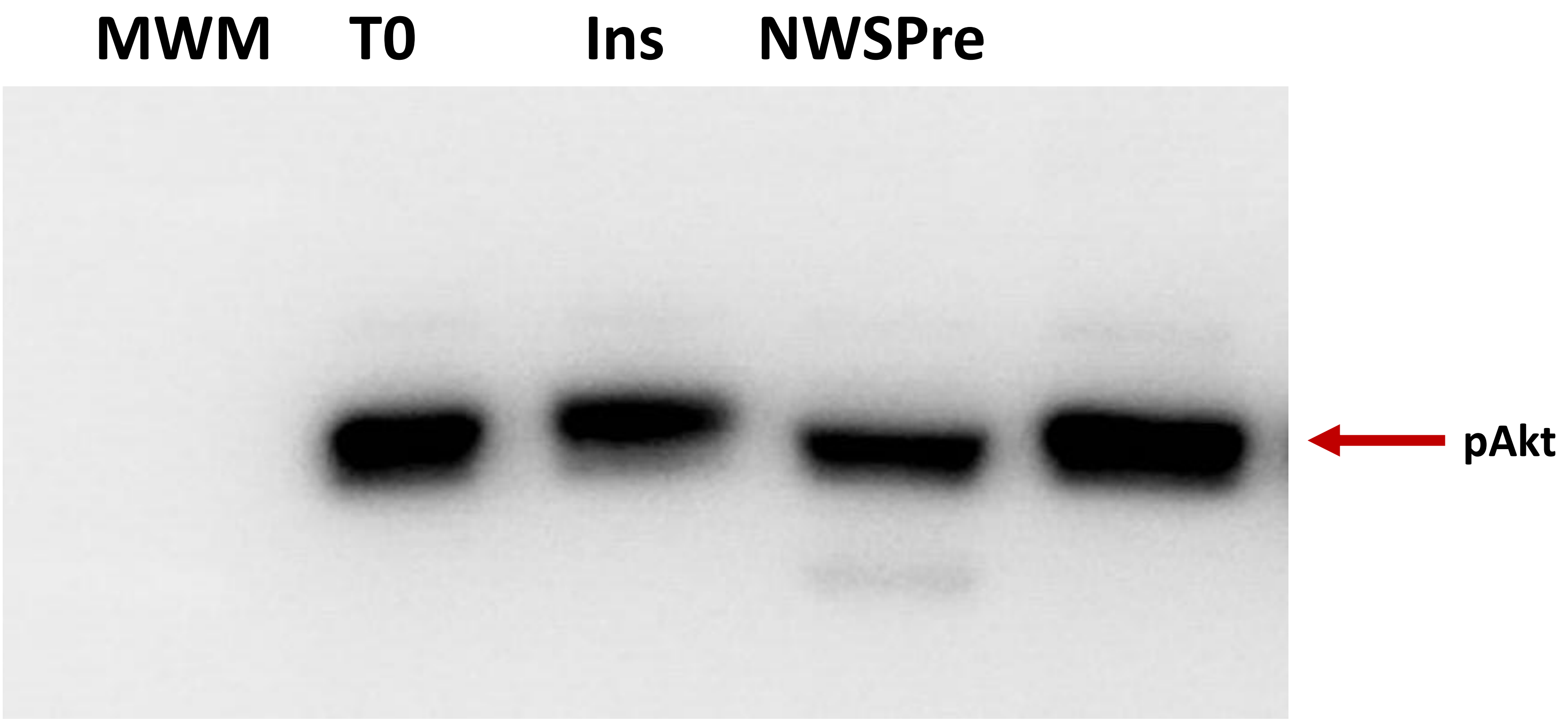

Supplementary Figure 3I

MDA-MB-468 p70S6K(Thr 389)

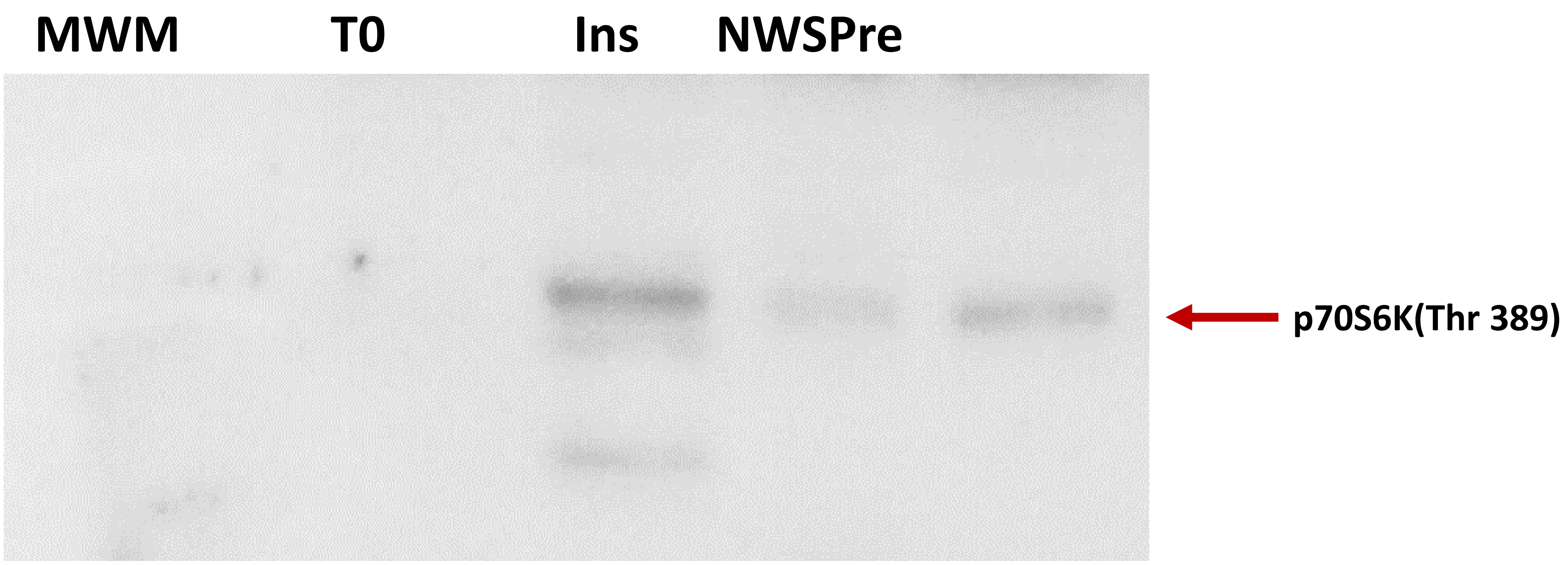

Supplementary Figure 3I

MDA-MB-468 p70S6K

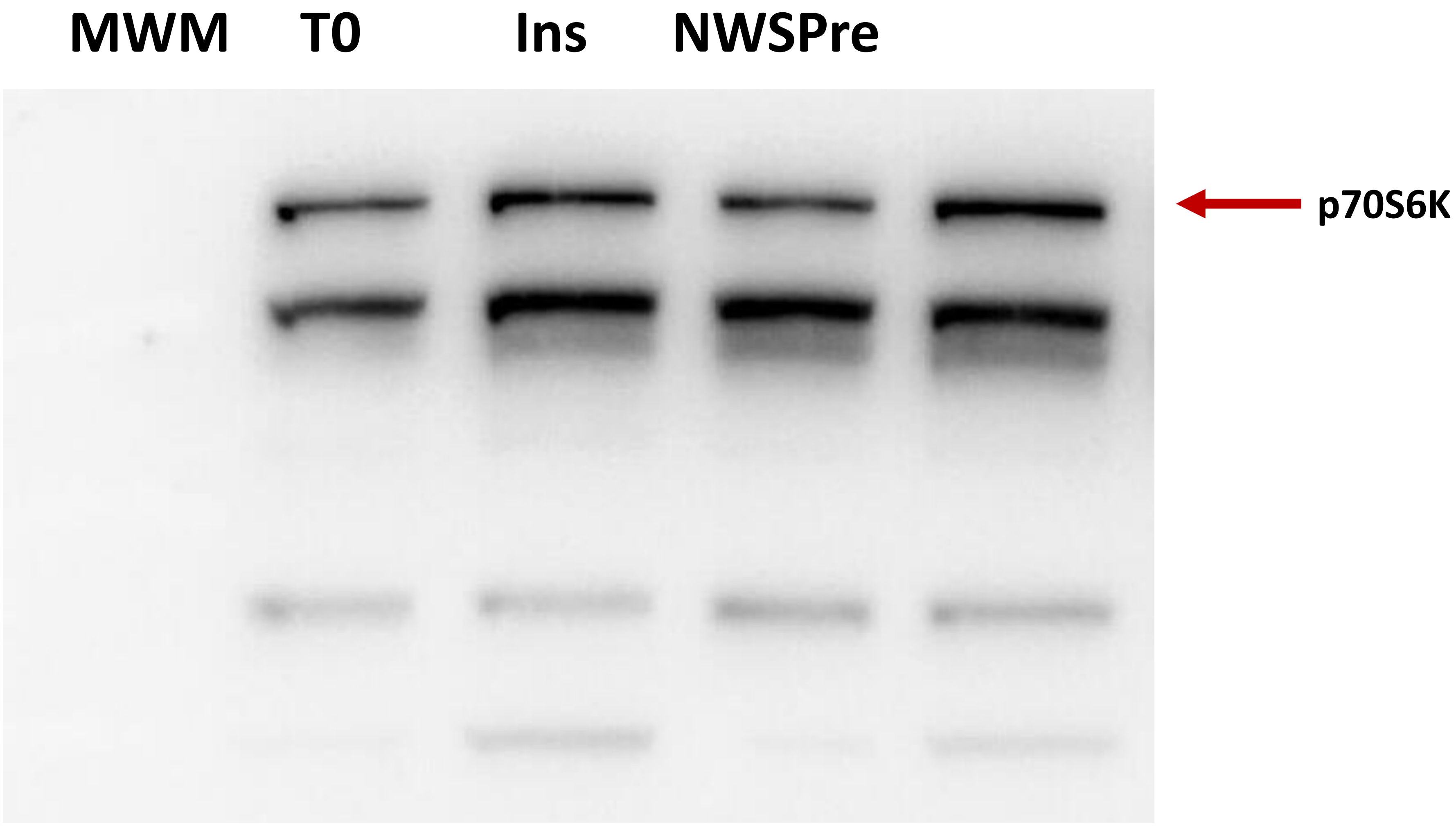

Supplementary Figure 3I

MDA-MB-468 actin

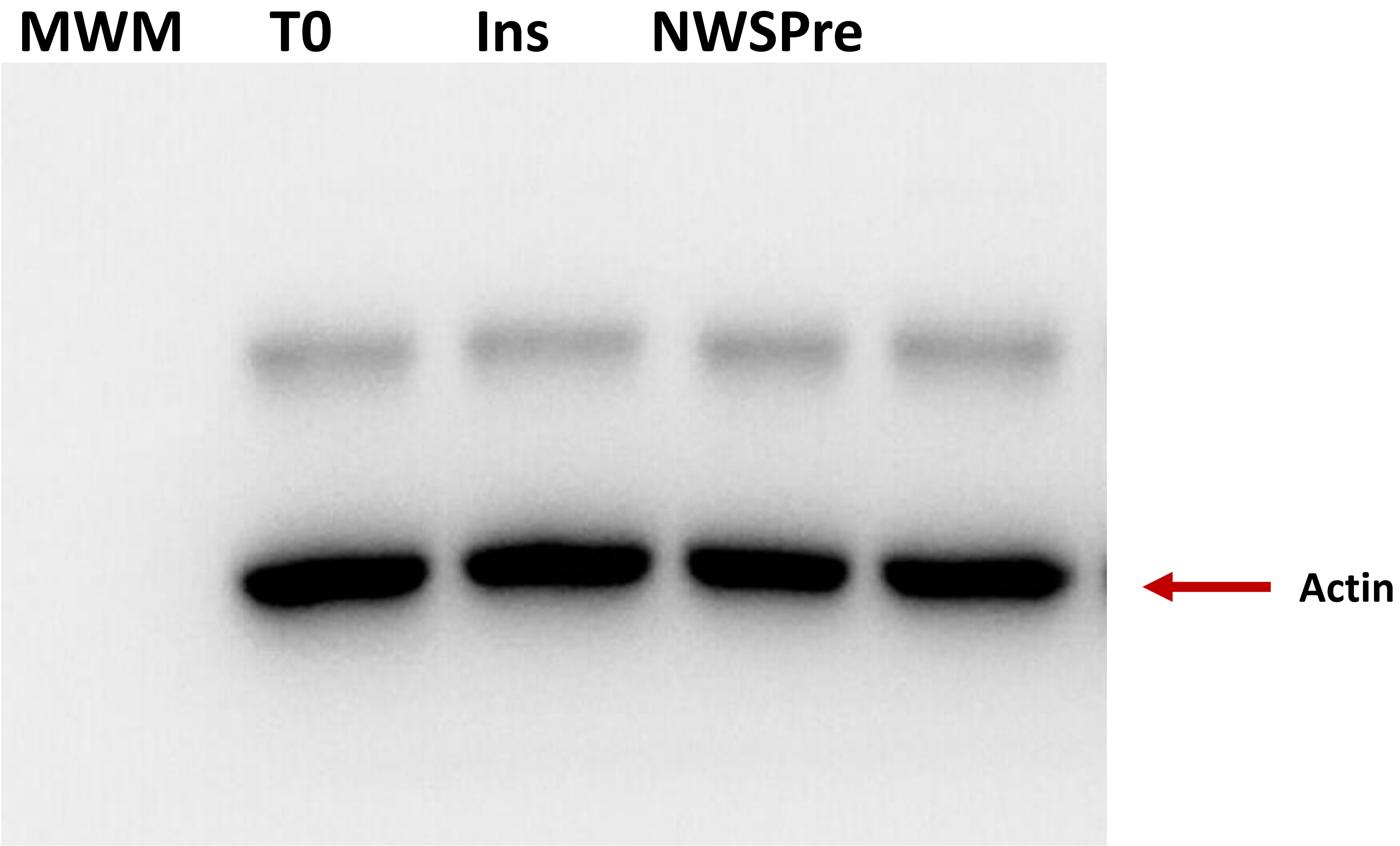

Supplementary Figure 5

Akt-1 mRNA NWSPre

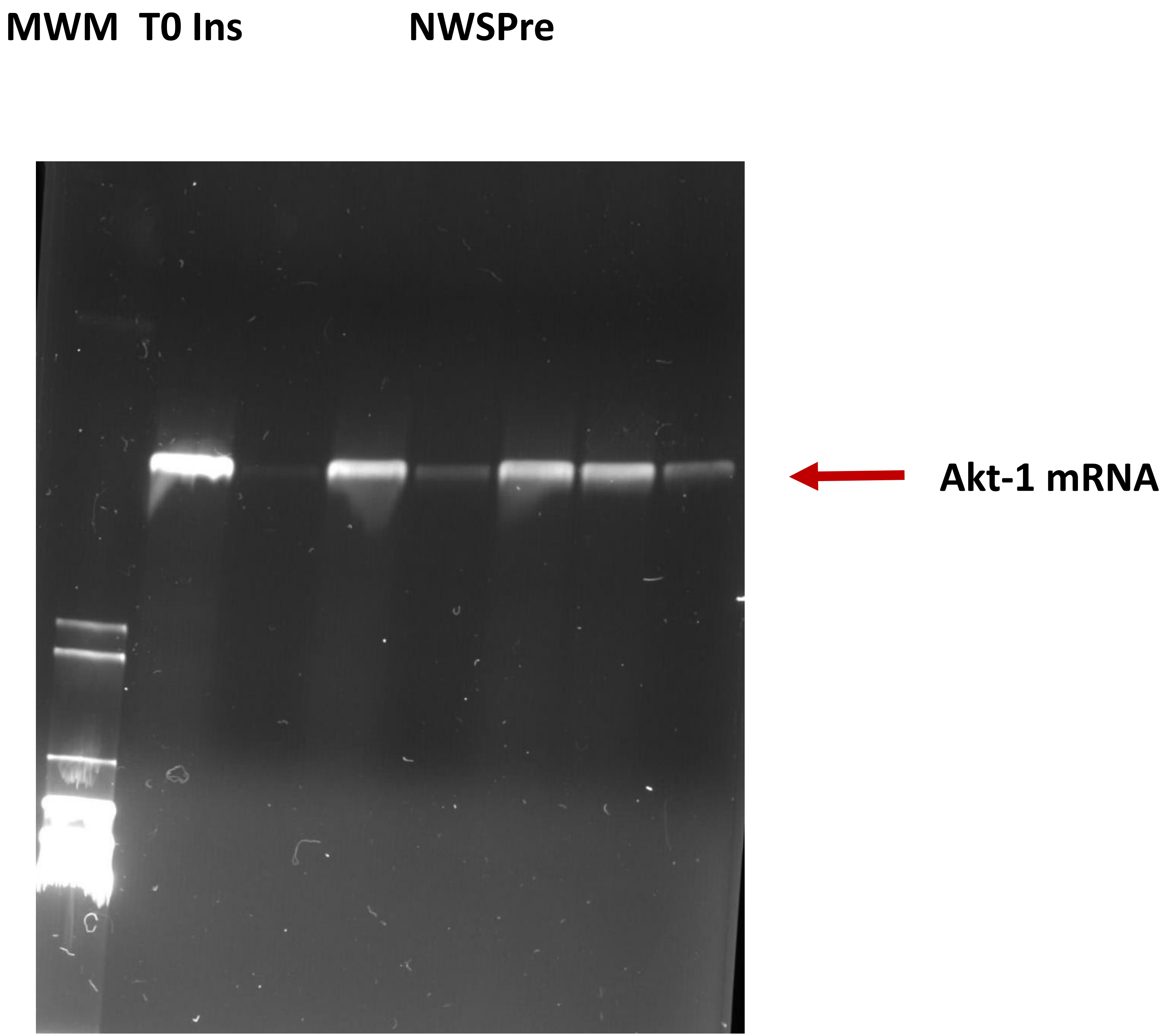

Supplementary Figure 5

Akt-1 mRNA OSPre

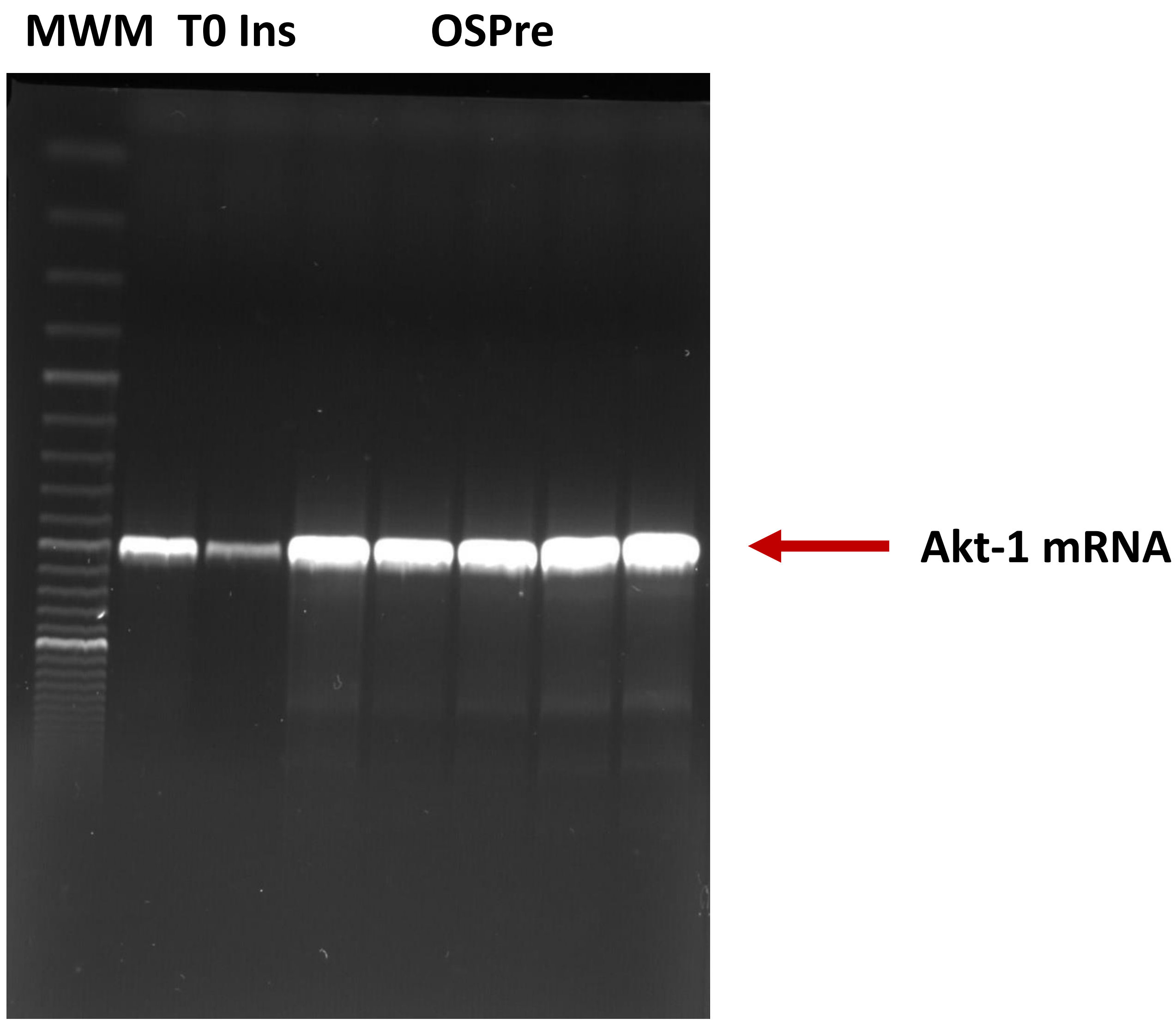

Supplementary Figure 5

Akt-1 mRNA OSPost and NWSPost

MWM T0 Ins OSPostNWSPost

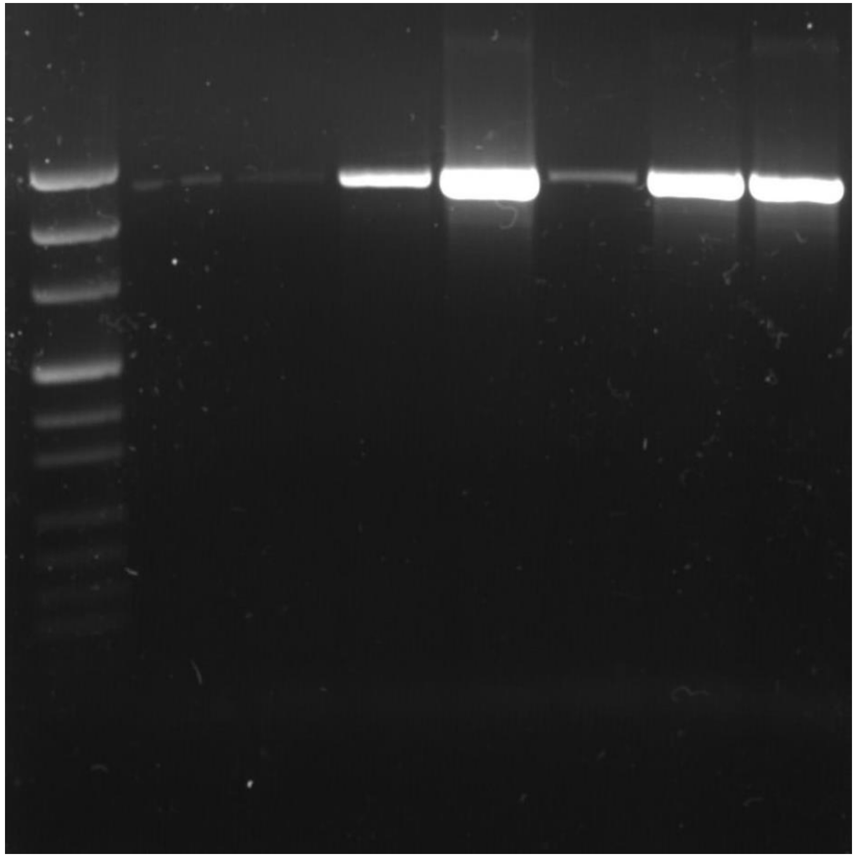

Akt-1 mRNA

Supplementary Figure 5

Akt-2 mRNA NWSPre

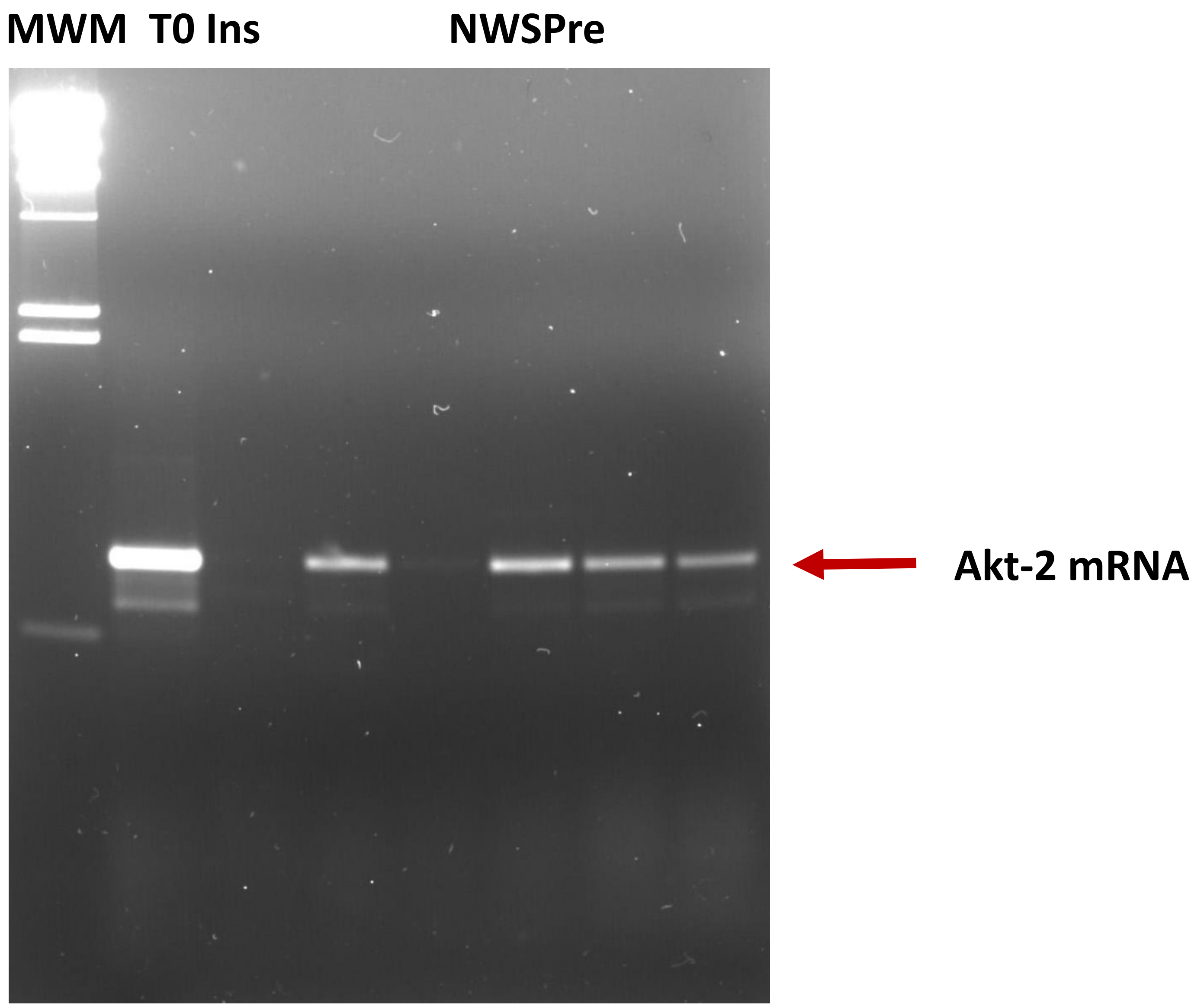

Supplementary Figure 5

Akt-2 mRNA OSPre

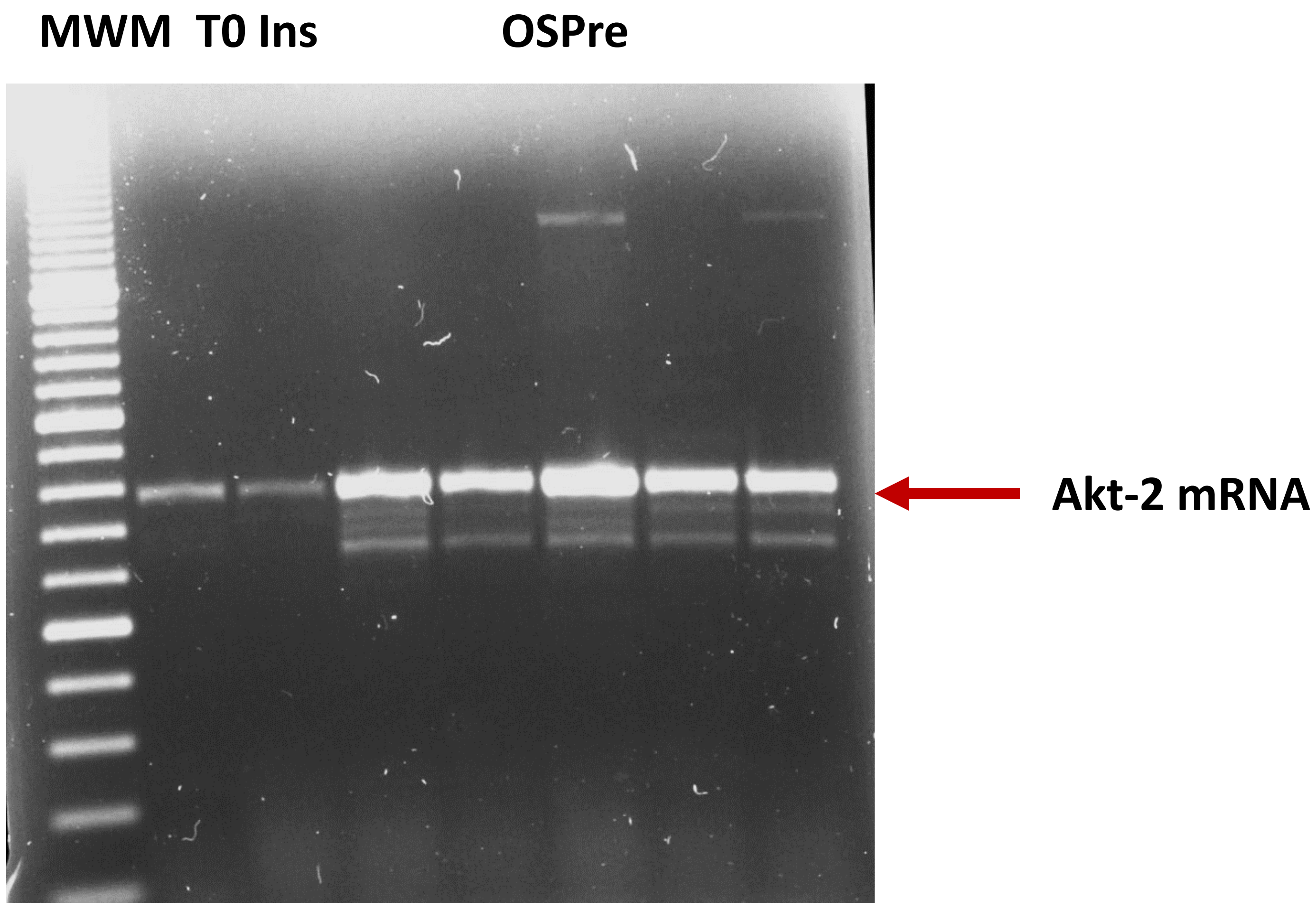

Supplementary Figure 5

Akt-2 mRNA OSPost and NWSPost

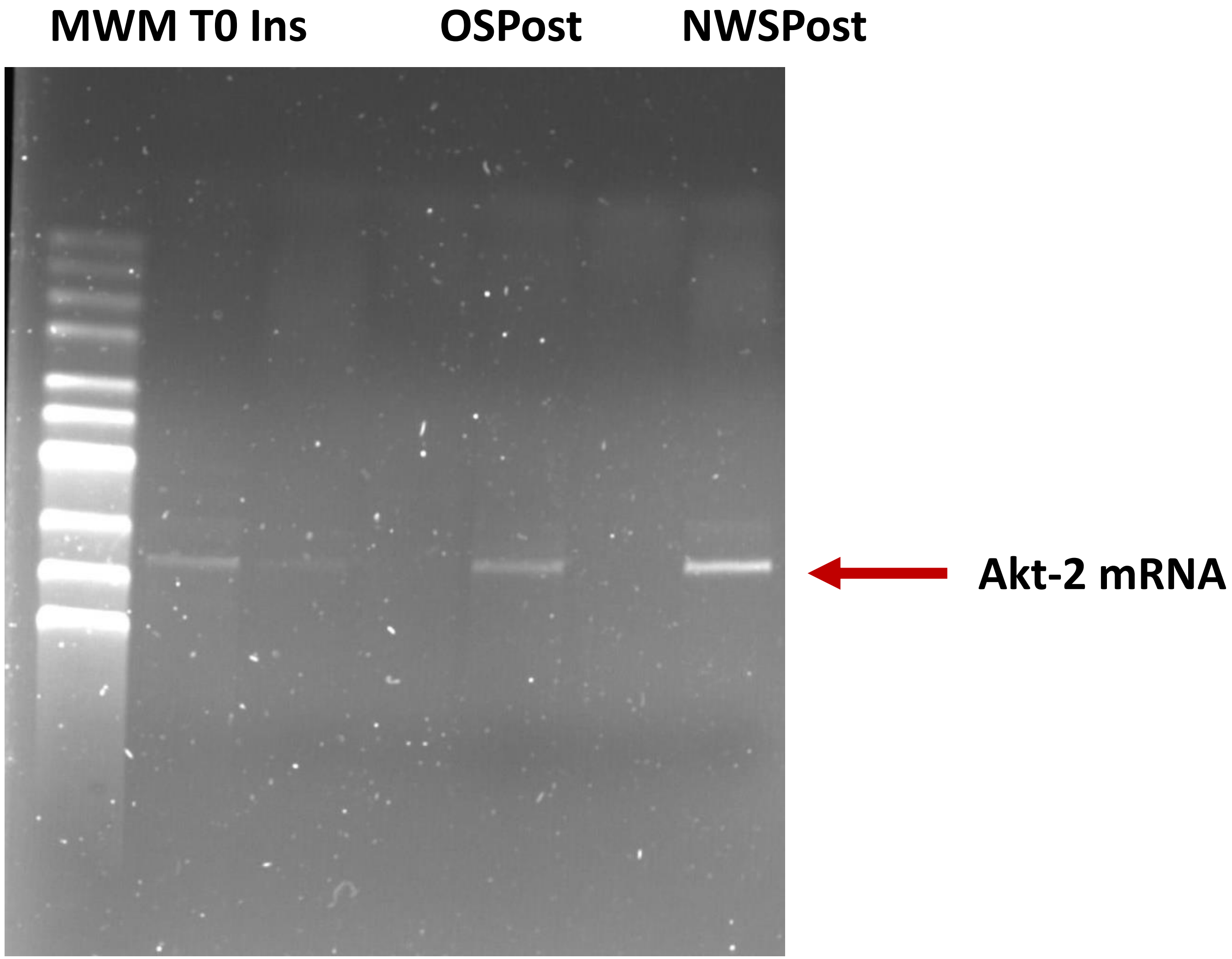

Supplementary Figure 5

Akt-3 mRNA NWSPre

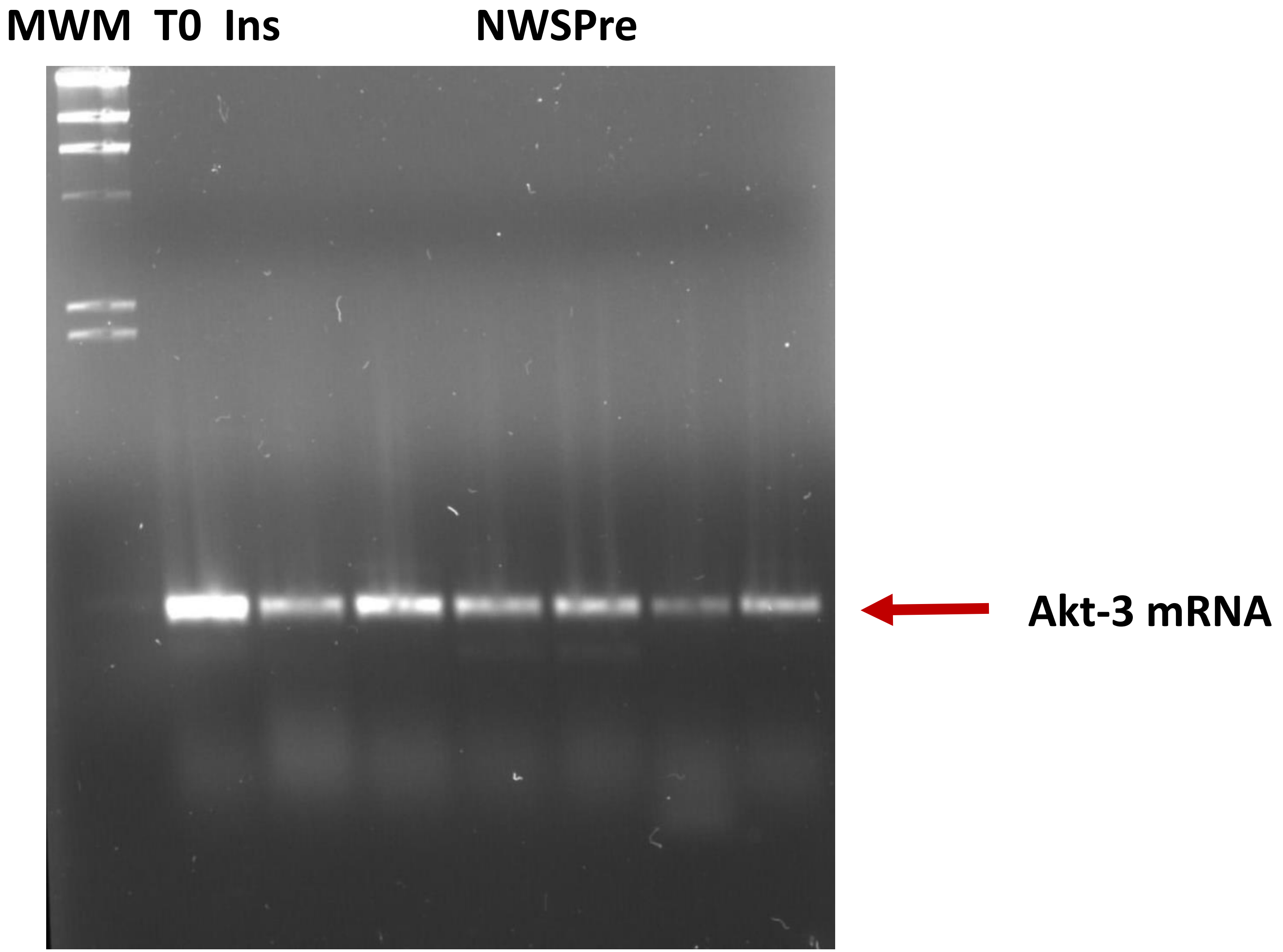

Supplementary Figure 5

Akt-3 mRNA OSPre

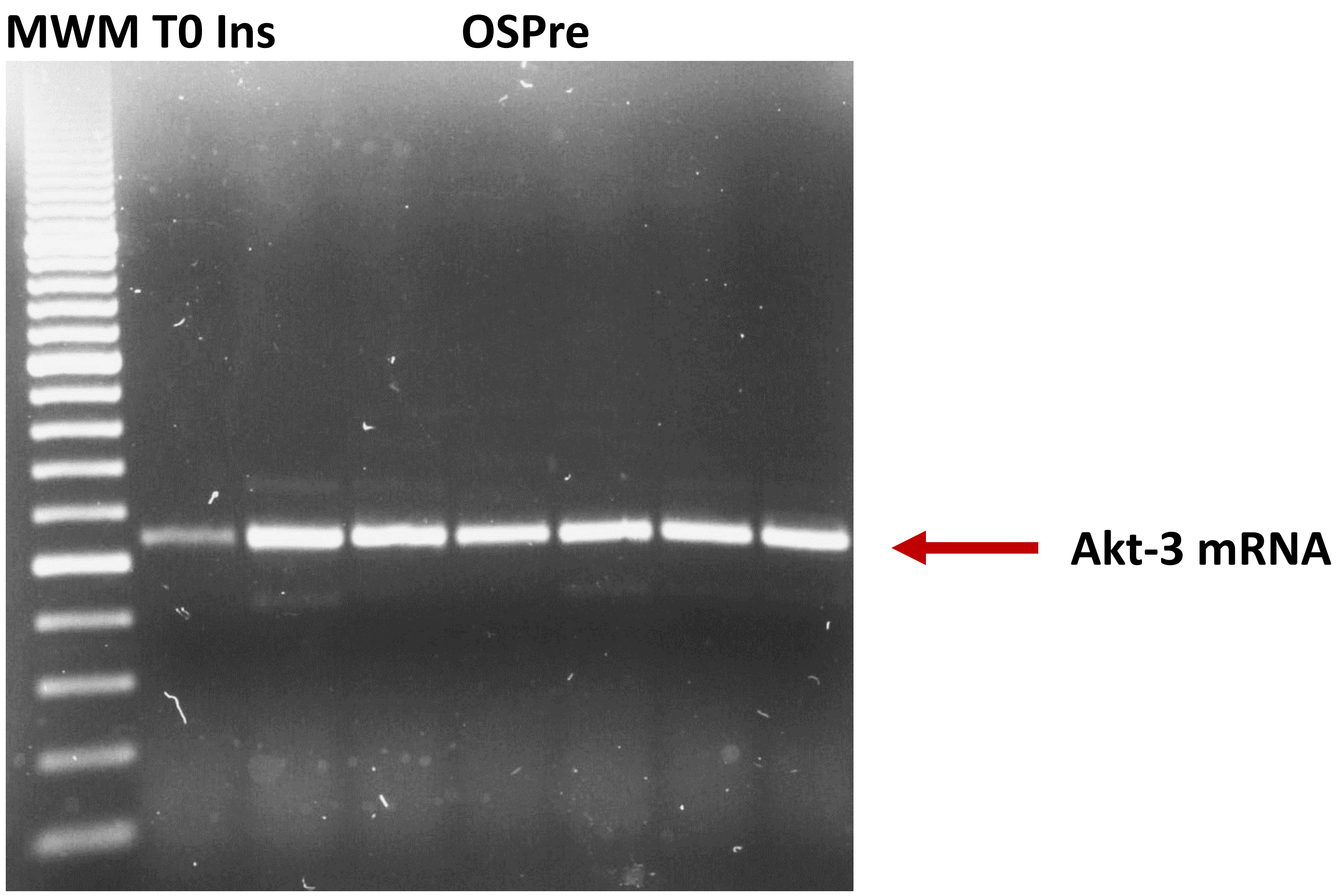

Supplementary Figure 5

Akt-3 mRNA OSPost and NWSPost

MWM T0 Ins OSPost NWSPost

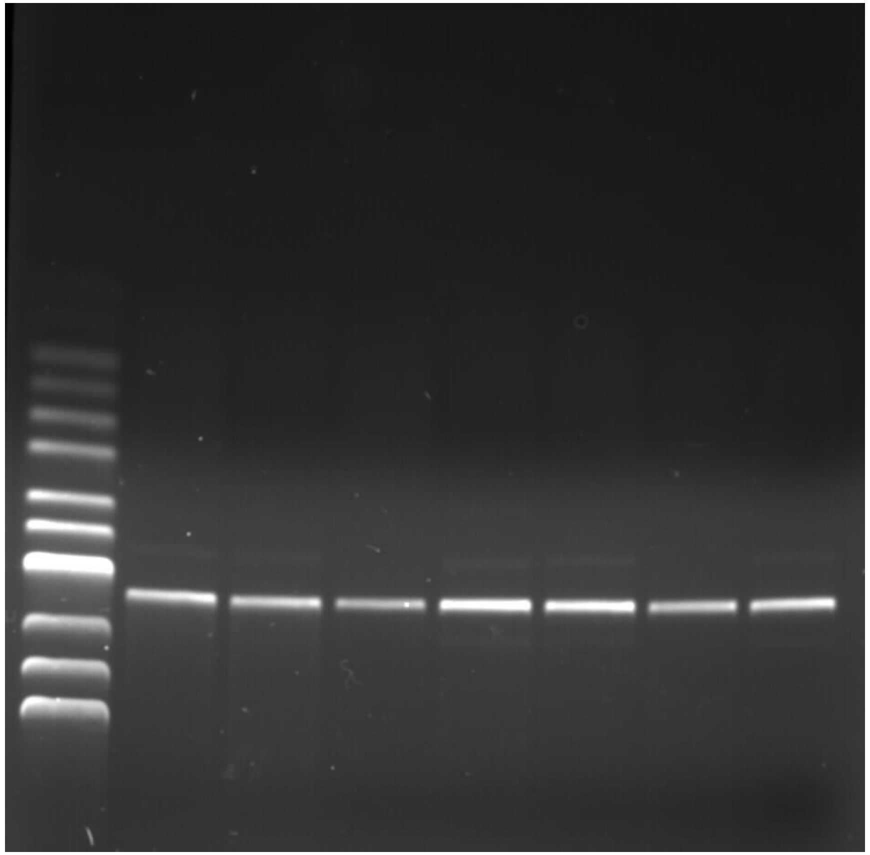

Akt-3 mRNA

Supplementary Figure 5

PPIA mRNA NWSPre

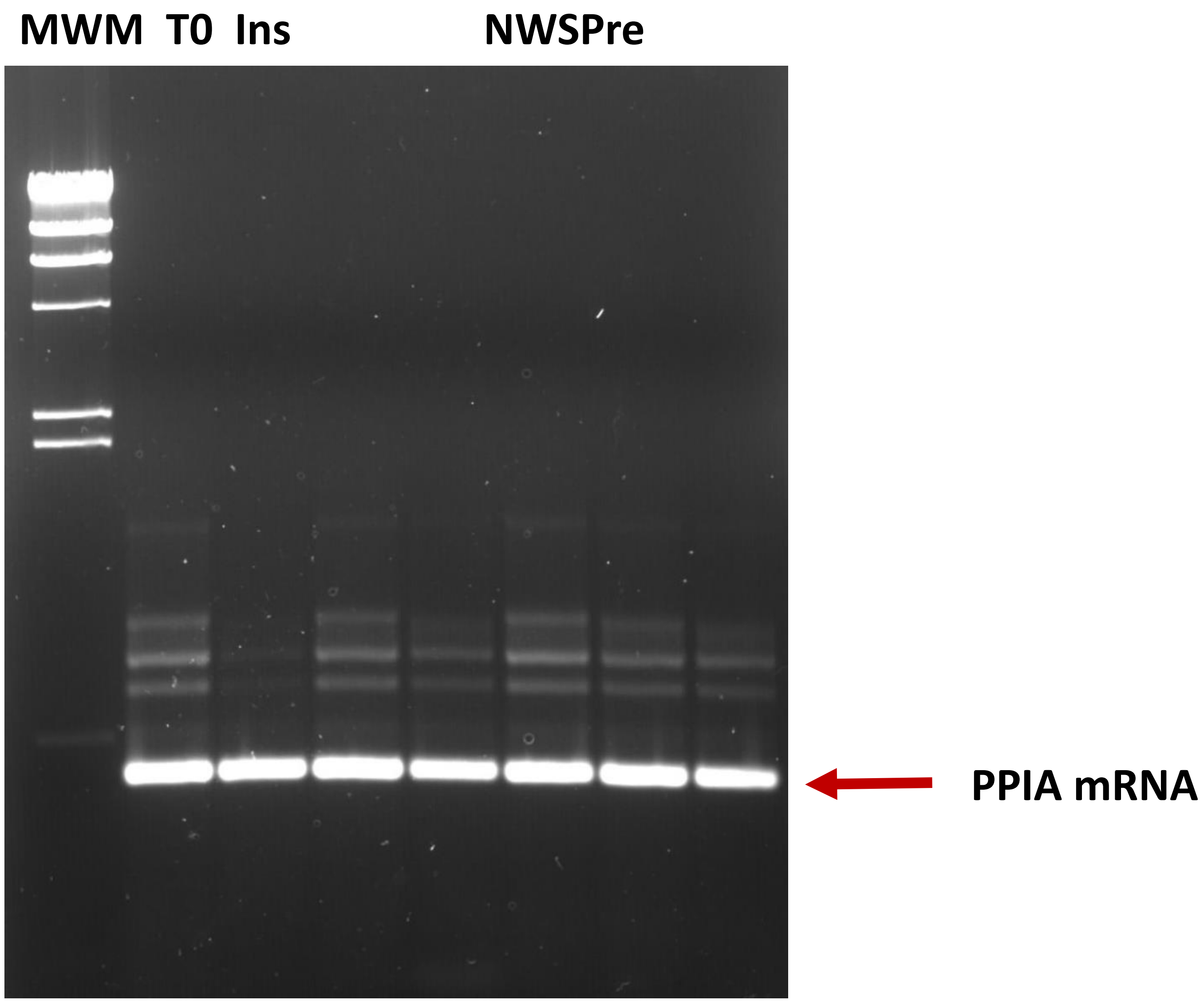

Supplementary Figure 5

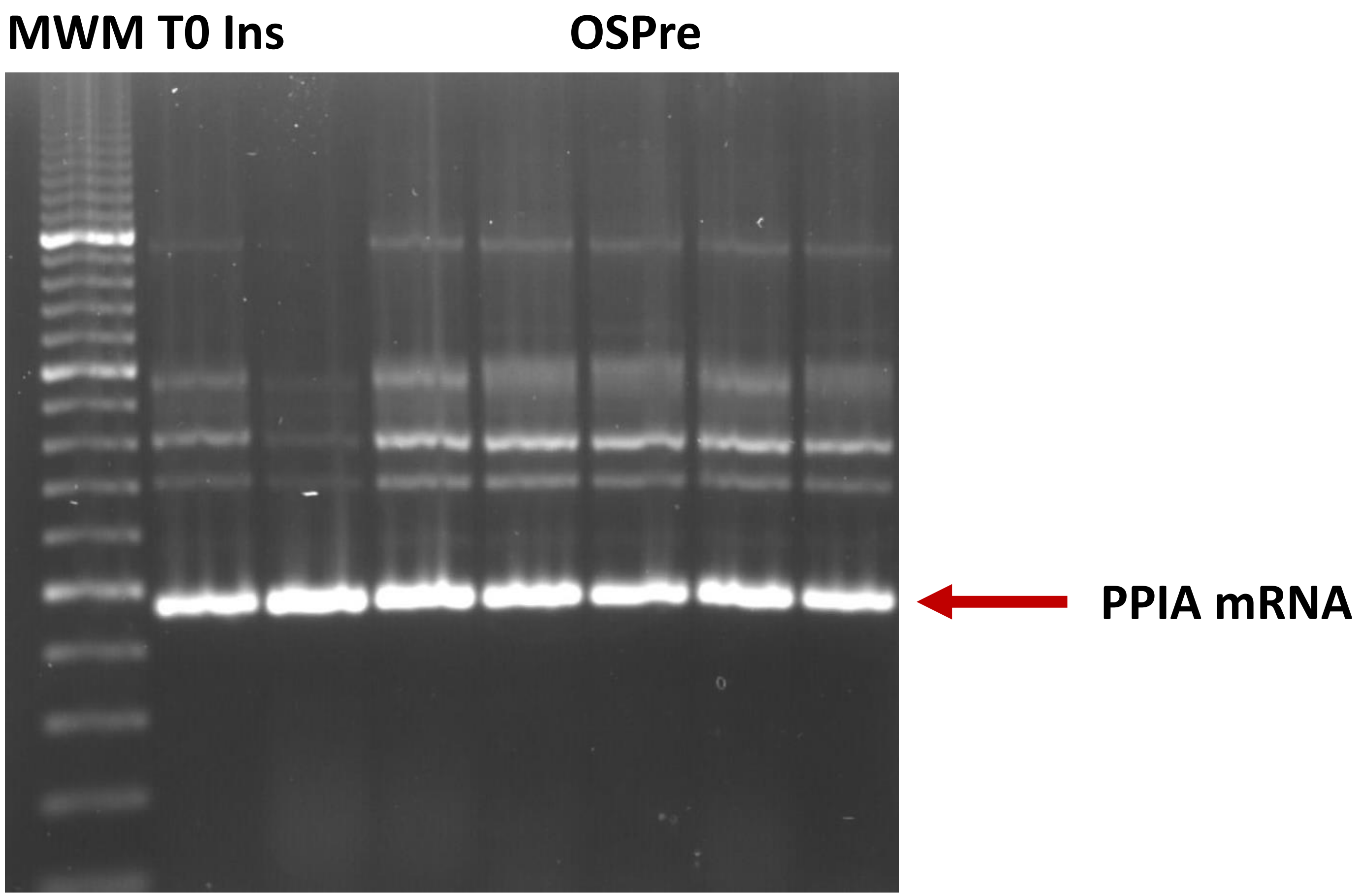

Supplementary Figure 5

PPIA mRNA OSPost and NWSPost

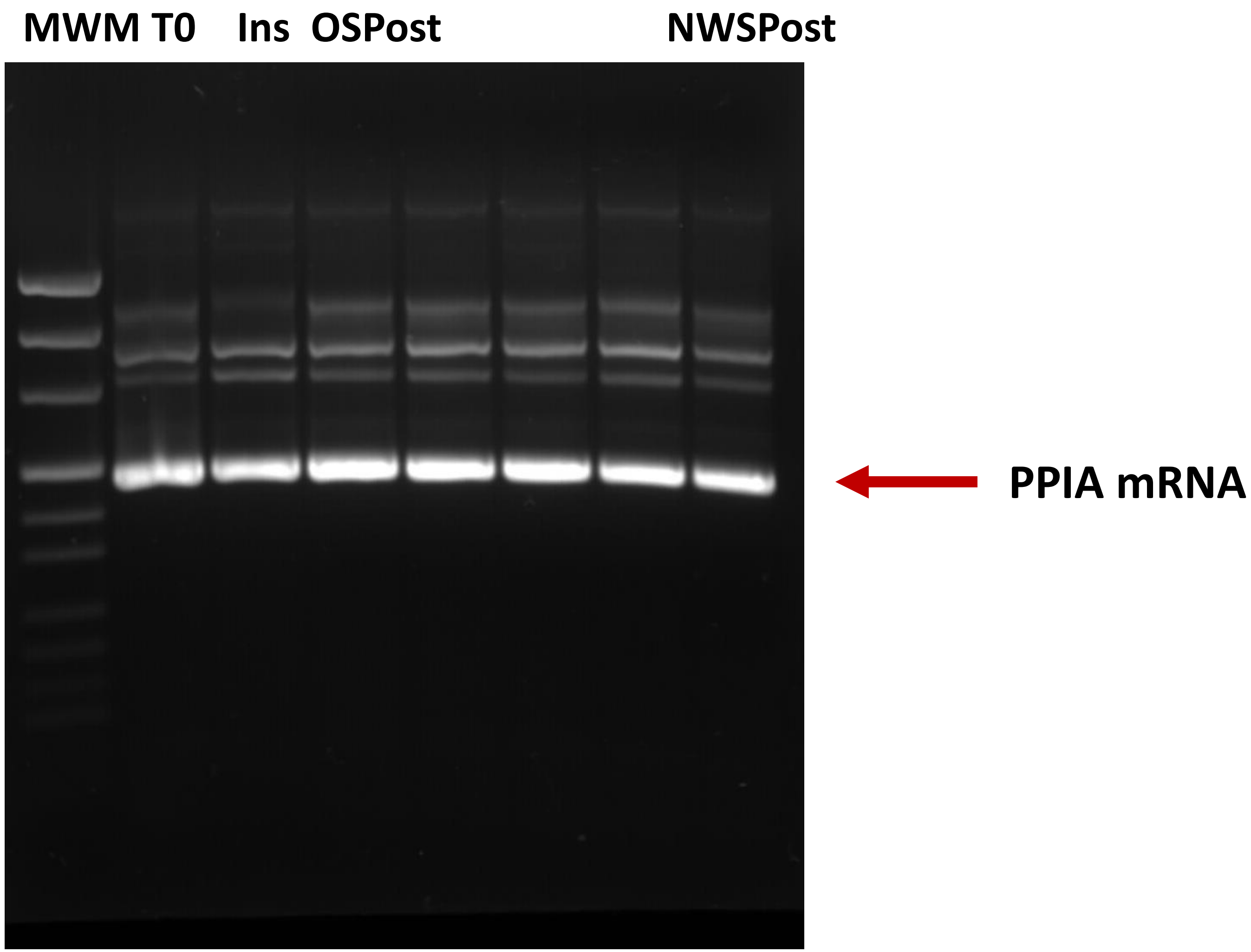

Supplement: S1 Raw images — (PDF) [file pone.0266073.s010.pdf]
